# Supplementary material for: RNF20-mediated H2B monoubiquitination protects stalled forks from degradation and promotes fork restart
Source: EMBO Rep. 2025 Jun 10;26(15):3773–803. doi: 10.1038/s44319-025-00497-3 (PMC12331980; doi:10.1038/s44319-025-00497-3)
Supplement: Supplementary file 1 — Table EV1 [file 44319_2025_497_MOESM1_ESM.docx]

**Table EV 1. List of shRNA sequences used in this study**

| **shRNA** | **Sequence** | **References** |
| --- | --- | --- |
| RNF20 #1 | GGGGTGAGAGCTGGAATCTCTGC | This paper |
| RNF20 #2 | GAAGGCAGCTGTTGAAGATTC | 1 |
| RAD51C | CACCTTCTGTTCAGCACTAGA | 2 |
| XRCC2 | TTGCAACGACACAAACTATAA | 3 |
| XRCC3 | GAATTATTGCTGCAATTAA | 1 |
| SMARCAL1 | GCTTTGACCTTCTTAGCAAT | 4 |
| ZRANB3 | TGGTGTGTGTCAGCTCTGT | 5 |
| HLTF | GGAATATAATGTTAACGAT | 6 |

**References**

1. Wu C, Cui Y, Liu X, Zhang F, Lu LY, Yu X (2020) The RNF20/40 complex regulates p53-dependent gene transcription and mRNA splicing. J Mol Cell Biol 12: 113-124
2. Somyajit K, Basavaraju S, Scully R, Nagaraju G (2013) ATM- and ATR-mediated phosphorylation of XRCC3 regulates DNA double-strand break-induced checkpoint activation and repair. Mol Cell Biol 33: 1830-44
3. Saxena S, Somyajit K, Nagaraju G (2018) XRCC2 Regulates Replication Fork Progression during dNTP Alterations. Cell Rep 25: 3273-3282 e6
4. Postow L, Woo EM, Chait BT, Funabiki H (2009) Identification of SMARCAL1 as a component of the DNA damage response. J Biol Chem 284: 35951-61
5. Ciccia A, Nimonkar AV, Hu Y, Hajdu I, Achar YJ, Izhar L, Petit SA, Adamson B, Yoon JC, Kowalczykowski SC, Livingston DM, Haracska L, Elledge SJ (2012) Polyubiquitinated PCNA recruits the ZRANB3 translocase to maintain genomic integrity after replication stress. Mol Cell 47: 396-409
6. Kang Z, Fu P, Alcivar AL, Fu H, Redon C, Foo TK, Zuo Y, Ye C, Baxley R, Madireddy A, Buisson R, Bielinsky AK, Zou L, Shen Z, Aladjem MI, Xia B (2021) BRCA2 associates with MCM10 to suppress PRIMPOL-mediated repriming and single-stranded gap formation after DNA damage. Nat Commun 12: 5966
