# Supplementary material for: RNF20-mediated H2B monoubiquitination protects stalled forks from degradation and promotes fork restart
Source: EMBO Rep. 2025 Jun 10;26(15):3773–803. doi: 10.1038/s44319-025-00497-3 (PMC12331980; doi:10.1038/s44319-025-00497-3)
Supplement: Supplementary file 2 — Table EV2 [file 44319_2025_497_MOESM2_ESM.docx]

**Table EV 2. List of primer sequences used for generating WT and mutant RNF20 constructs**

| **Primer name** | **Orientation** | **Sequence** |
| --- | --- | --- |
| WT-RNF20 | Forward | GCGCAACGCTTATGTCAGGAATTGGAAATAAAAG |
|  | Reverse | GCGCGGATCCTTACTTATCGTCGTCATCCTTGTAATCACCAATGTAGATGCGATGAAAATC |
| C922S-RNF20 | Forward | GATTACAAGGCACGCTTGACCTCTCCGTGCTGTAACATGCGT |
|  | Reverse | ACGCATGTTACAGCACGGAGAGGTCAAGCGTGCCTTGTAATC |
| C960A-RNF20 | Forward | GCCAGCGCAAATGTCCCAAGGCTAATGCTGCTTTTGGTGC |
|  | Reverse | GCACCAAAAGCAGCATTAGCCTTGGGACATTTGCGCTGGC |
| S172A-RNF20 | Forward | CCAGTGAAGAGATGGAGGCTCAGCTGCAGGAACGTGTGG |
|  | Reverse | CCACACGTTCCTGCAGCTGAGCCTCCATCTCTTCACTGG |
| S553A-RNF20 | Forward | CCAGTCCTCAGCTTCAAAGGCAGCTCAGGAGGATGCCAATGAAATC |
|  | Reverse | GATTTCATTGGCATCCTCCTGAGCTGCCTTTGAAGCTGAGGACTGG |
