# Supplementary figures and images for: RNF20-mediated H2B monoubiquitination protects stalled forks from degradation and promotes fork restart (part 1 of 3)
Source: EMBO Rep. 2025 Jun 10;26(15):3773–803. doi: 10.1038/s44319-025-00497-3 (PMC12331980; doi:10.1038/s44319-025-00497-3)

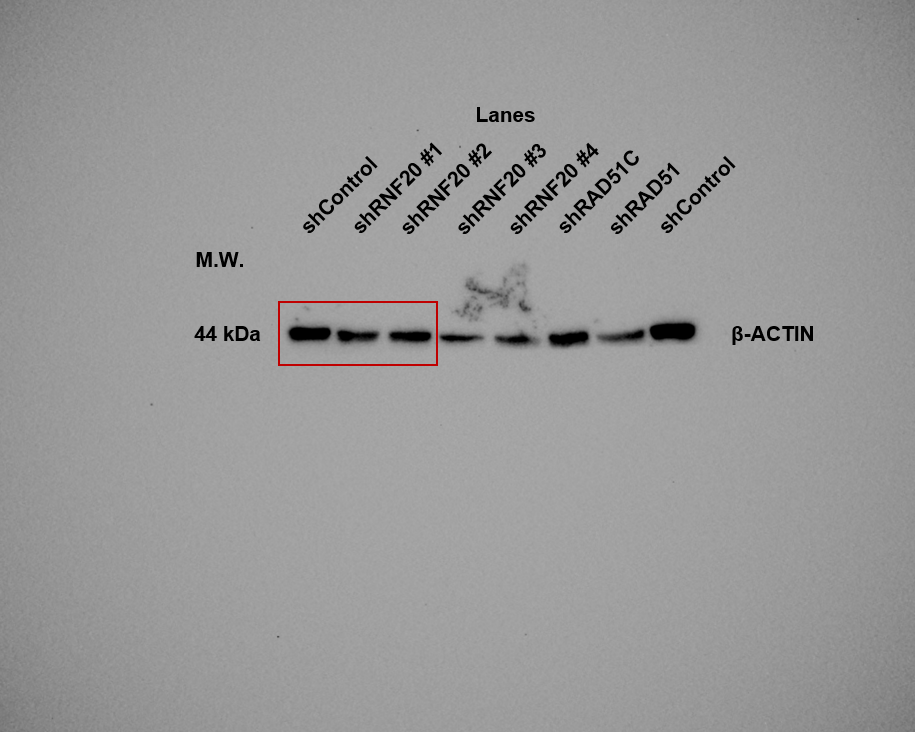

Supplement: Supplementary file 4 — Source data Fig. 1 [file 44319_2025_497_MOESM4_ESM.zip › 1A/ACTIN western.tif]

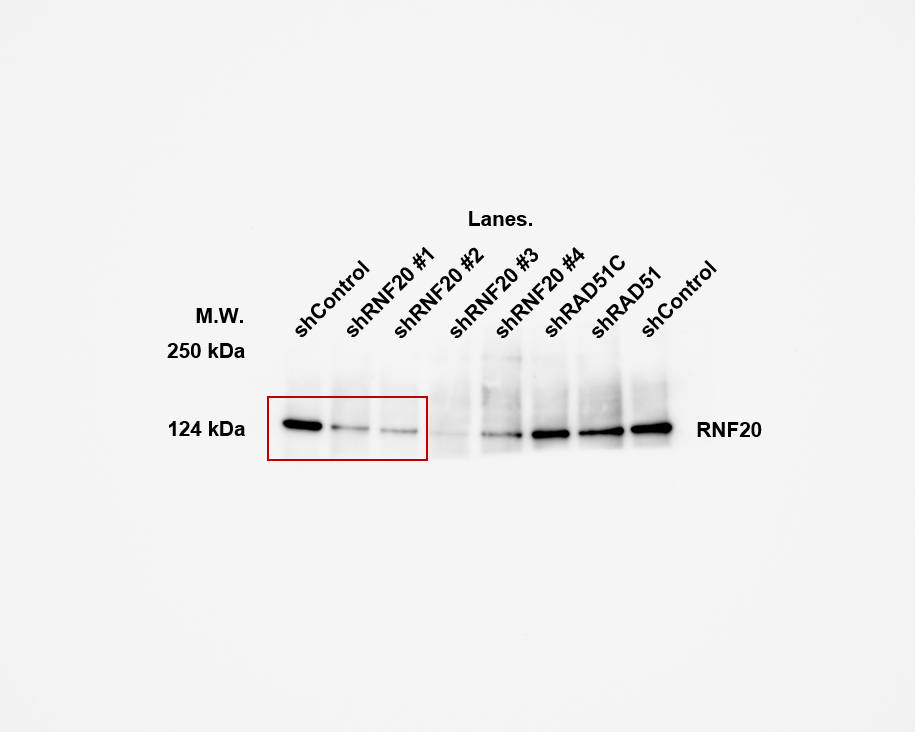

Supplement: Supplementary file 4 — Source data Fig. 1 [file 44319_2025_497_MOESM4_ESM.zip › 1A/RNF20 western.tif]

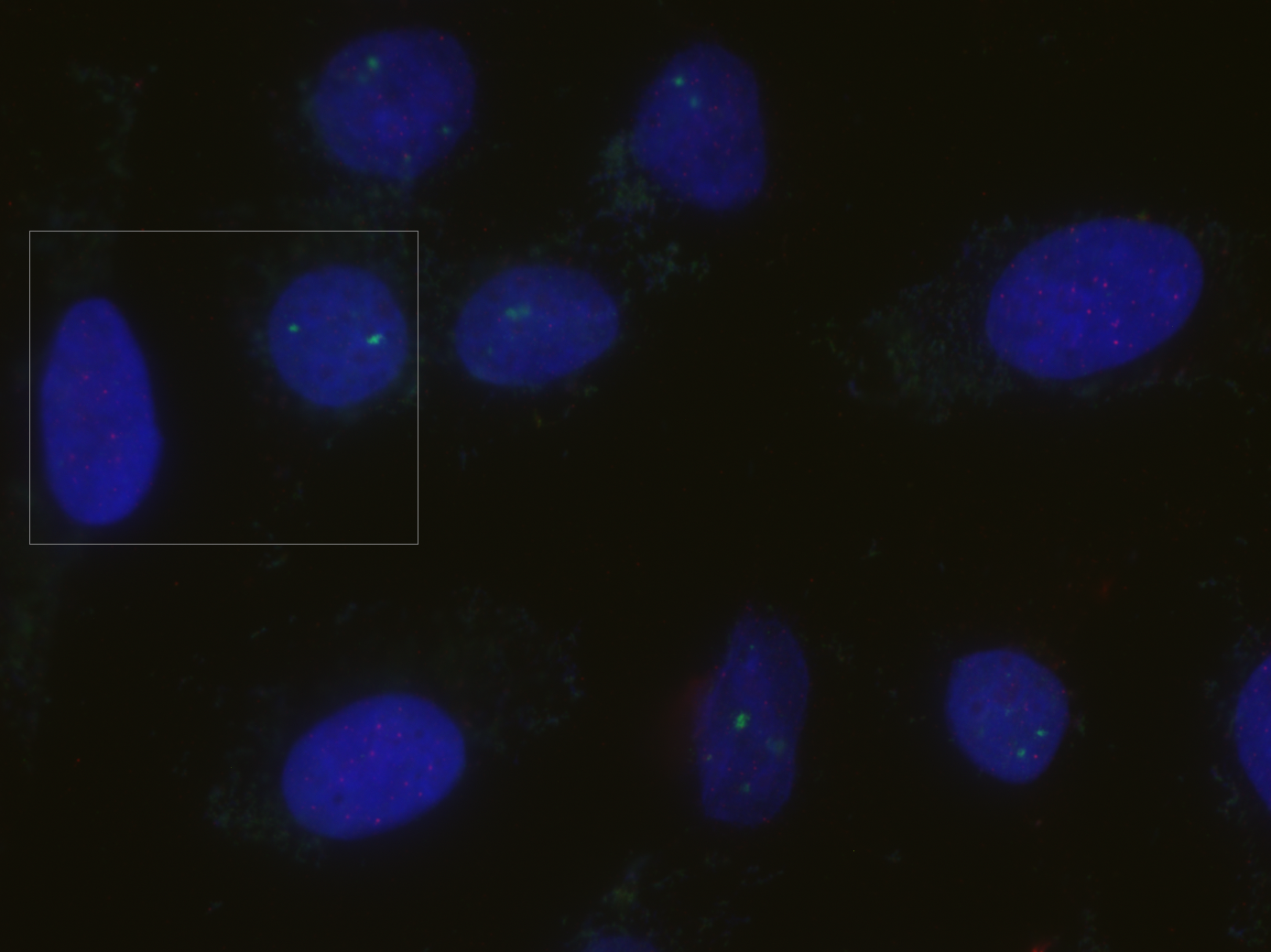

Supplement: Supplementary file 4 — Source data Fig. 1 [file 44319_2025_497_MOESM4_ESM.zip › 1B and 1C/shControl 53BP1 nuclear bodies.tif]

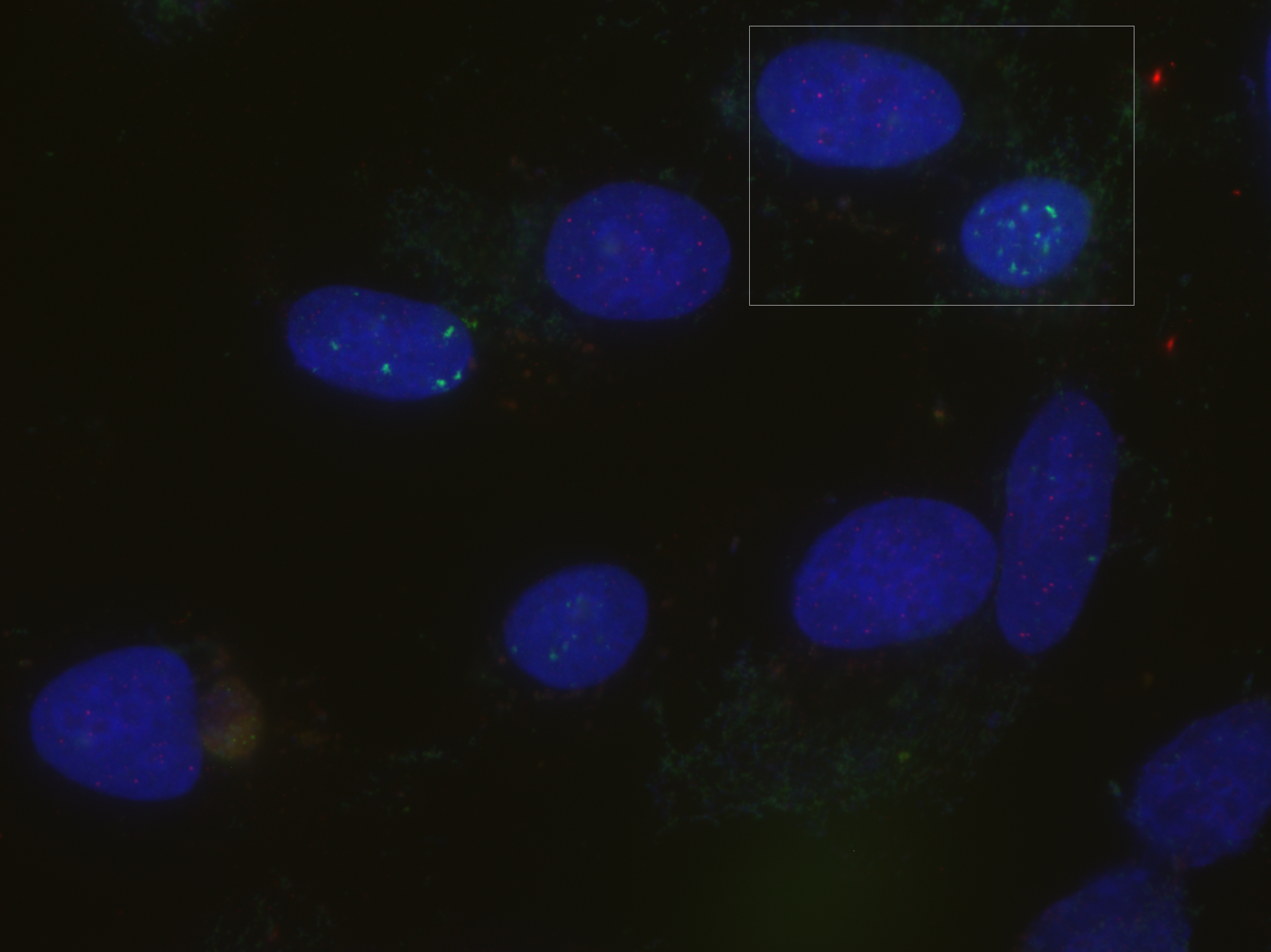

Supplement: Supplementary file 4 — Source data Fig. 1 [file 44319_2025_497_MOESM4_ESM.zip › 1B and 1C/shRNF20 53BP1 nuclear bodies.tif]

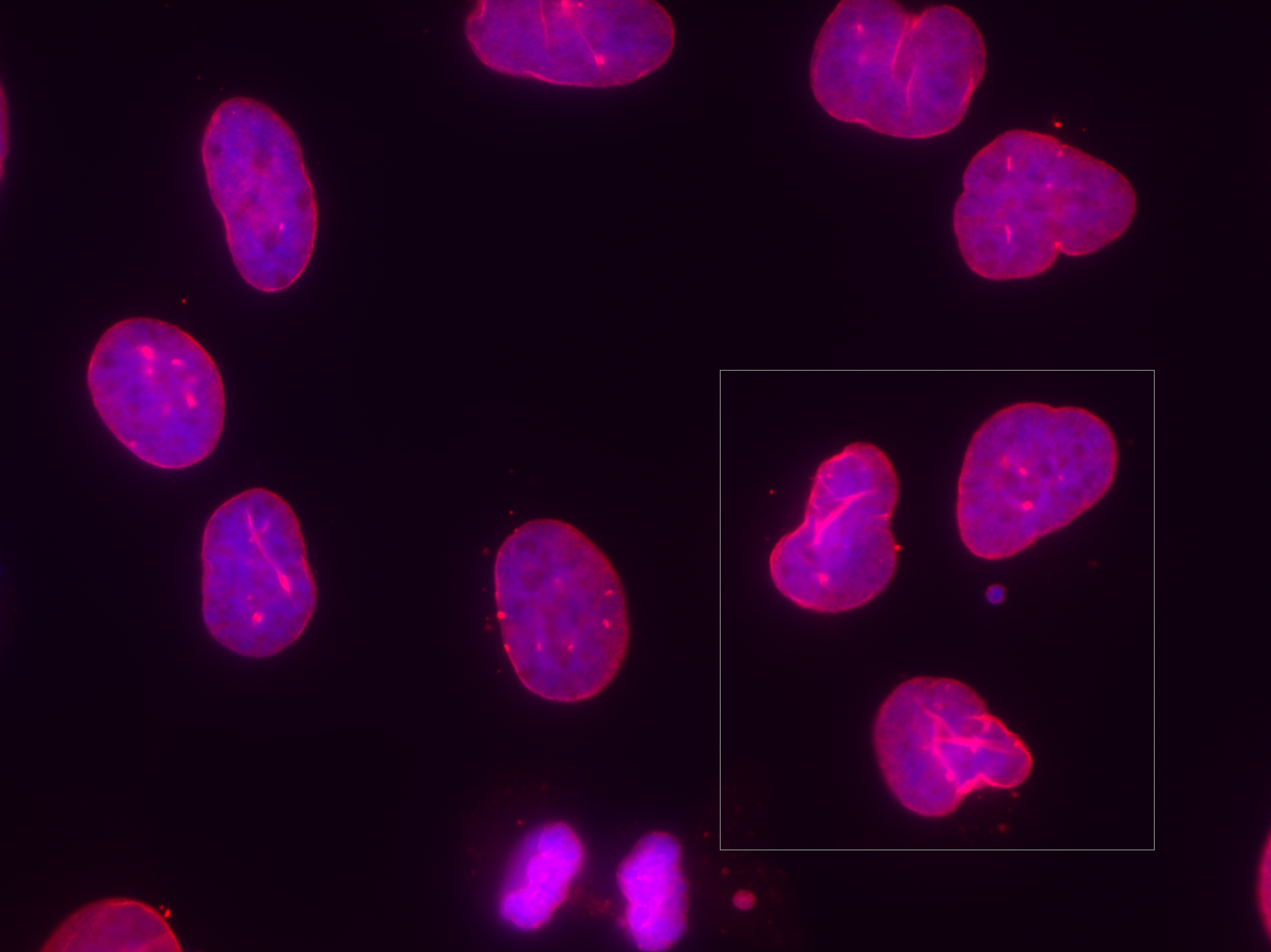

Supplement: Supplementary file 4 — Source data Fig. 1 [file 44319_2025_497_MOESM4_ESM.zip › 1D and 1E/shControl APH micronuclei.tif]

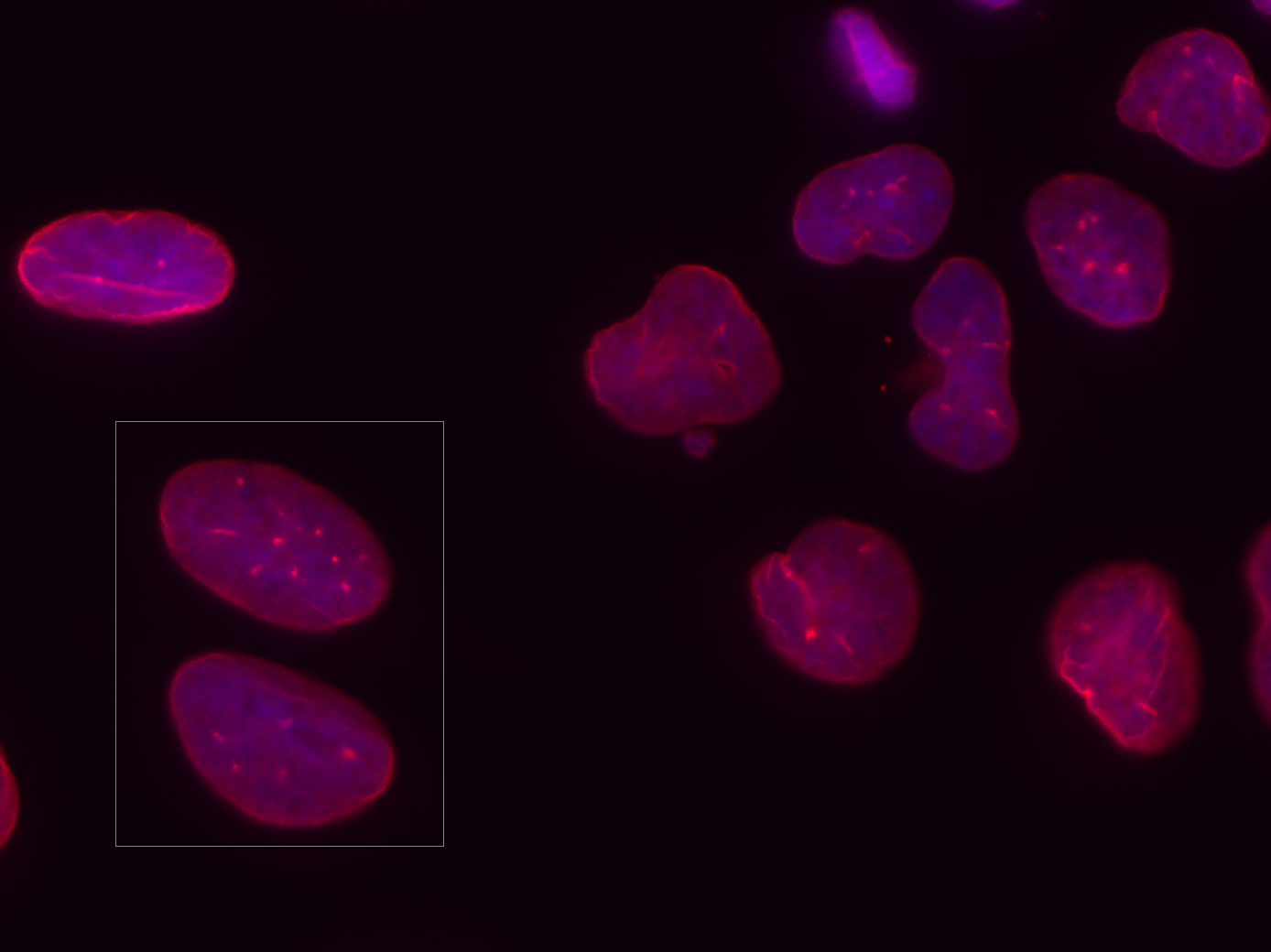

Supplement: Supplementary file 4 — Source data Fig. 1 [file 44319_2025_497_MOESM4_ESM.zip › 1D and 1E/shControl UT micronuclei.tif]

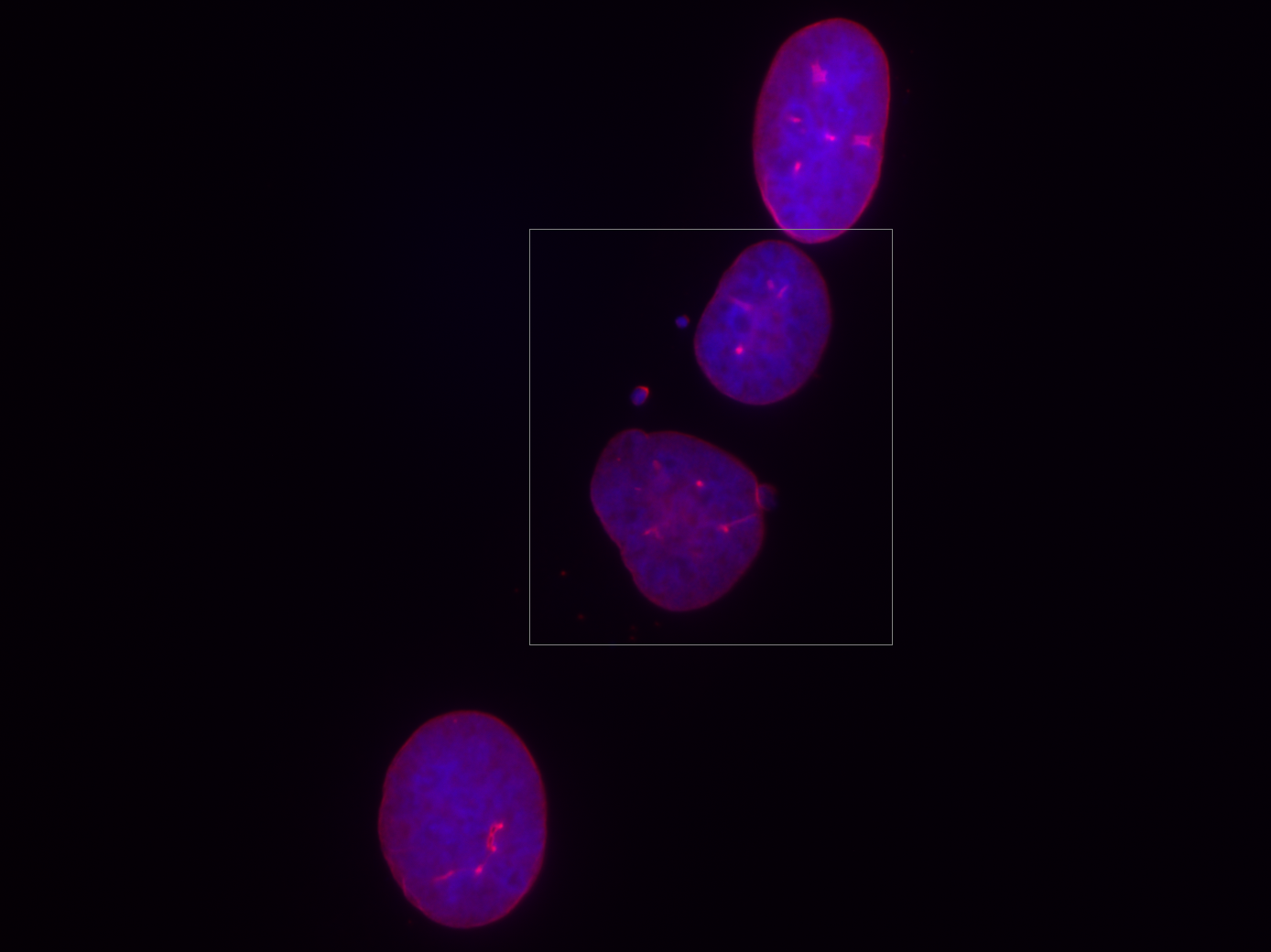

Supplement: Supplementary file 4 — Source data Fig. 1 [file 44319_2025_497_MOESM4_ESM.zip › 1D and 1E/shRNF APH micronuclei.tif]

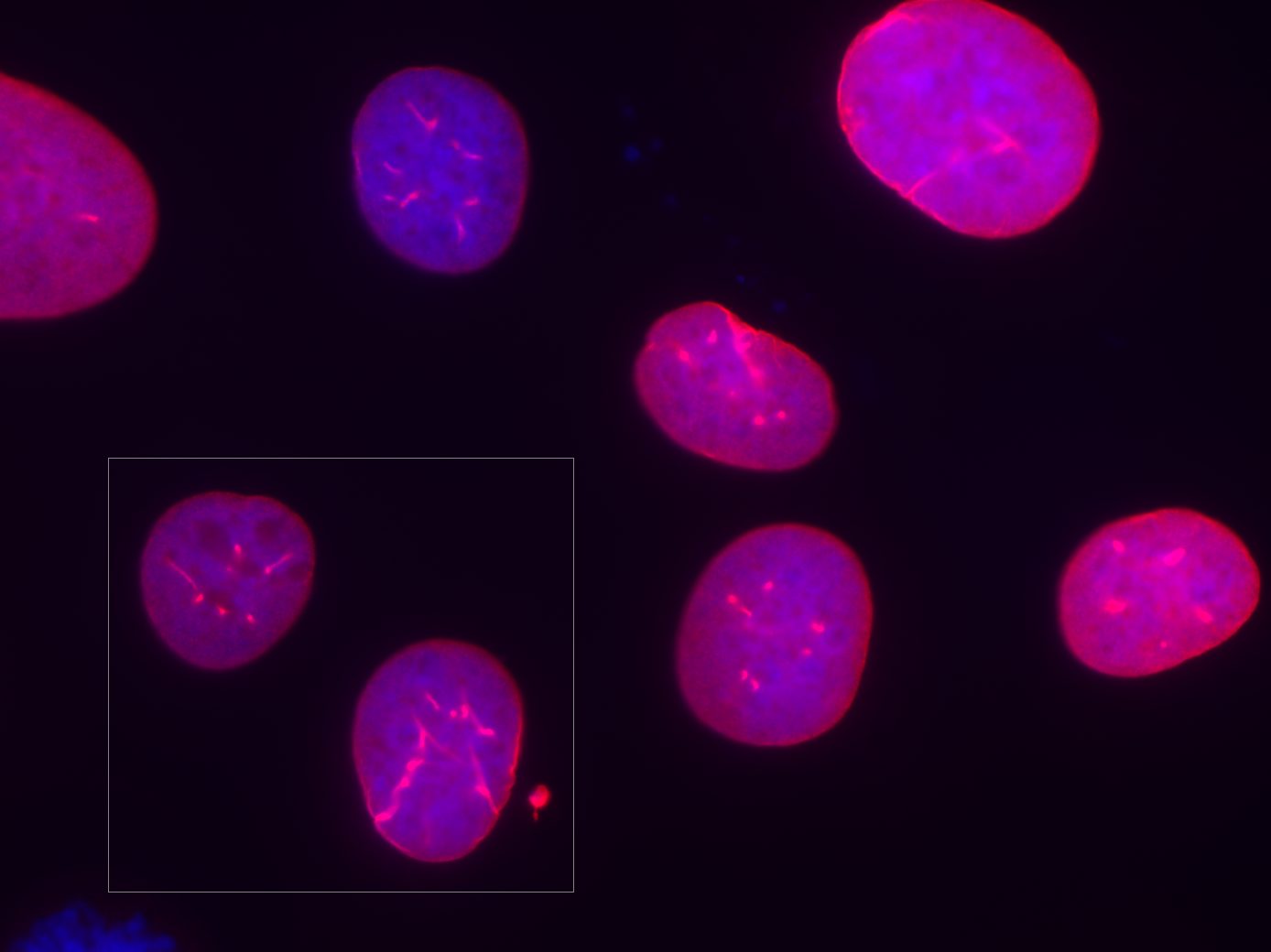

Supplement: Supplementary file 4 — Source data Fig. 1 [file 44319_2025_497_MOESM4_ESM.zip › 1D and 1E/shRNF20 UT micronuclei.tif]

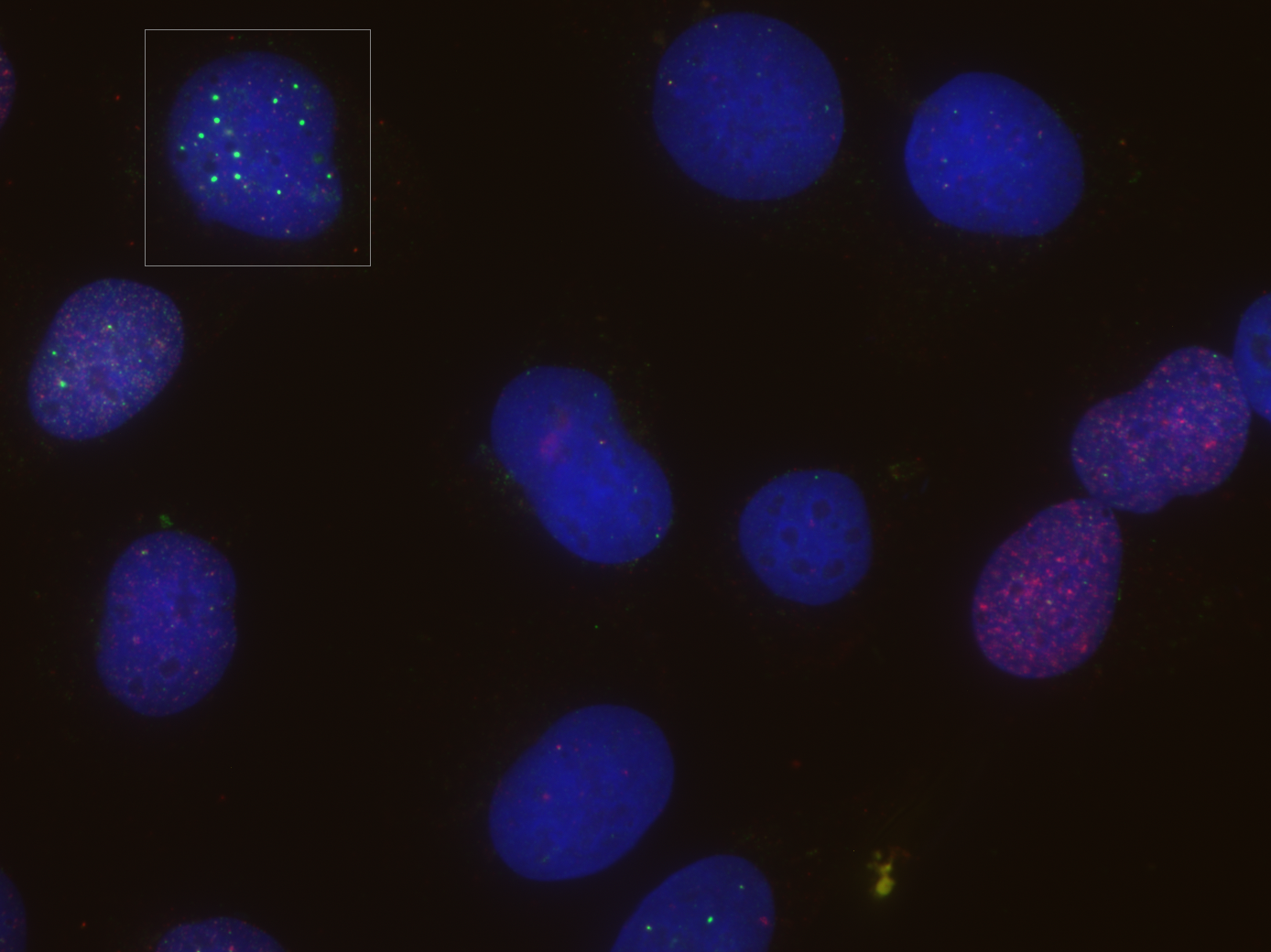

Supplement: Supplementary file 4 — Source data Fig. 1 [file 44319_2025_497_MOESM4_ESM.zip › 1F and 1G/shControl HU RPA70.tif]

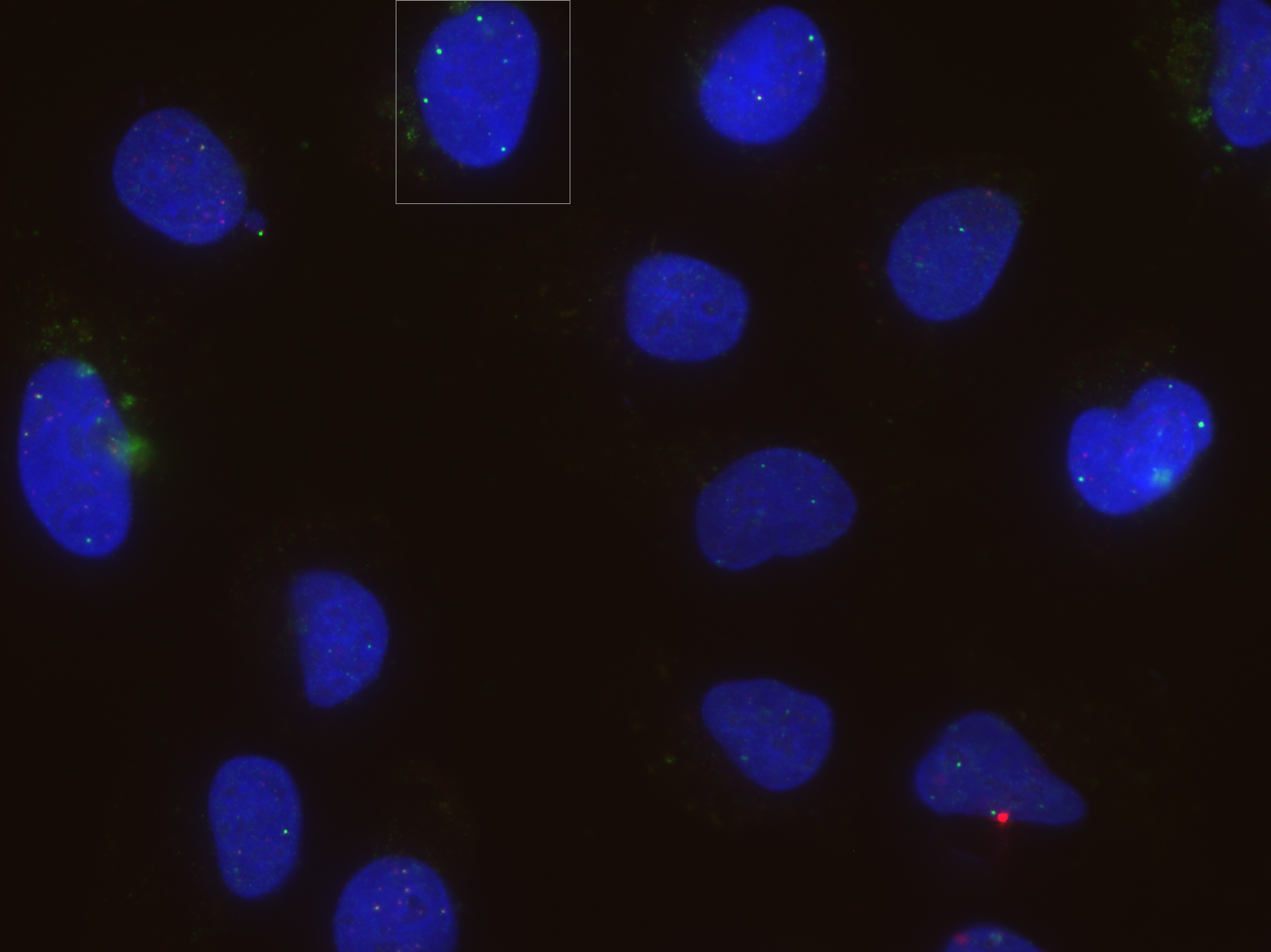

Supplement: Supplementary file 4 — Source data Fig. 1 [file 44319_2025_497_MOESM4_ESM.zip › 1F and 1G/shControl UT RPA70.tif]

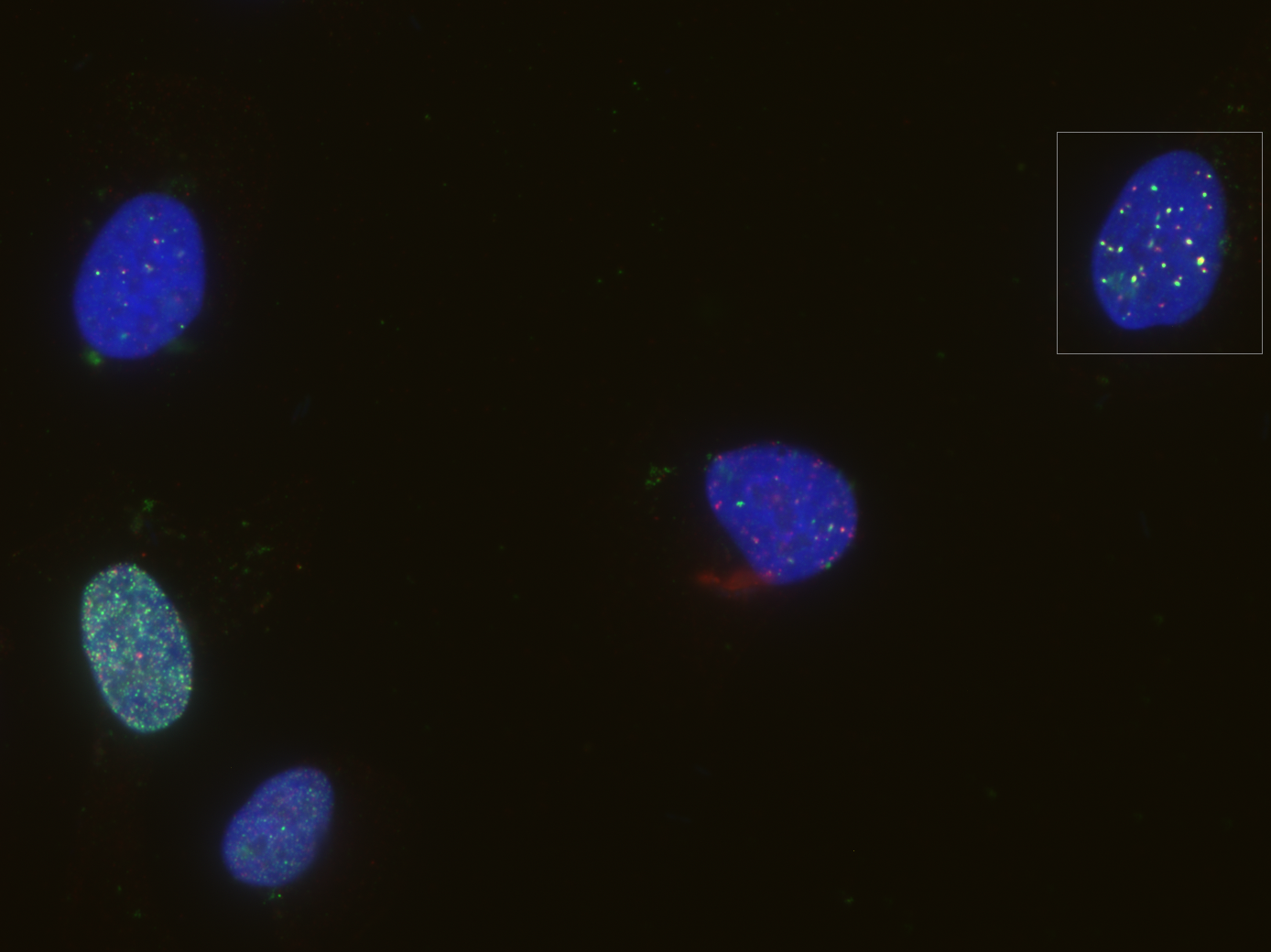

Supplement: Supplementary file 4 — Source data Fig. 1 [file 44319_2025_497_MOESM4_ESM.zip › 1F and 1G/shRNF20 HU RPA70.tif]

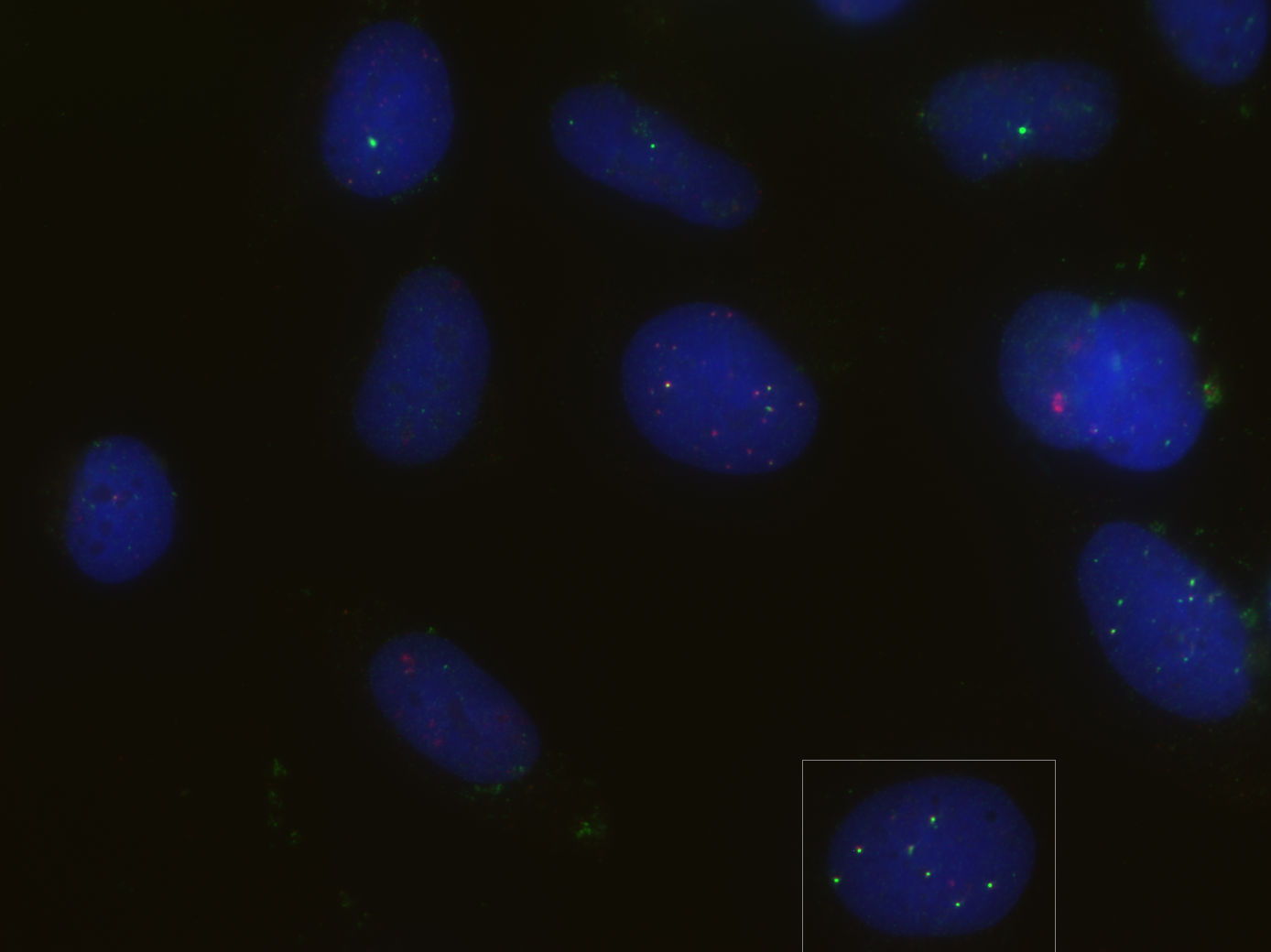

Supplement: Supplementary file 4 — Source data Fig. 1 [file 44319_2025_497_MOESM4_ESM.zip › 1F and 1G/shRNF20 UT RPA70.tif]

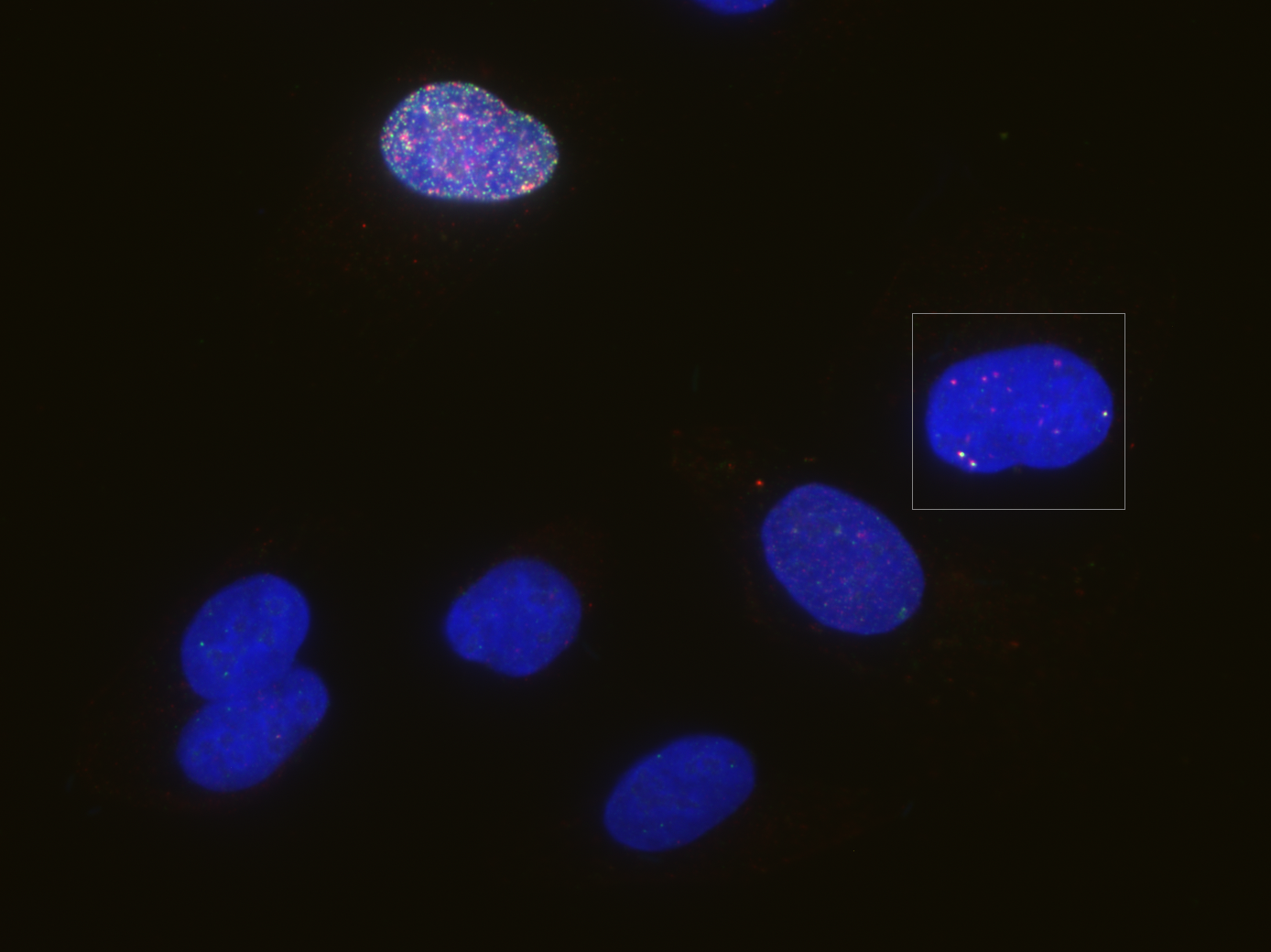

Supplement: Supplementary file 4 — Source data Fig. 1 [file 44319_2025_497_MOESM4_ESM.zip › 1H and 1I/shControl HU gH2AX.tif]

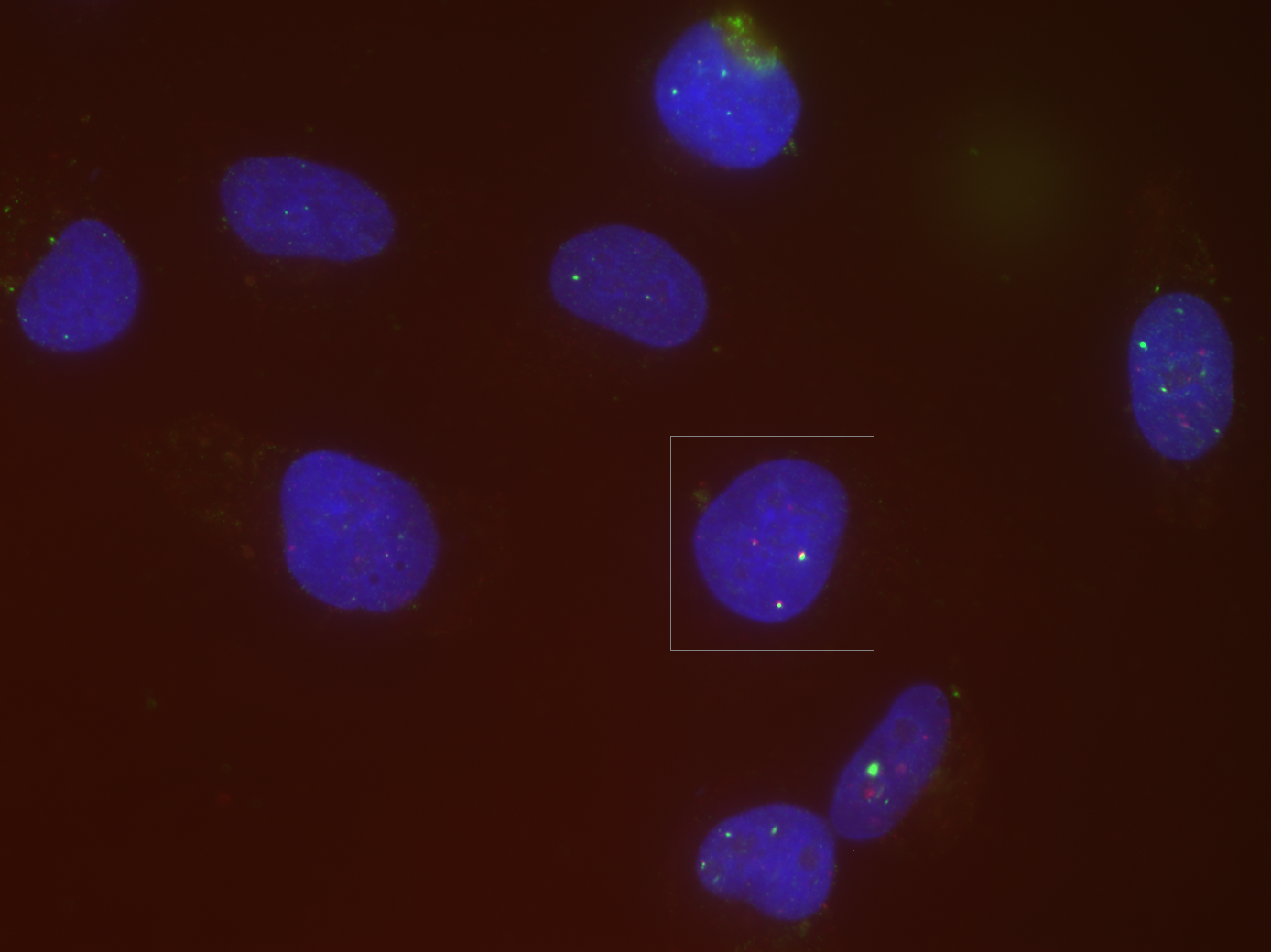

Supplement: Supplementary file 4 — Source data Fig. 1 [file 44319_2025_497_MOESM4_ESM.zip › 1H and 1I/shControl UT gH2AX.tif]

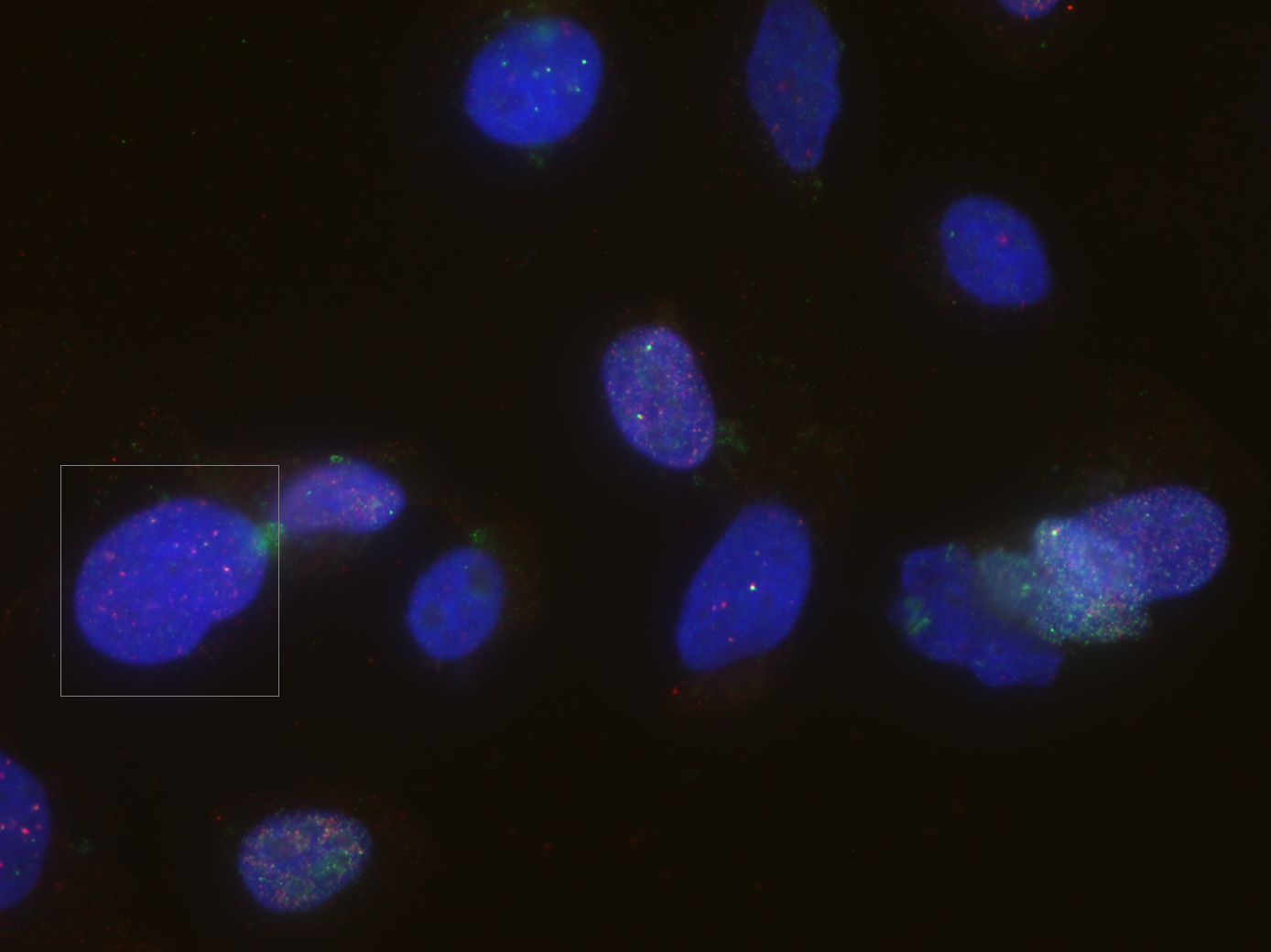

Supplement: Supplementary file 4 — Source data Fig. 1 [file 44319_2025_497_MOESM4_ESM.zip › 1H and 1I/shRNF20 HU gH2AX.tif]

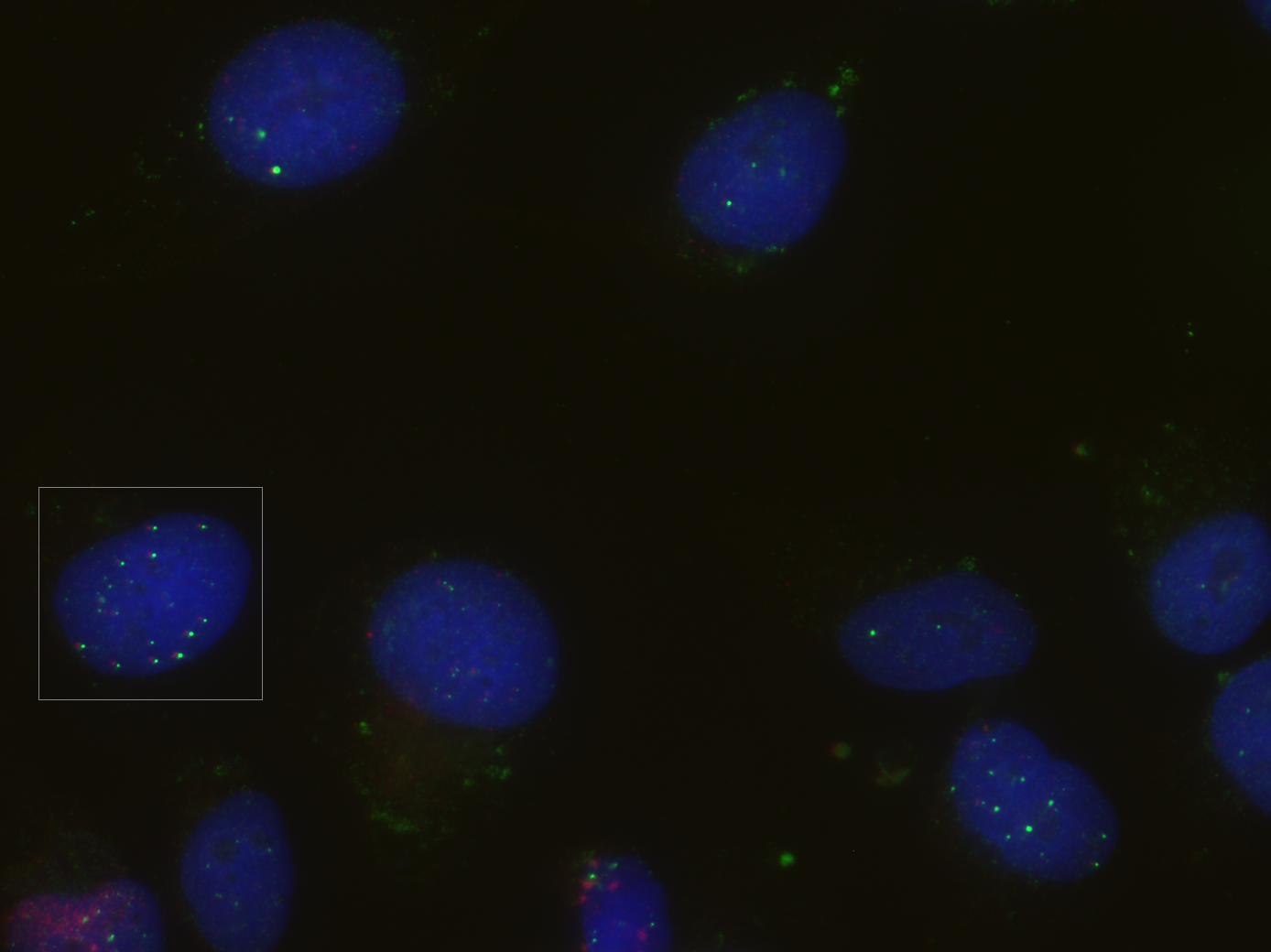

Supplement: Supplementary file 4 — Source data Fig. 1 [file 44319_2025_497_MOESM4_ESM.zip › 1H and 1I/shRNF20 UT gH2AX.tif]

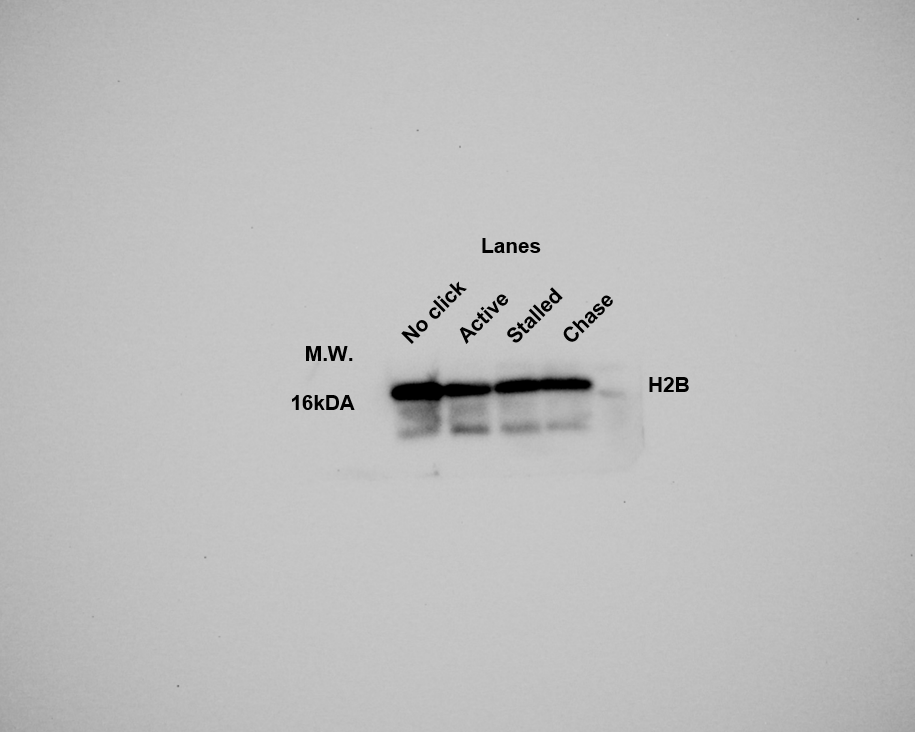

Supplement: Supplementary file 5 — Source data Fig. 2 [file 44319_2025_497_MOESM5_ESM.zip › 2B/H2B input western.tif]

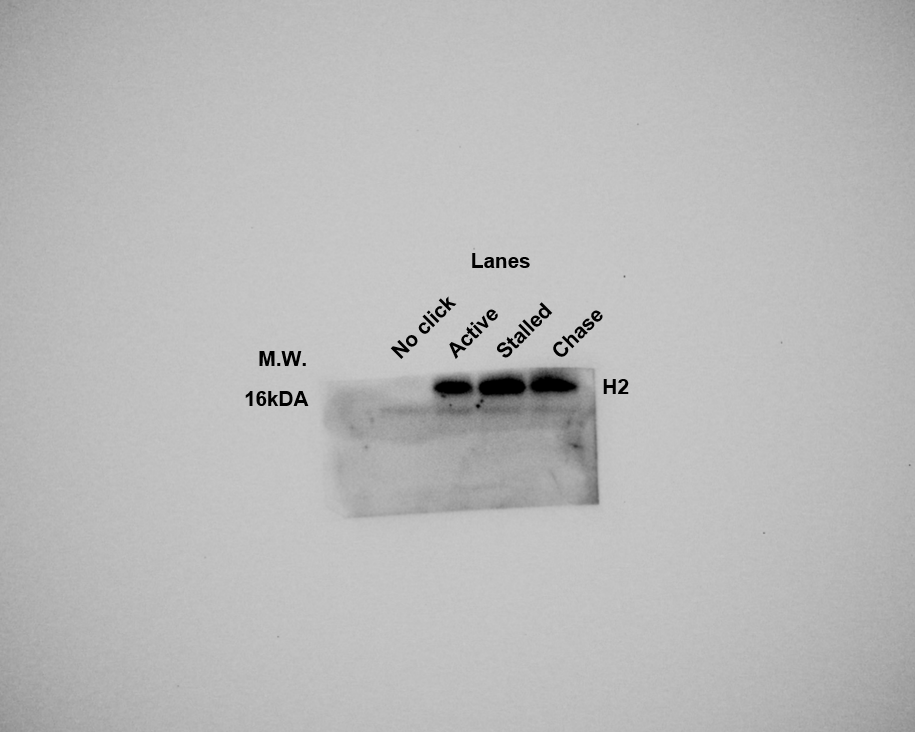

Supplement: Supplementary file 5 — Source data Fig. 2 [file 44319_2025_497_MOESM5_ESM.zip › 2B/H2B iPOND western.tif]

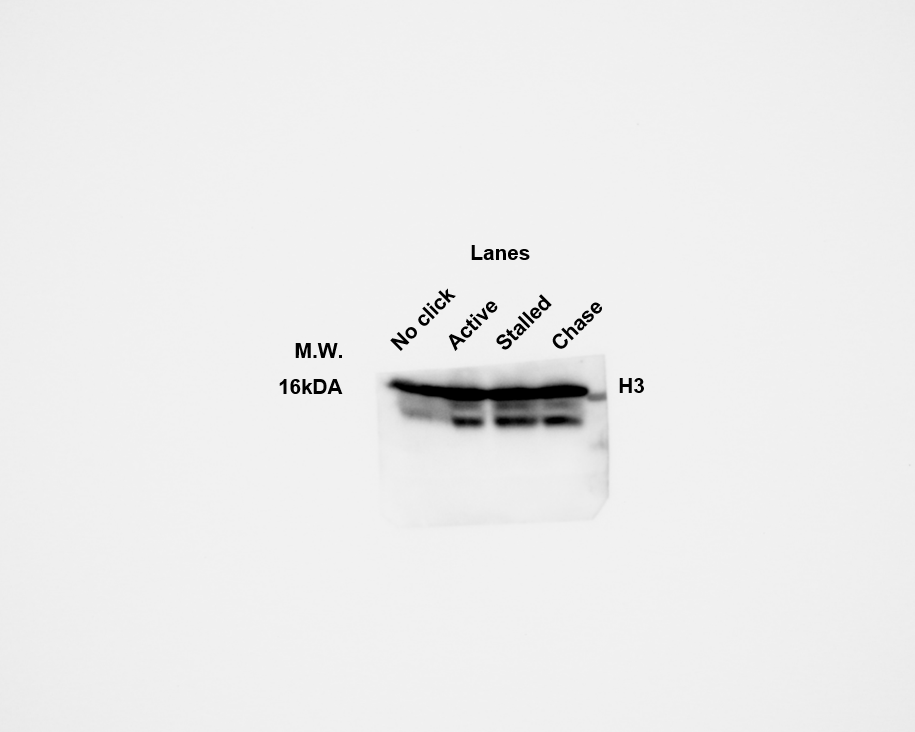

Supplement: Supplementary file 5 — Source data Fig. 2 [file 44319_2025_497_MOESM5_ESM.zip › 2B/H3 input western.tif]

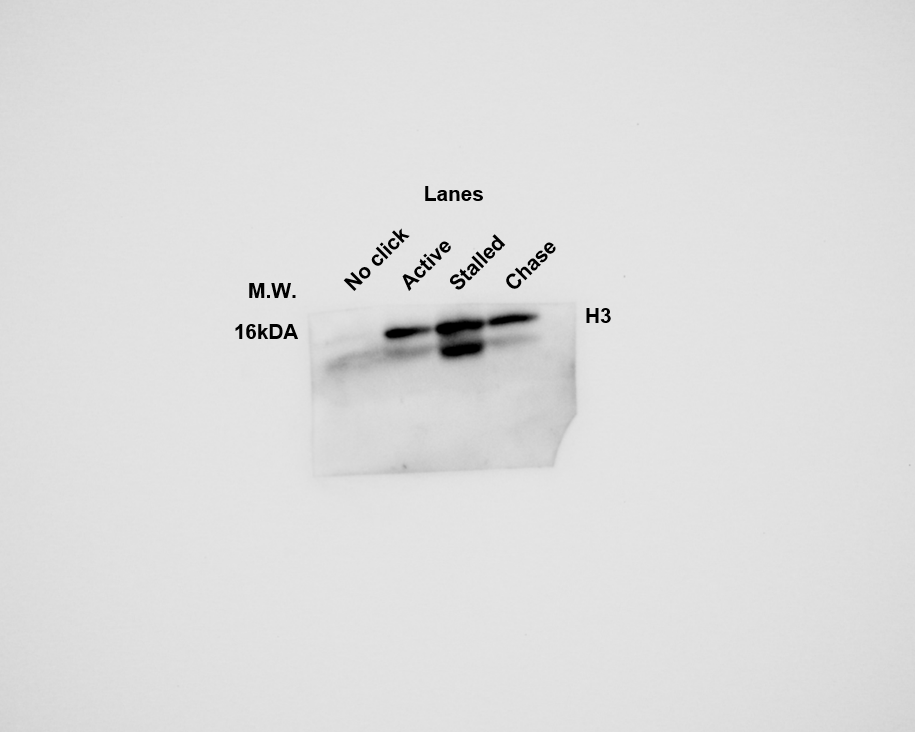

Supplement: Supplementary file 5 — Source data Fig. 2 [file 44319_2025_497_MOESM5_ESM.zip › 2B/H3 iPOND western.tif]

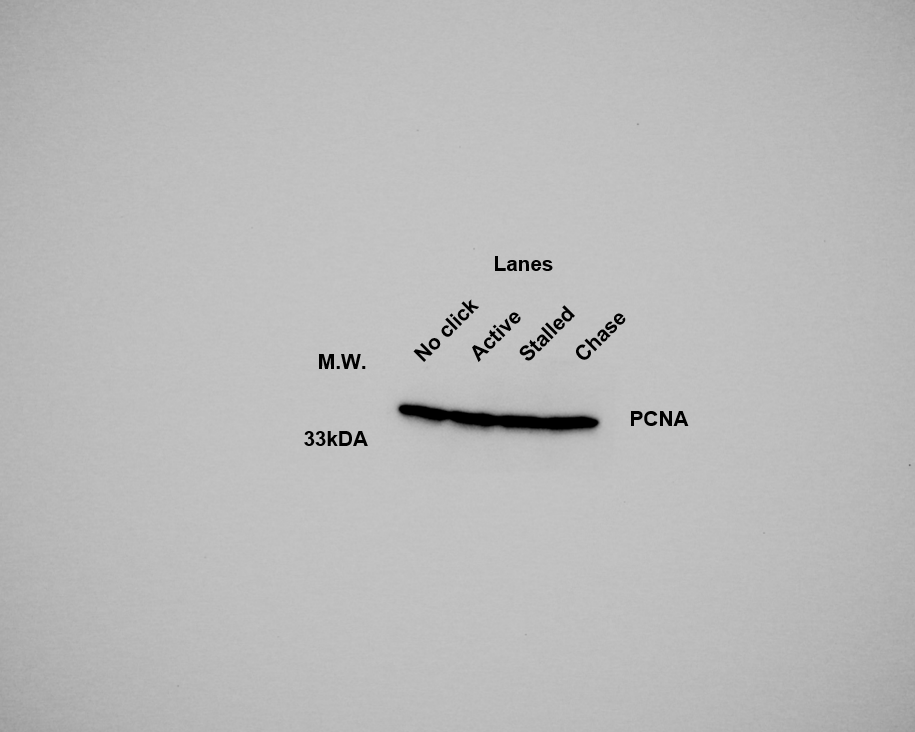

Supplement: Supplementary file 5 — Source data Fig. 2 [file 44319_2025_497_MOESM5_ESM.zip › 2B/PCNA input western.tif]

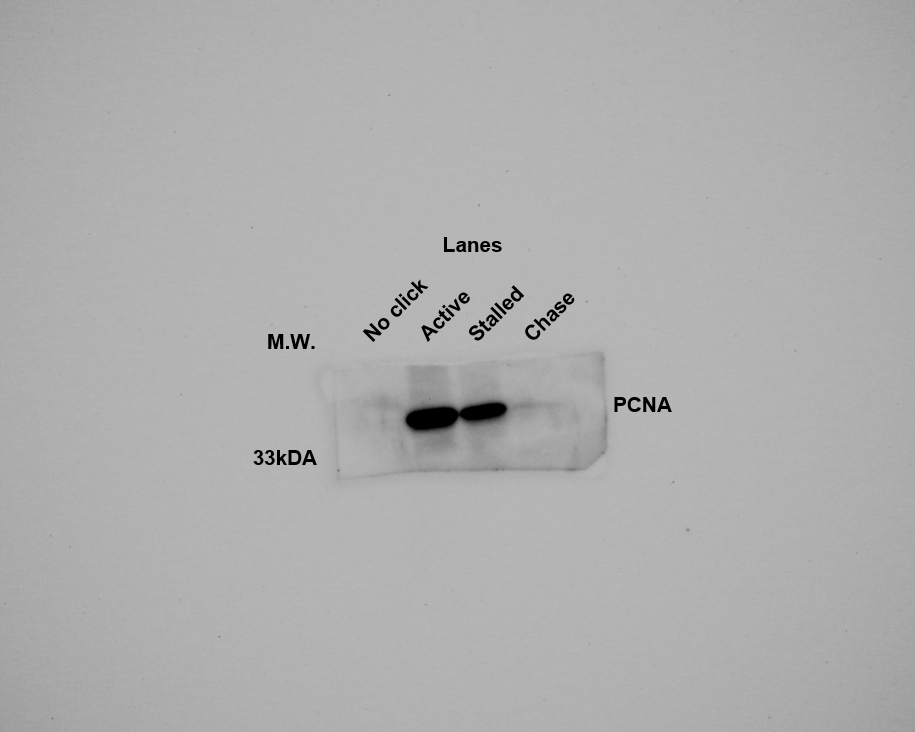

Supplement: Supplementary file 5 — Source data Fig. 2 [file 44319_2025_497_MOESM5_ESM.zip › 2B/PCNA iPOND western.tif]

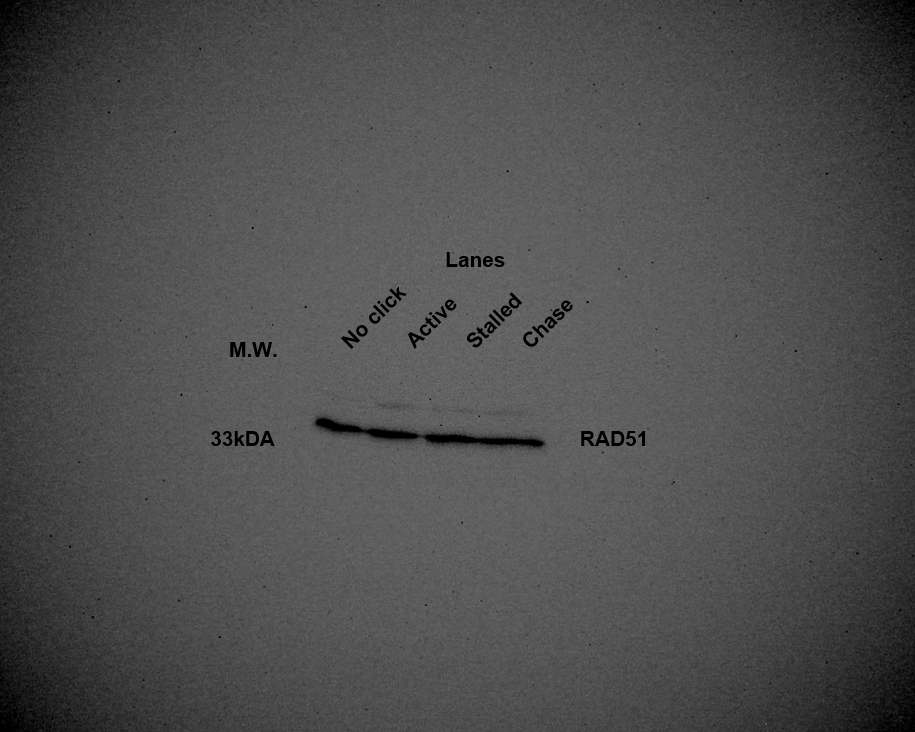

Supplement: Supplementary file 5 — Source data Fig. 2 [file 44319_2025_497_MOESM5_ESM.zip › 2B/RAD51 input western.tif]

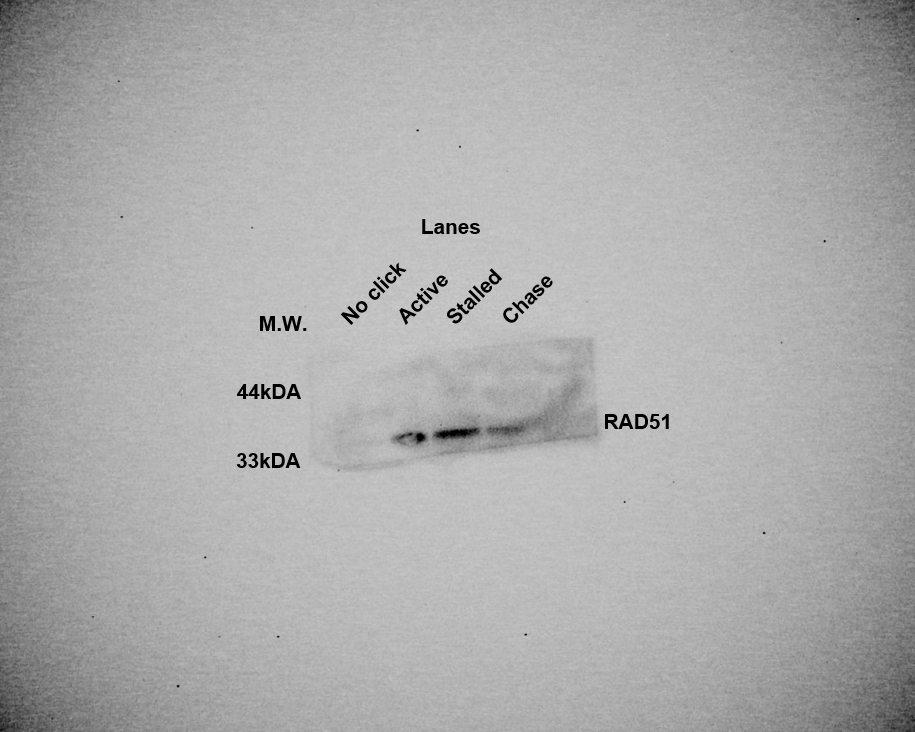

Supplement: Supplementary file 5 — Source data Fig. 2 [file 44319_2025_497_MOESM5_ESM.zip › 2B/RAD51 iPOND western.tif]

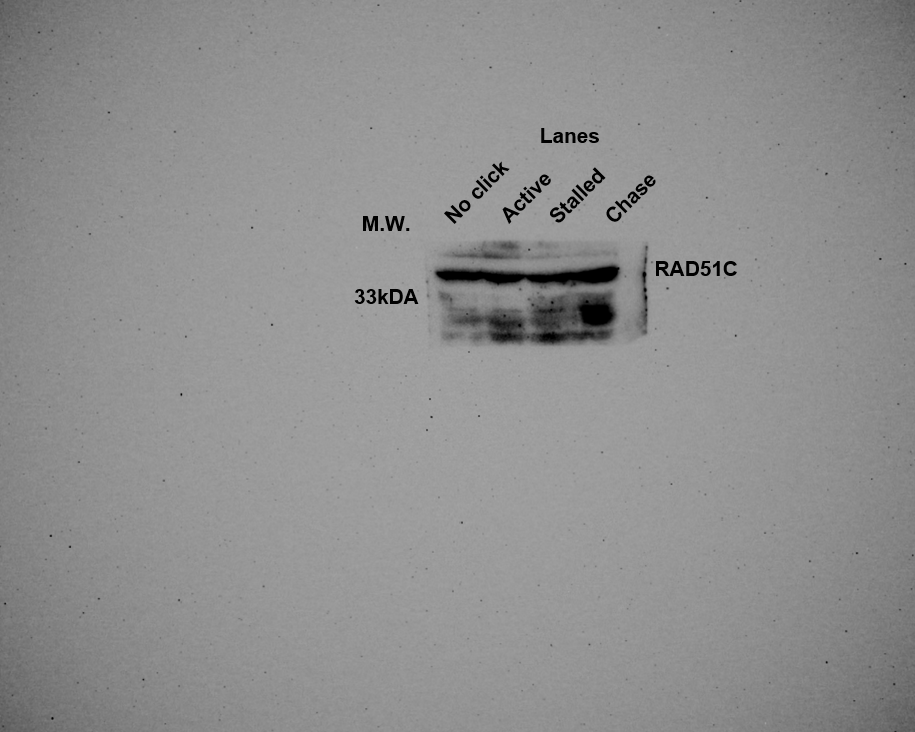

Supplement: Supplementary file 5 — Source data Fig. 2 [file 44319_2025_497_MOESM5_ESM.zip › 2B/RAD51C input western.tif]

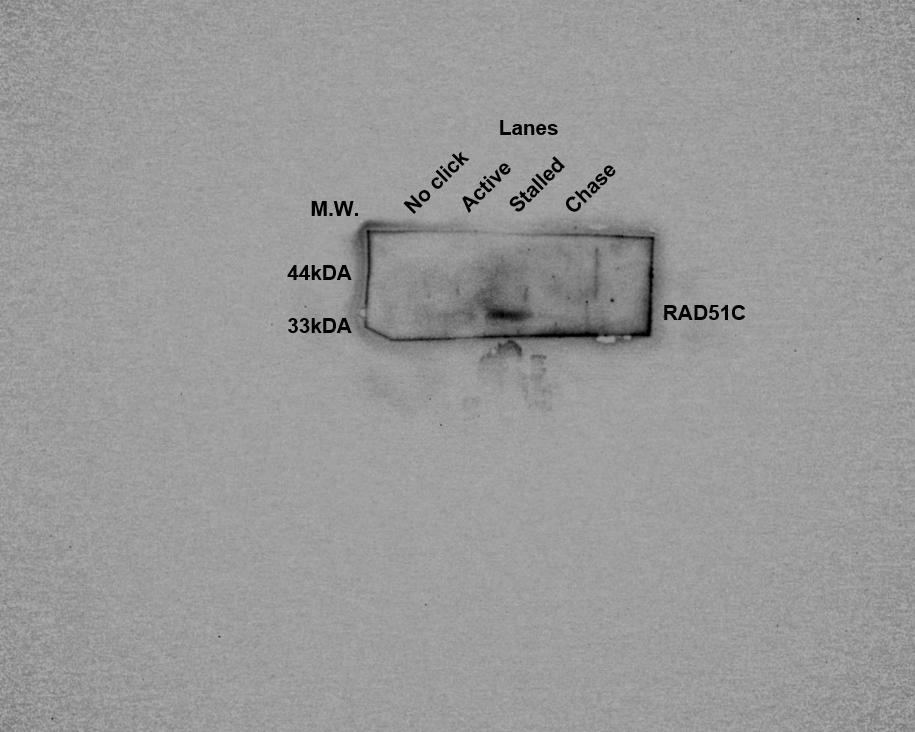

Supplement: Supplementary file 5 — Source data Fig. 2 [file 44319_2025_497_MOESM5_ESM.zip › 2B/RAD51C iPOND western.tif]

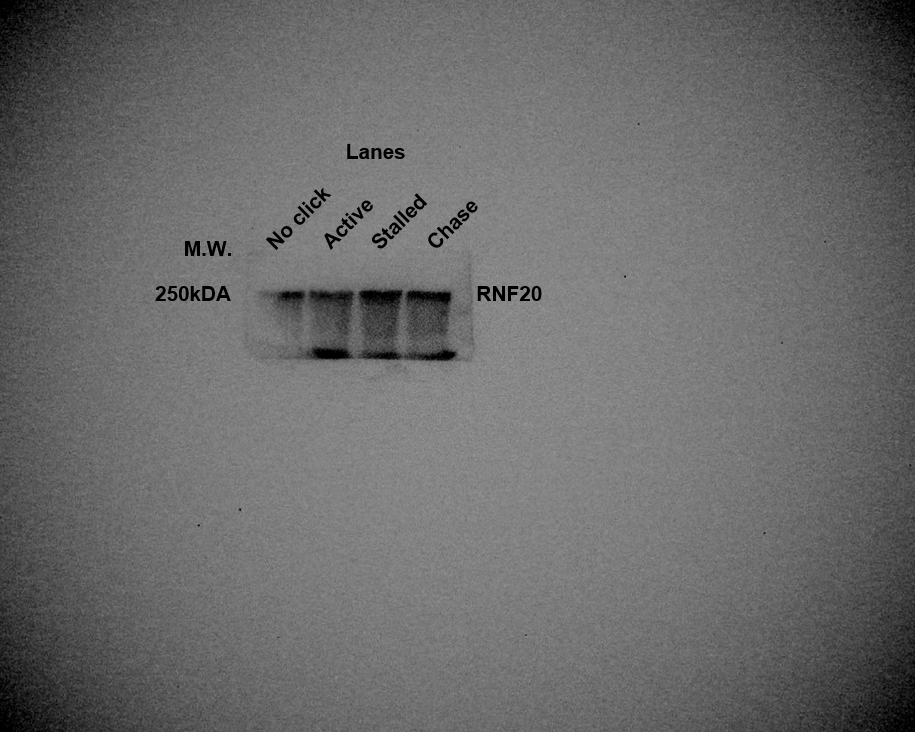

Supplement: Supplementary file 5 — Source data Fig. 2 [file 44319_2025_497_MOESM5_ESM.zip › 2B/RNF20 input western.tif]

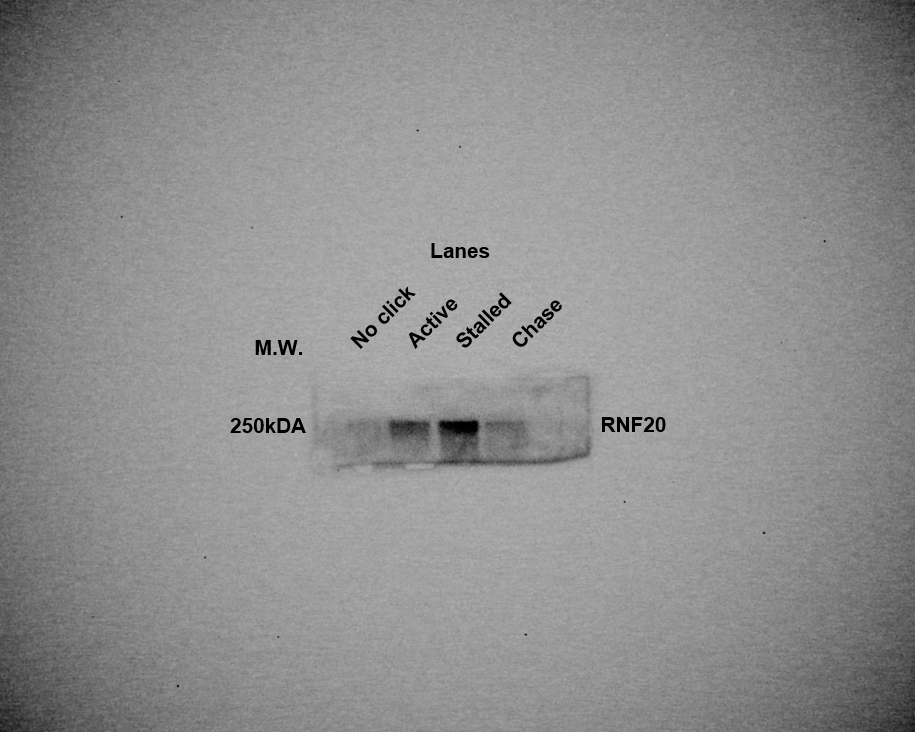

Supplement: Supplementary file 5 — Source data Fig. 2 [file 44319_2025_497_MOESM5_ESM.zip › 2B/RNF20 iPOND western.tif]

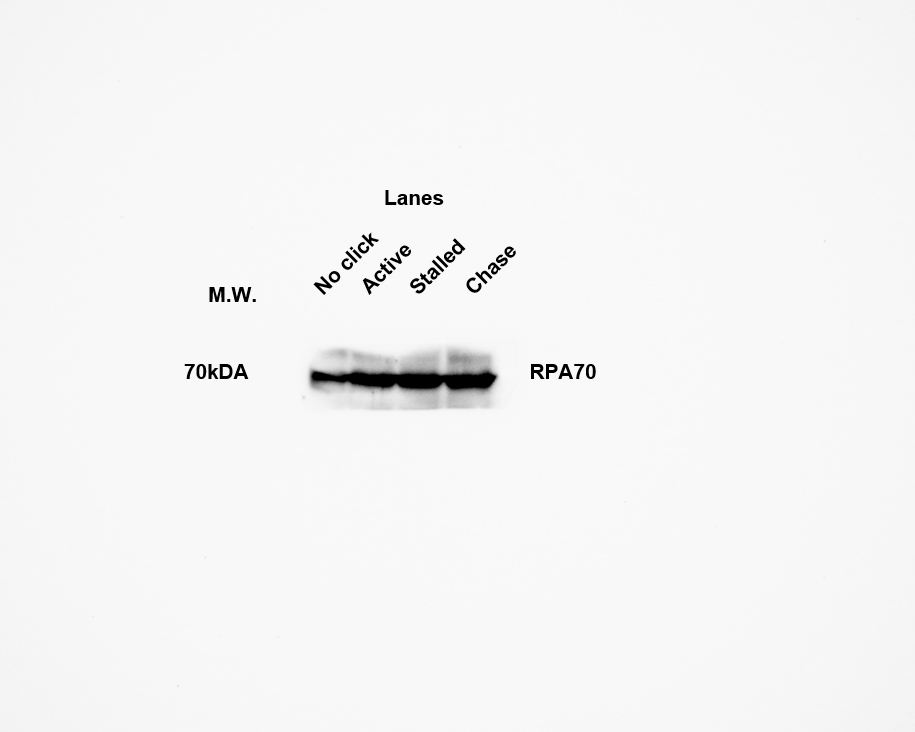

Supplement: Supplementary file 5 — Source data Fig. 2 [file 44319_2025_497_MOESM5_ESM.zip › 2B/RPA70 input western.tif]

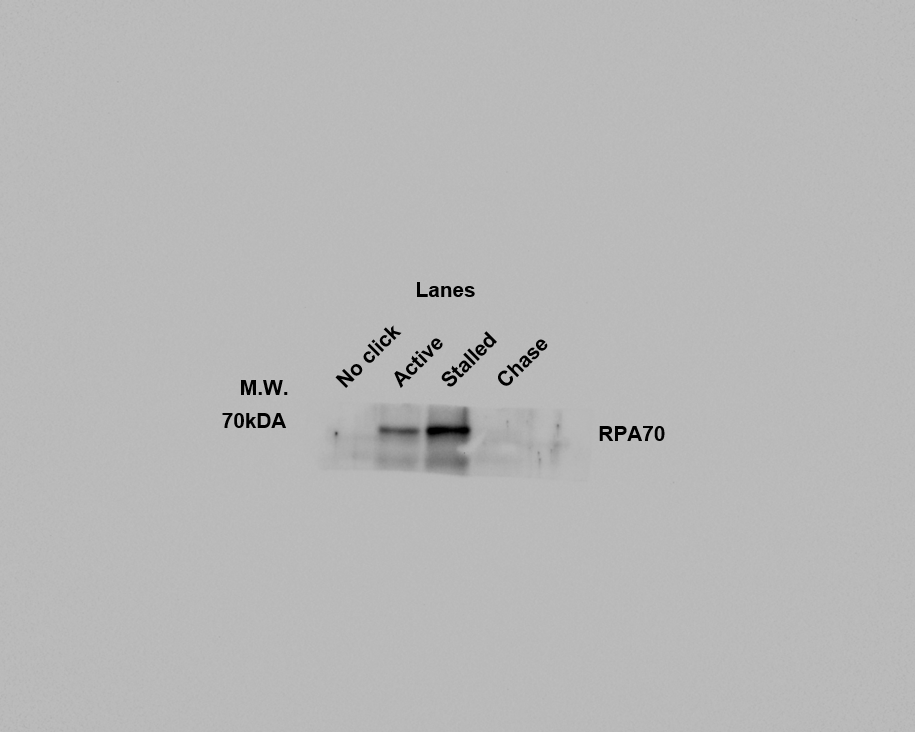

Supplement: Supplementary file 5 — Source data Fig. 2 [file 44319_2025_497_MOESM5_ESM.zip › 2B/RPA70 iPOND western.tif]

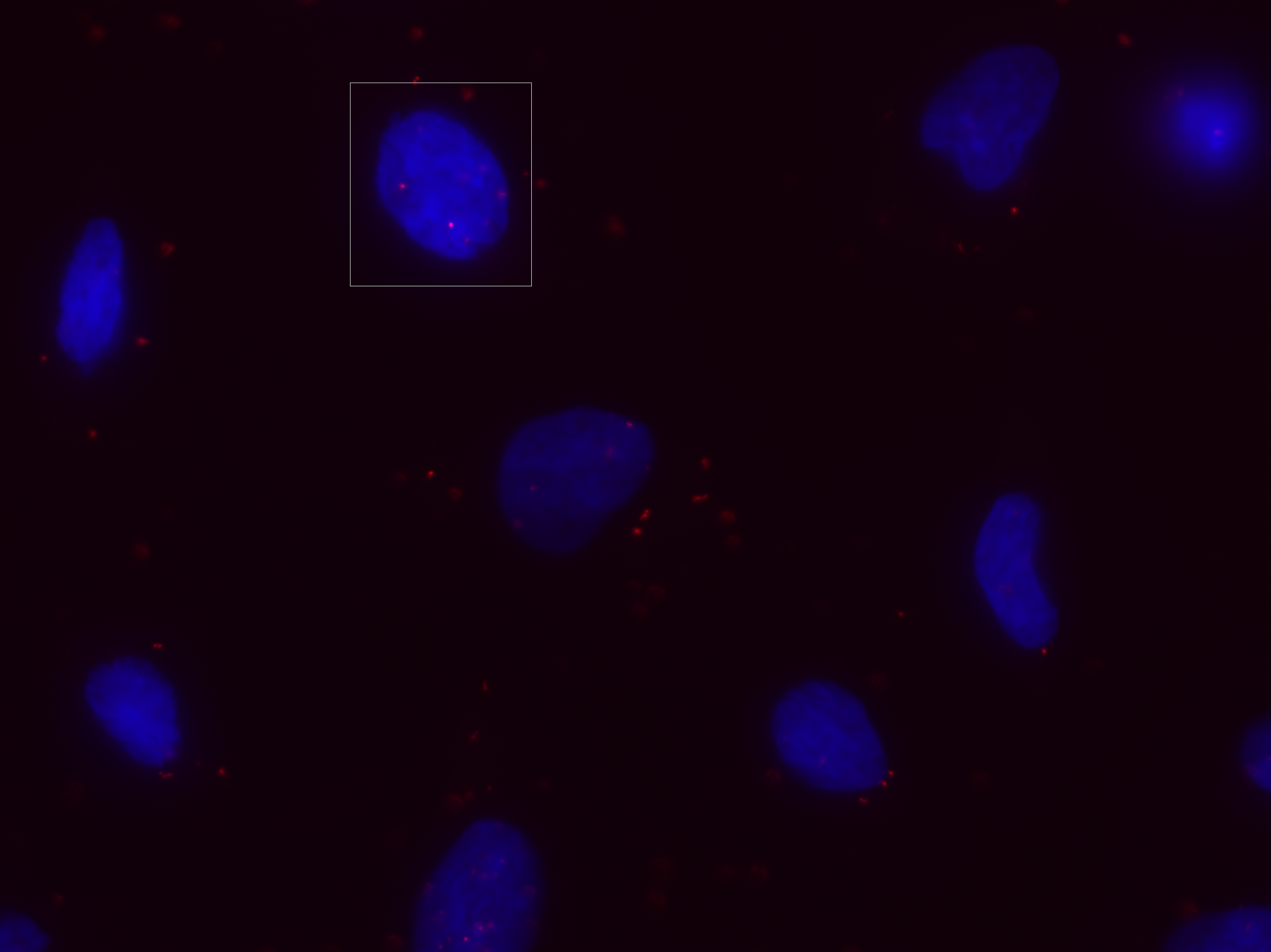

Supplement: Supplementary file 5 — Source data Fig. 2 [file 44319_2025_497_MOESM5_ESM.zip › 2C and 2D/Chase RNF20 SIRF.tif]

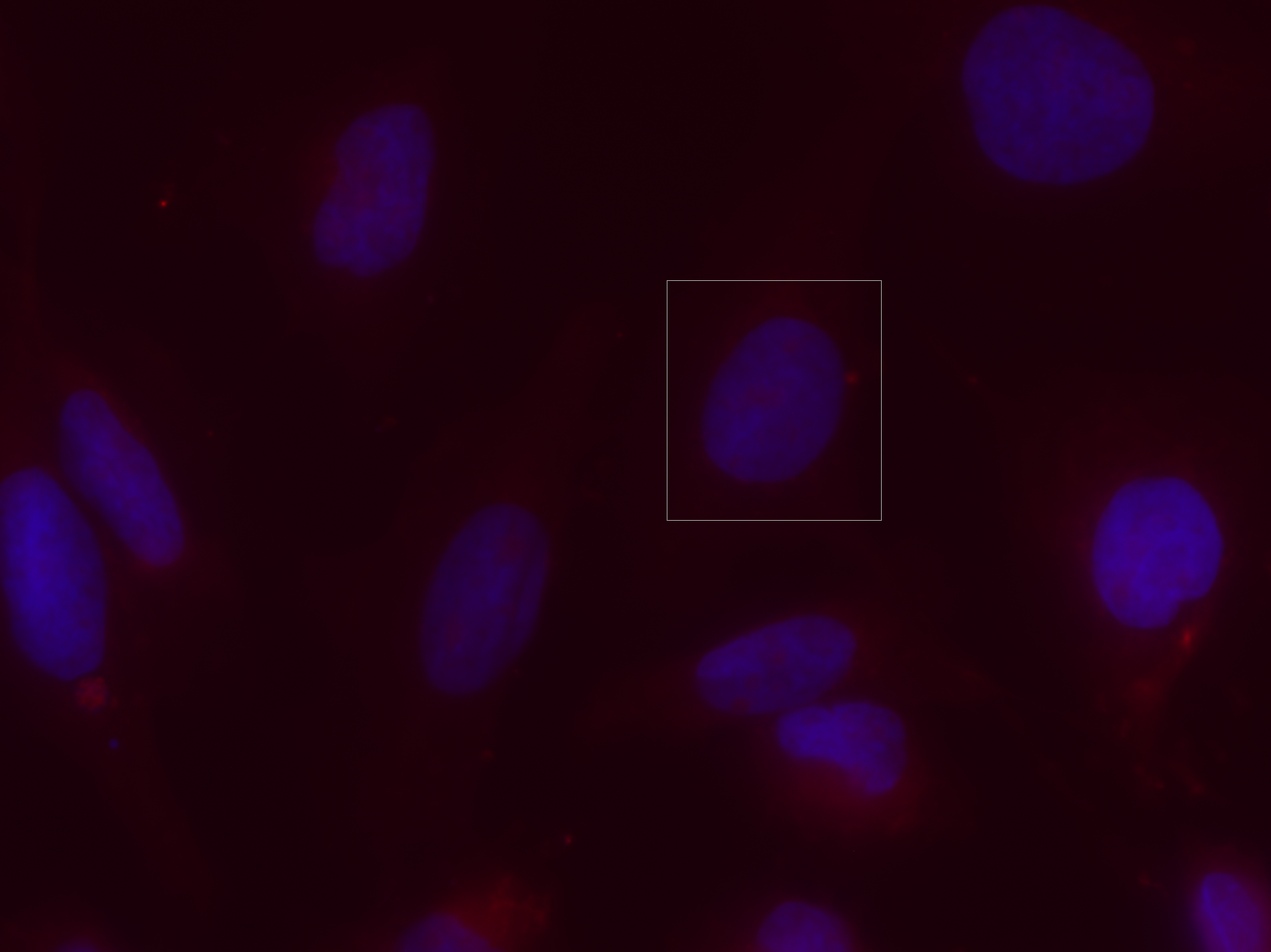

Supplement: Supplementary file 5 — Source data Fig. 2 [file 44319_2025_497_MOESM5_ESM.zip › 2C and 2D/No ab RNF20 SIRF.tif]

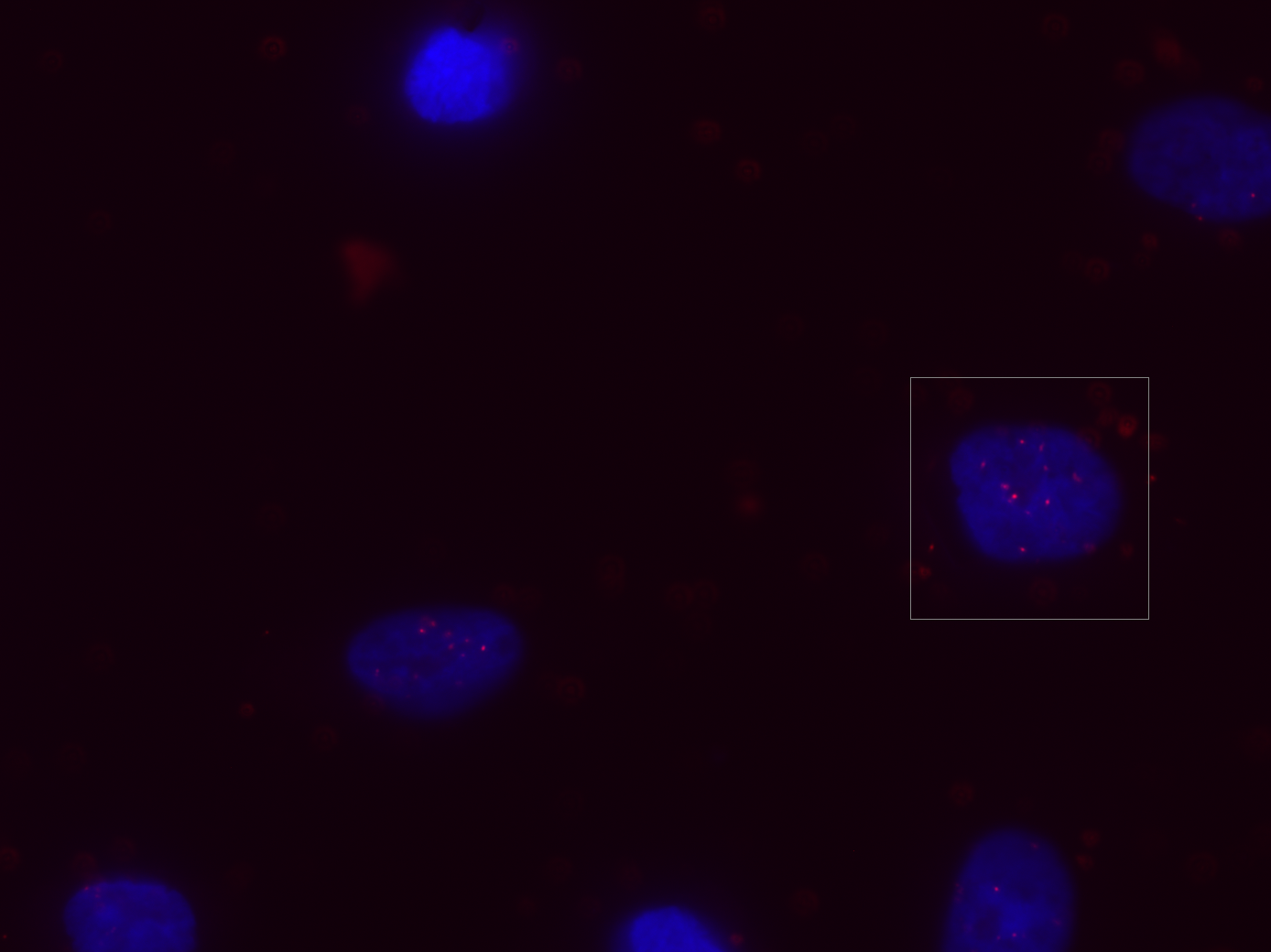

Supplement: Supplementary file 5 — Source data Fig. 2 [file 44319_2025_497_MOESM5_ESM.zip › 2C and 2D/Progressing RNF20 SIRF.tif]

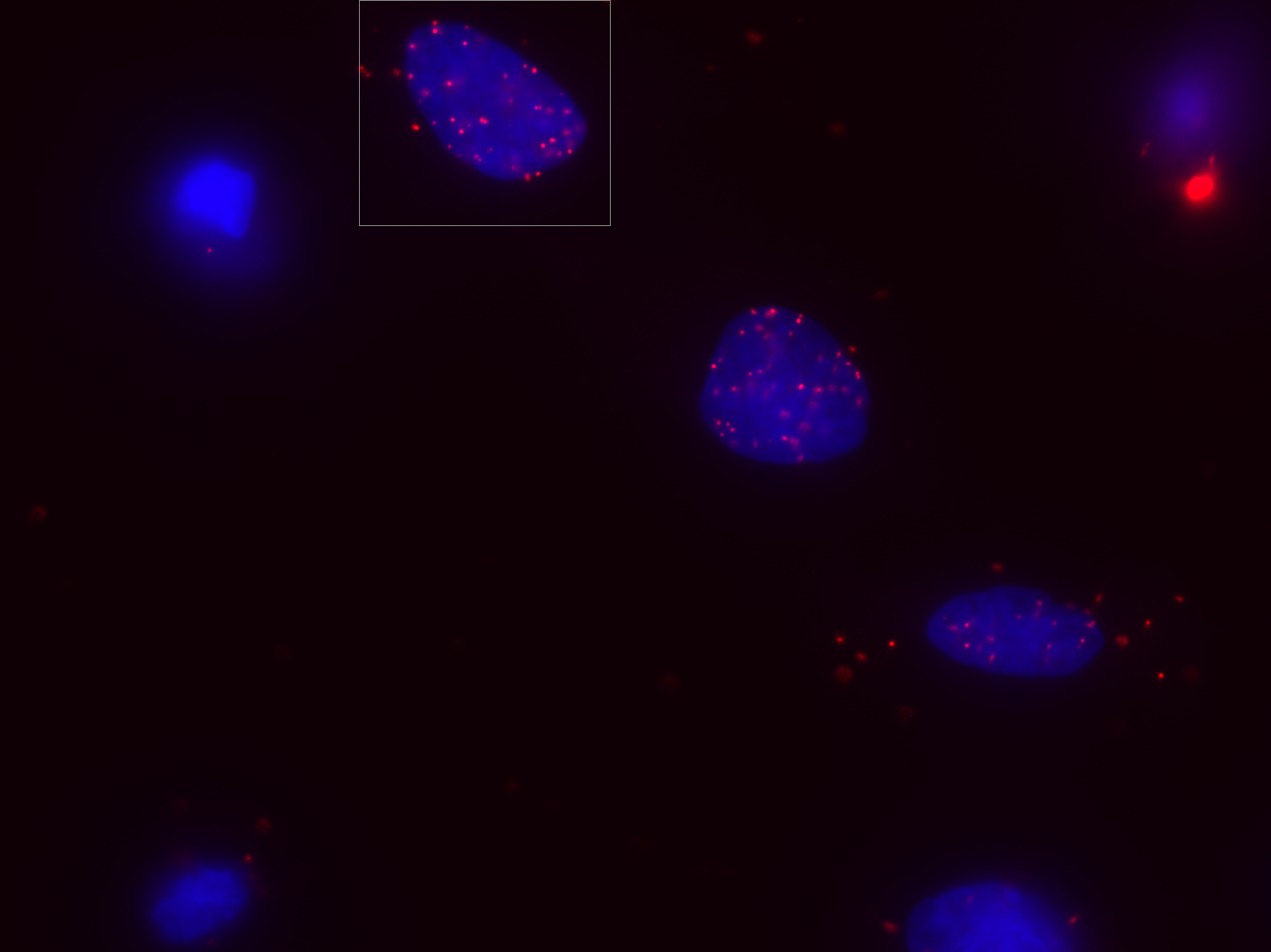

Supplement: Supplementary file 5 — Source data Fig. 2 [file 44319_2025_497_MOESM5_ESM.zip › 2C and 2D/Stalled RNF20 SIRF.tif]

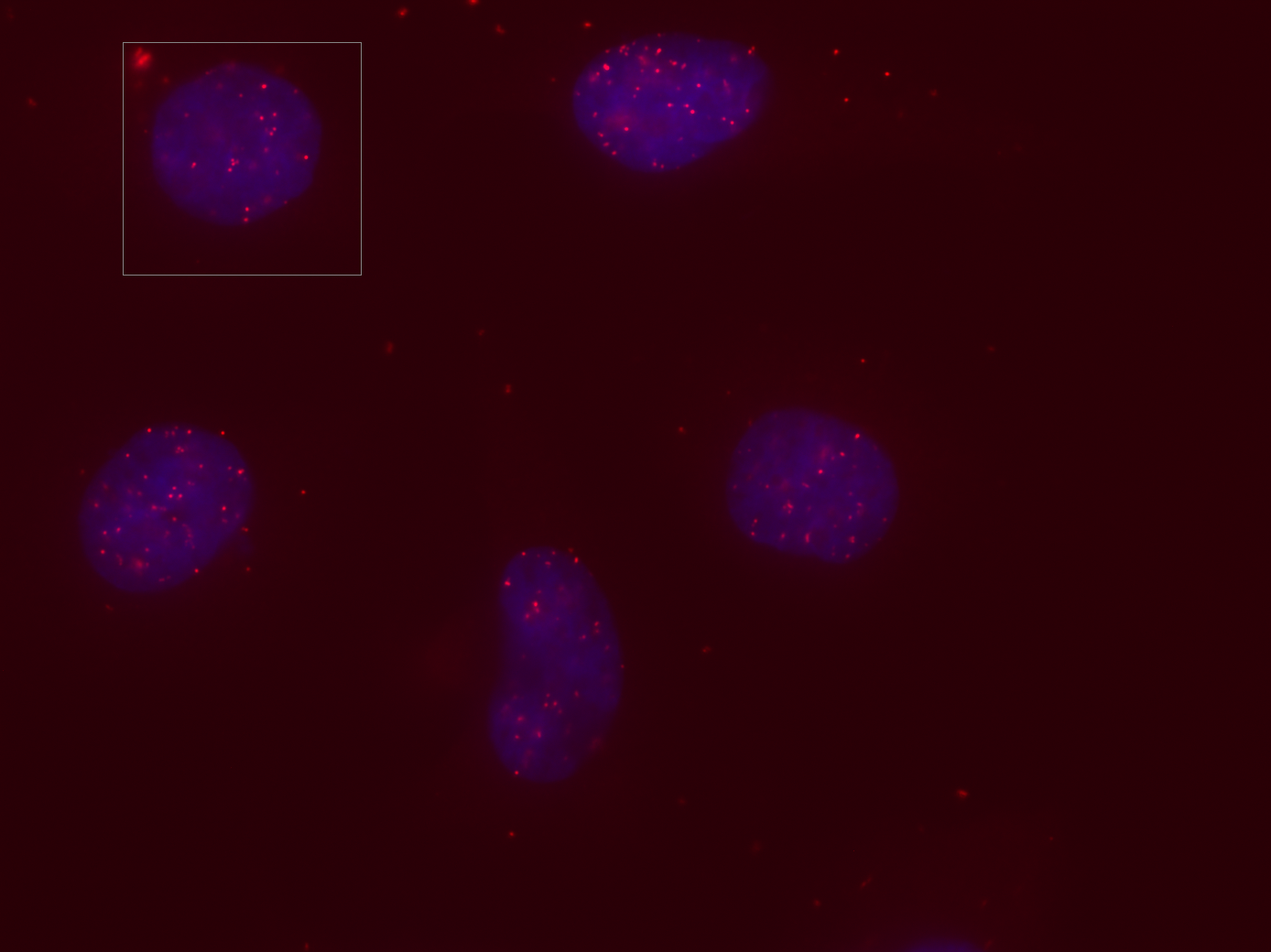

Supplement: Supplementary file 5 — Source data Fig. 2 [file 44319_2025_497_MOESM5_ESM.zip › 2E and 2F/shControl progressing H2BK120ub SIRF.tif]

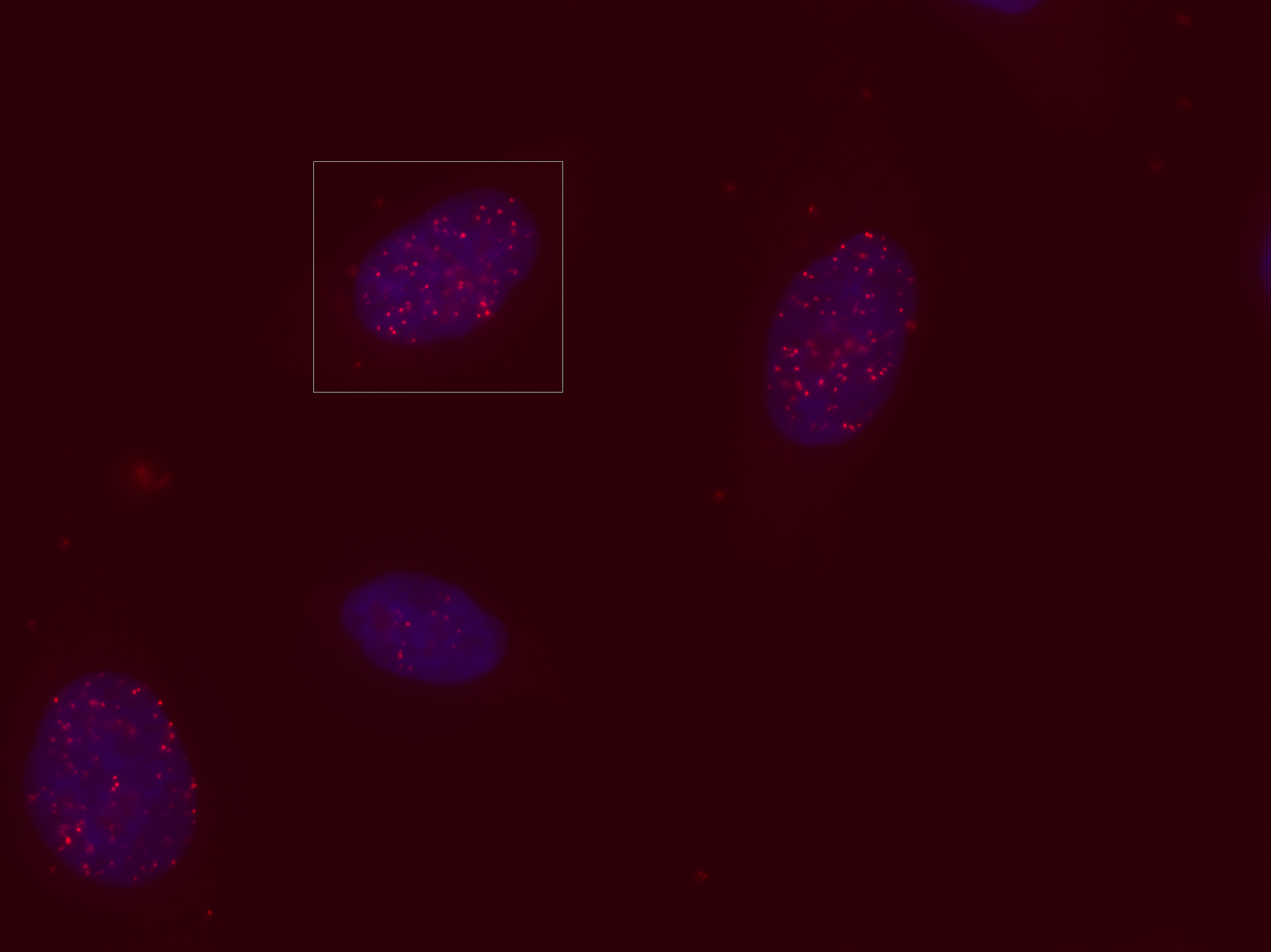

Supplement: Supplementary file 5 — Source data Fig. 2 [file 44319_2025_497_MOESM5_ESM.zip › 2E and 2F/shControl stalled H2BK120ub SIRF.tif]

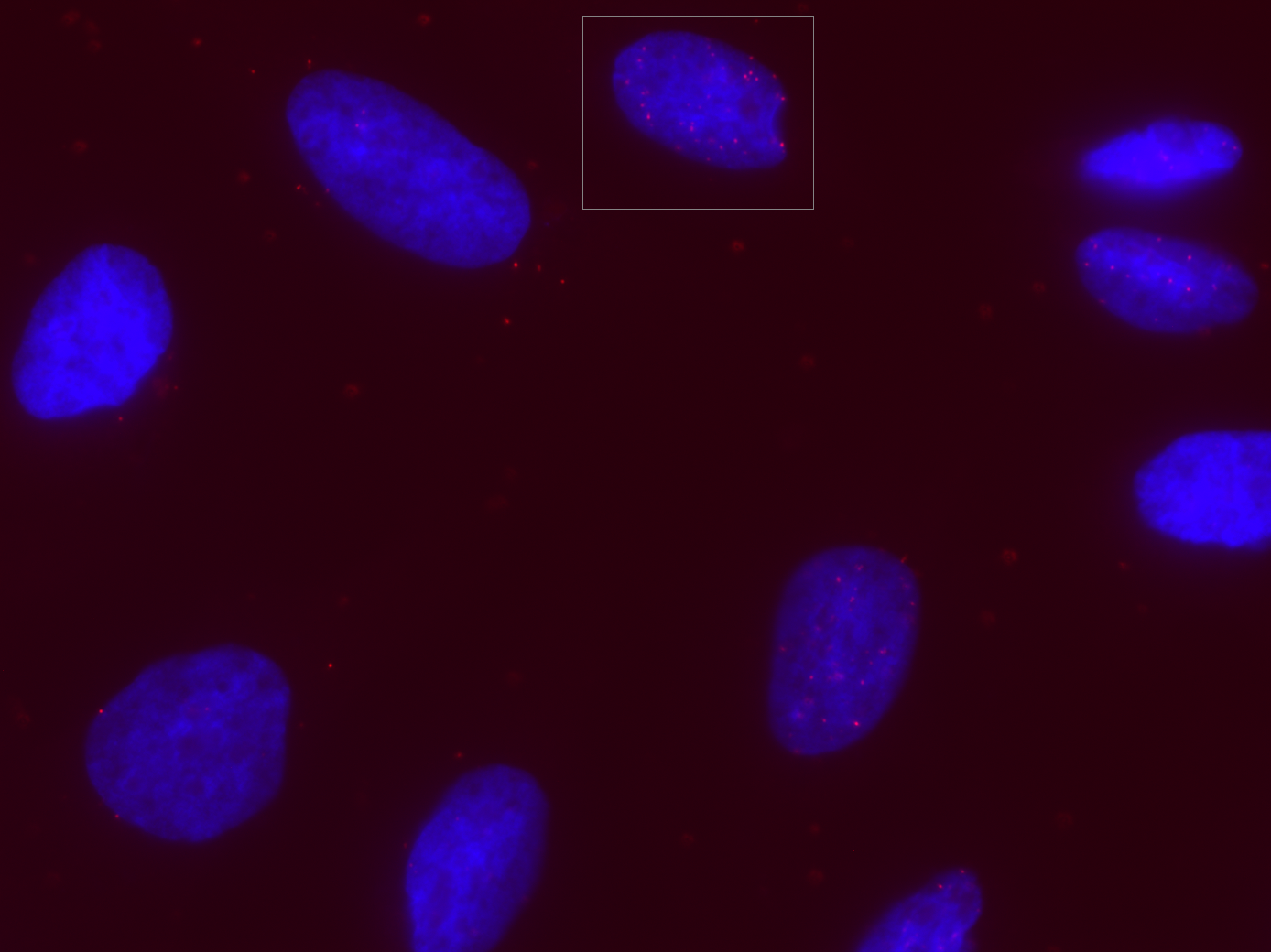

Supplement: Supplementary file 5 — Source data Fig. 2 [file 44319_2025_497_MOESM5_ESM.zip › 2E and 2F/shRNF20 progressing H2BK120ub SIRF.tif]

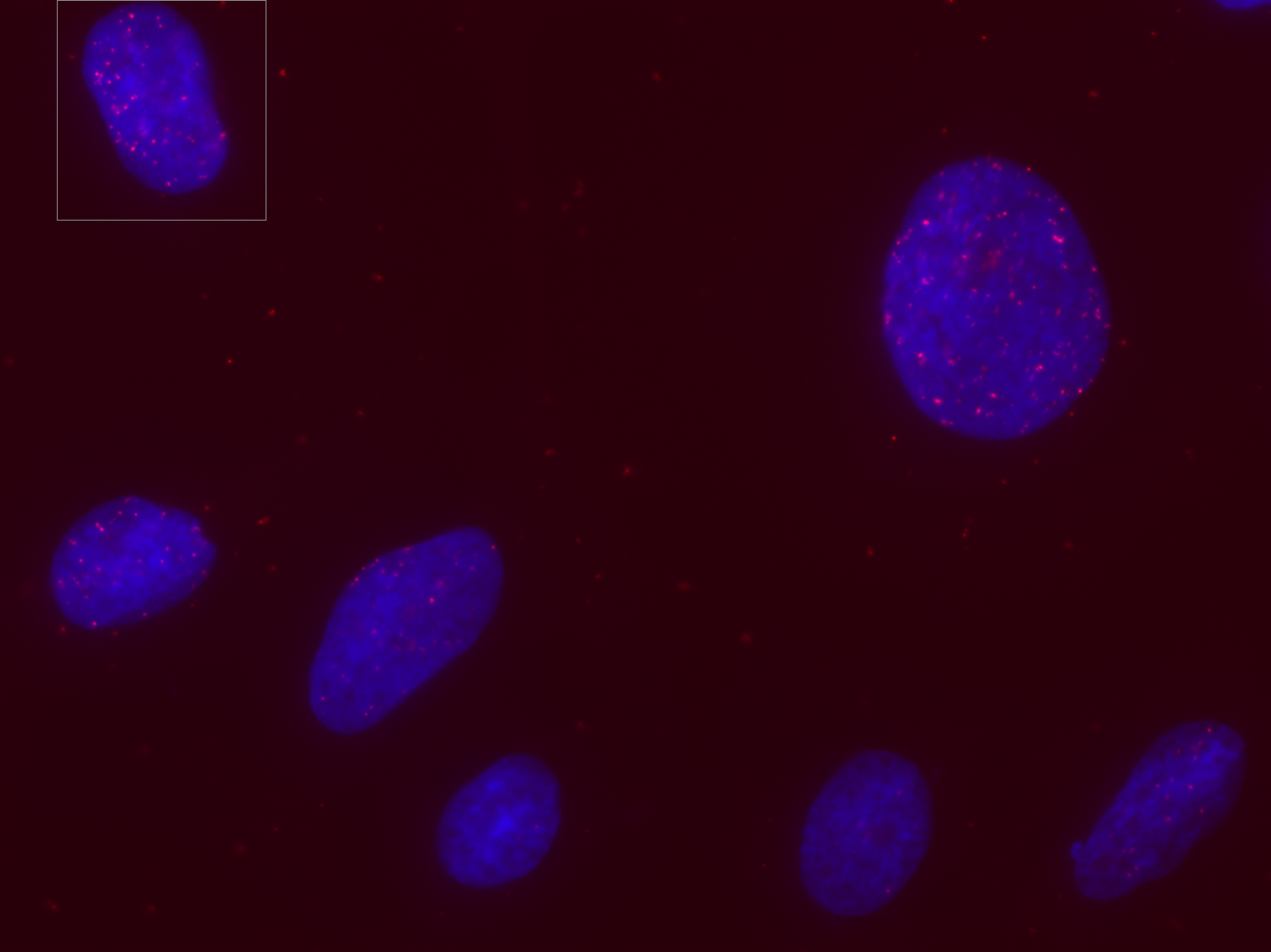

Supplement: Supplementary file 5 — Source data Fig. 2 [file 44319_2025_497_MOESM5_ESM.zip › 2E and 2F/shRNF20 stalled H2BK120ub SIRF.tif]

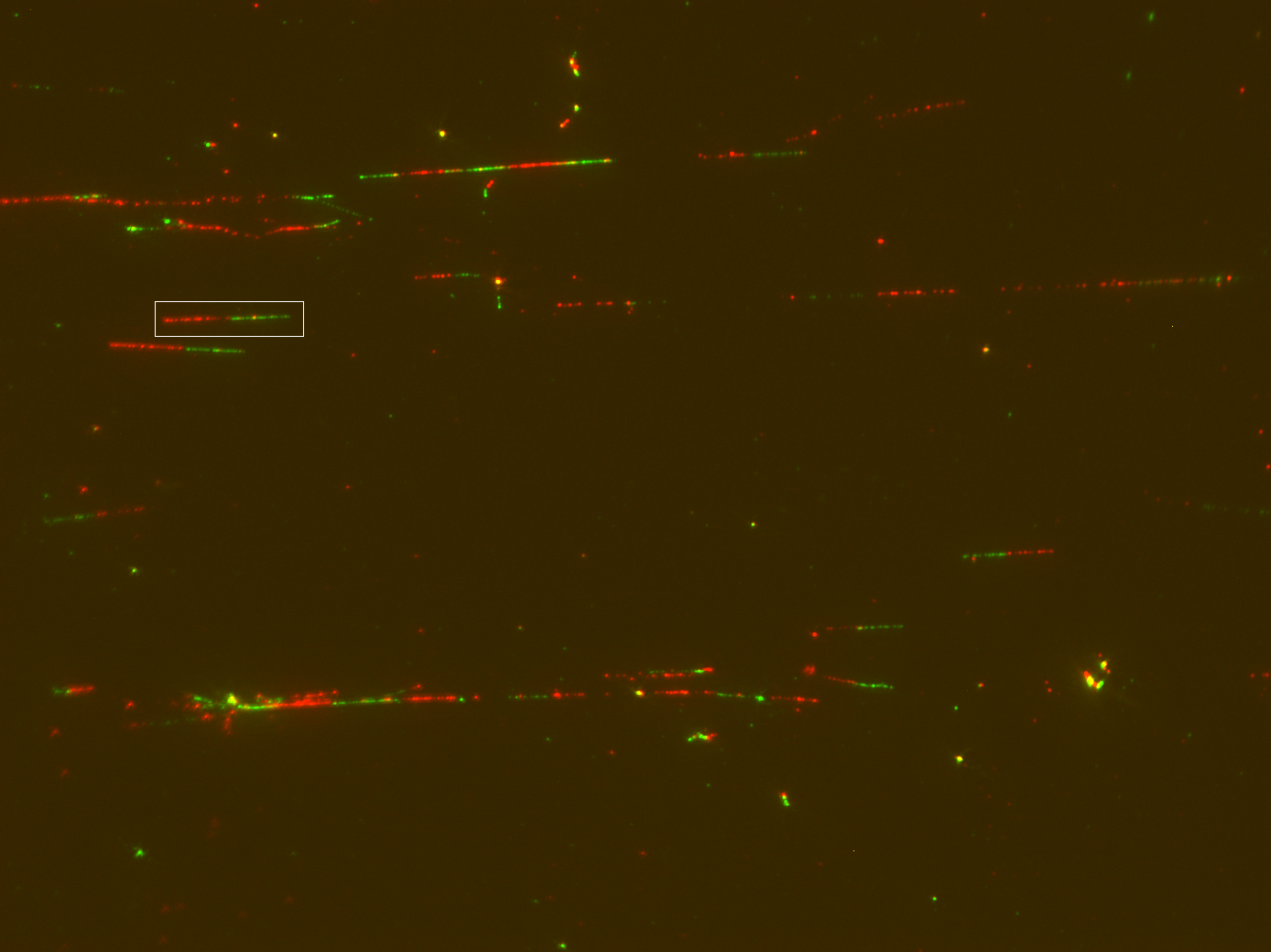

Supplement: Supplementary file 6 — Source data Fig. 3 [file 44319_2025_497_MOESM6_ESM.zip › 3A and 3B/shControl DNA fiber degradation.tif]

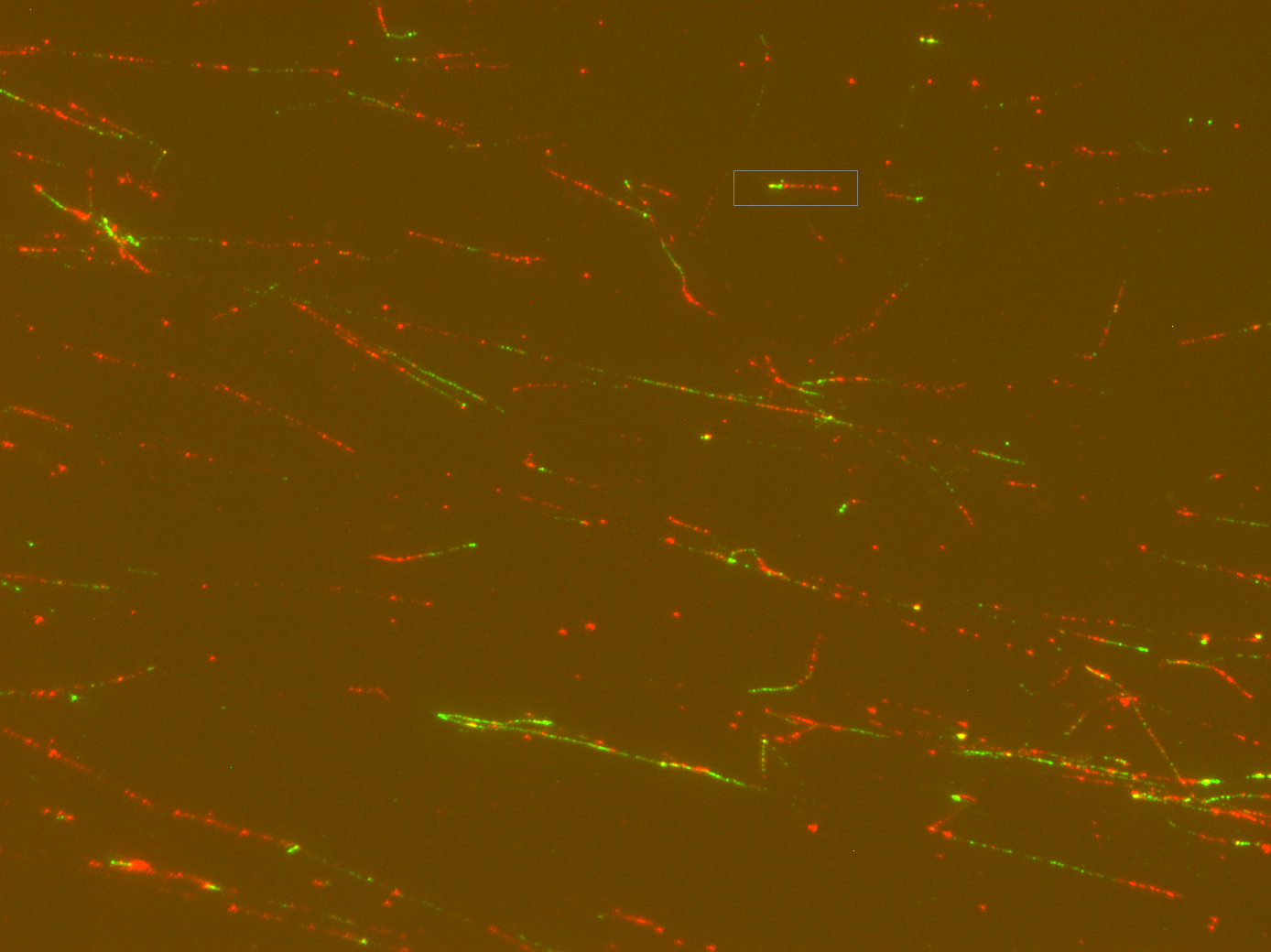

Supplement: Supplementary file 6 — Source data Fig. 3 [file 44319_2025_497_MOESM6_ESM.zip › 3A and 3B/shRNF20 #1 DNA fiber degradation.tif]

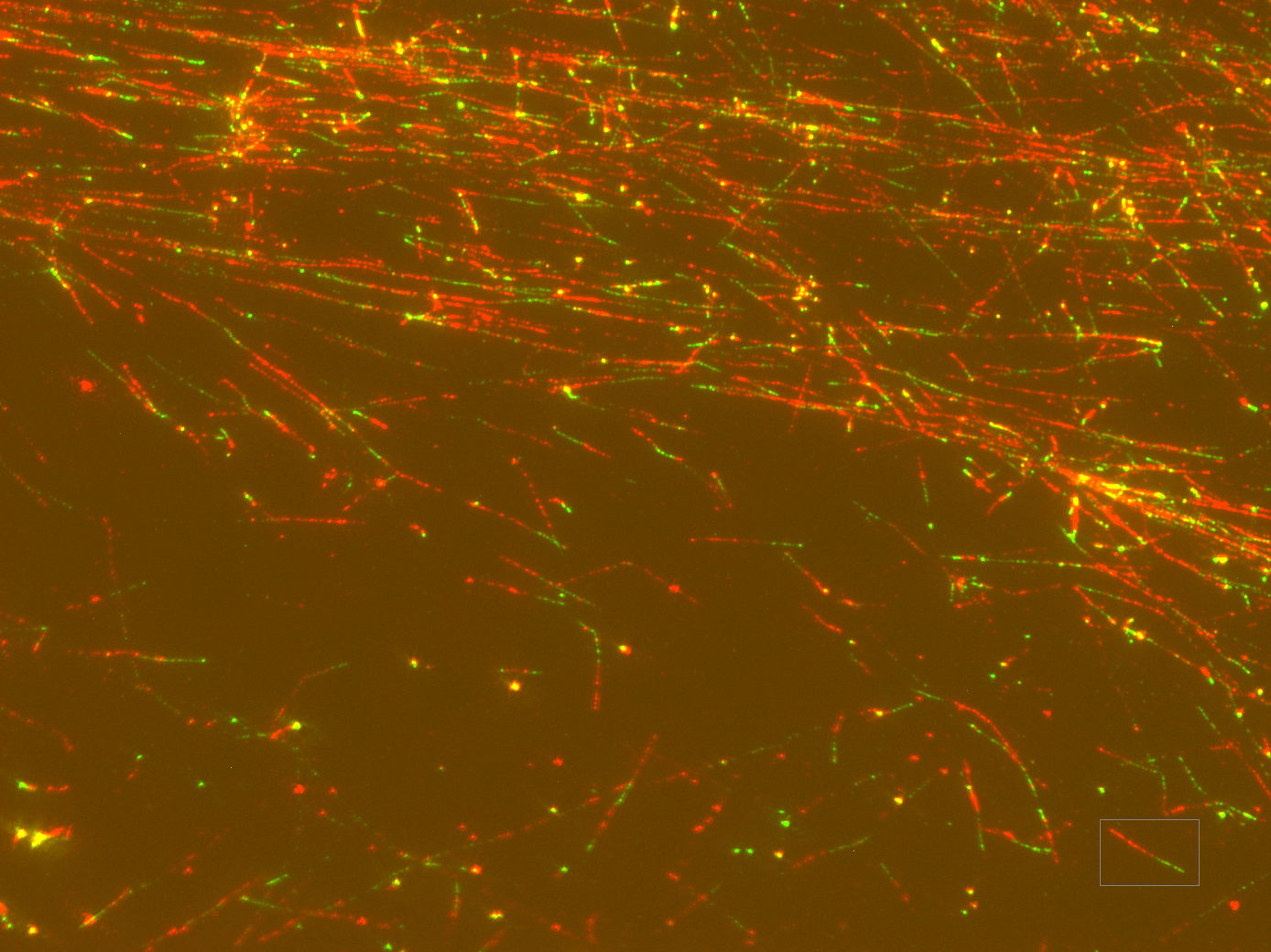

Supplement: Supplementary file 6 — Source data Fig. 3 [file 44319_2025_497_MOESM6_ESM.zip › 3A and 3B/shRNF20 #2 DNA fiber degradation.tif]

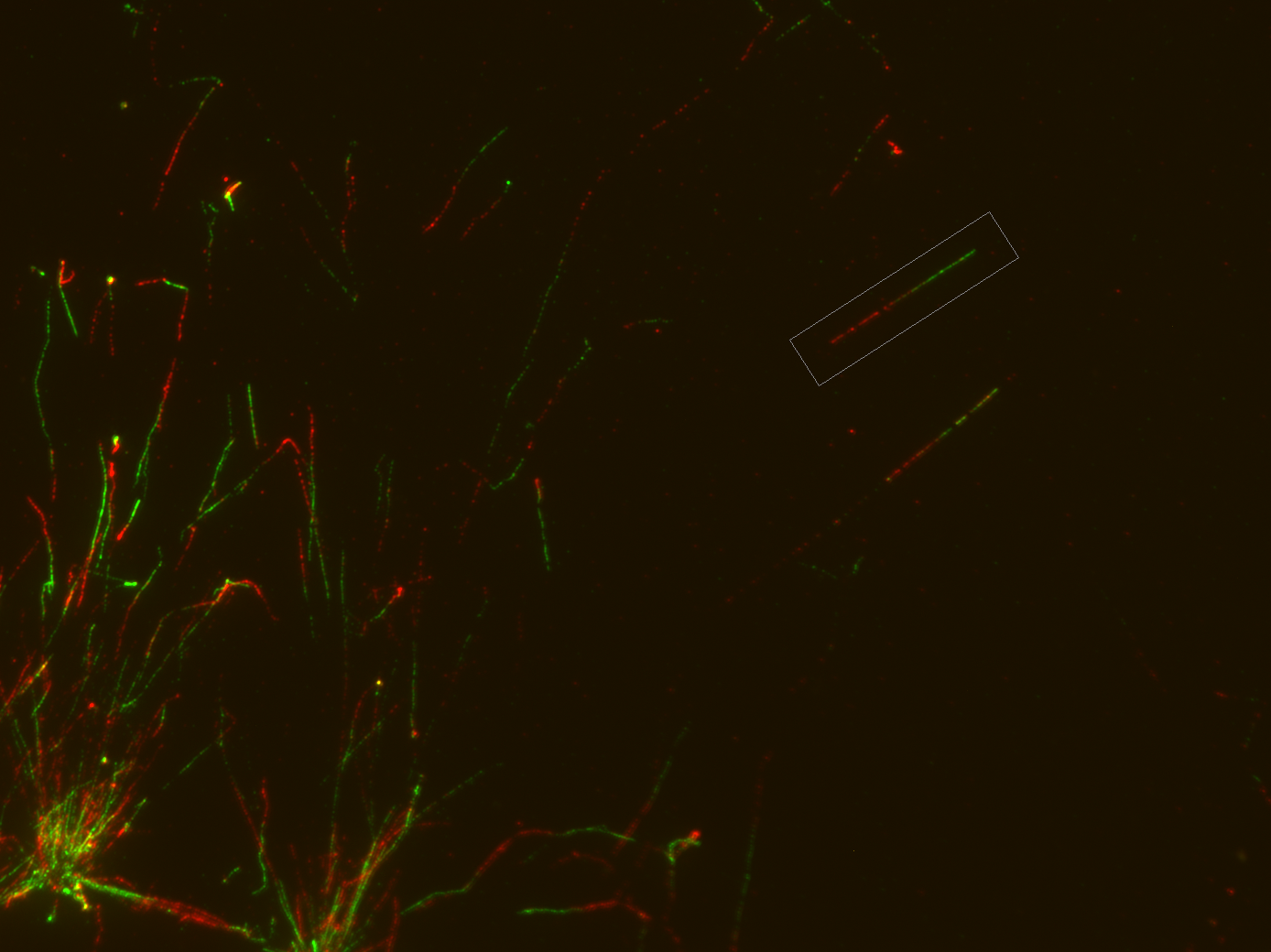

Supplement: Supplementary file 6 — Source data Fig. 3 [file 44319_2025_497_MOESM6_ESM.zip › 3C and 3D/shControl fork protection with mirin DNA fiber.tif]

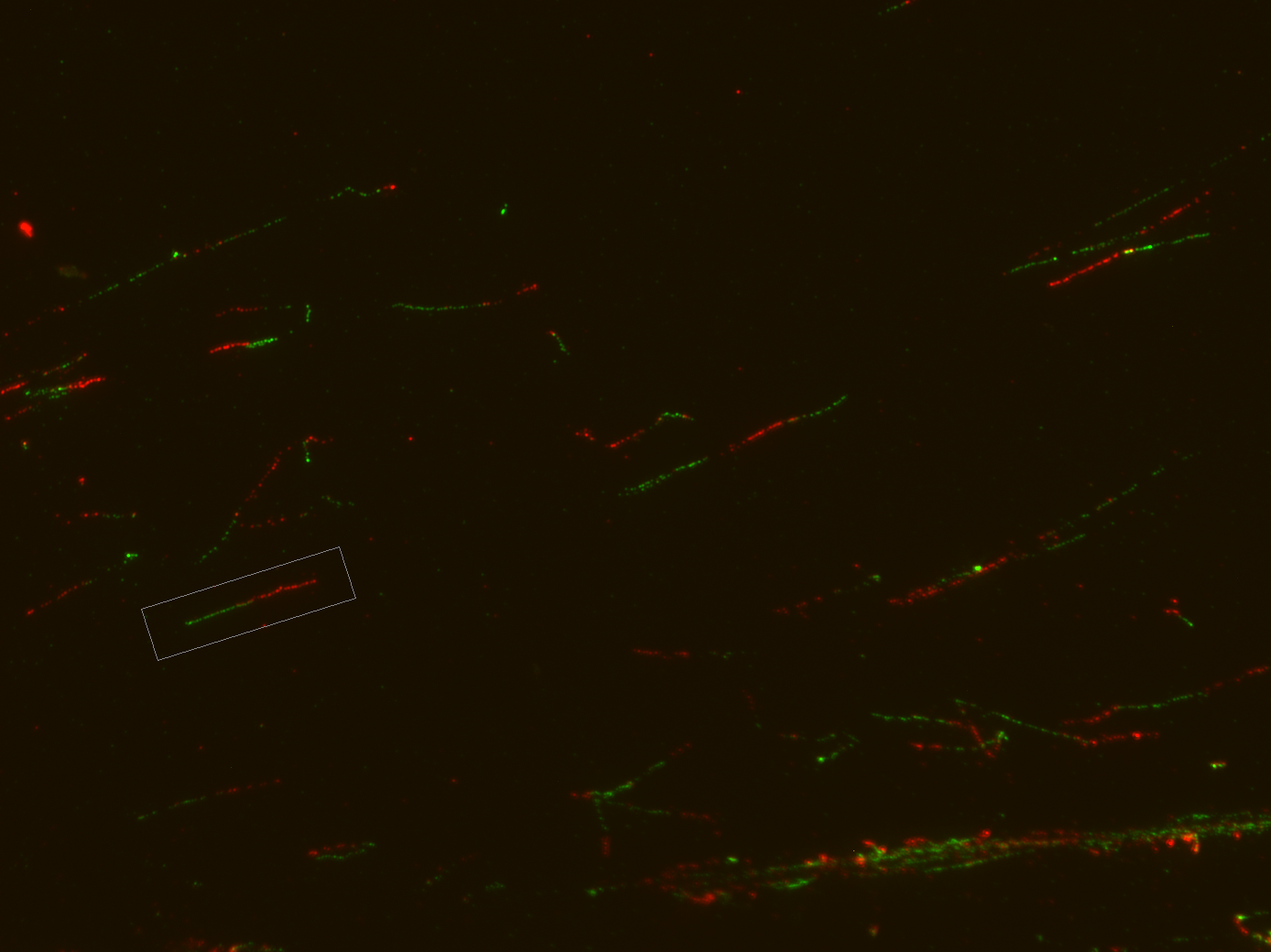

Supplement: Supplementary file 6 — Source data Fig. 3 [file 44319_2025_497_MOESM6_ESM.zip › 3C and 3D/shControl+mirin fork protection with mirin DNA fiber.tif]

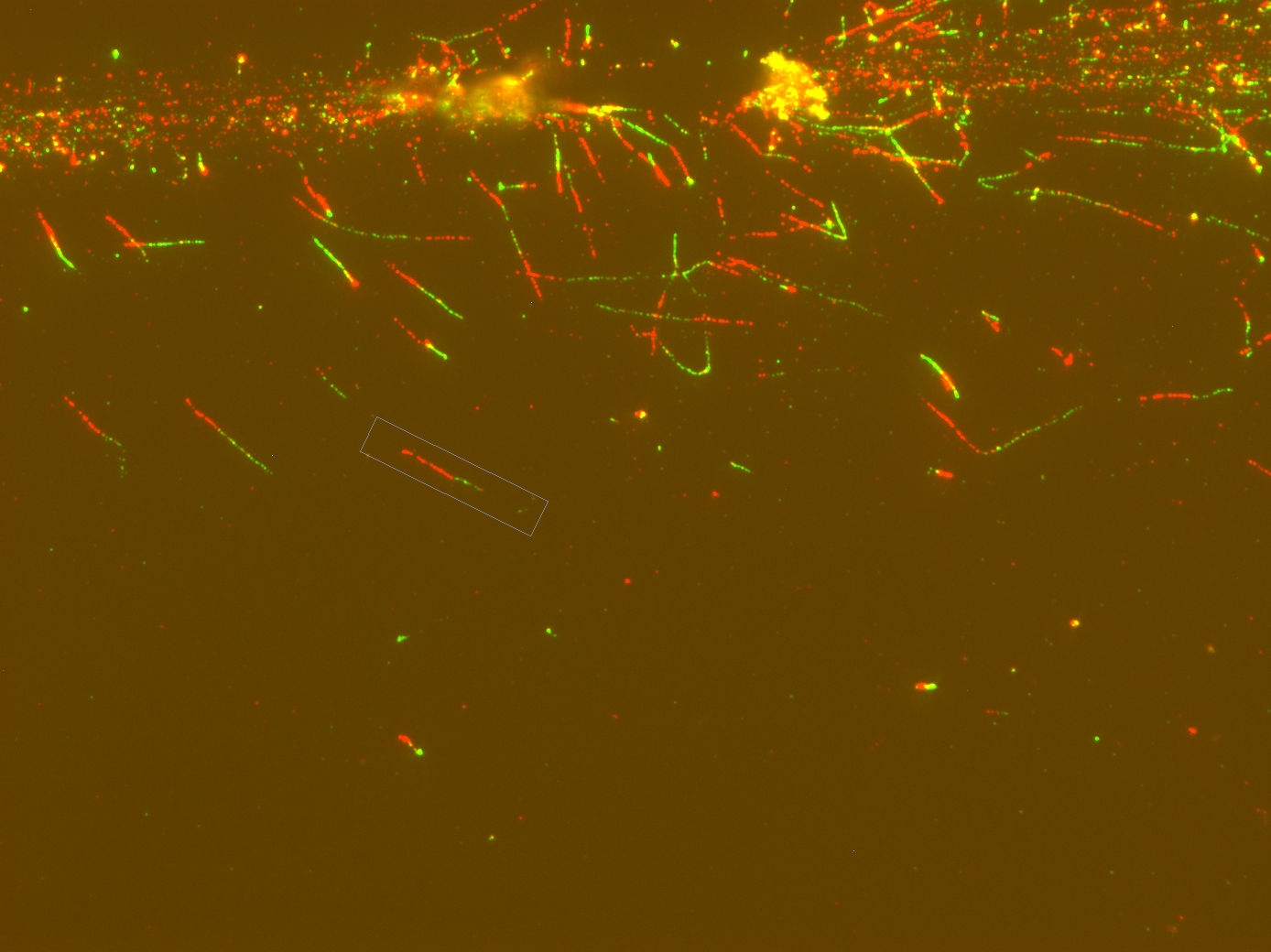

Supplement: Supplementary file 6 — Source data Fig. 3 [file 44319_2025_497_MOESM6_ESM.zip › 3C and 3D/shRNF20-1 fork protection with mirin DNA fiber.tif]

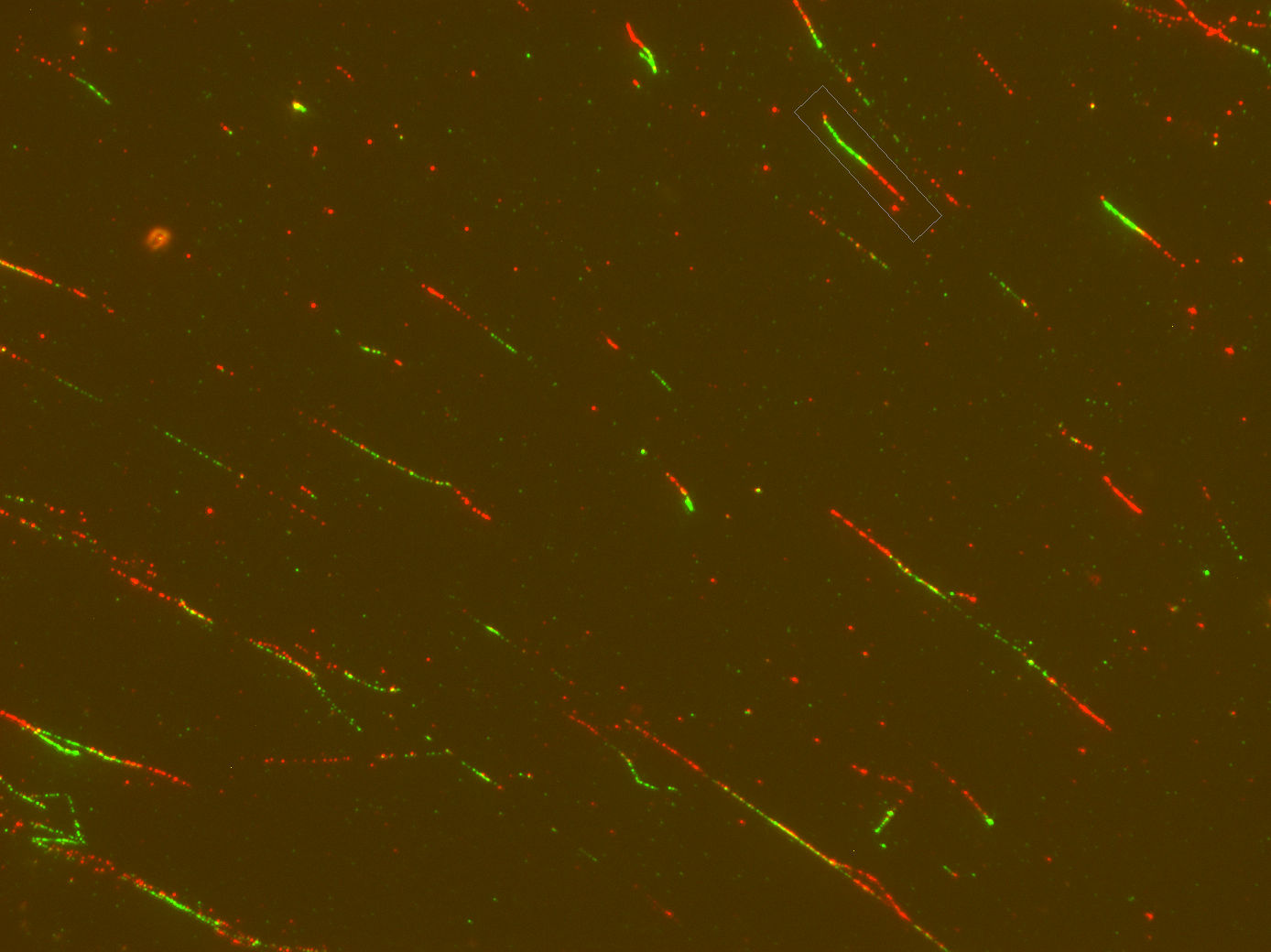

Supplement: Supplementary file 6 — Source data Fig. 3 [file 44319_2025_497_MOESM6_ESM.zip › 3C and 3D/shRNF20-1+mirin fork protection with mirin DNA fiber.tif]

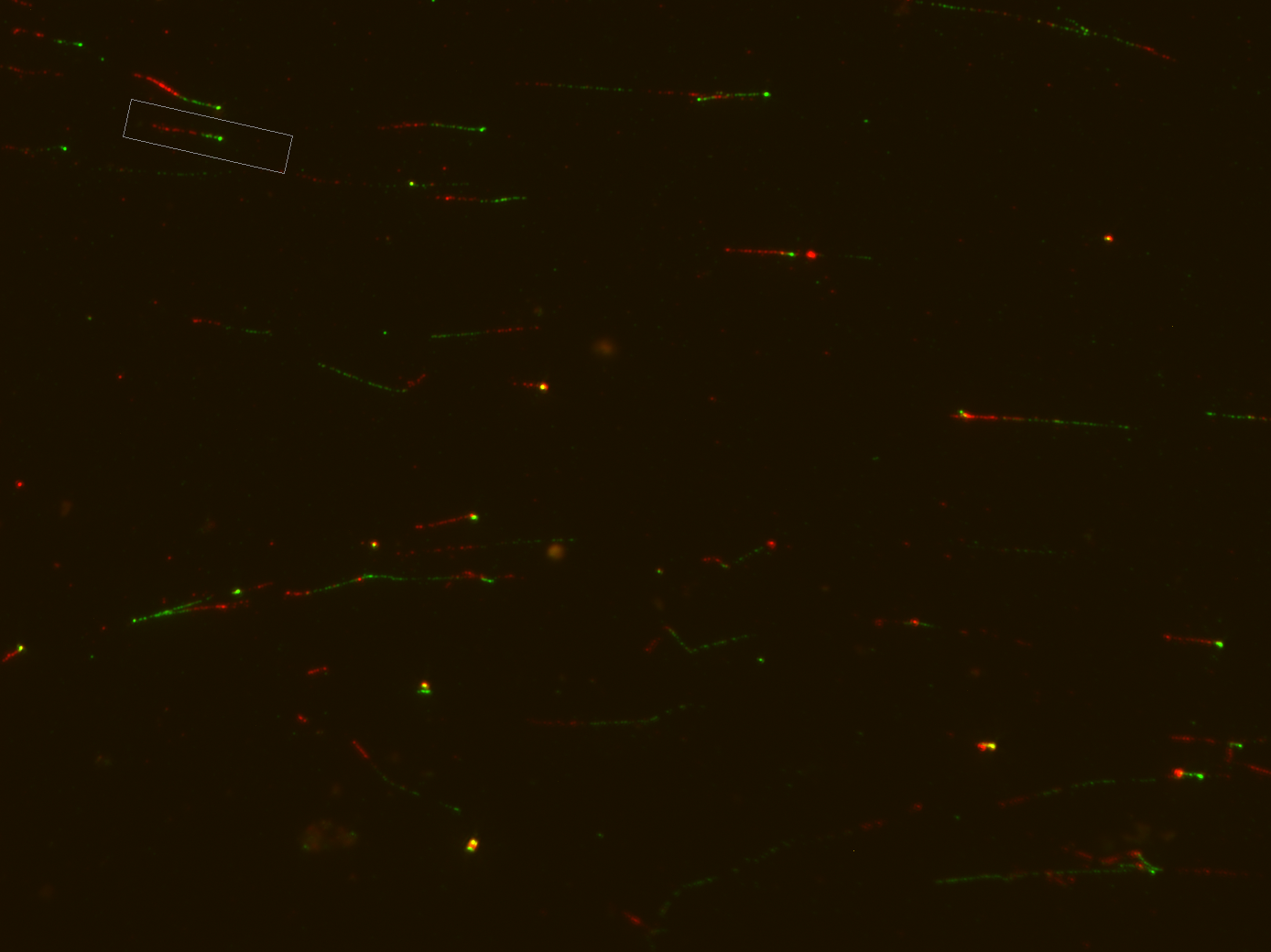

Supplement: Supplementary file 6 — Source data Fig. 3 [file 44319_2025_497_MOESM6_ESM.zip › 3C and 3D/shRNF20-2 fork protection with mirin DNA fiber.tif]

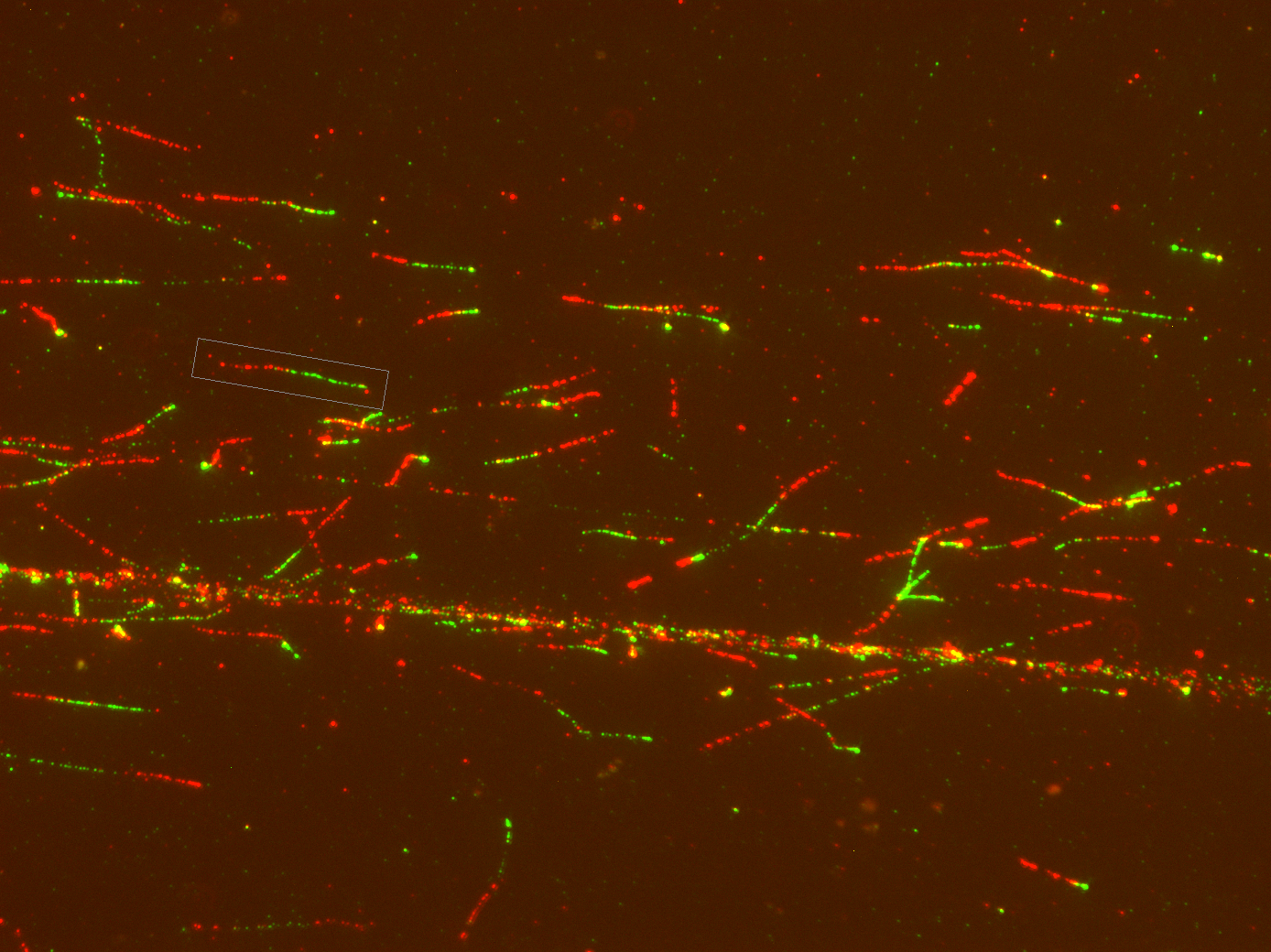

Supplement: Supplementary file 6 — Source data Fig. 3 [file 44319_2025_497_MOESM6_ESM.zip › 3C and 3D/shRNF20-2+mirin fork protection with mirin DNA fiber.tif]

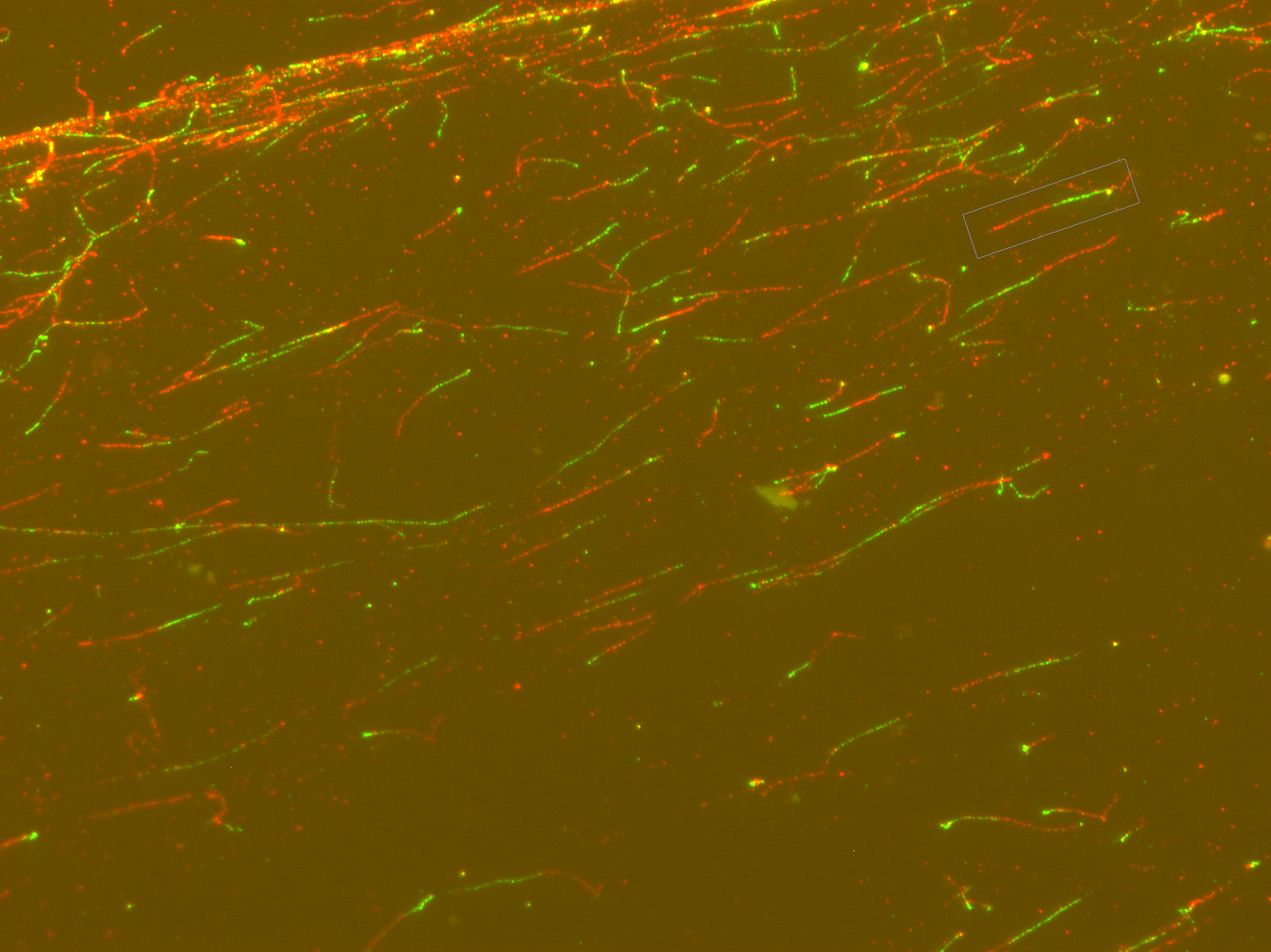

Supplement: Supplementary file 6 — Source data Fig. 3 [file 44319_2025_497_MOESM6_ESM.zip › 3E and 3F/shControl fork protection with C5 DNA fiber.tif]

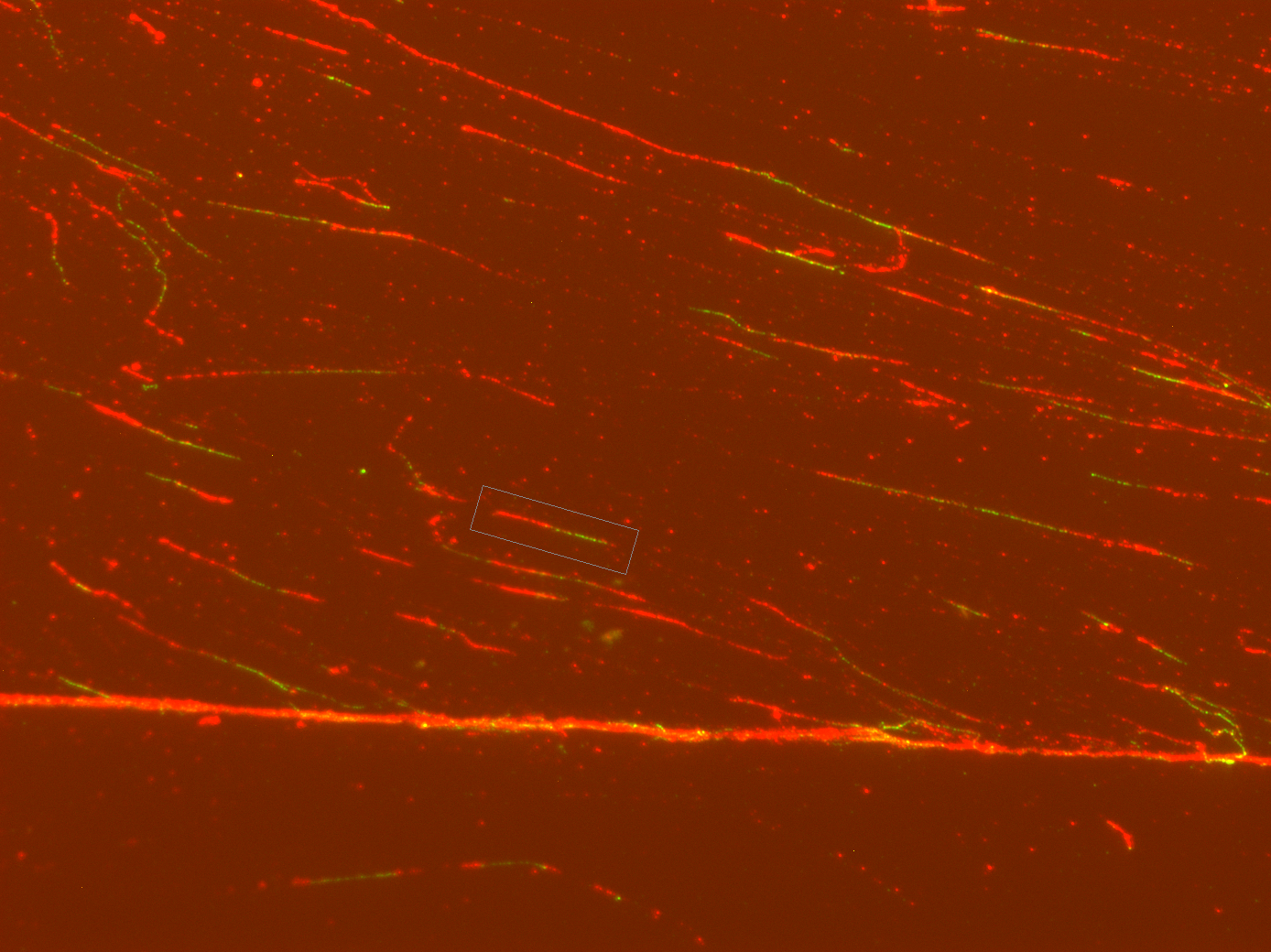

Supplement: Supplementary file 6 — Source data Fig. 3 [file 44319_2025_497_MOESM6_ESM.zip › 3E and 3F/shControl+C5 fork protection with C5 DNA fiber.tif]

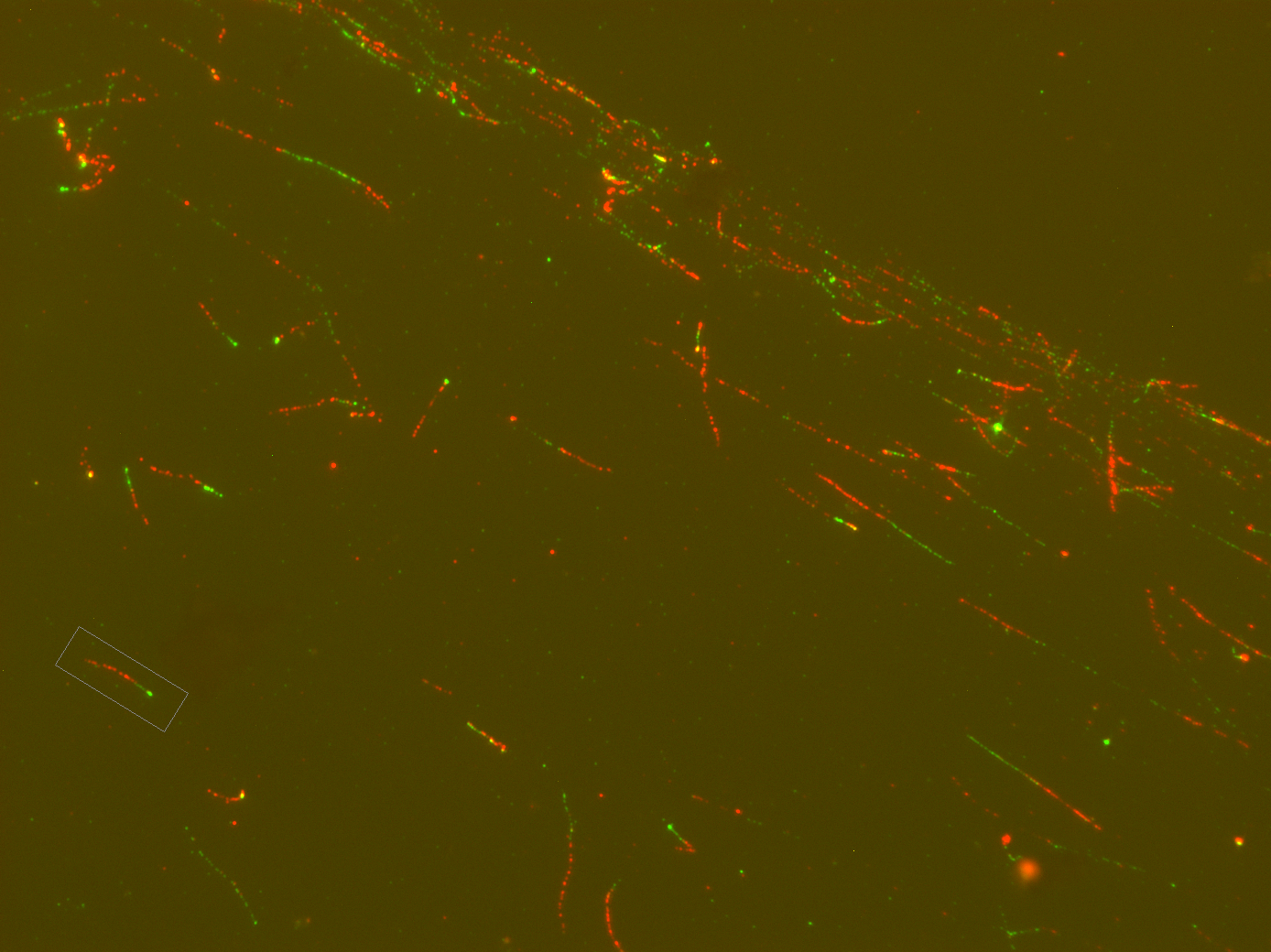

Supplement: Supplementary file 6 — Source data Fig. 3 [file 44319_2025_497_MOESM6_ESM.zip › 3E and 3F/shRNF20 fork protection with C5 DNA fiber.tif]

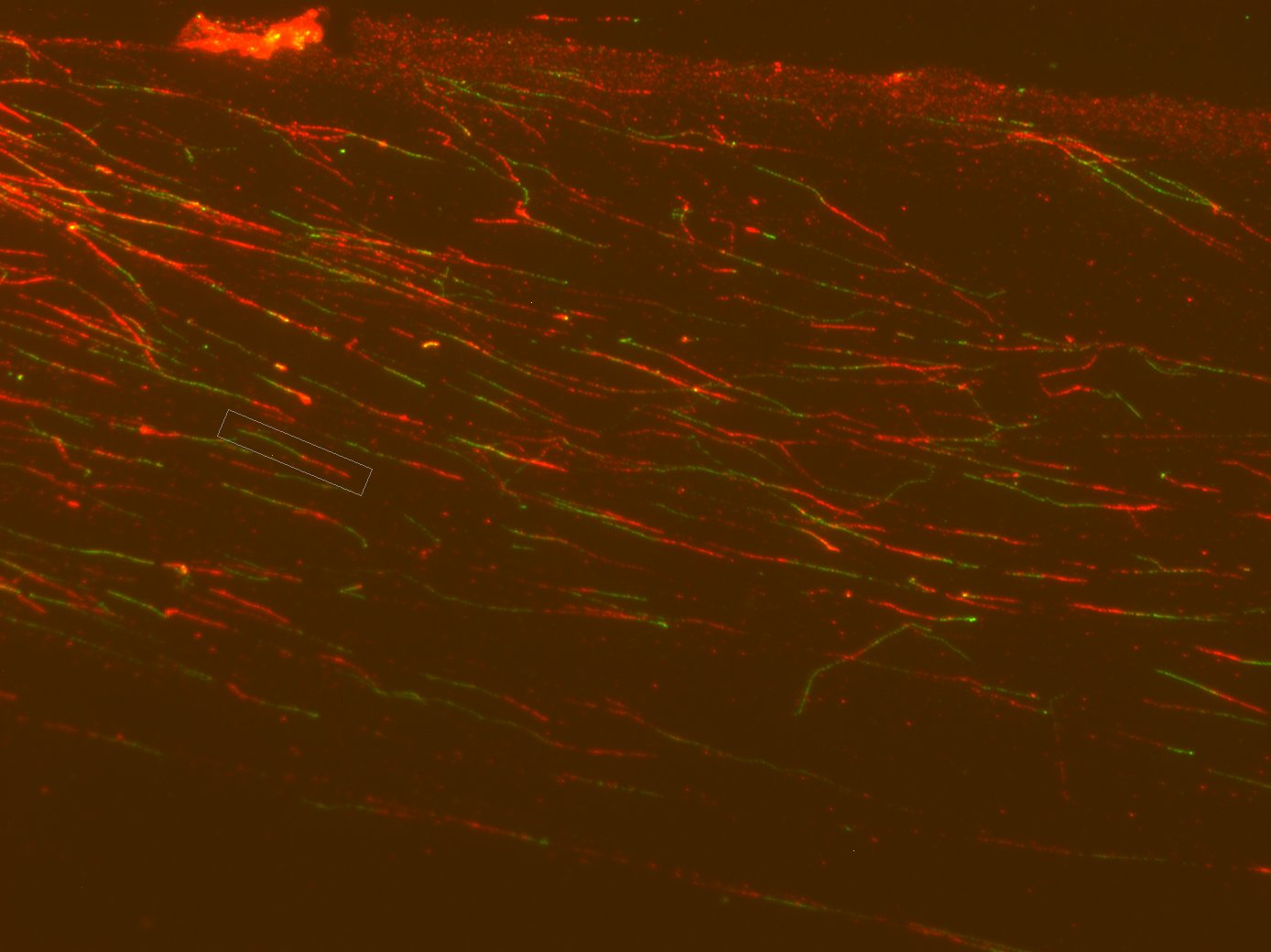

Supplement: Supplementary file 6 — Source data Fig. 3 [file 44319_2025_497_MOESM6_ESM.zip › 3E and 3F/shRNF20+C5 fork protection with C5 DNA fiber.tif]

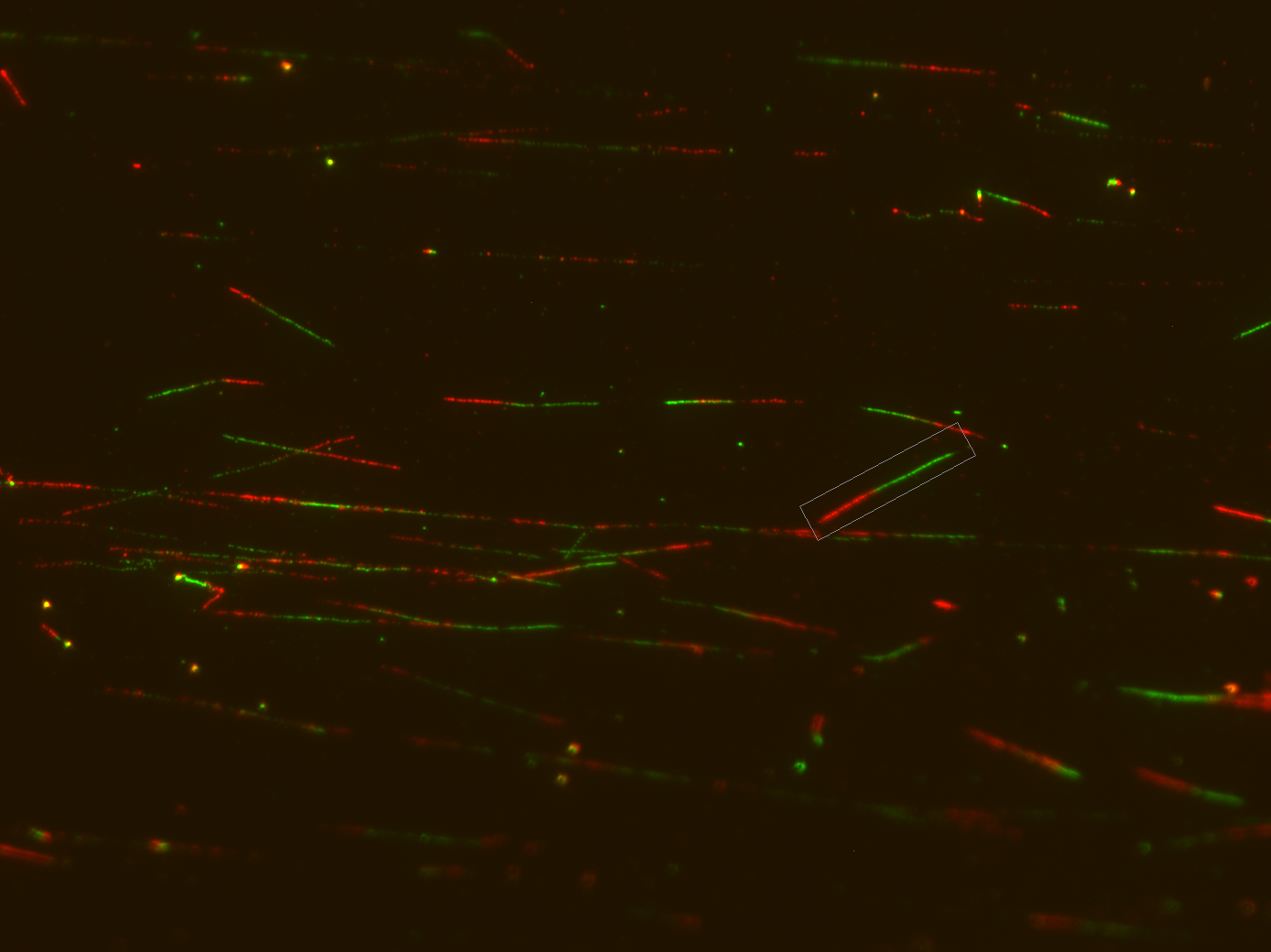

Supplement: Supplementary file 6 — Source data Fig. 3 [file 44319_2025_497_MOESM6_ESM.zip › 3G and 3H/shControl DNA fiber.tif]

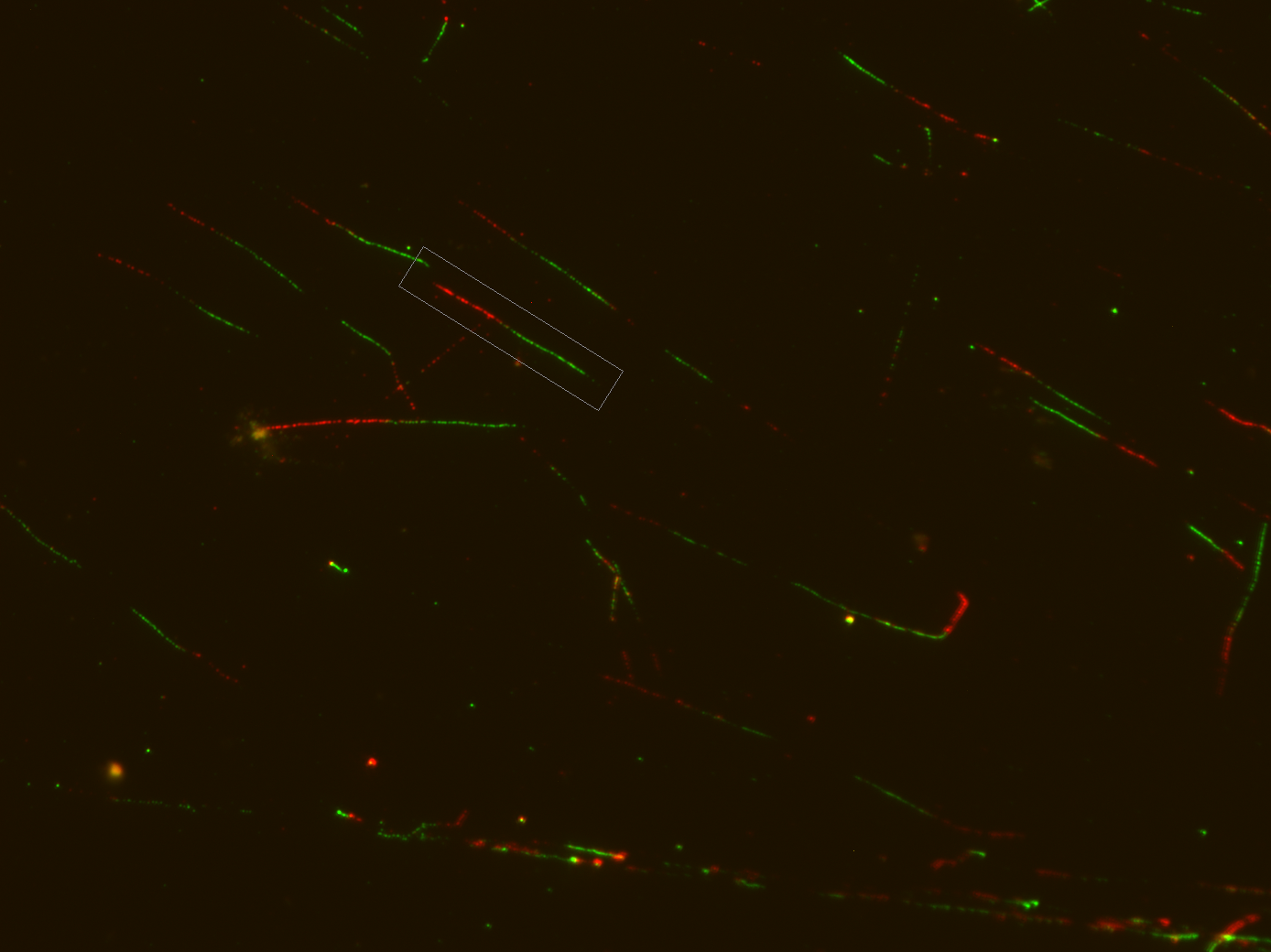

Supplement: Supplementary file 6 — Source data Fig. 3 [file 44319_2025_497_MOESM6_ESM.zip › 3G and 3H/shHLTF DNA fiber.tif]

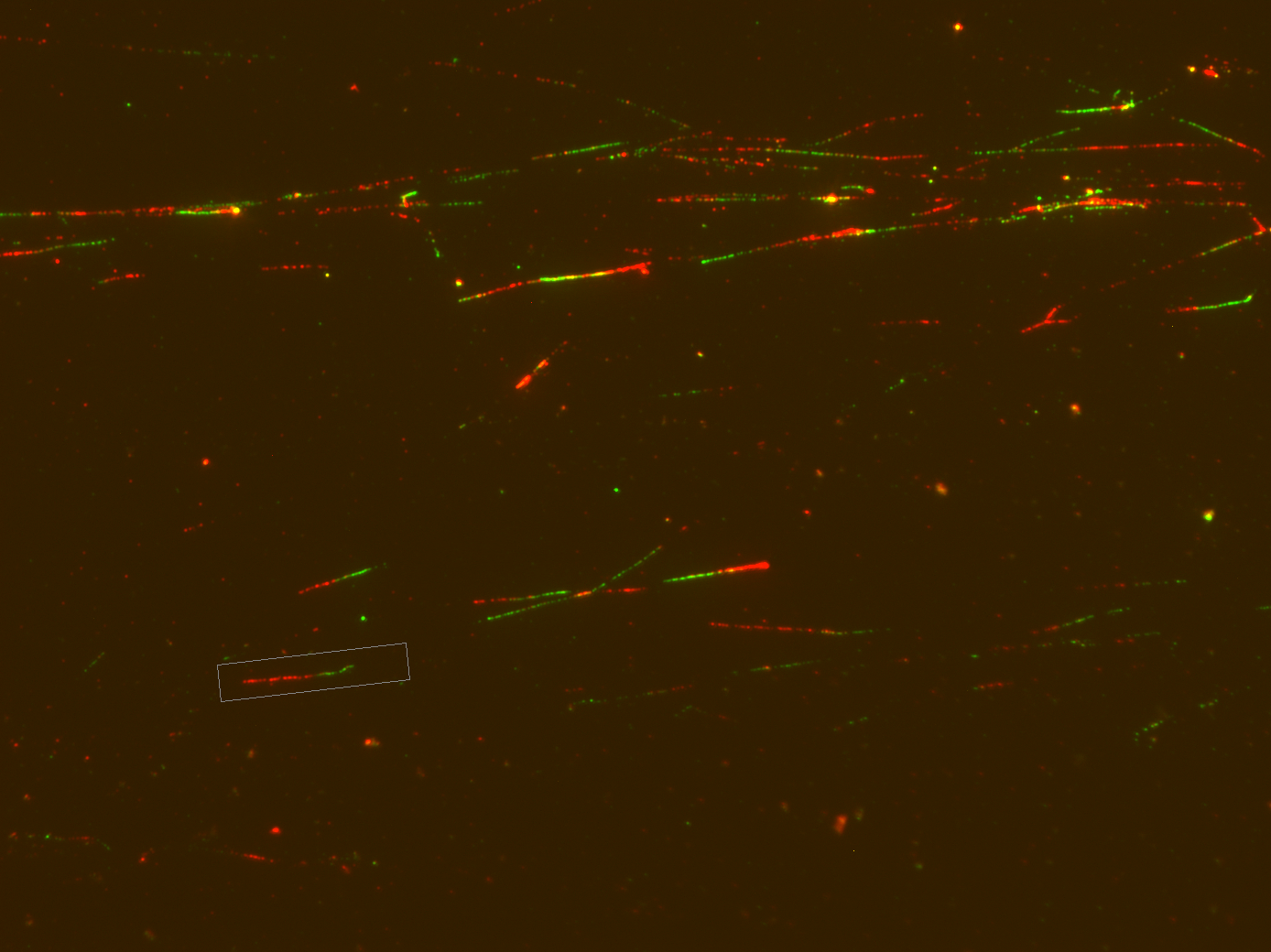

Supplement: Supplementary file 6 — Source data Fig. 3 [file 44319_2025_497_MOESM6_ESM.zip › 3G and 3H/shRNF20 DNA fiber.tif]

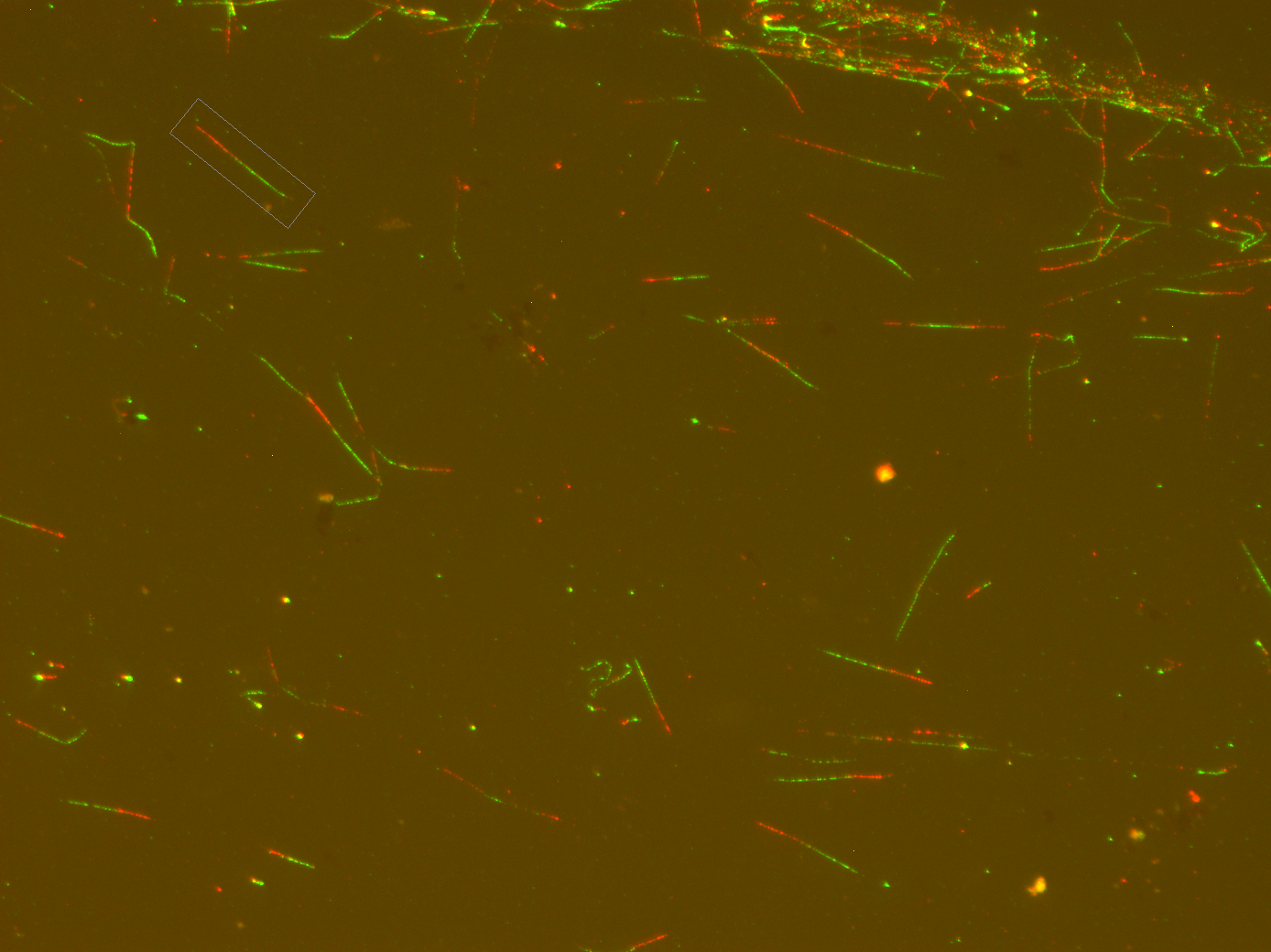

Supplement: Supplementary file 6 — Source data Fig. 3 [file 44319_2025_497_MOESM6_ESM.zip › 3G and 3H/shRNF20+shHLTF.tif]

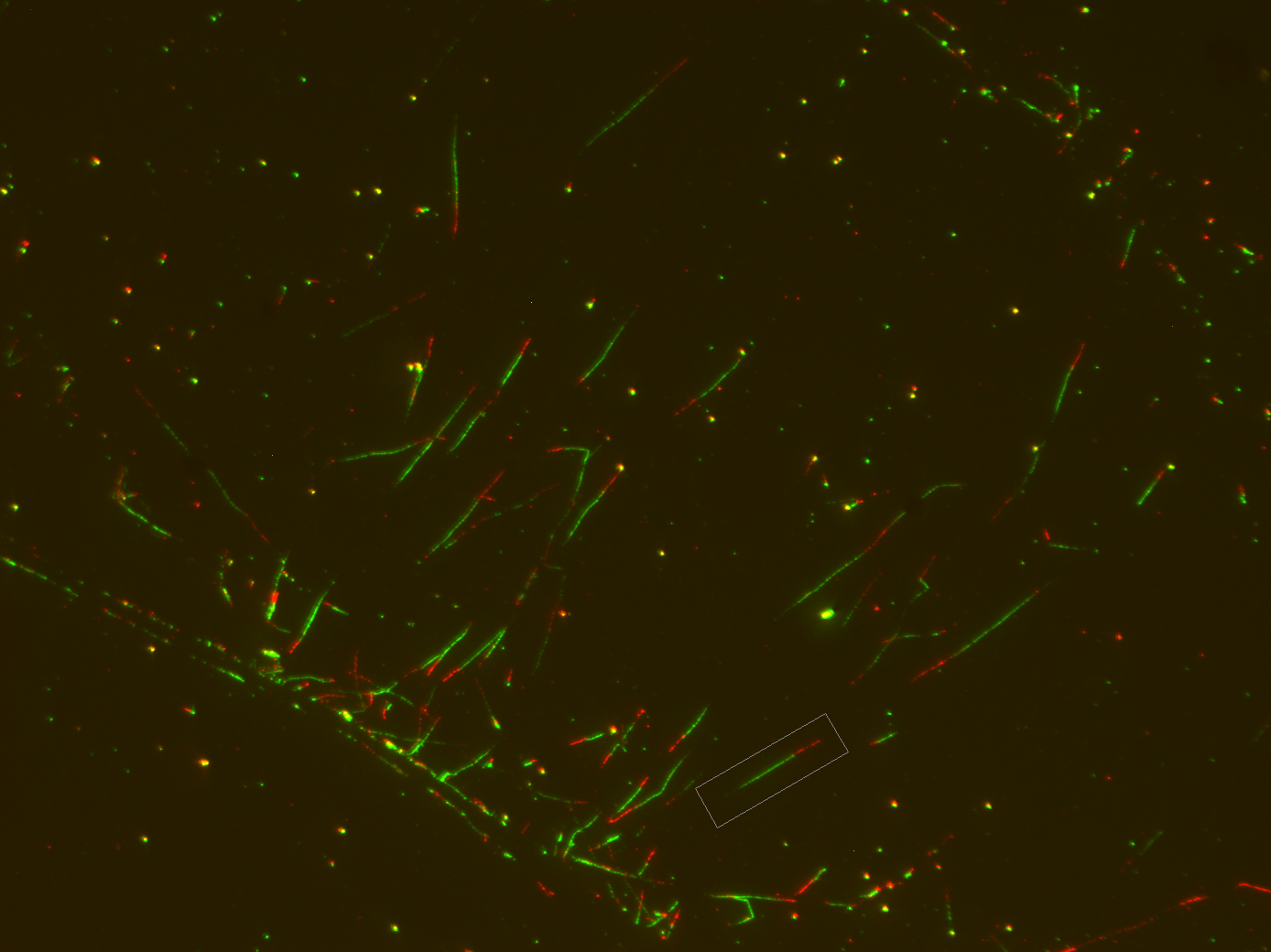

Supplement: Supplementary file 6 — Source data Fig. 3 [file 44319_2025_497_MOESM6_ESM.zip › 3G and 3H/shRNF20+shSMARCAL1 DNA fiber.tif]

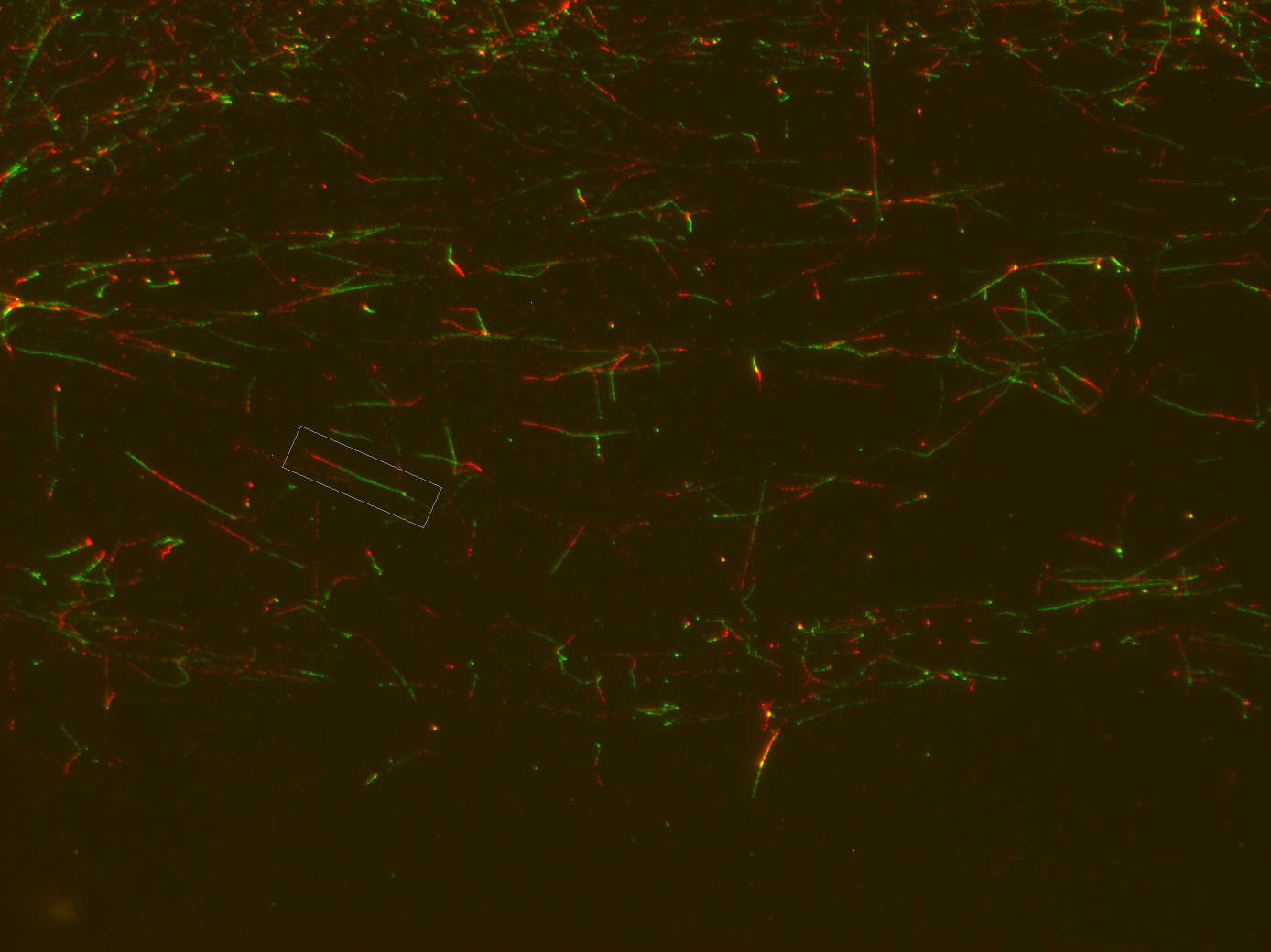

Supplement: Supplementary file 6 — Source data Fig. 3 [file 44319_2025_497_MOESM6_ESM.zip › 3G and 3H/shRNF20+shZRANB3 DNA fiber.tif]

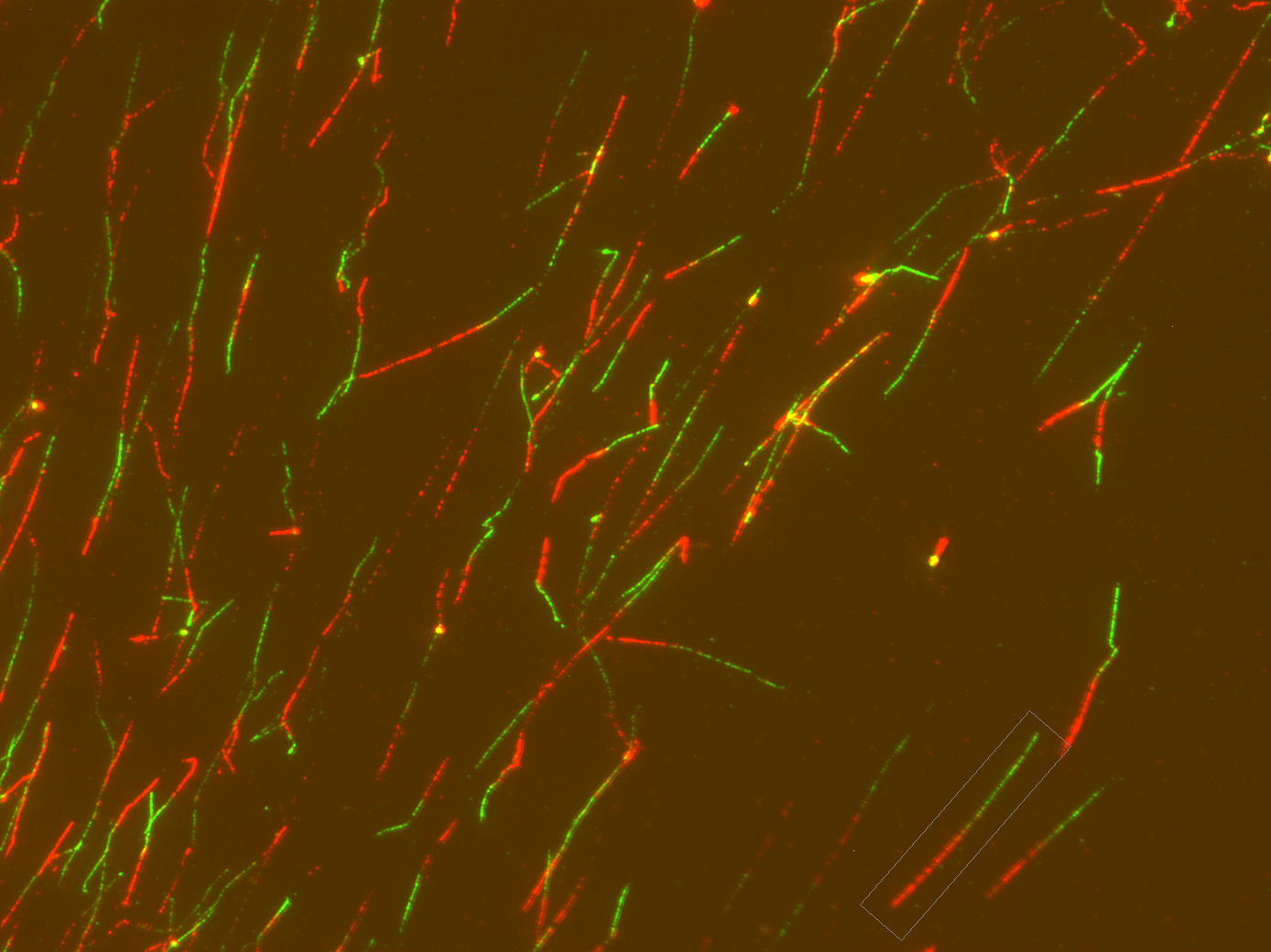

Supplement: Supplementary file 6 — Source data Fig. 3 [file 44319_2025_497_MOESM6_ESM.zip › 3G and 3H/shSMARCAL1 DNA fiber.tif]

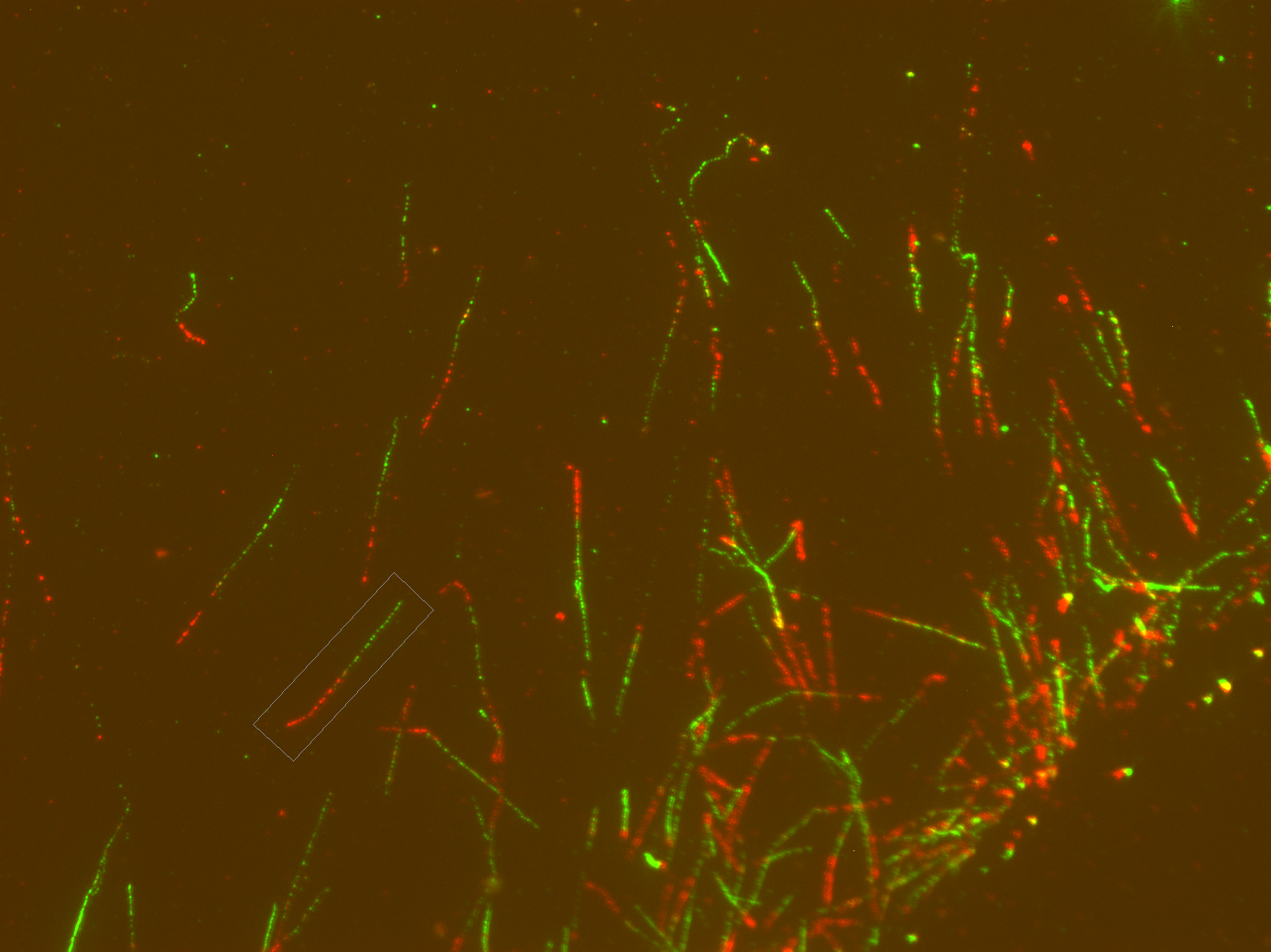

Supplement: Supplementary file 6 — Source data Fig. 3 [file 44319_2025_497_MOESM6_ESM.zip › 3G and 3H/shZRANB3 DNA fiber.tif]

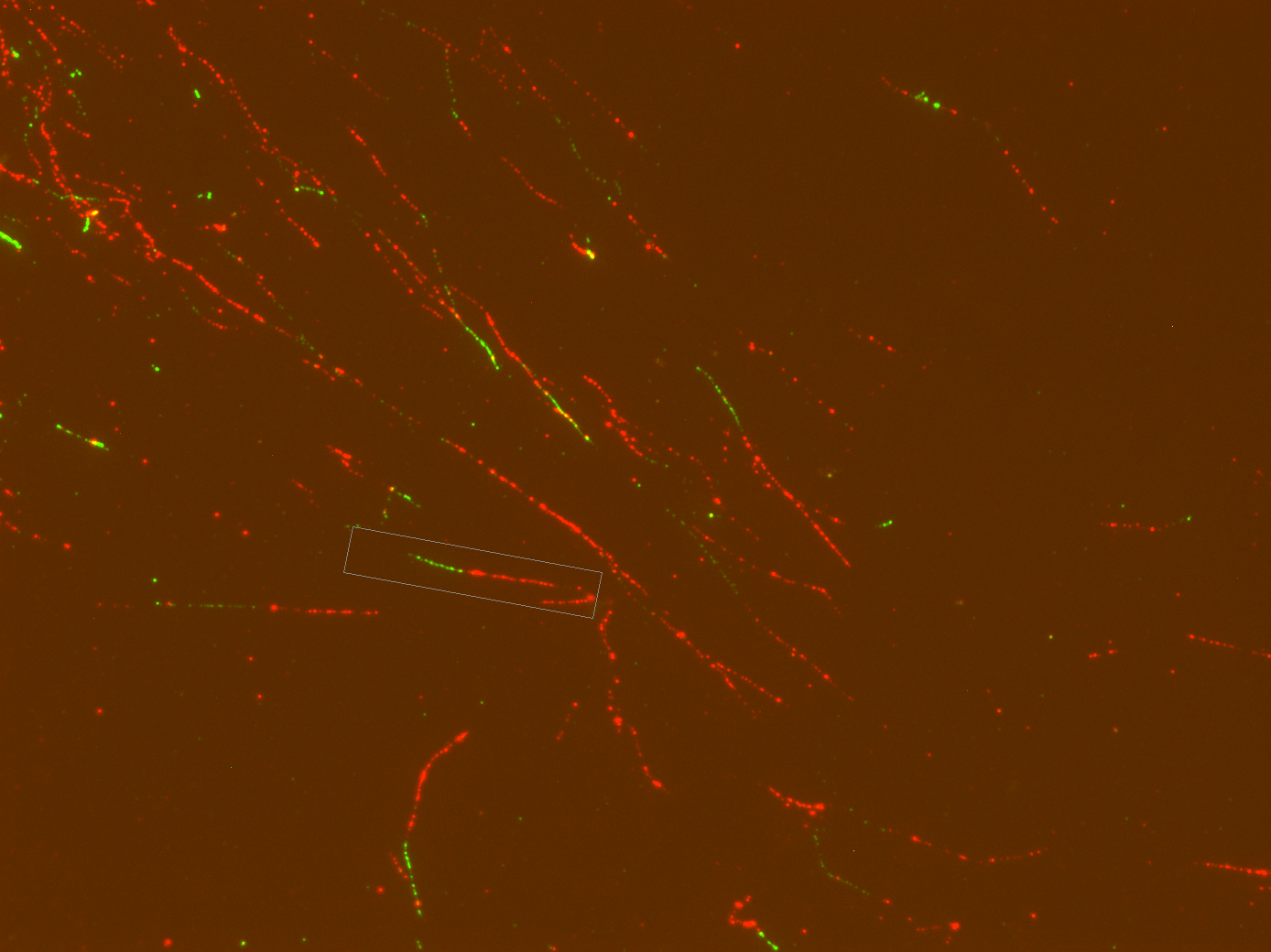

Supplement: Supplementary file 6 — Source data Fig. 3 [file 44319_2025_497_MOESM6_ESM.zip › 3I/Restarted fork representative DNA fiber.tif]

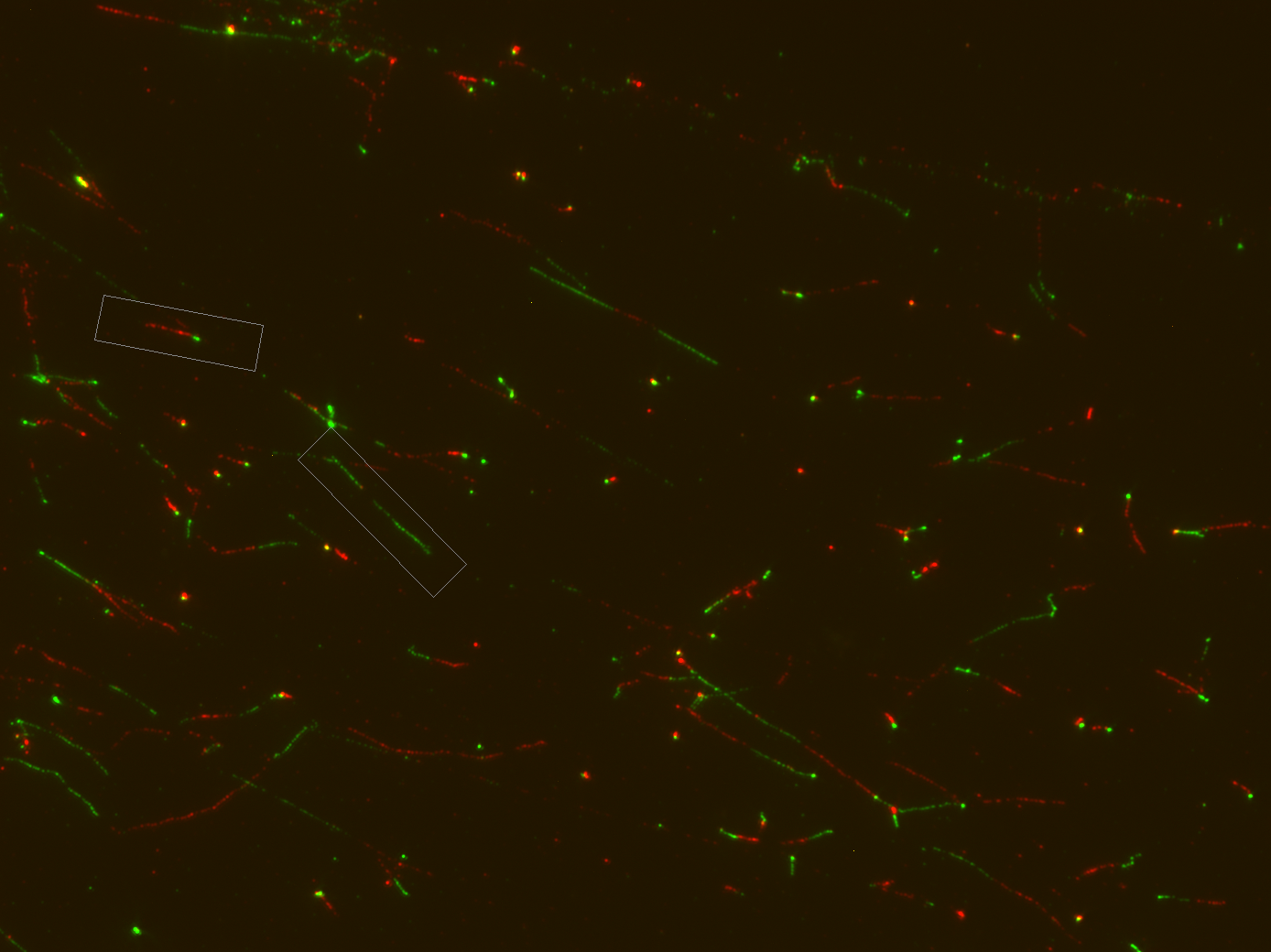

Supplement: Supplementary file 6 — Source data Fig. 3 [file 44319_2025_497_MOESM6_ESM.zip › 3I/Stalled fork and new origin firing representative DNA fiber.tif]

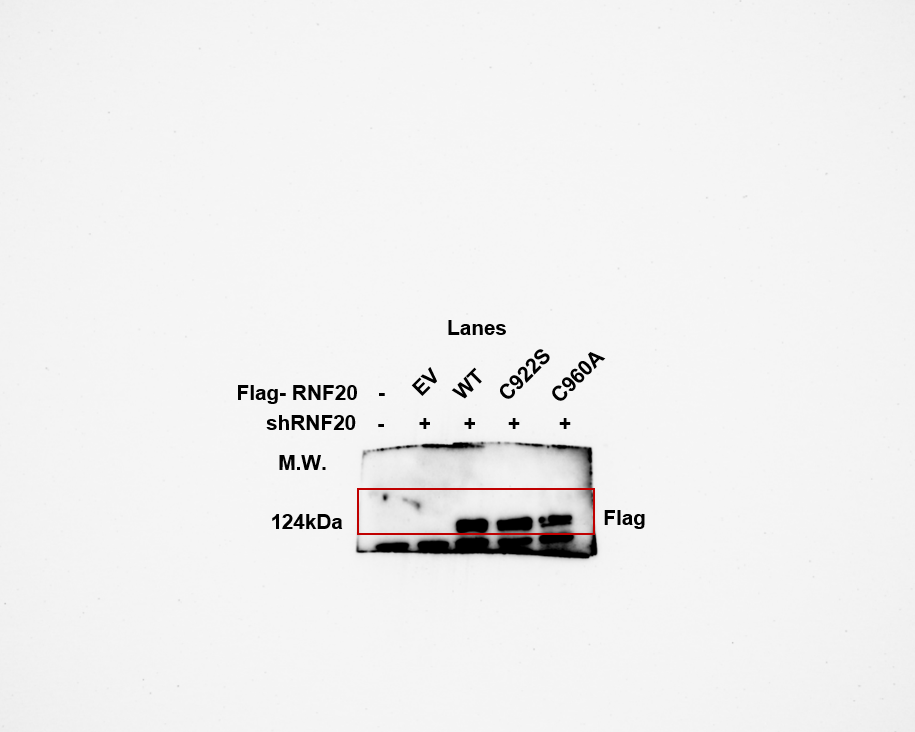

Supplement: Supplementary file 8 — Source data Fig. 5 [file 44319_2025_497_MOESM8_ESM.zip › 5B/Flag catalytic mutants western blot.tif]

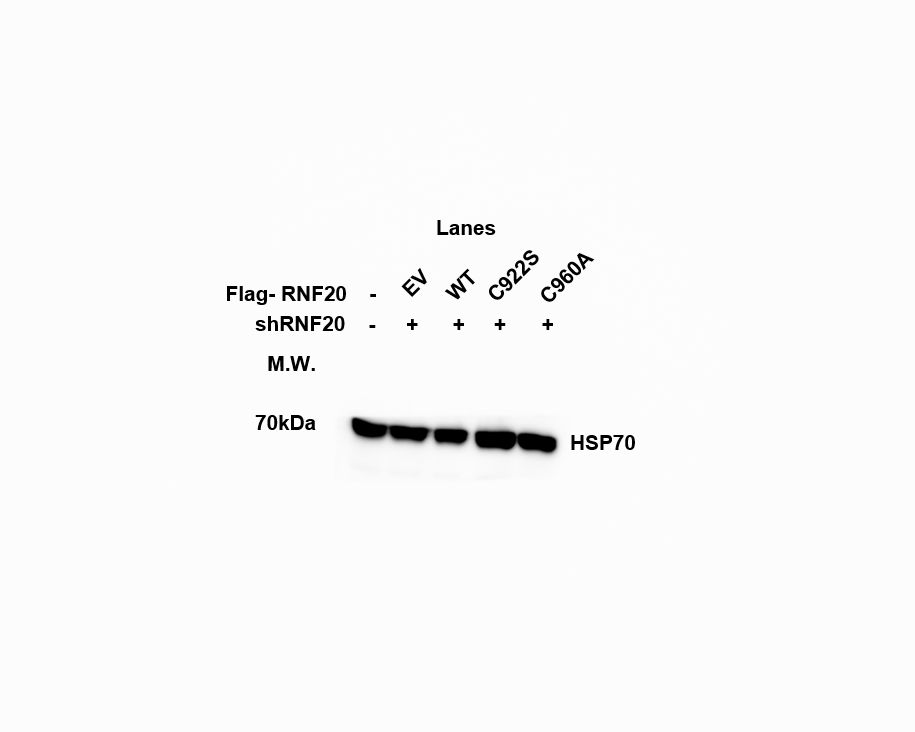

Supplement: Supplementary file 8 — Source data Fig. 5 [file 44319_2025_497_MOESM8_ESM.zip › 5B/HSP70 loading control western blot.tif]

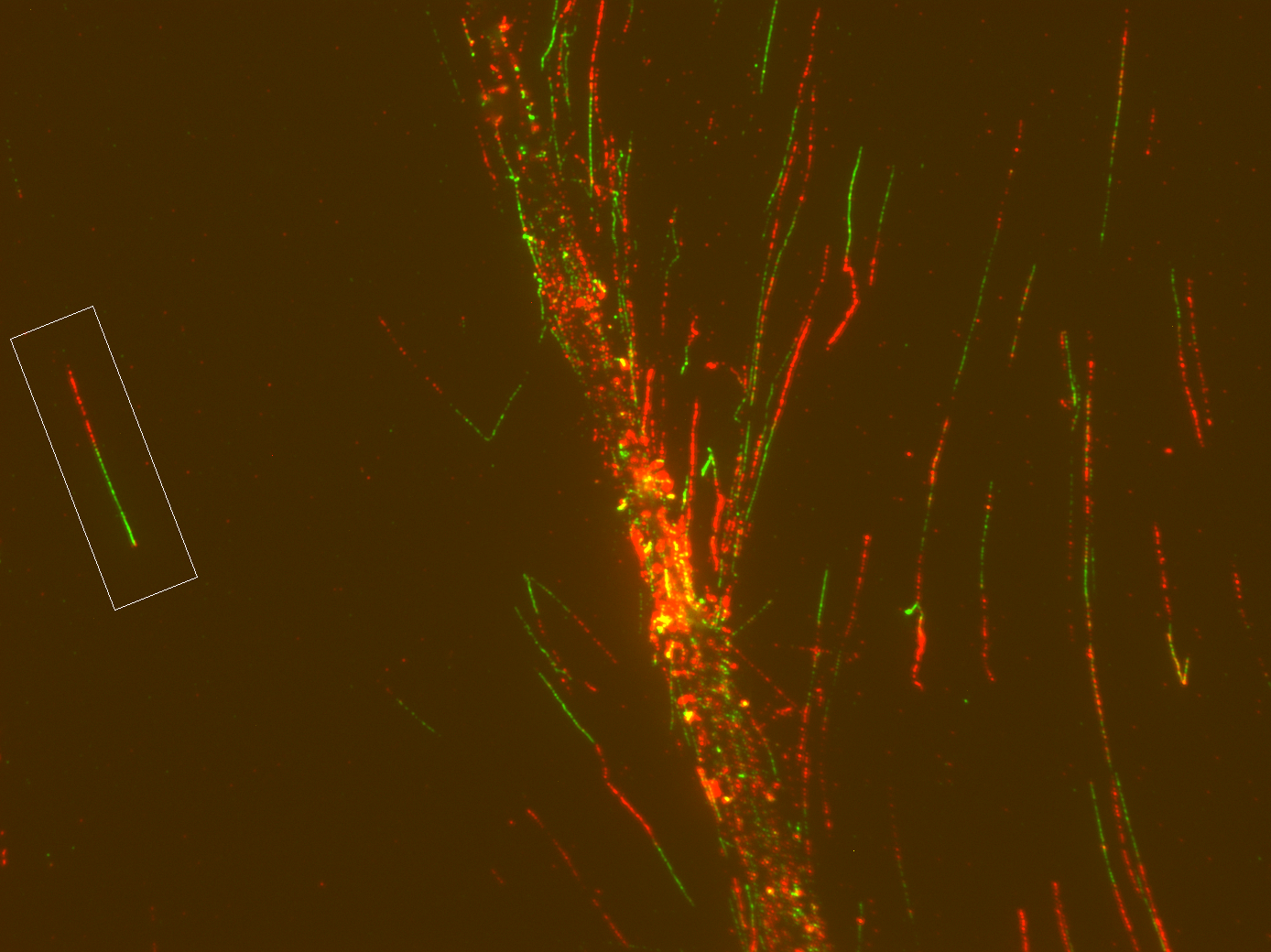

Supplement: Supplementary file 8 — Source data Fig. 5 [file 44319_2025_497_MOESM8_ESM.zip › 5C and 5D/shControl DNA fiber.tif]

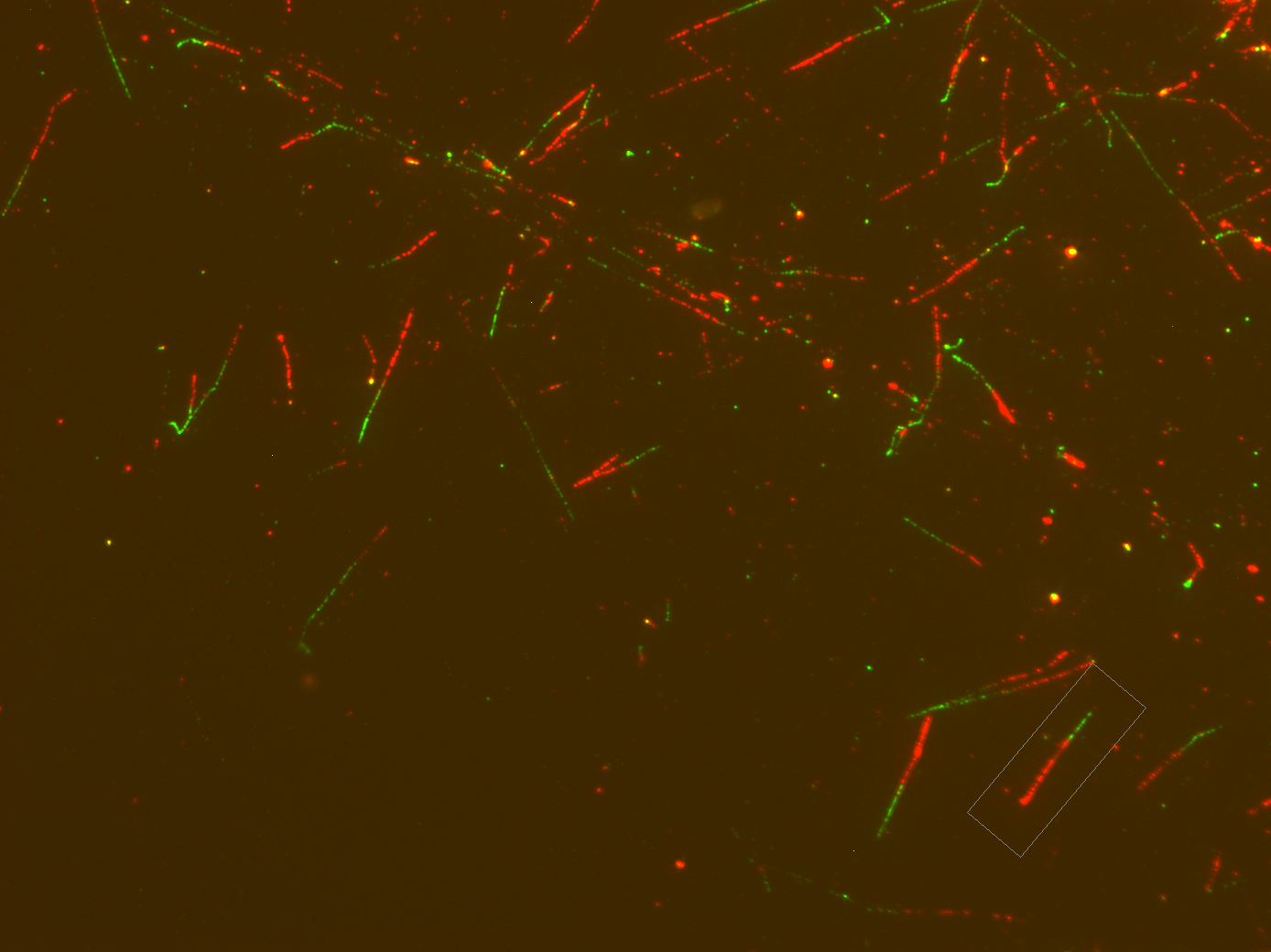

Supplement: Supplementary file 8 — Source data Fig. 5 [file 44319_2025_497_MOESM8_ESM.zip › 5C and 5D/shRNF20 + C960A RNF20 DNA fiber.tif]

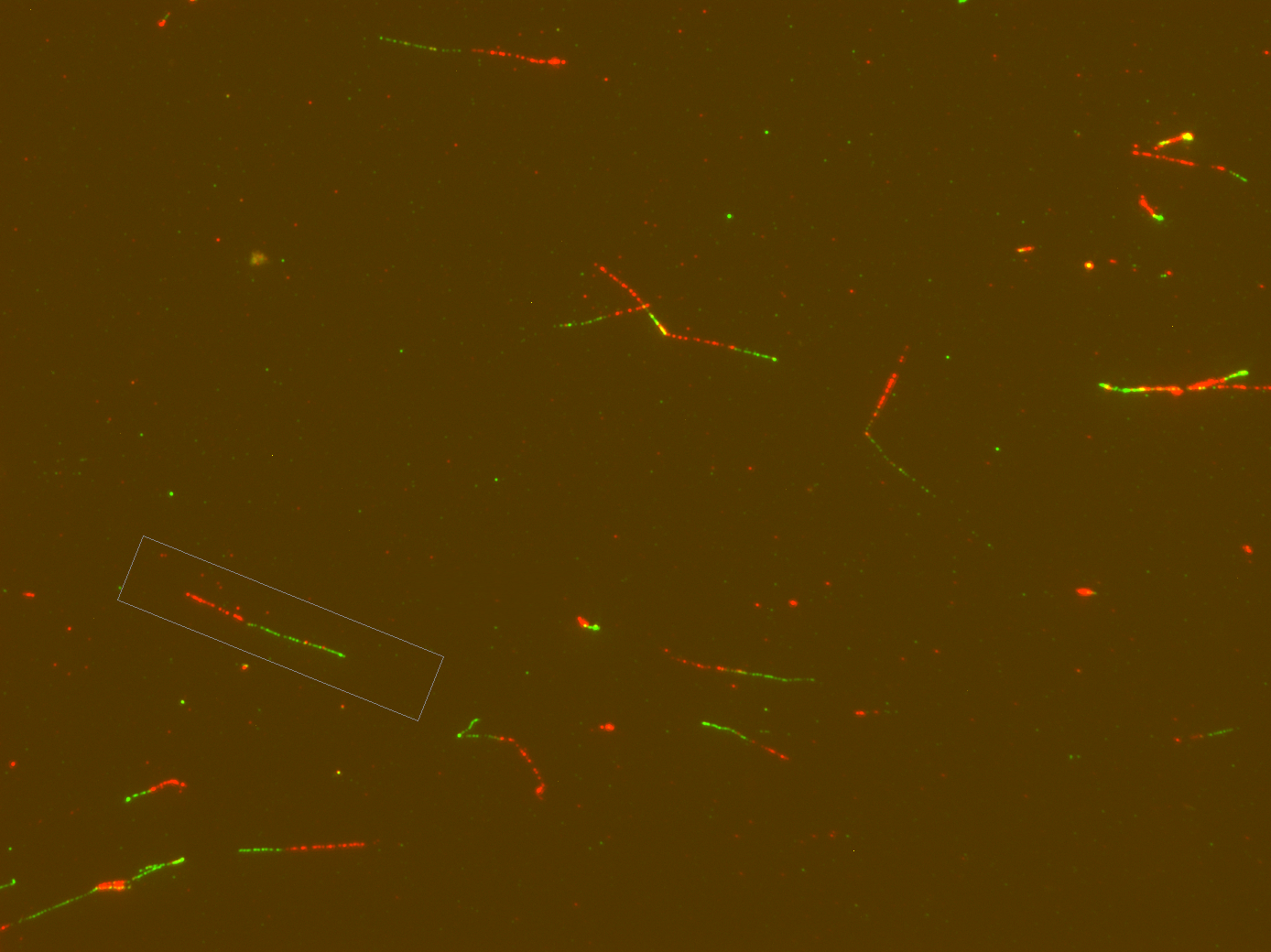

Supplement: Supplementary file 8 — Source data Fig. 5 [file 44319_2025_497_MOESM8_ESM.zip › 5C and 5D/shRNF20 + WT RNF20 DNA fiber.tif]

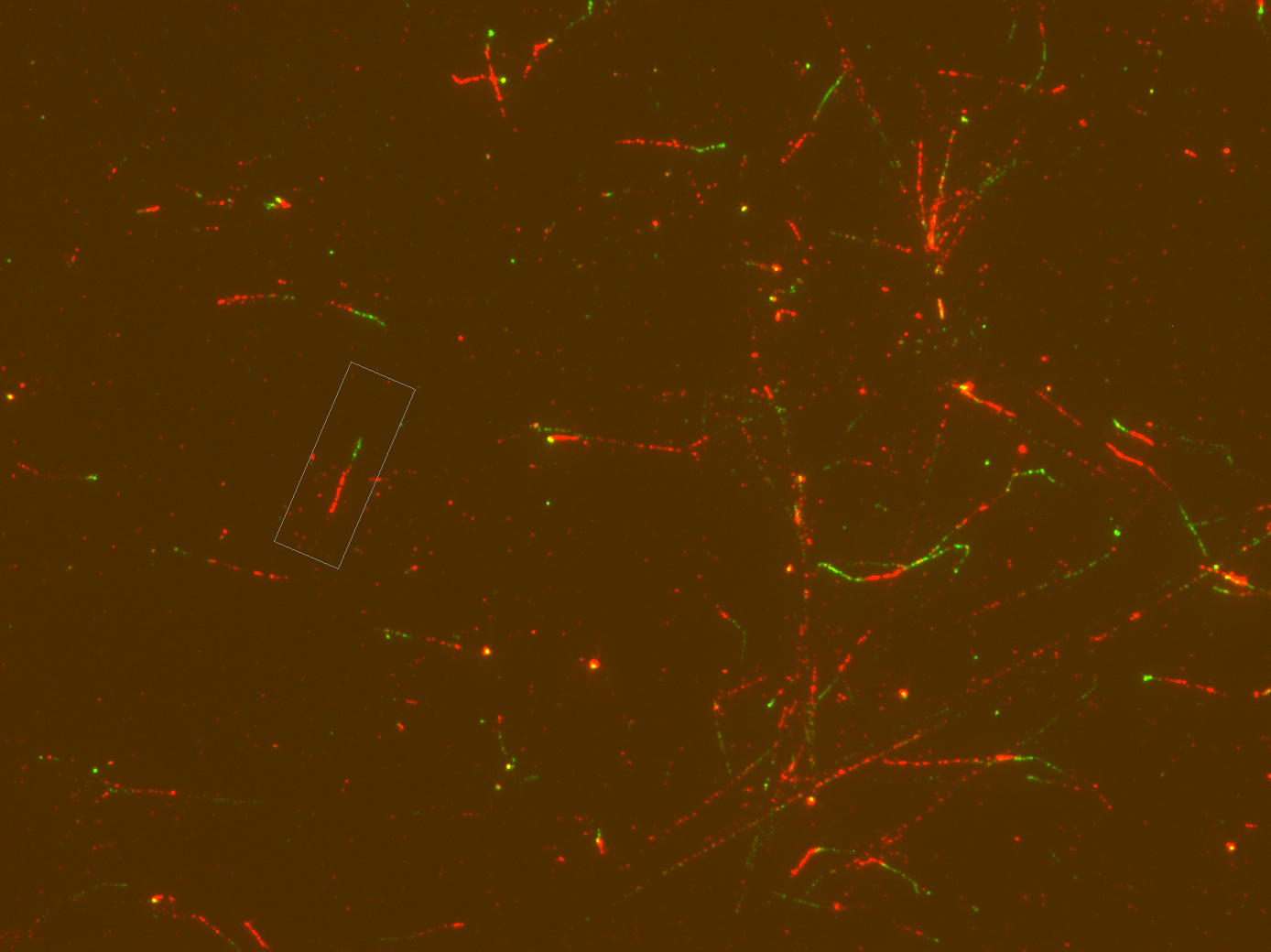

Supplement: Supplementary file 8 — Source data Fig. 5 [file 44319_2025_497_MOESM8_ESM.zip › 5C and 5D/shRNF20+ C922S DNA fiber.tif]

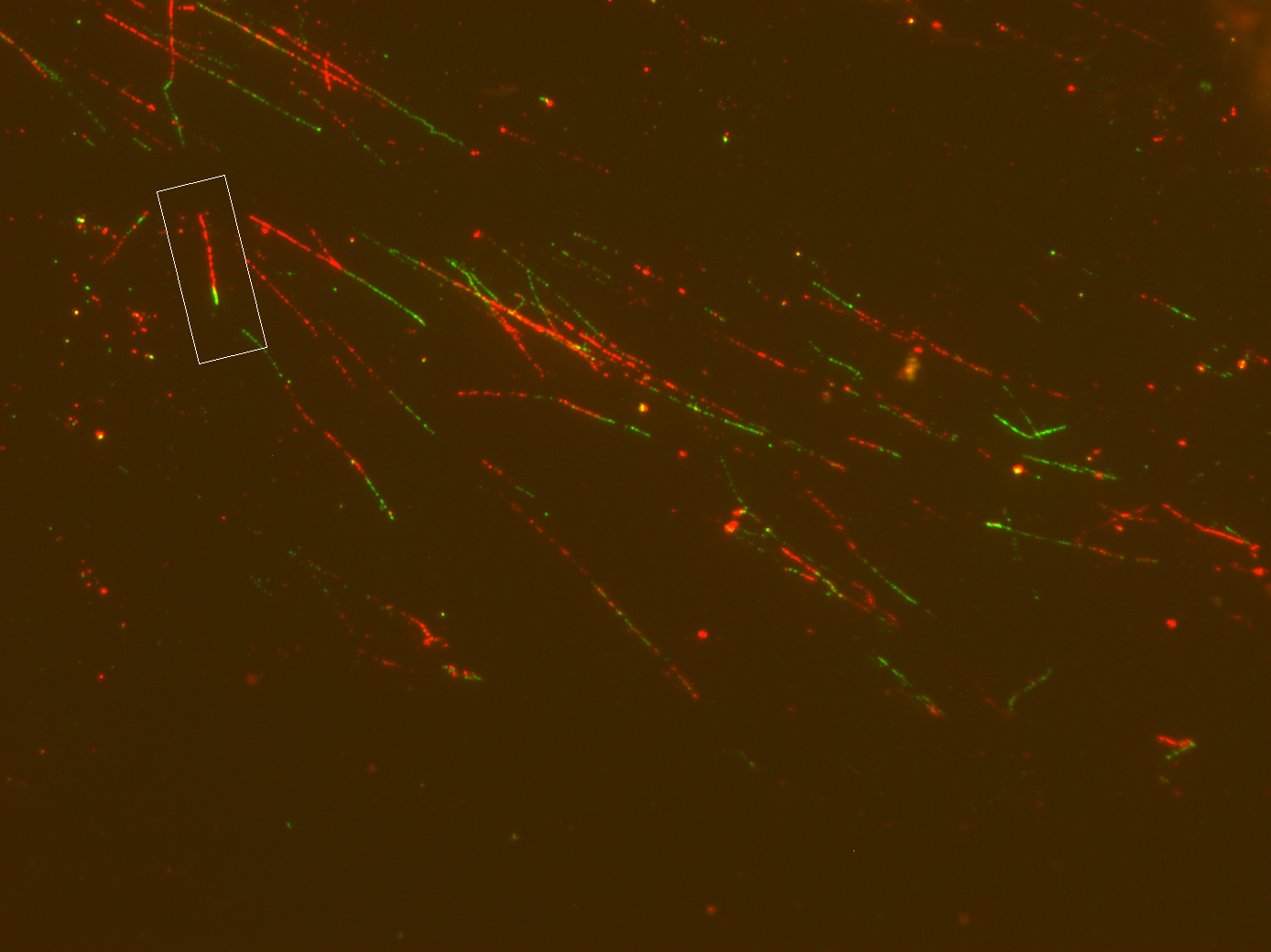

Supplement: Supplementary file 8 — Source data Fig. 5 [file 44319_2025_497_MOESM8_ESM.zip › 5C and 5D/shRNF20+ EV.tif]

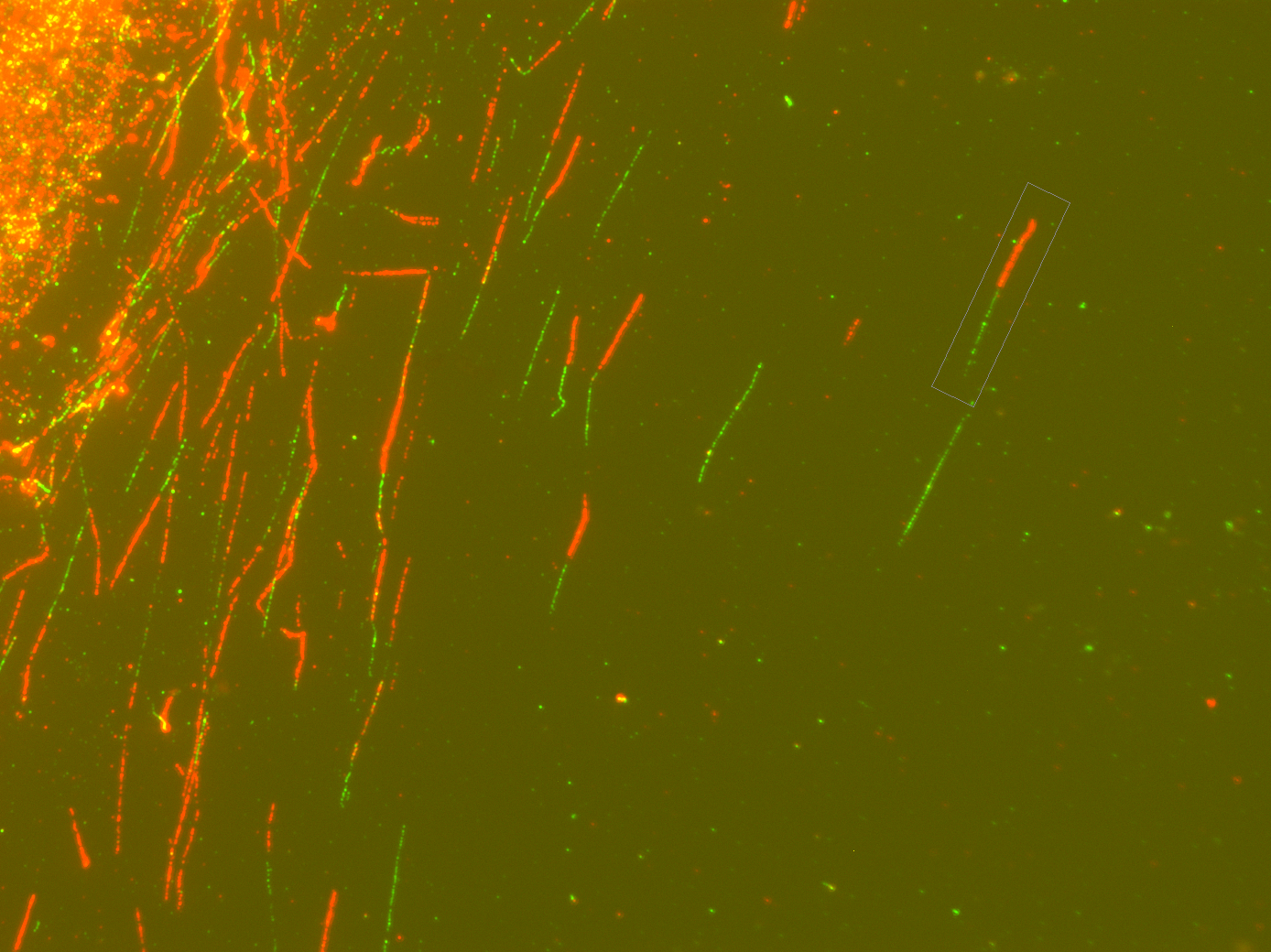

Supplement: Supplementary file 8 — Source data Fig. 5 [file 44319_2025_497_MOESM8_ESM.zip › 5E and 5F/shControl DNA fiber.tif]

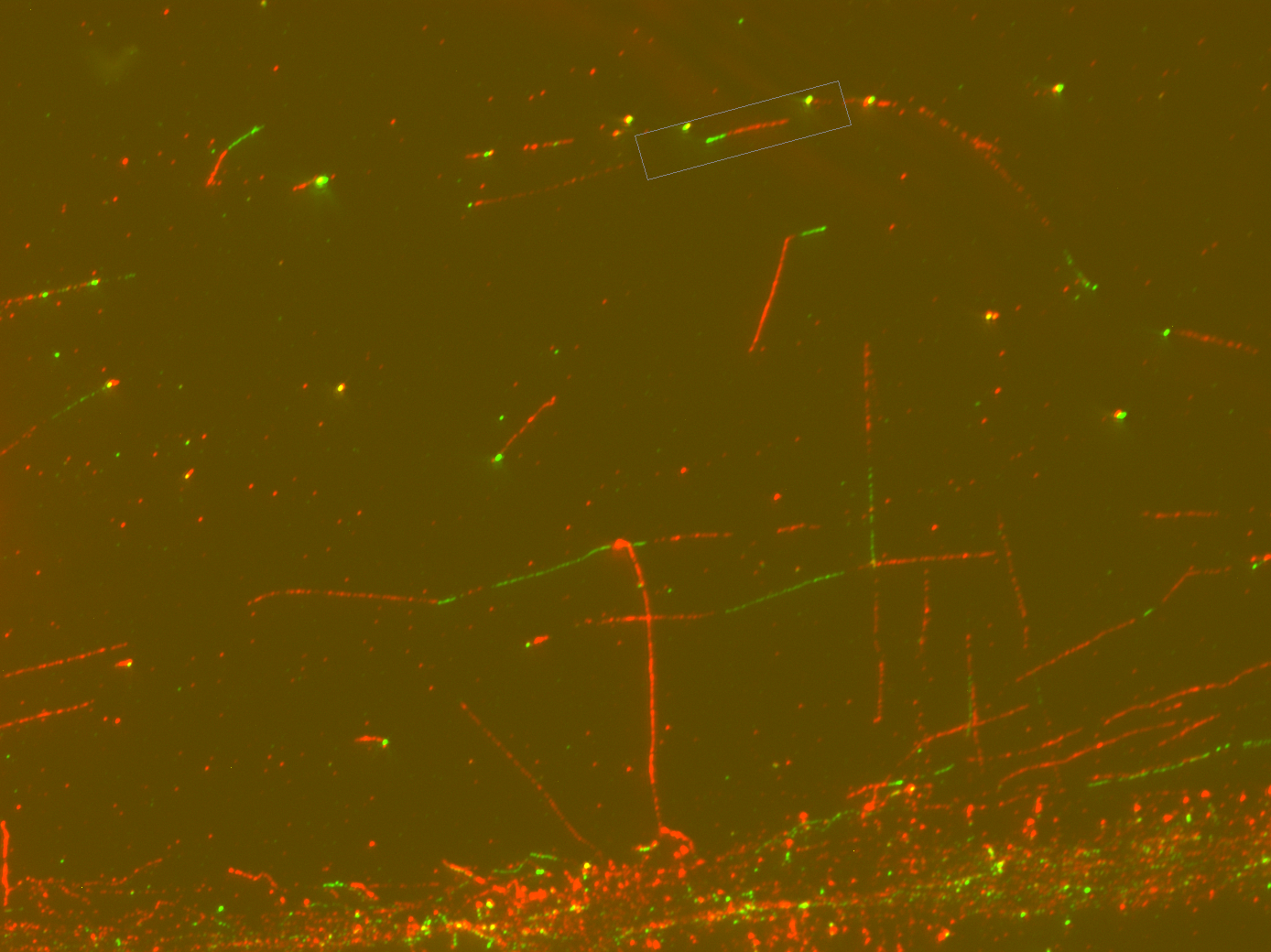

Supplement: Supplementary file 8 — Source data Fig. 5 [file 44319_2025_497_MOESM8_ESM.zip › 5E and 5F/shRNF20 + EV DNA fiber.tif]

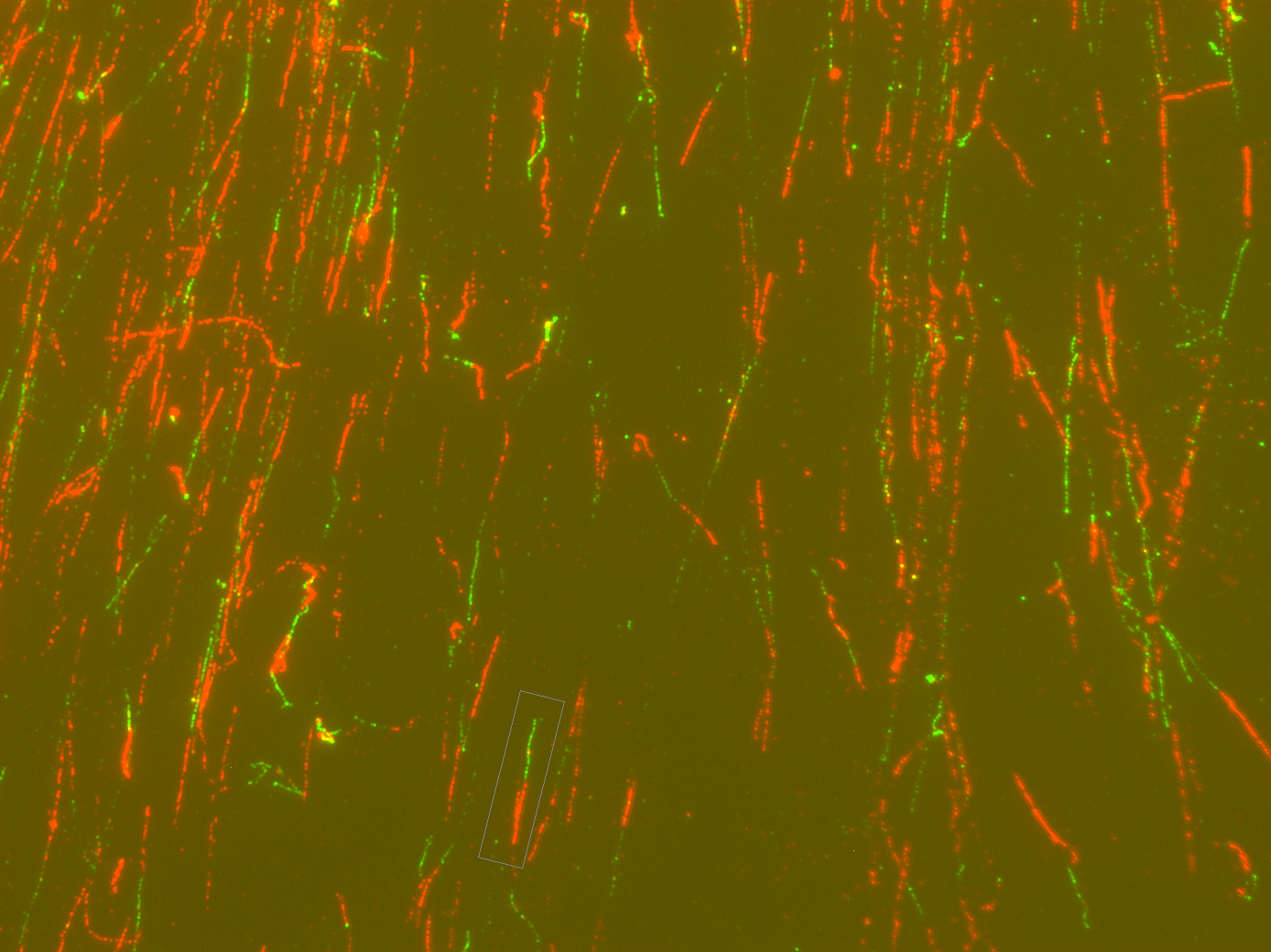

Supplement: Supplementary file 8 — Source data Fig. 5 [file 44319_2025_497_MOESM8_ESM.zip › 5E and 5F/shRNF20 + WT RNF20 DNA fiber.tif]

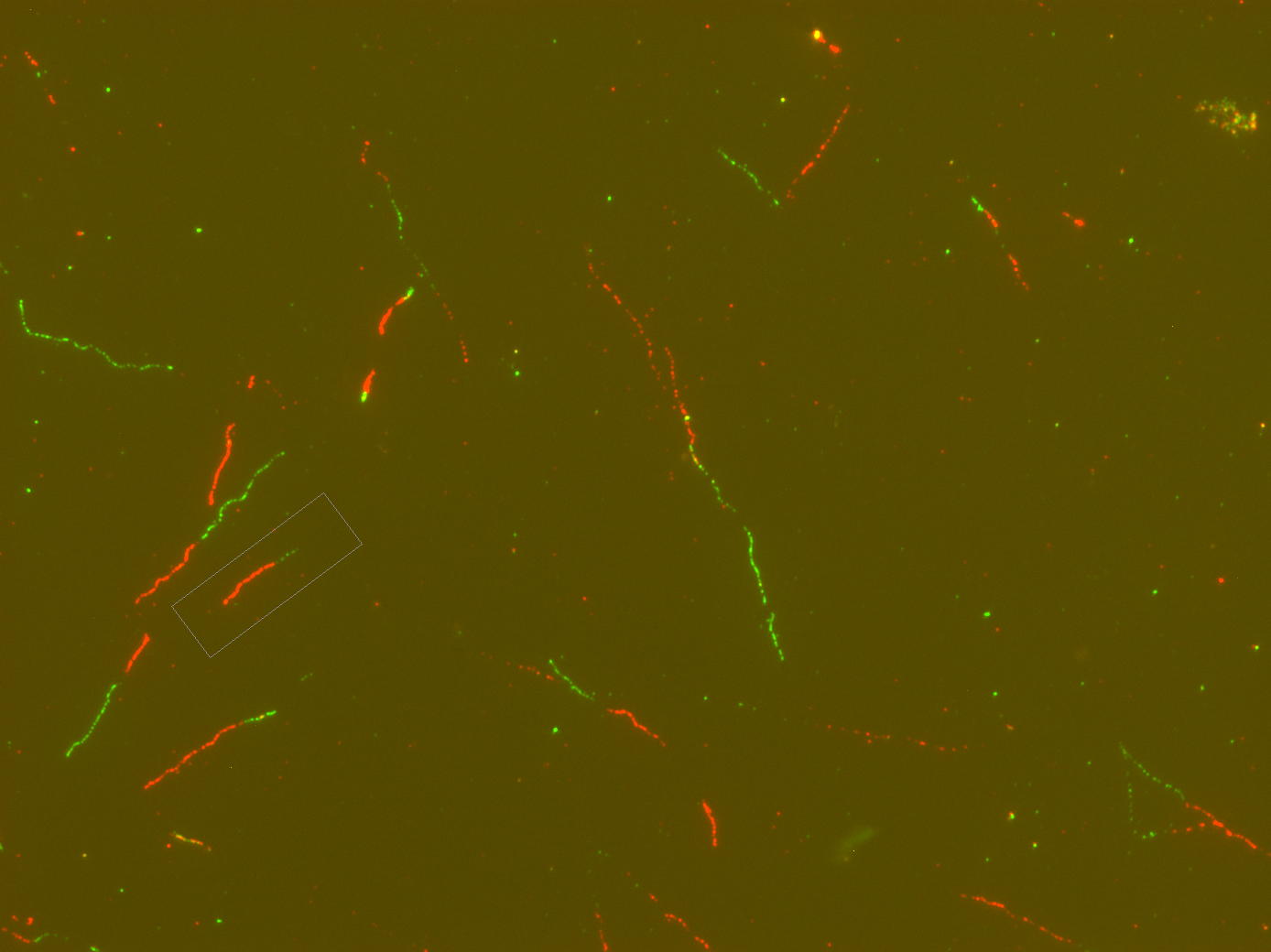

Supplement: Supplementary file 8 — Source data Fig. 5 [file 44319_2025_497_MOESM8_ESM.zip › 5E and 5F/shRNF20+ C922S RNF20 DNA fiber.tif]

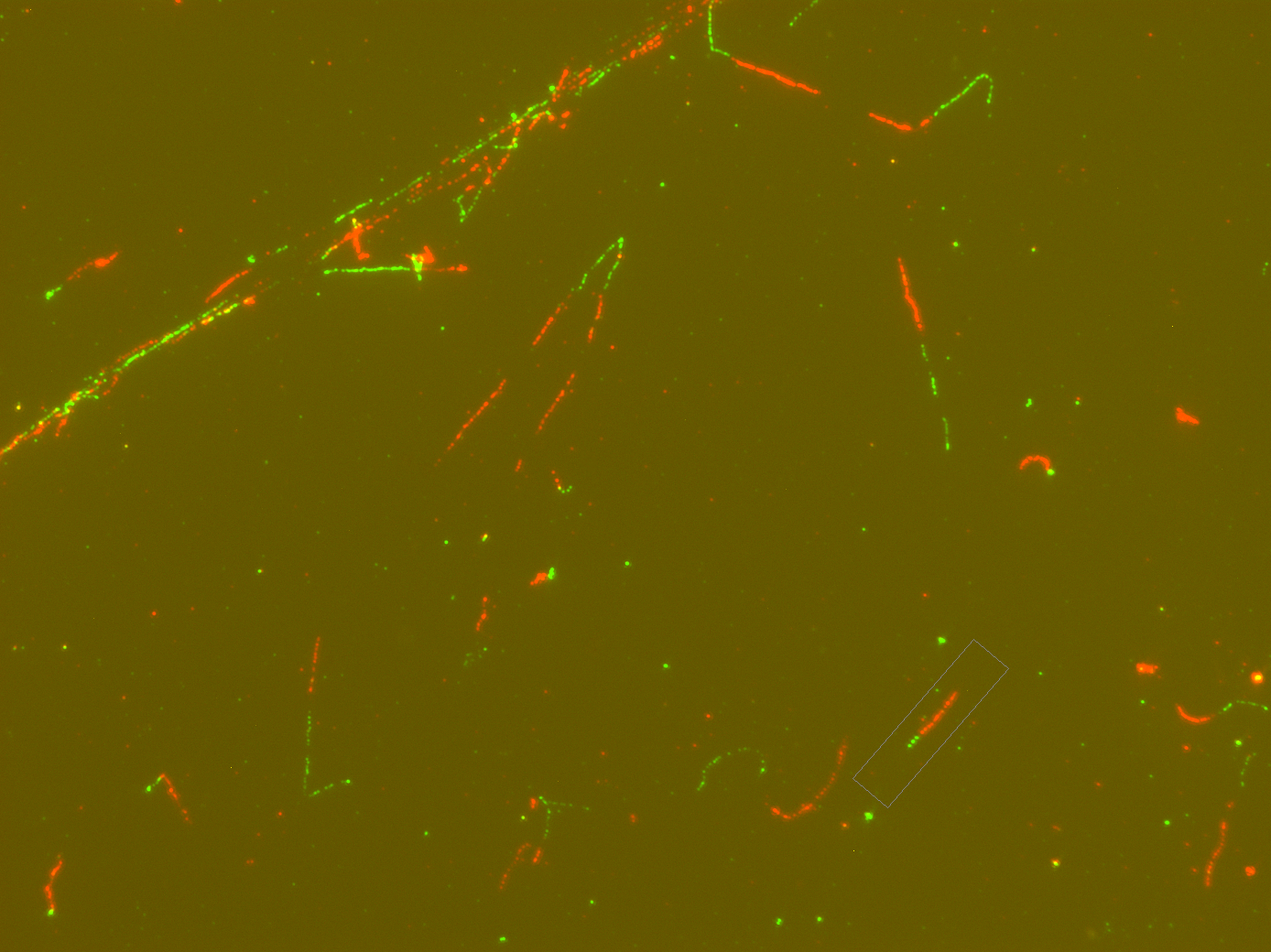

Supplement: Supplementary file 8 — Source data Fig. 5 [file 44319_2025_497_MOESM8_ESM.zip › 5E and 5F/shRNF20+ C960A RNF20 DNA fiber.tif]

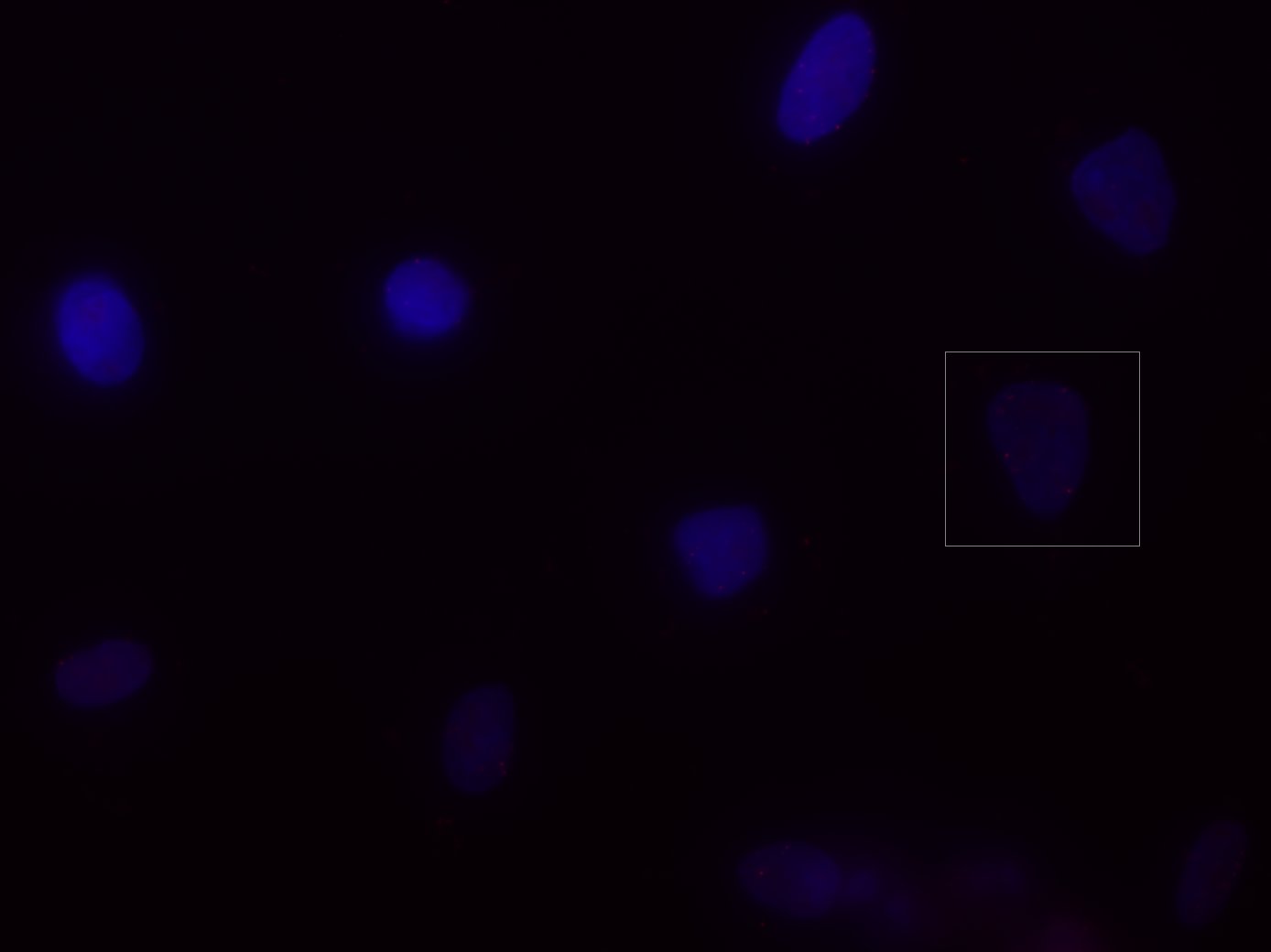

Supplement: Supplementary file 8 — Source data Fig. 5 [file 44319_2025_497_MOESM8_ESM.zip › 5G and 5H/Only EdU H3K27ac SIRF.jpg]

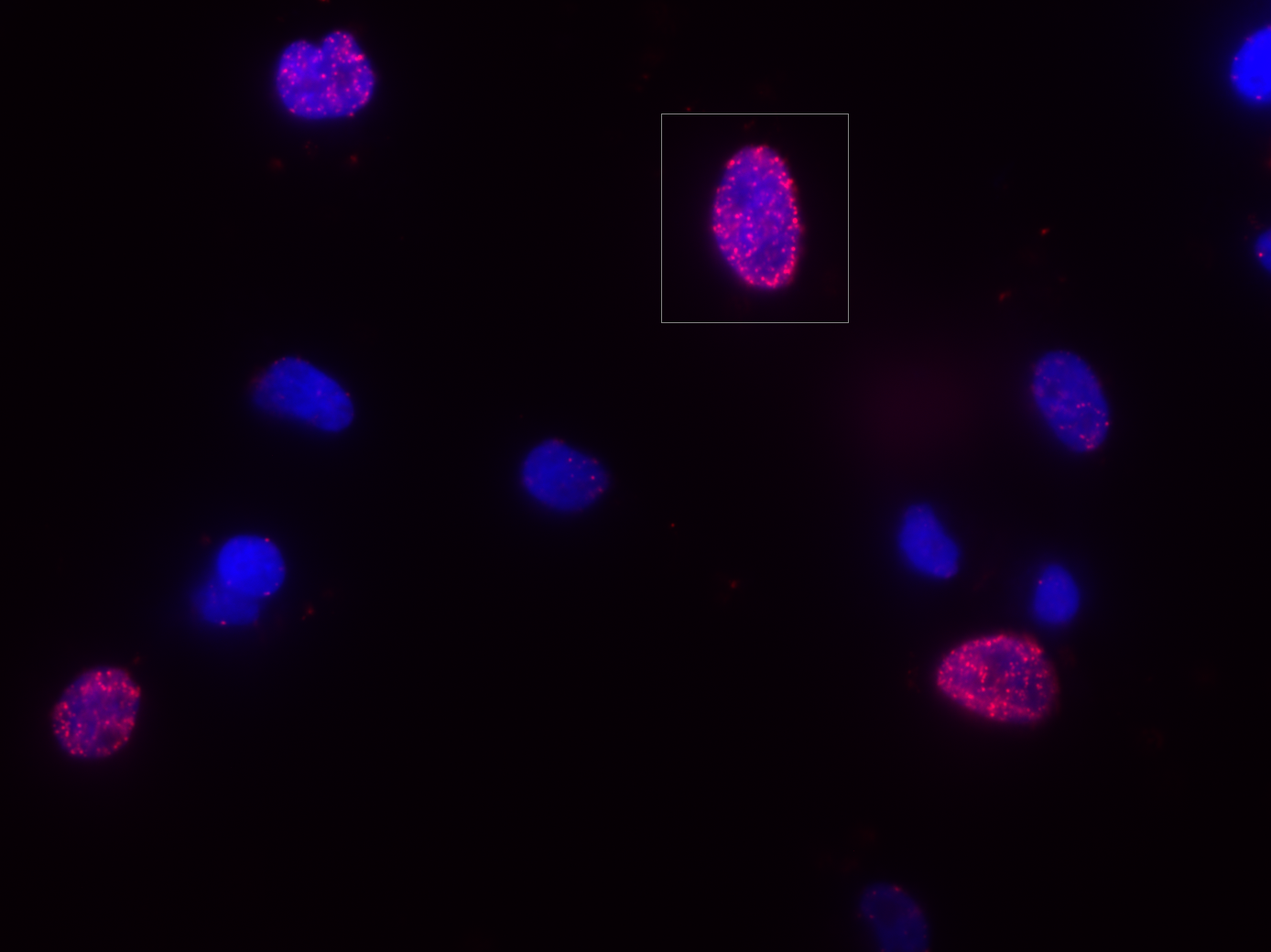

Supplement: Supplementary file 8 — Source data Fig. 5 [file 44319_2025_497_MOESM8_ESM.zip › 5G and 5H/shControl H3K27ac SIRF.tif]

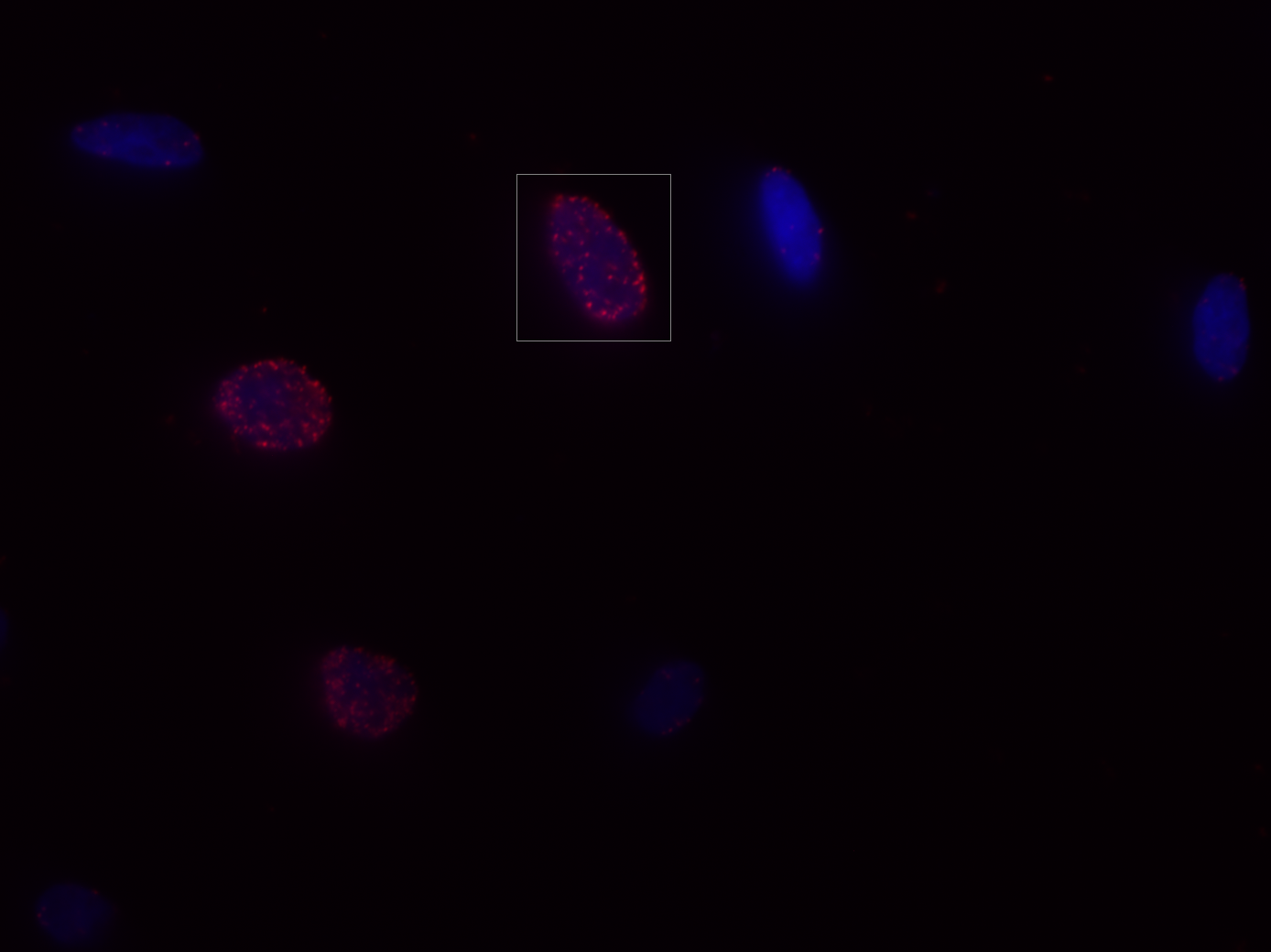

Supplement: Supplementary file 8 — Source data Fig. 5 [file 44319_2025_497_MOESM8_ESM.zip › 5G and 5H/shRNF20 H3K27ac SIRF.tif]

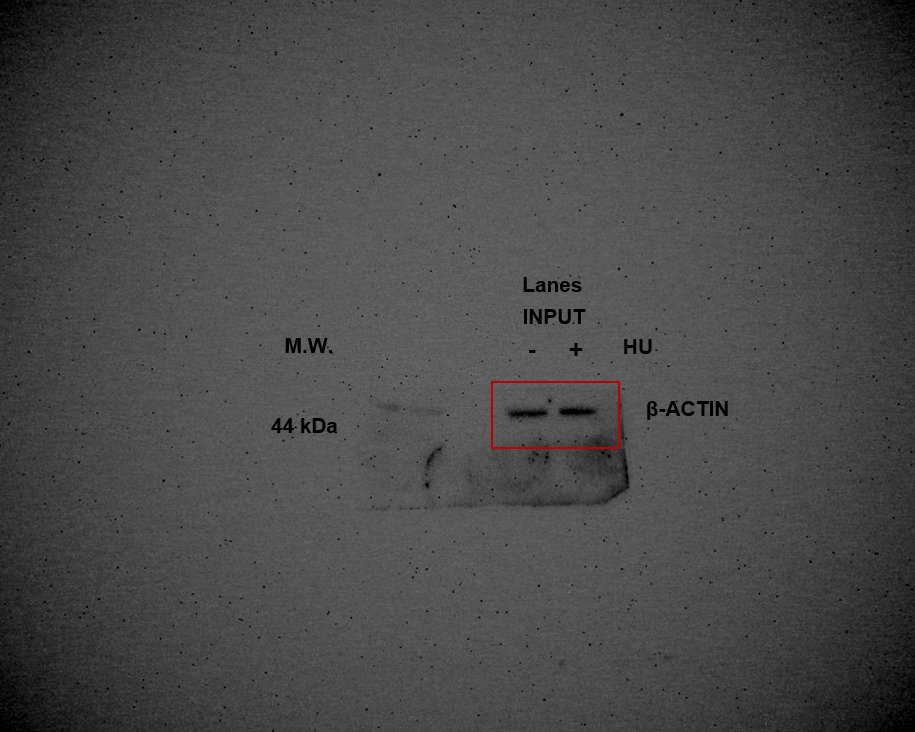

Supplement: Supplementary file 9 — Source data Fig. 6 [file 44319_2025_497_MOESM9_ESM.zip › 6A/ACTIN INPUT western.tif]

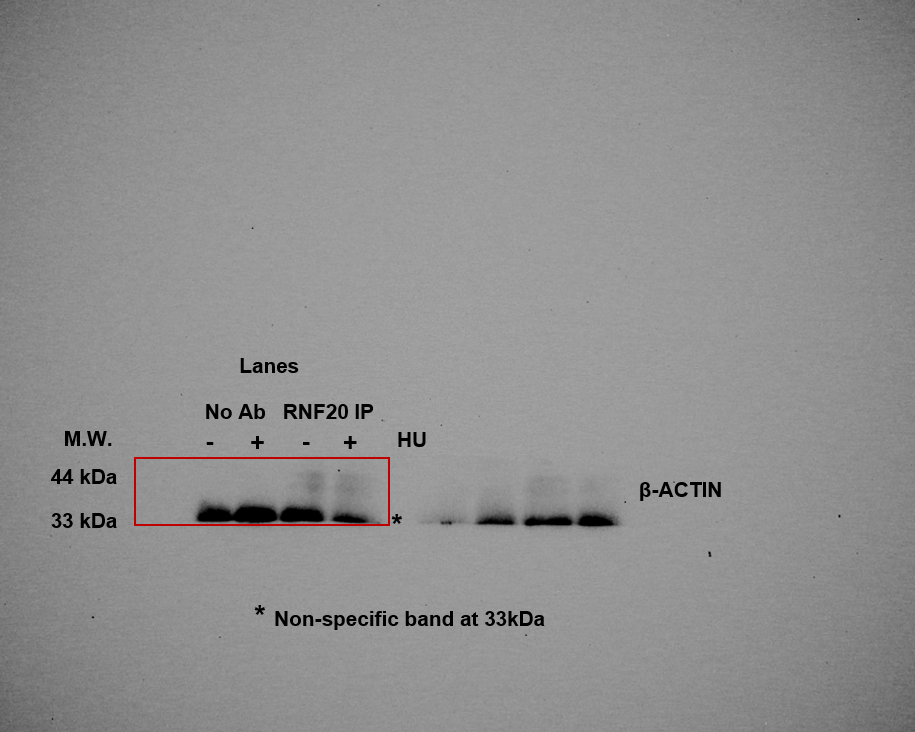

Supplement: Supplementary file 9 — Source data Fig. 6 [file 44319_2025_497_MOESM9_ESM.zip › 6A/ACTIN IP western.tif]

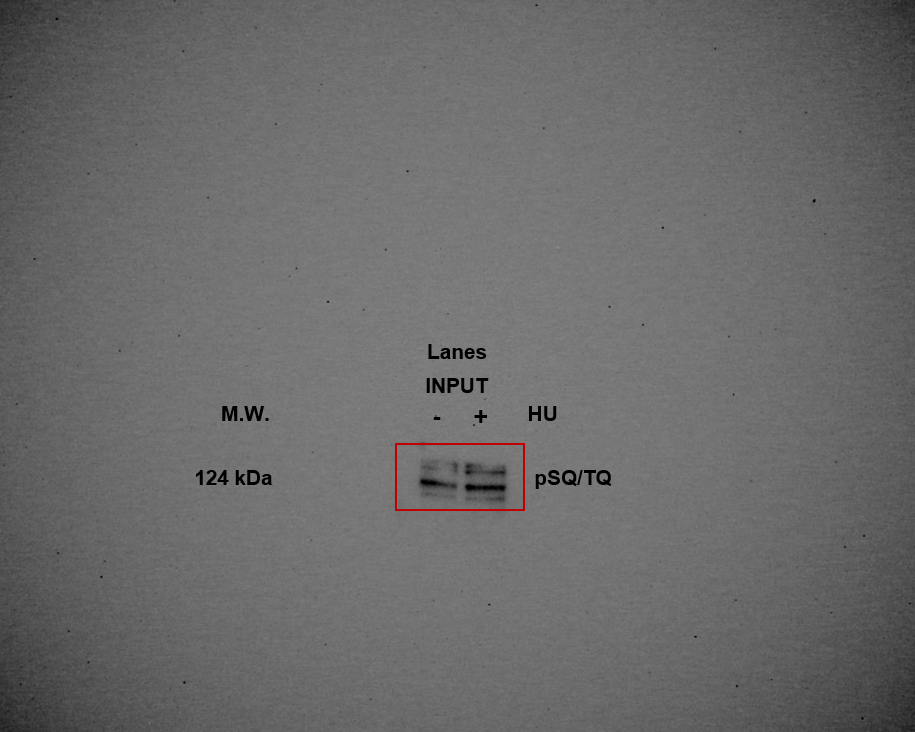

Supplement: Supplementary file 9 — Source data Fig. 6 [file 44319_2025_497_MOESM9_ESM.zip › 6A/pSQTQ INPUT western.tif]

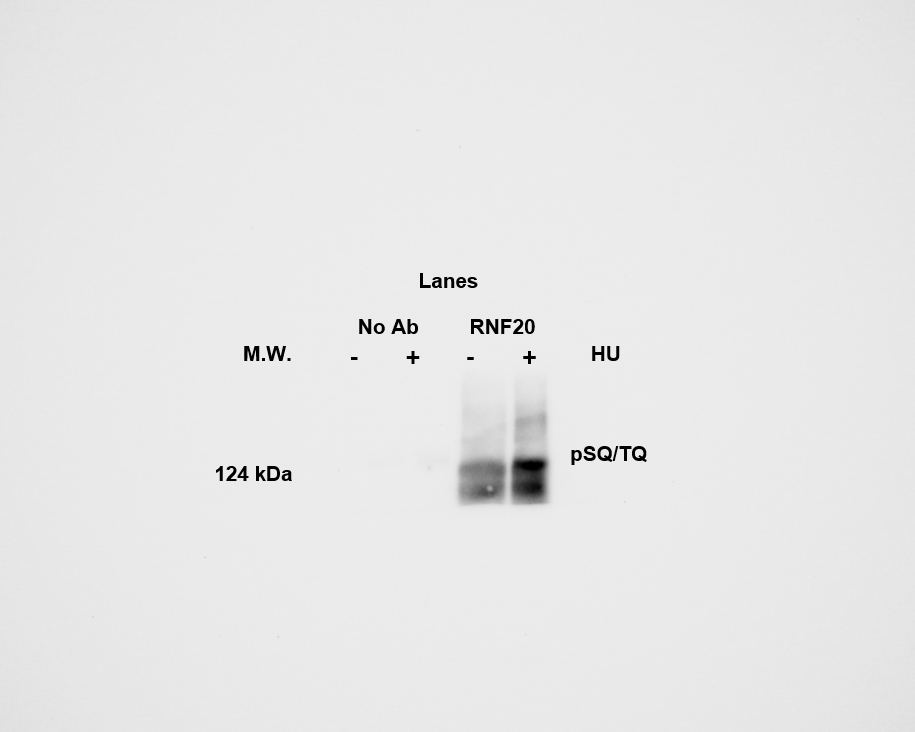

Supplement: Supplementary file 9 — Source data Fig. 6 [file 44319_2025_497_MOESM9_ESM.zip › 6A/pSQTQ IP western.tif]

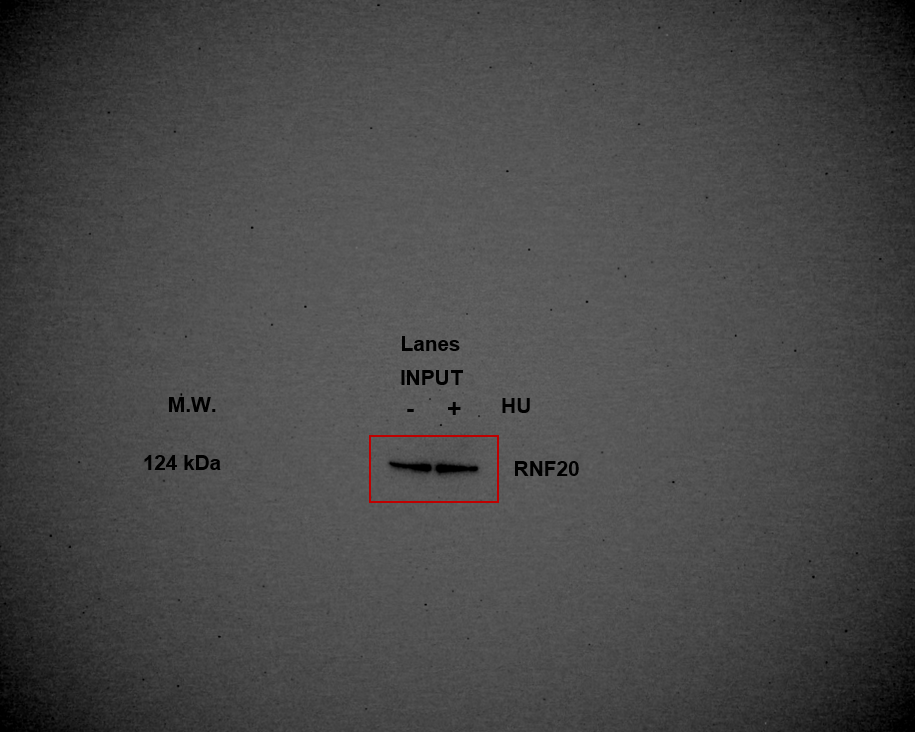

Supplement: Supplementary file 9 — Source data Fig. 6 [file 44319_2025_497_MOESM9_ESM.zip › 6A/RNF20 INPUT western.tif]

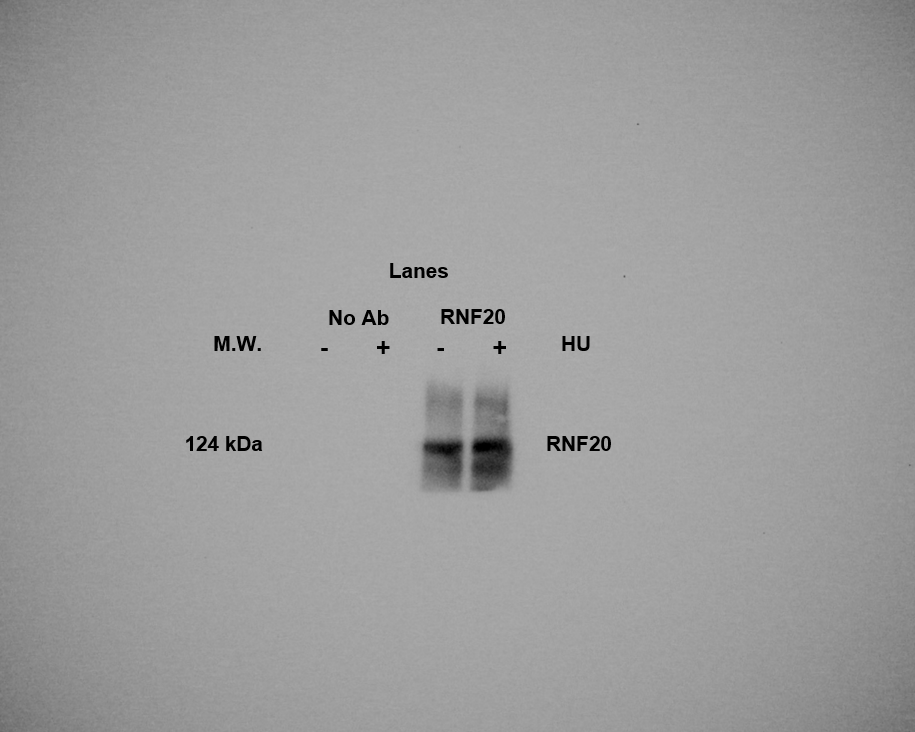

Supplement: Supplementary file 9 — Source data Fig. 6 [file 44319_2025_497_MOESM9_ESM.zip › 6A/RNF20 IP western.tif]

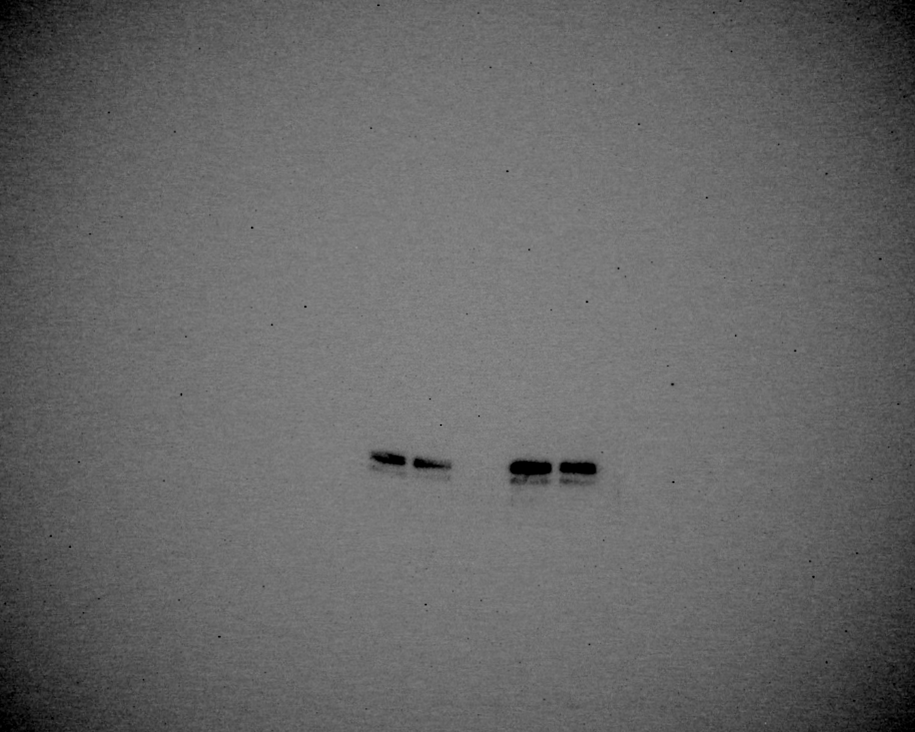

Supplement: Supplementary file 9 — Source data Fig. 6 [file 44319_2025_497_MOESM9_ESM.zip › 6A/RPA70 INPUT western.tif]

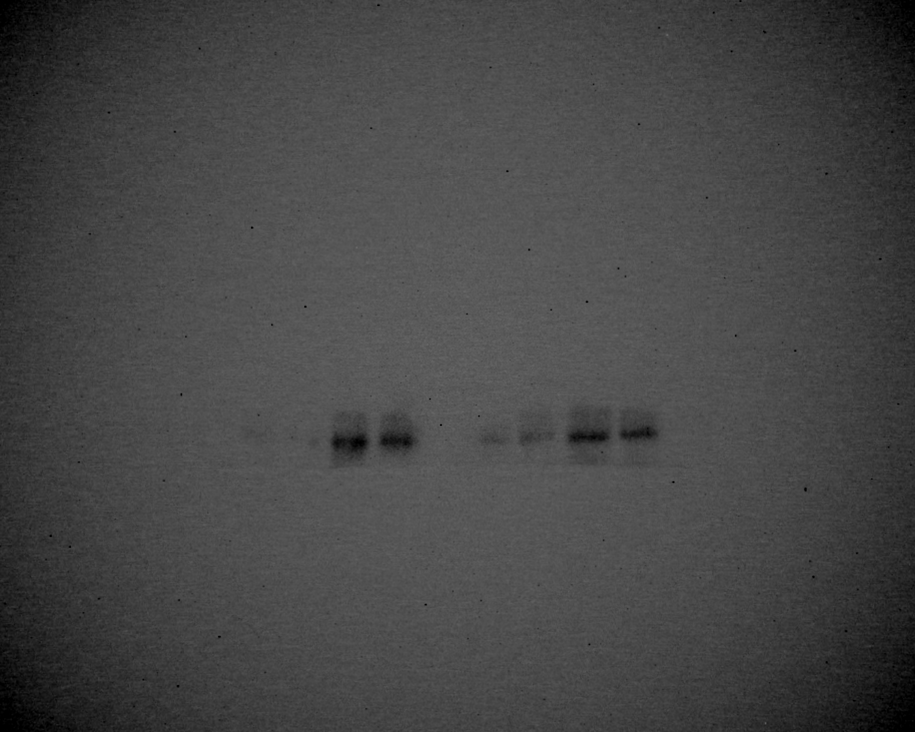

Supplement: Supplementary file 9 — Source data Fig. 6 [file 44319_2025_497_MOESM9_ESM.zip › 6A/RPA70 IP western.tif]

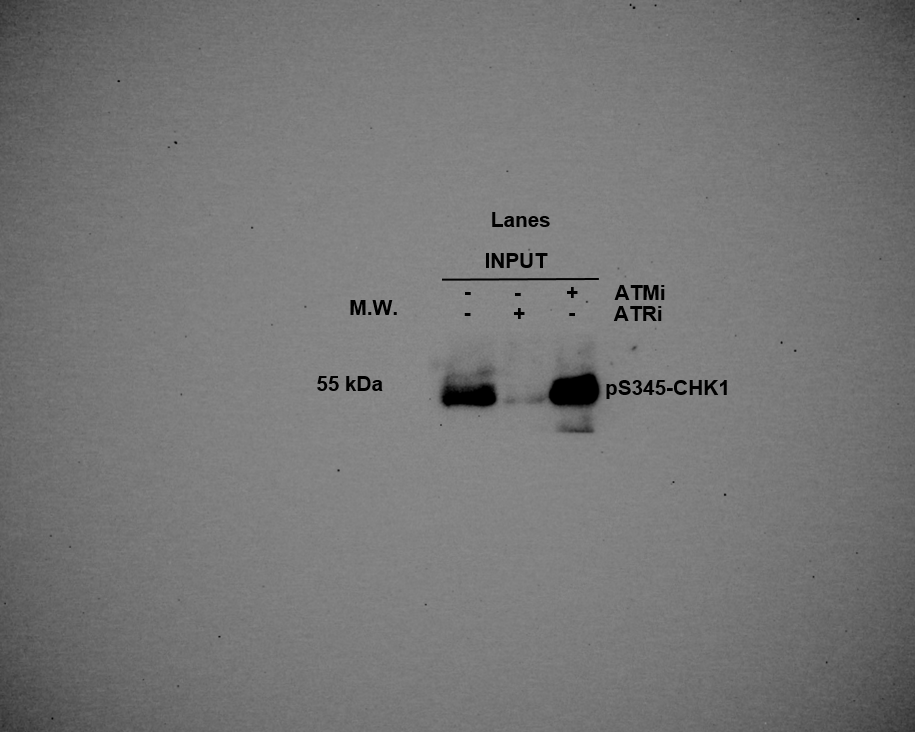

Supplement: Supplementary file 9 — Source data Fig. 6 [file 44319_2025_497_MOESM9_ESM.zip › 6B/pS345-CHK1 INPUT western.tif]

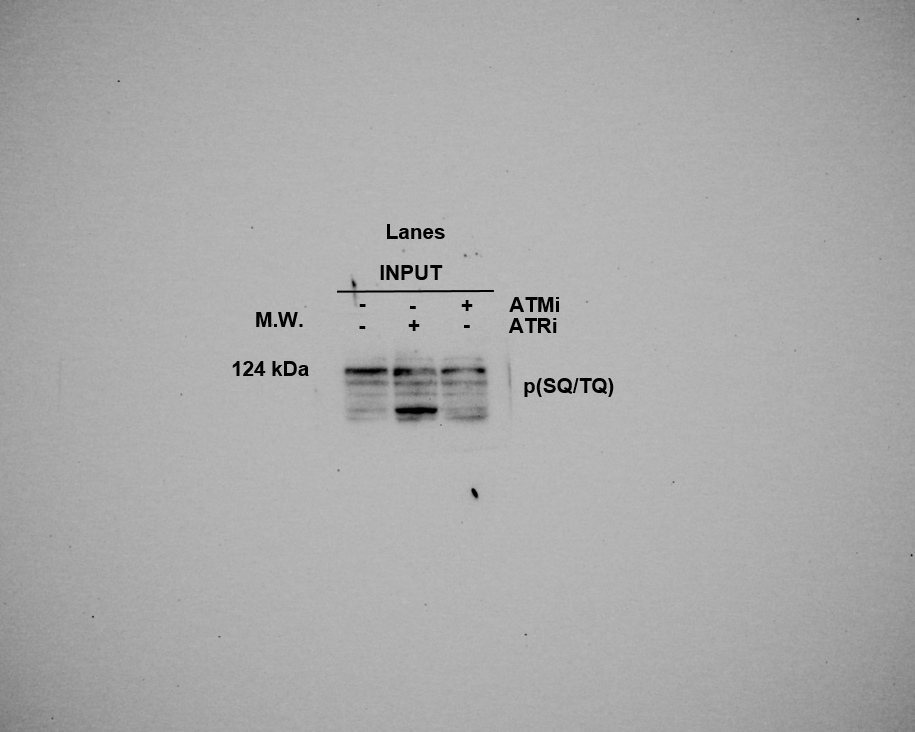

Supplement: Supplementary file 9 — Source data Fig. 6 [file 44319_2025_497_MOESM9_ESM.zip › 6B/pSQ-TQ IP INPUT western.tif]

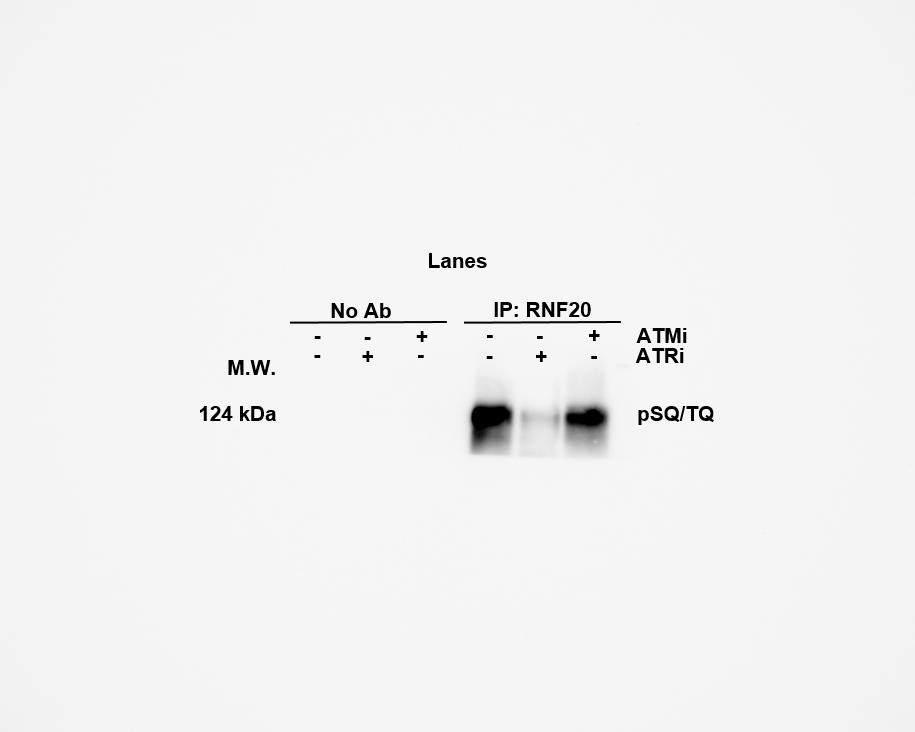

Supplement: Supplementary file 9 — Source data Fig. 6 [file 44319_2025_497_MOESM9_ESM.zip › 6B/pSQ-TQ IP western.tif]

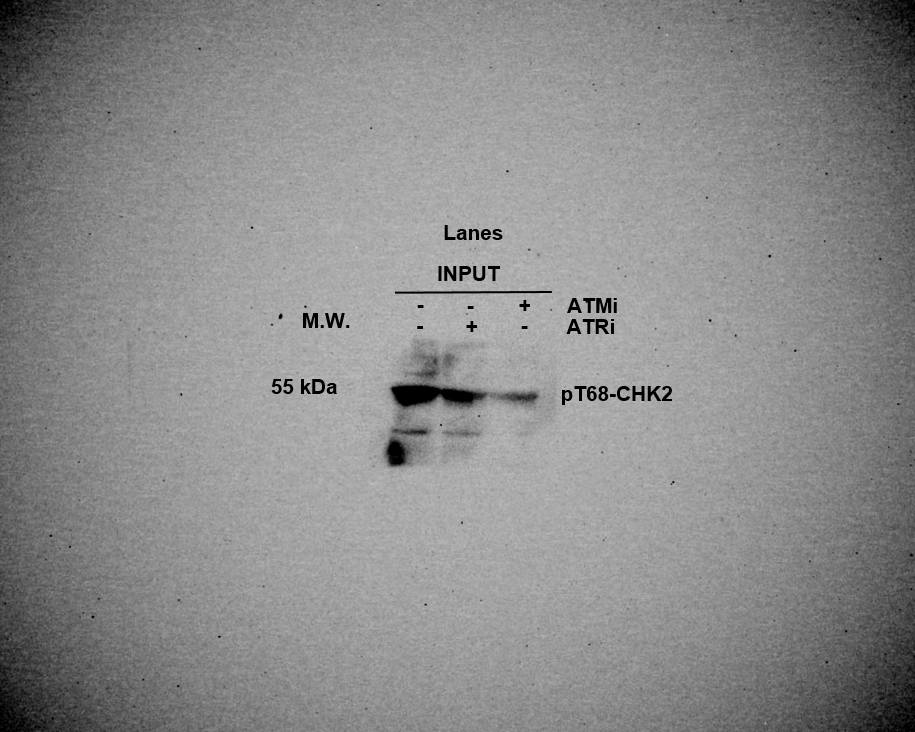

Supplement: Supplementary file 9 — Source data Fig. 6 [file 44319_2025_497_MOESM9_ESM.zip › 6B/pT68-CHK2 INPUT western.tif]

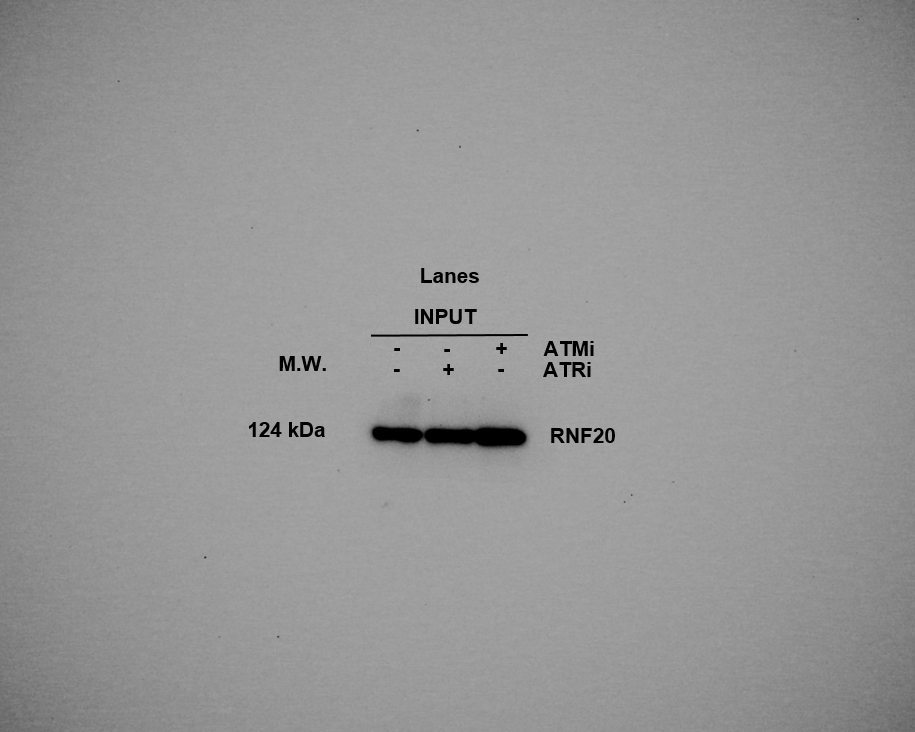

Supplement: Supplementary file 9 — Source data Fig. 6 [file 44319_2025_497_MOESM9_ESM.zip › 6B/RNF20 INPUT western.tif]

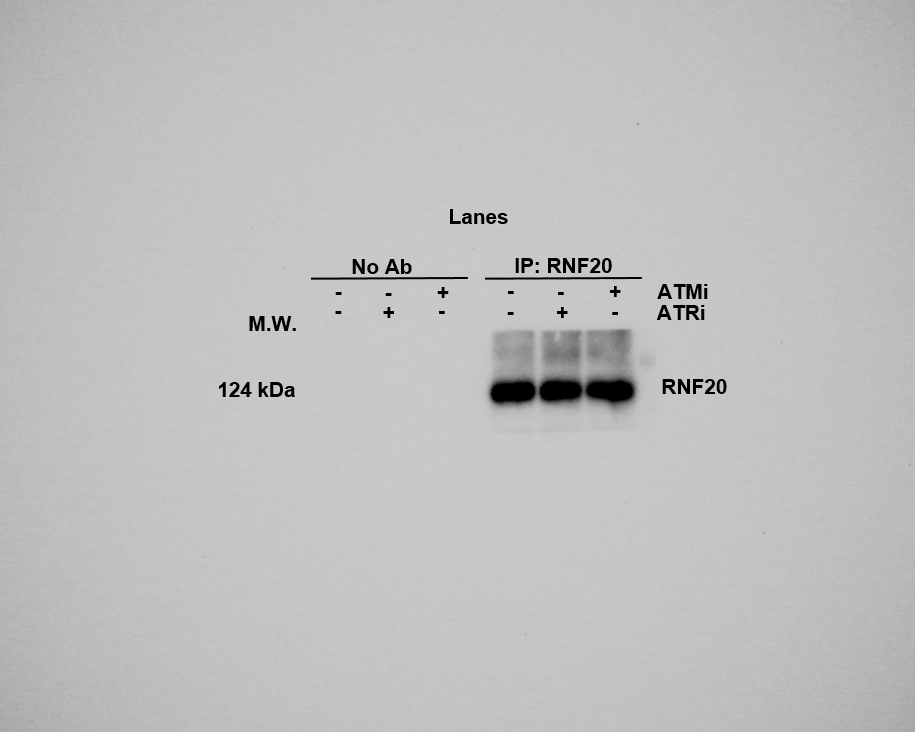

Supplement: Supplementary file 9 — Source data Fig. 6 [file 44319_2025_497_MOESM9_ESM.zip › 6B/RNF20 IP western.tif]

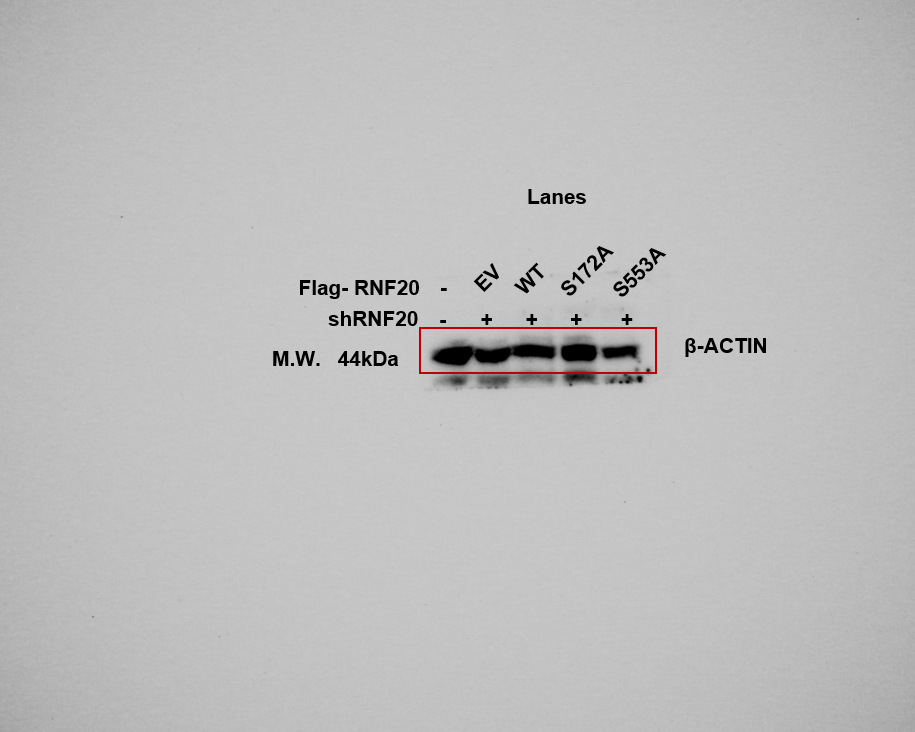

Supplement: Supplementary file 9 — Source data Fig. 6 [file 44319_2025_497_MOESM9_ESM.zip › 6C/betaACTIN western.tif]

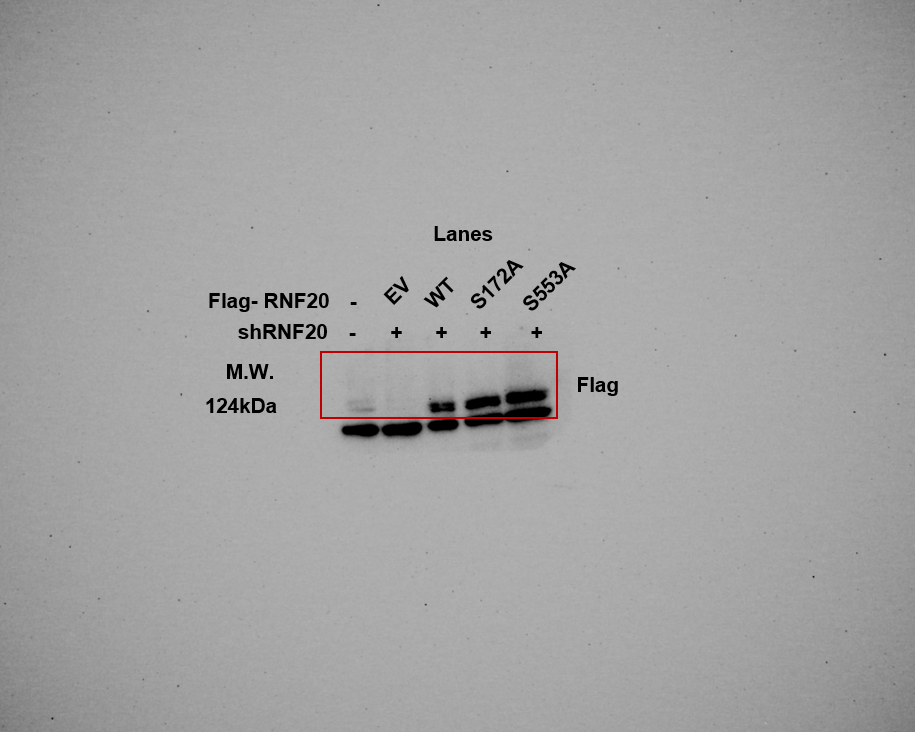

Supplement: Supplementary file 9 — Source data Fig. 6 [file 44319_2025_497_MOESM9_ESM.zip › 6C/Flag western.tif]

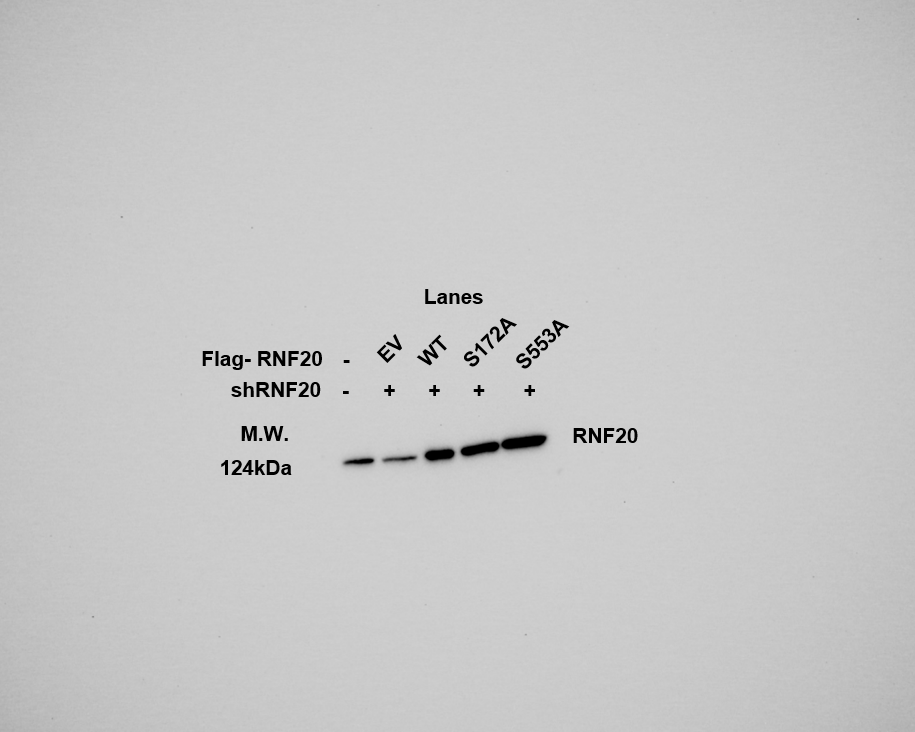

Supplement: Supplementary file 9 — Source data Fig. 6 [file 44319_2025_497_MOESM9_ESM.zip › 6C/RNF20 western.tif]

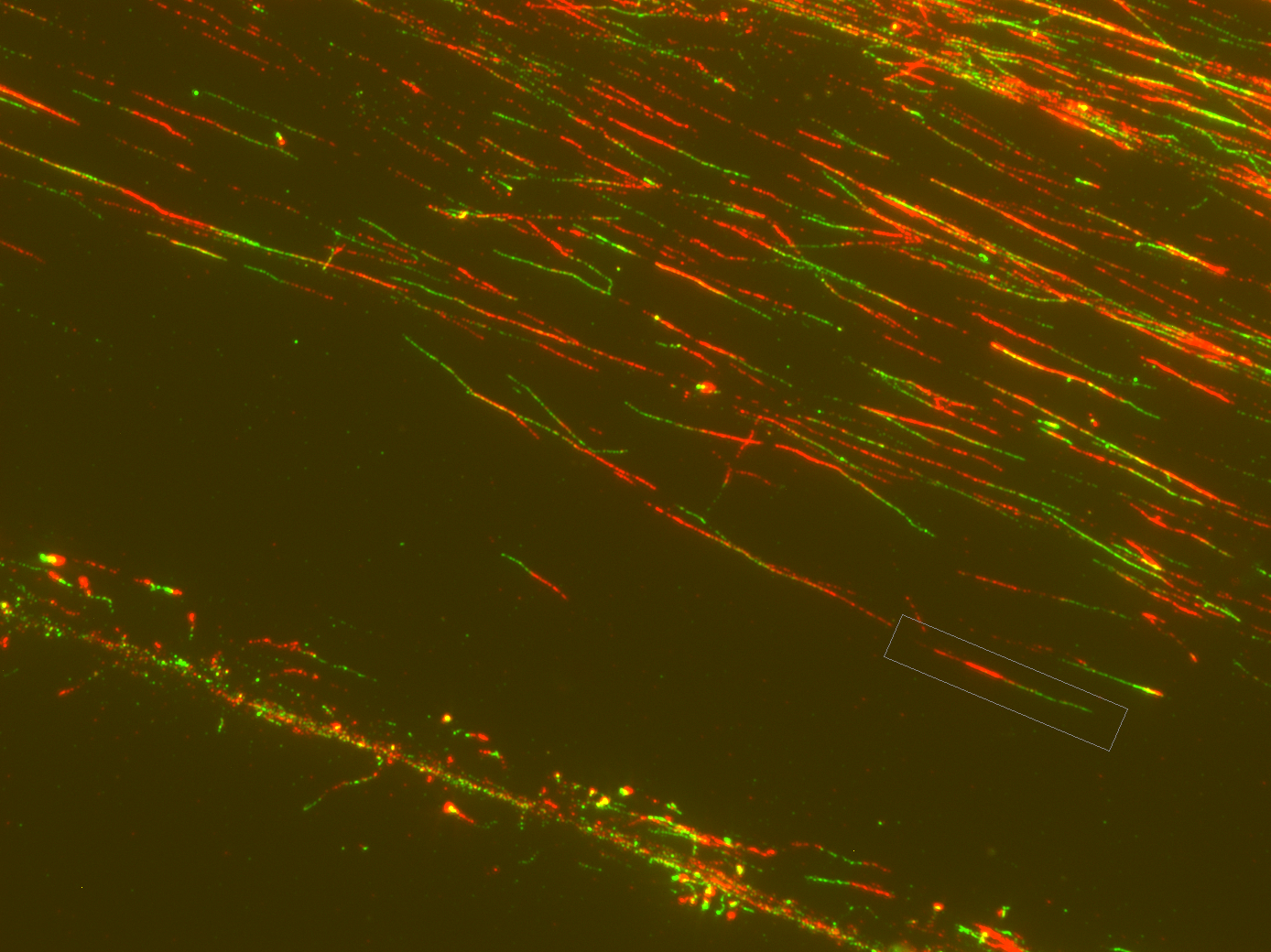

Supplement: Supplementary file 9 — Source data Fig. 6 [file 44319_2025_497_MOESM9_ESM.zip › 6D and 6E/shControl DNA fiber.tif]

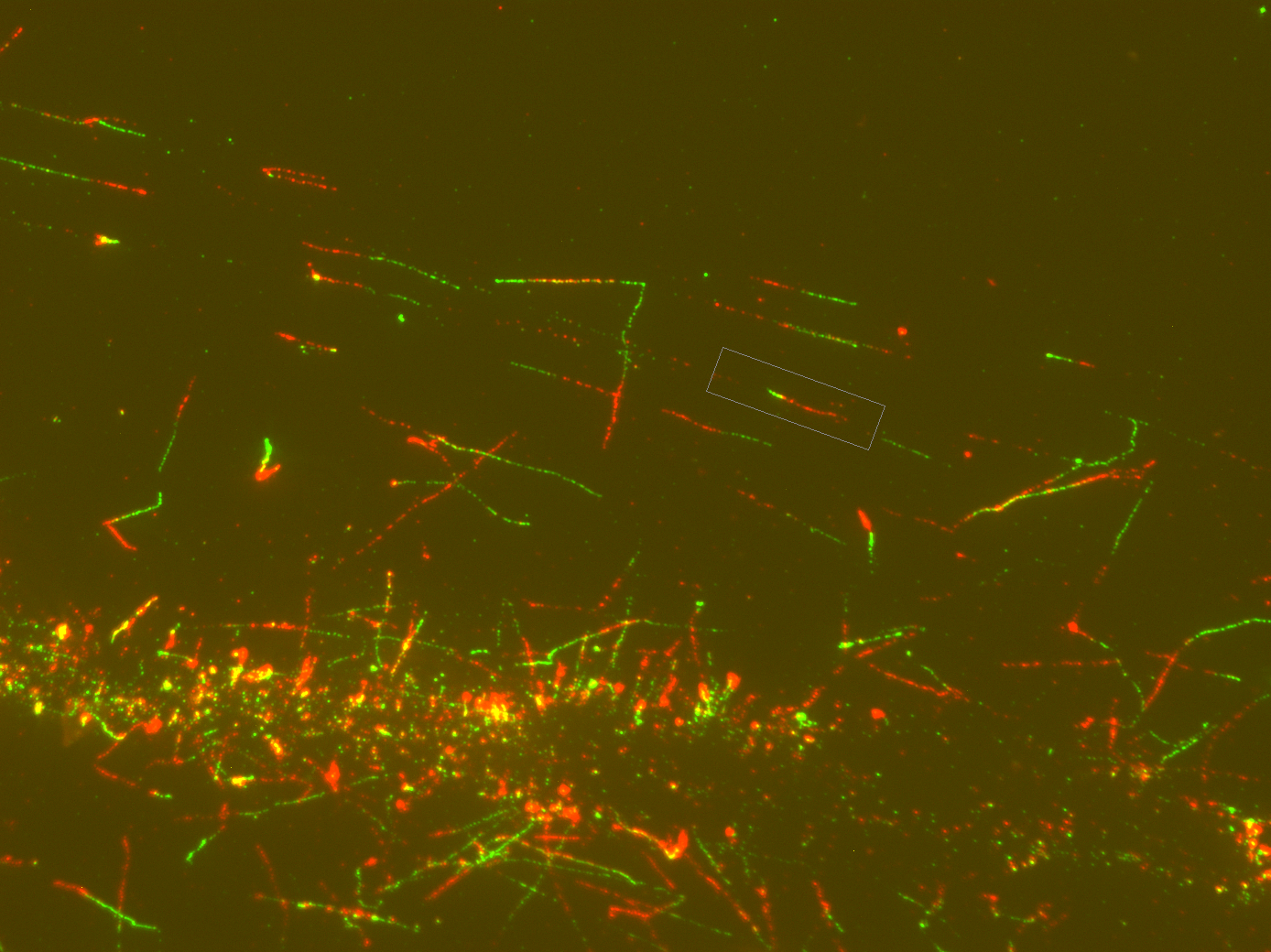

Supplement: Supplementary file 9 — Source data Fig. 6 [file 44319_2025_497_MOESM9_ESM.zip › 6D and 6E/shRNF20 DNA fiber.tif]

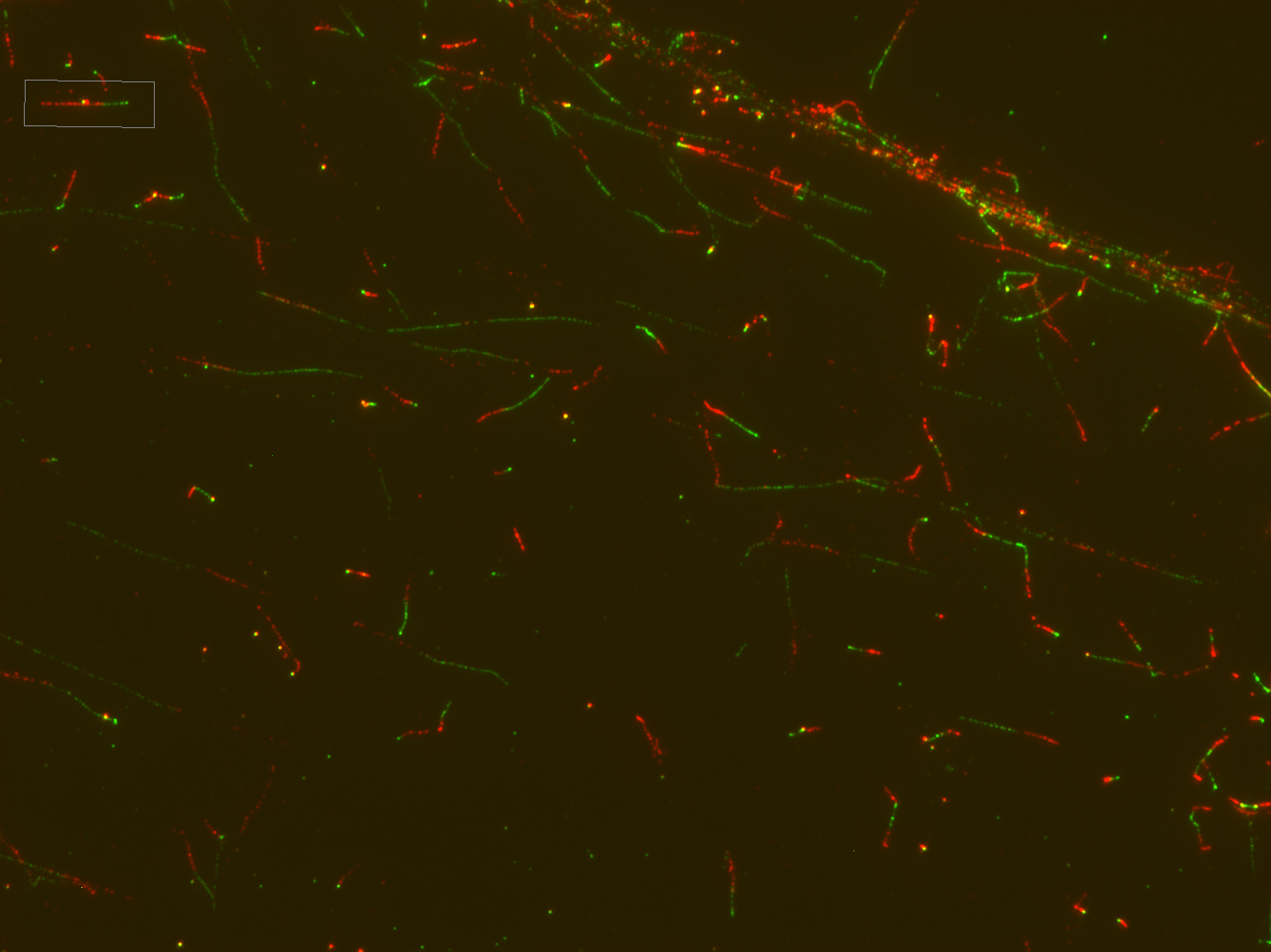

Supplement: Supplementary file 9 — Source data Fig. 6 [file 44319_2025_497_MOESM9_ESM.zip › 6D and 6E/shRNF20+ S172A RNF20.tif]

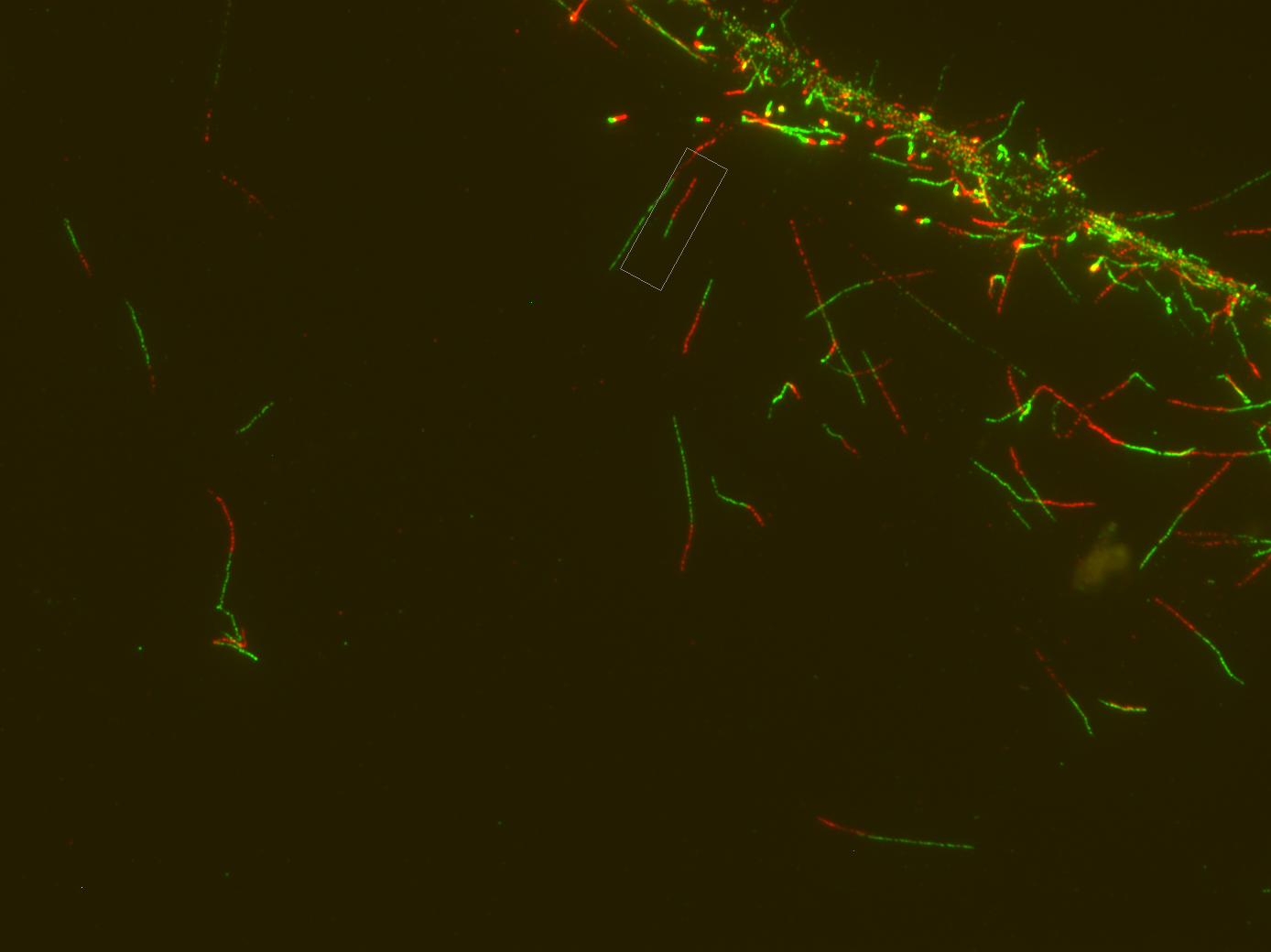

Supplement: Supplementary file 9 — Source data Fig. 6 [file 44319_2025_497_MOESM9_ESM.zip › 6D and 6E/shRNF20+ S553A RNF20.tif]

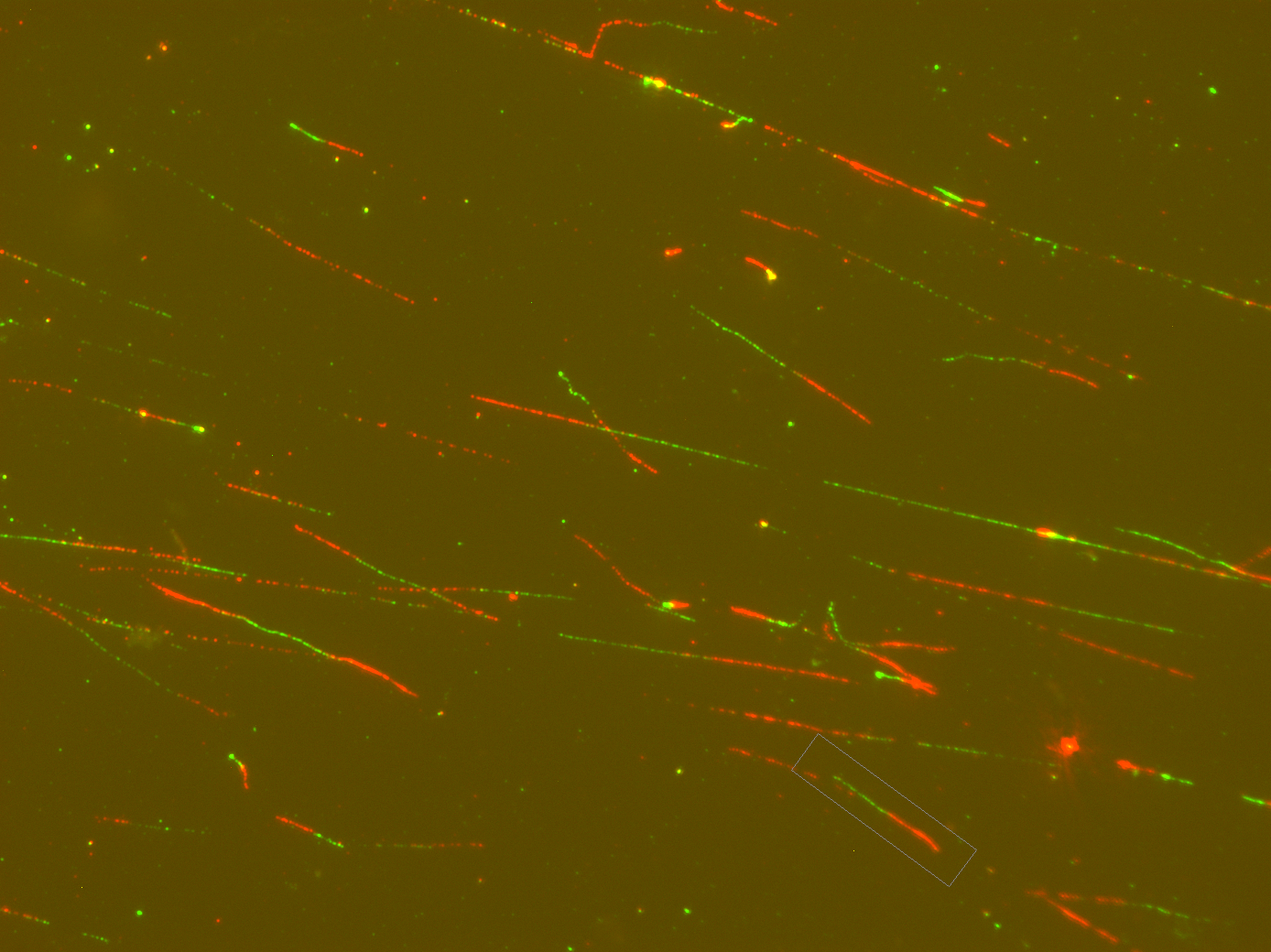

Supplement: Supplementary file 9 — Source data Fig. 6 [file 44319_2025_497_MOESM9_ESM.zip › 6D and 6E/shRNF20+WT RNF20 DNA fiber.tif]

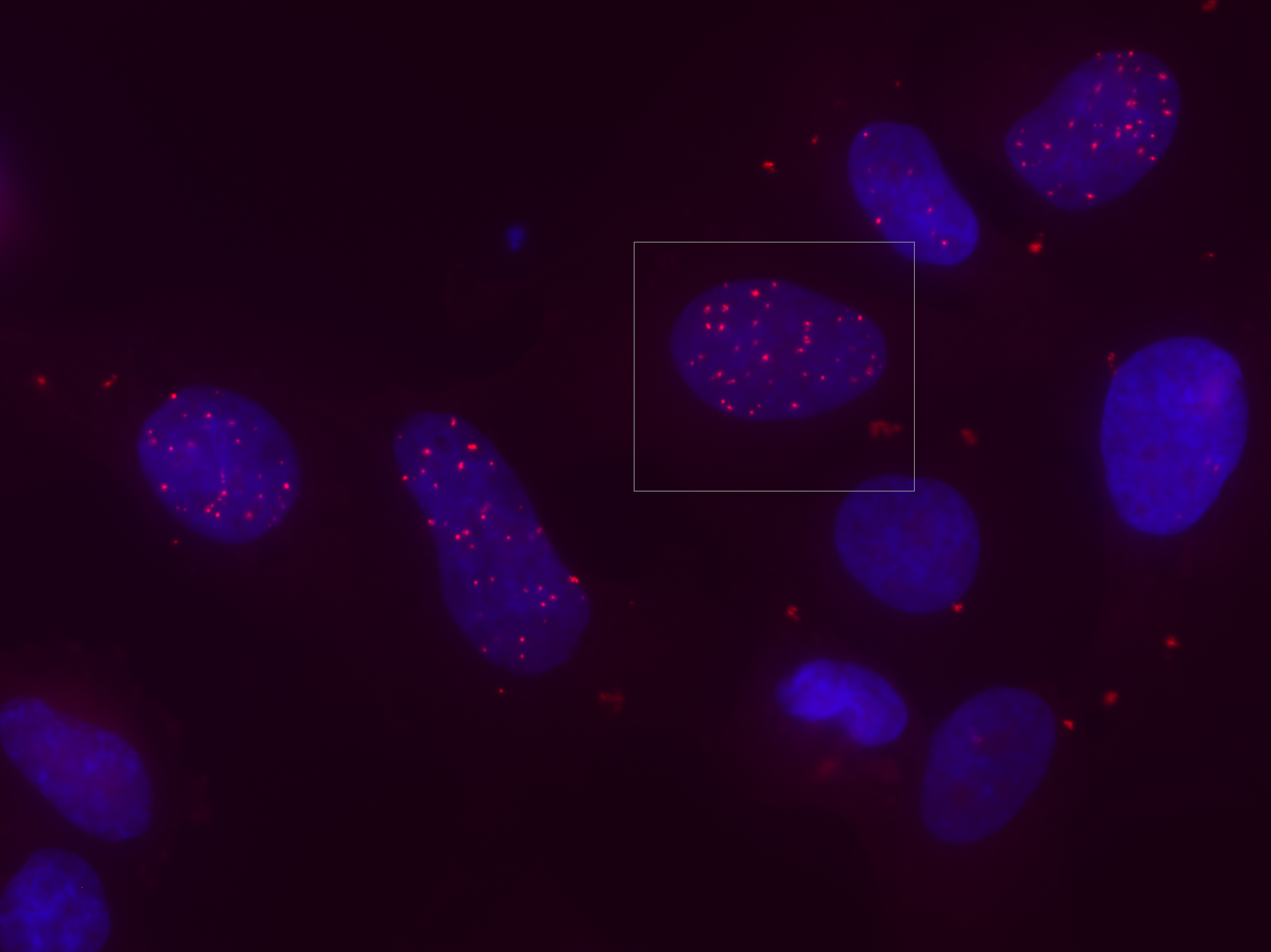

Supplement: Supplementary file 9 — Source data Fig. 6 [file 44319_2025_497_MOESM9_ESM.zip › 6G and 6H/shControl H2BK120ub SIRF.tif]

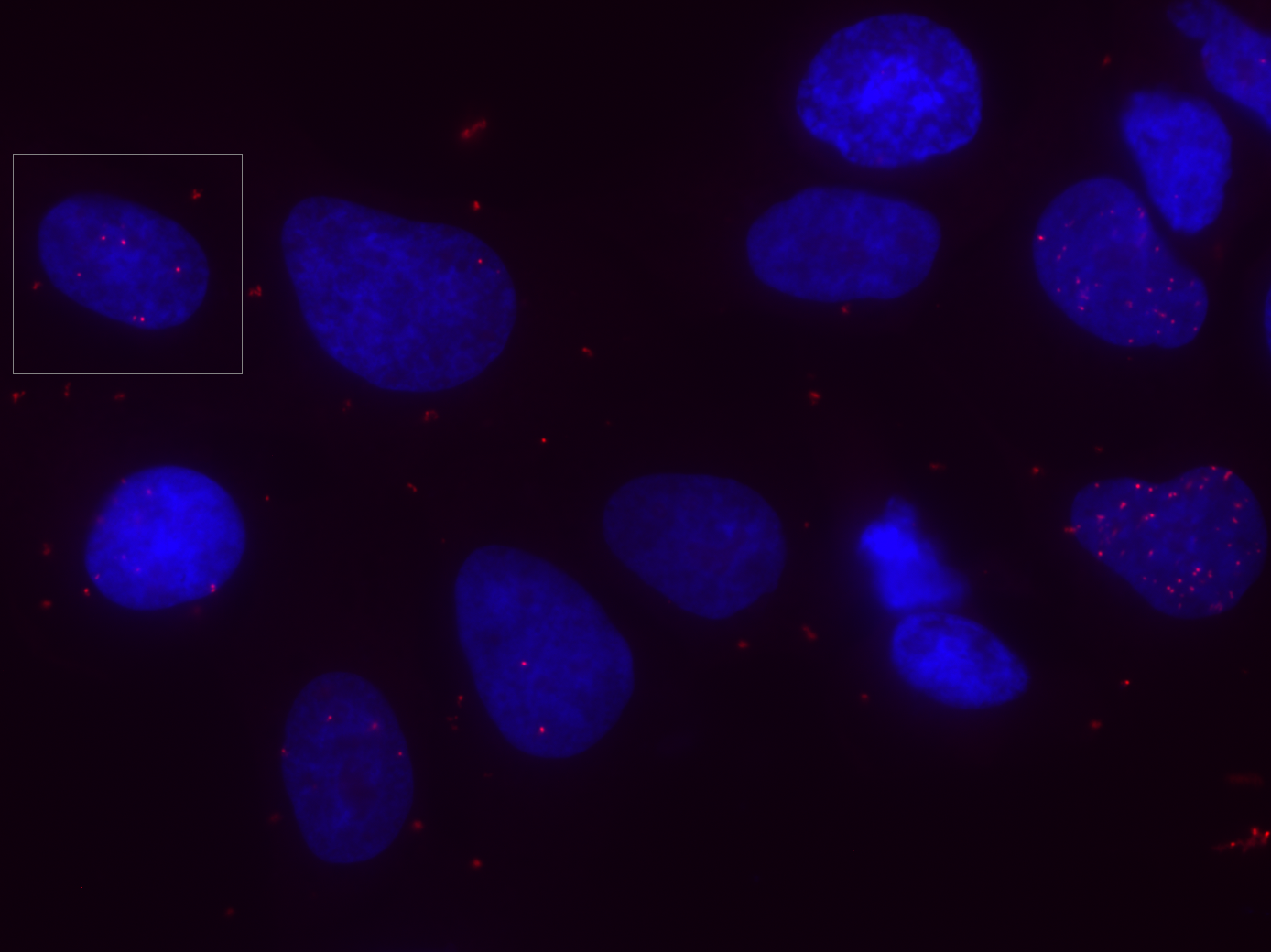

Supplement: Supplementary file 9 — Source data Fig. 6 [file 44319_2025_497_MOESM9_ESM.zip › 6G and 6H/shRNF20+ pcDNA-EV.tif]
