# Supplementary figures and images for: RNF20-mediated H2B monoubiquitination protects stalled forks from degradation and promotes fork restart (part 2 of 3)
Source: EMBO Rep. 2025 Jun 10;26(15):3773–803. doi: 10.1038/s44319-025-00497-3 (PMC12331980; doi:10.1038/s44319-025-00497-3)

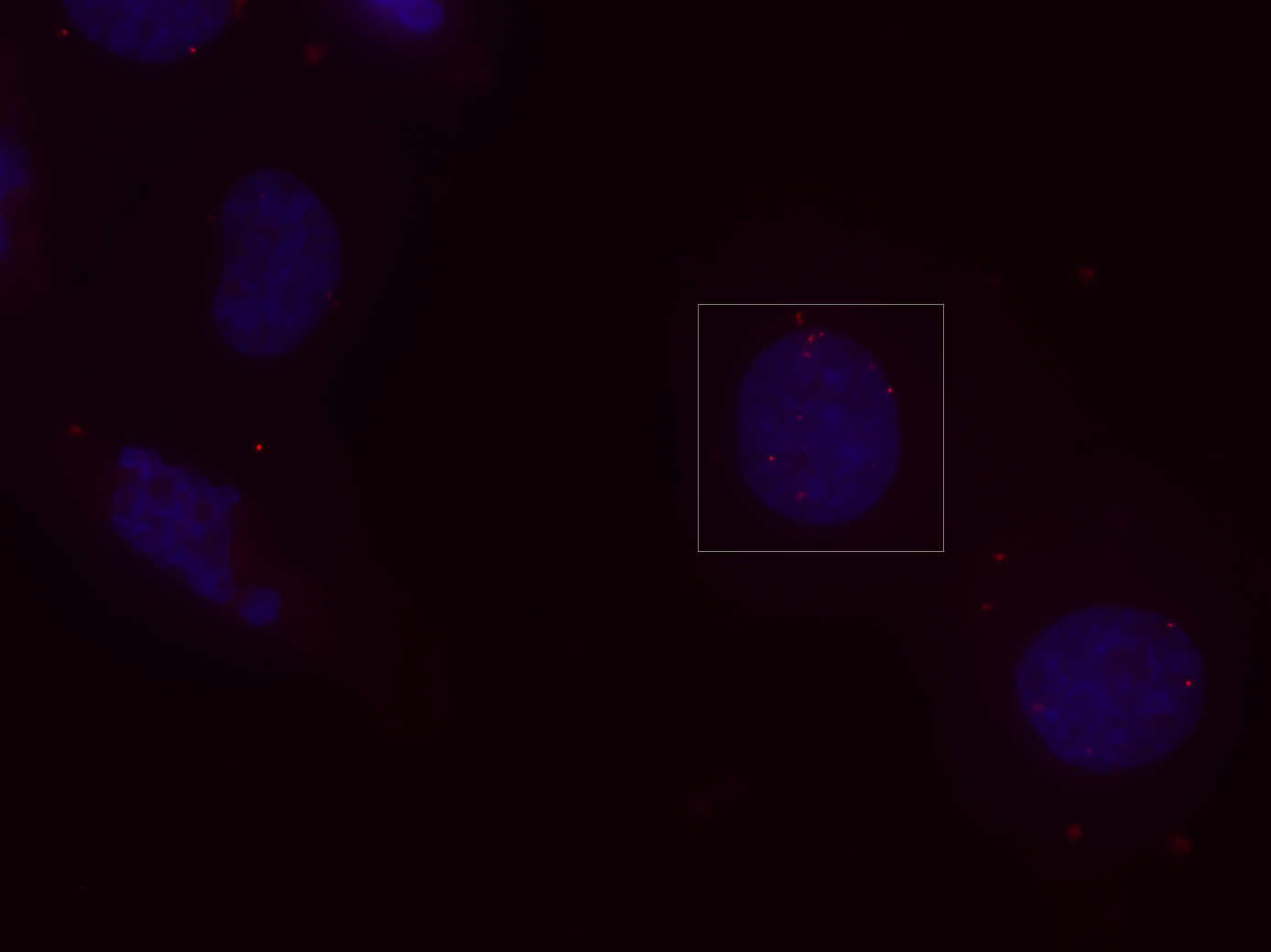

Supplement: Supplementary file 9 — Source data Fig. 6 [file 44319_2025_497_MOESM9_ESM.zip › 6G and 6H/shRNF20+ S172A.tif]

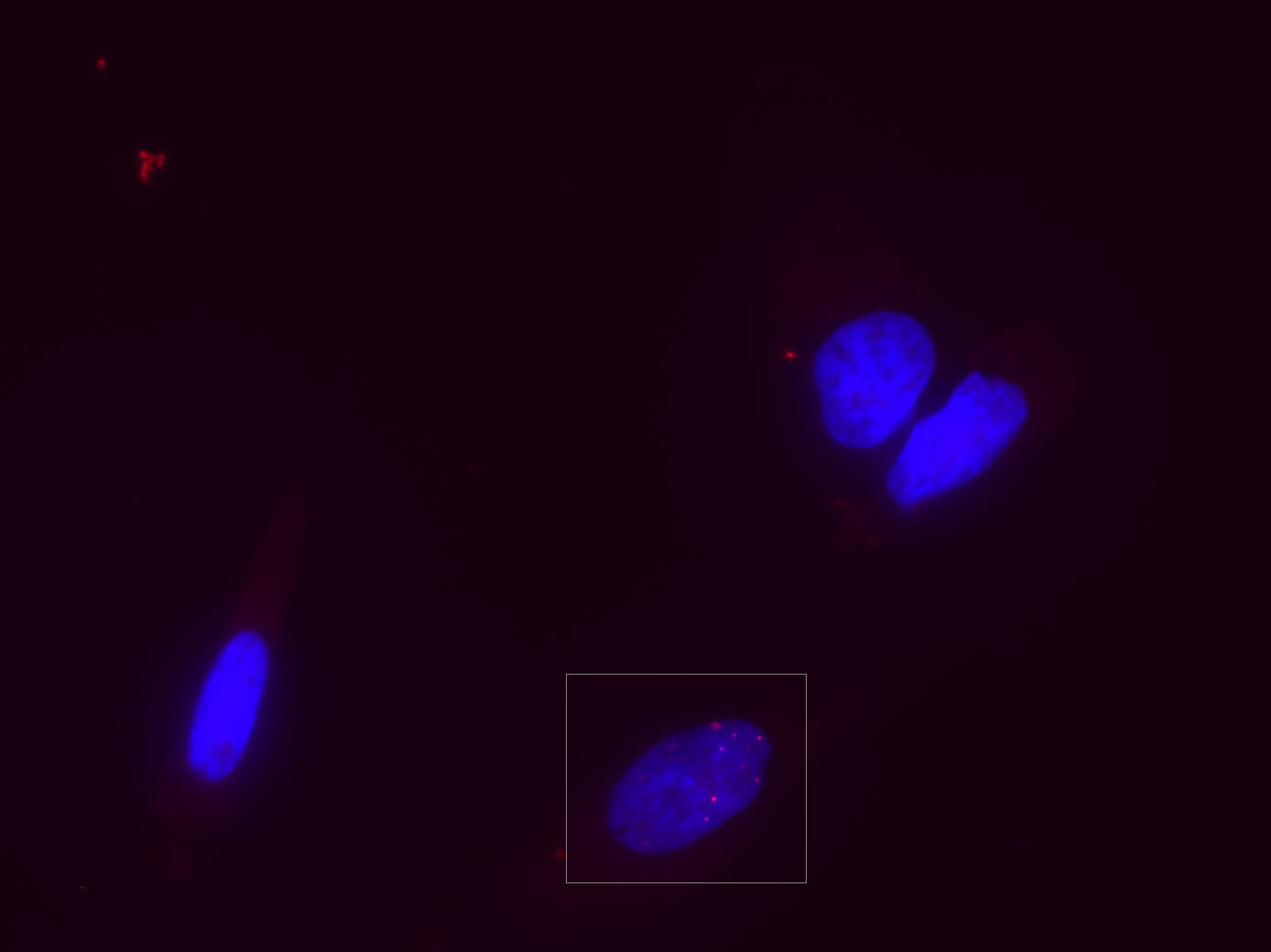

Supplement: Supplementary file 9 — Source data Fig. 6 [file 44319_2025_497_MOESM9_ESM.zip › 6G and 6H/shRNF20+ S553A RNF20.tif]

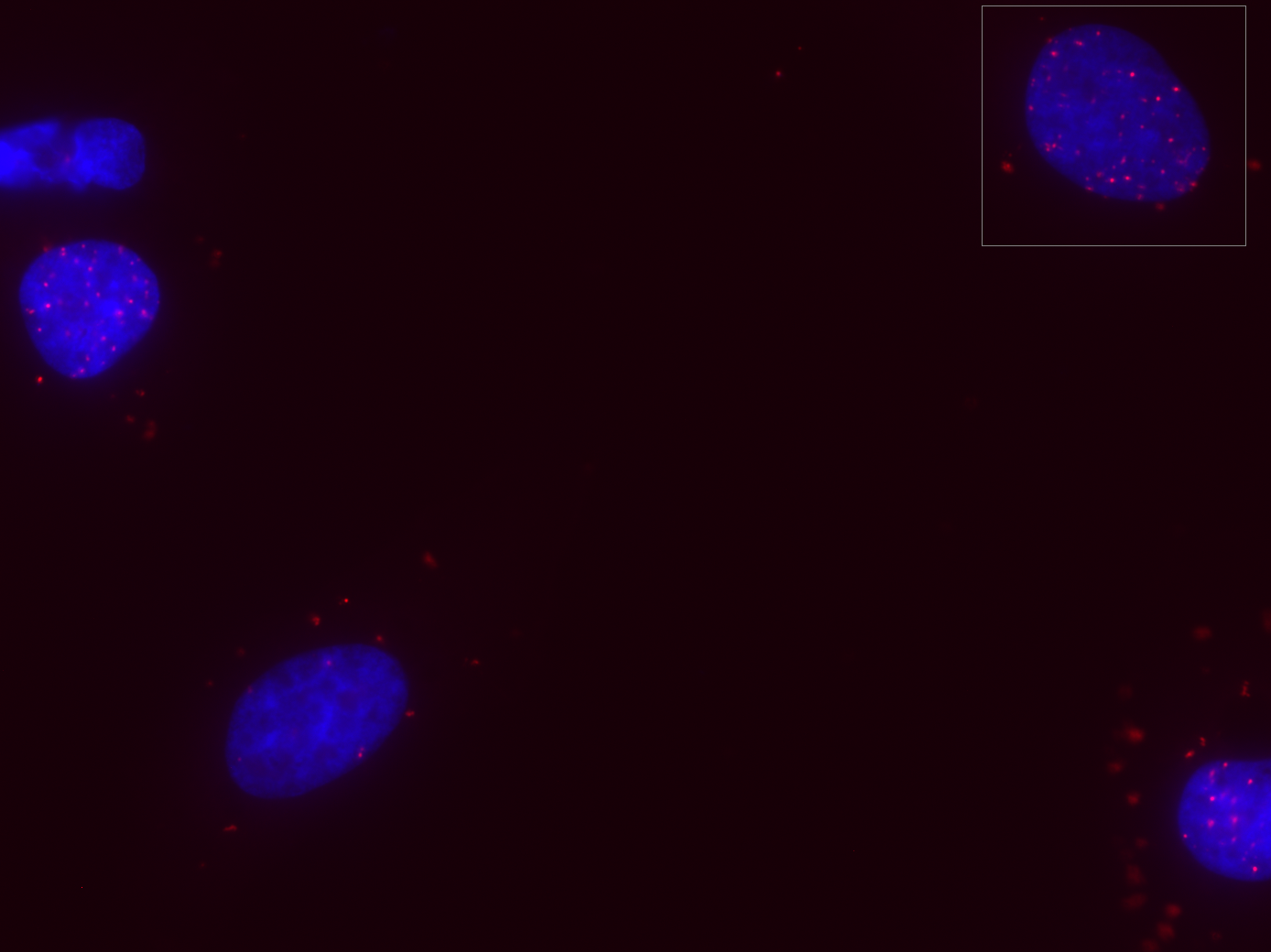

Supplement: Supplementary file 9 — Source data Fig. 6 [file 44319_2025_497_MOESM9_ESM.zip › 6G and 6H/shRNF20+ WT RNF20.tif]

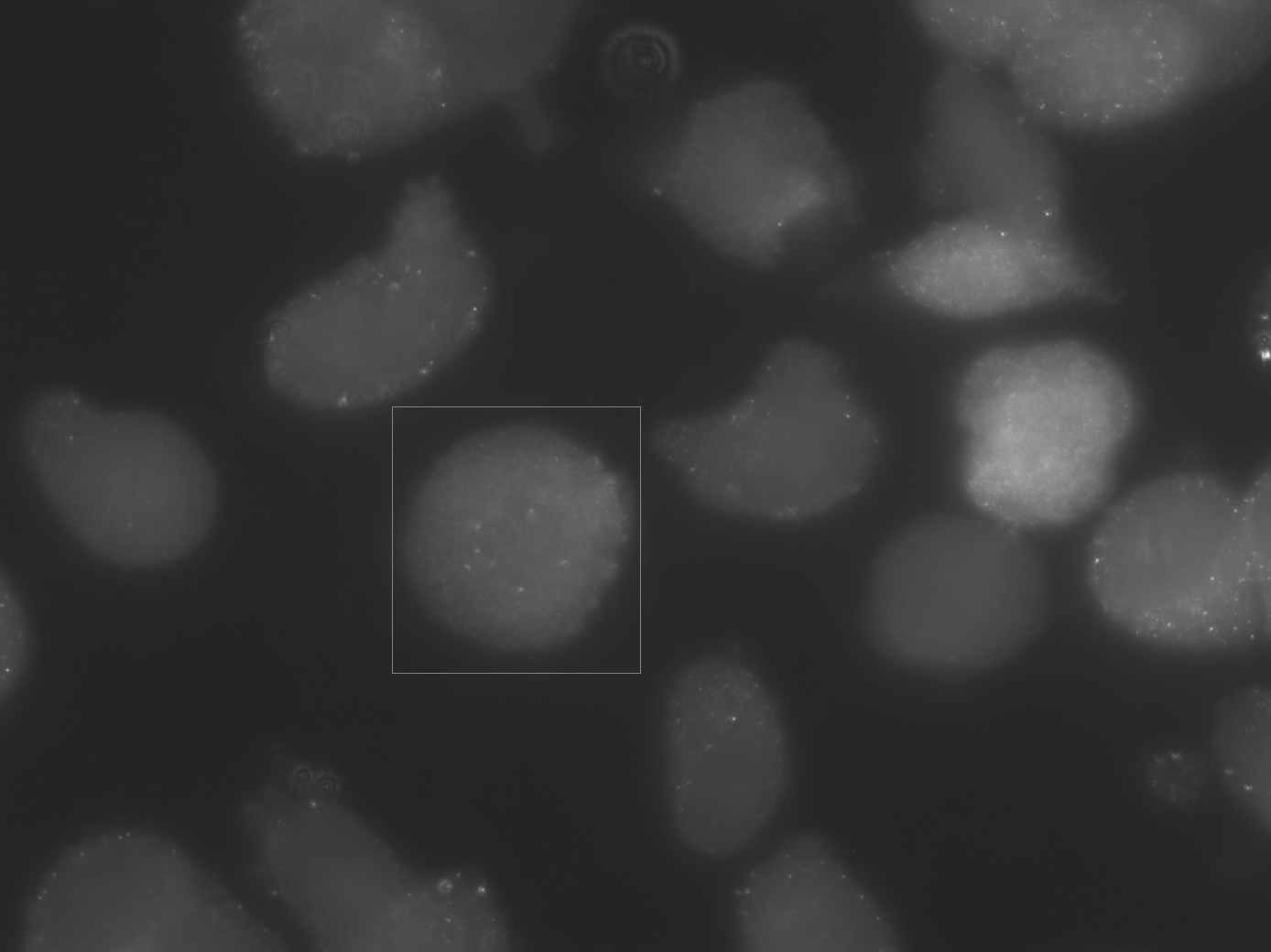

Supplement: Supplementary file 10 — Figure EV1 [file 44319_2025_497_MOESM10_ESM.zip › Figure EV 1A/shControl IF FANCD2 foci.tif]

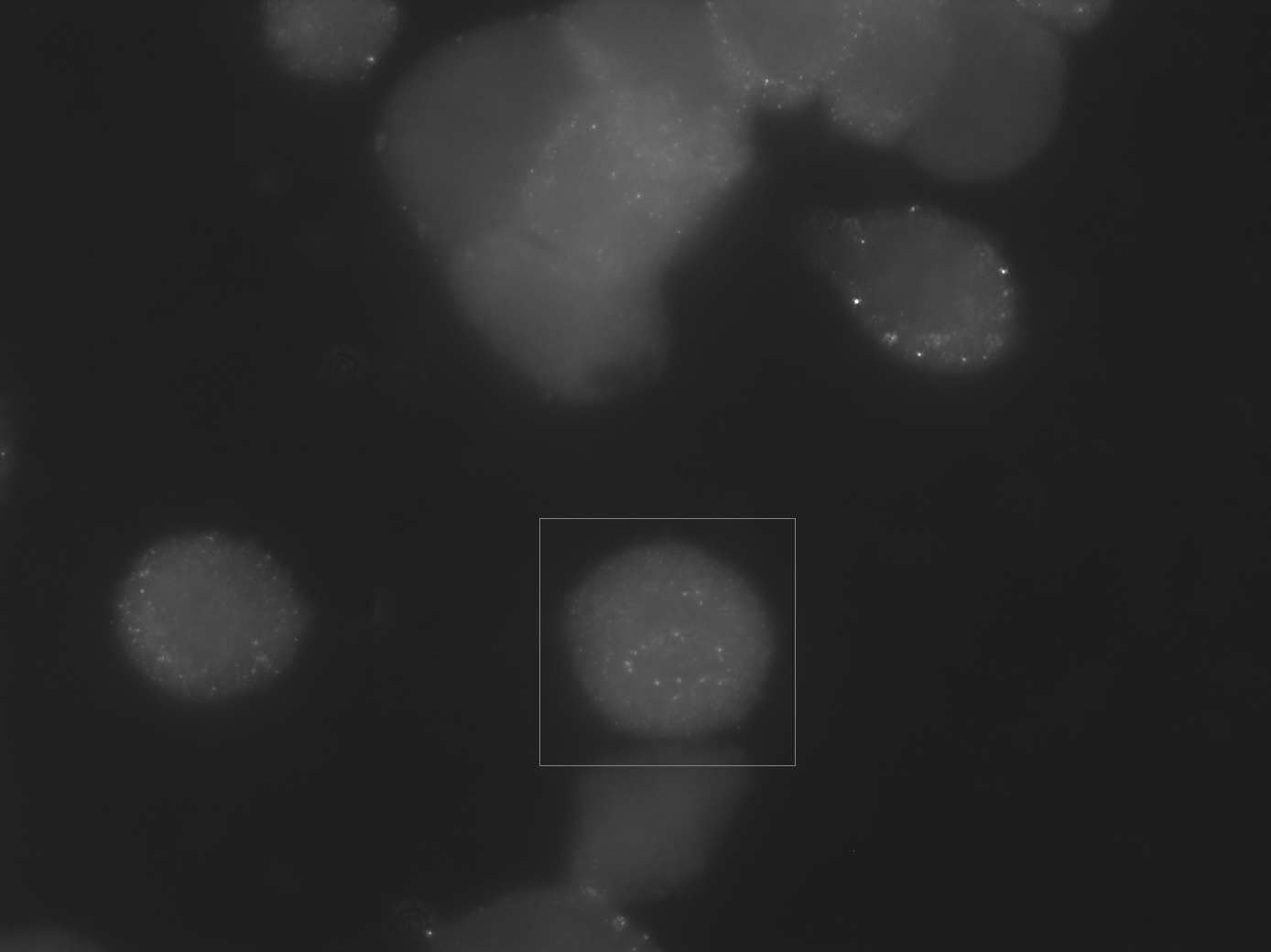

Supplement: Supplementary file 10 — Figure EV1 [file 44319_2025_497_MOESM10_ESM.zip › Figure EV 1A/shRNF20 IF FANCD2 foci.tif]

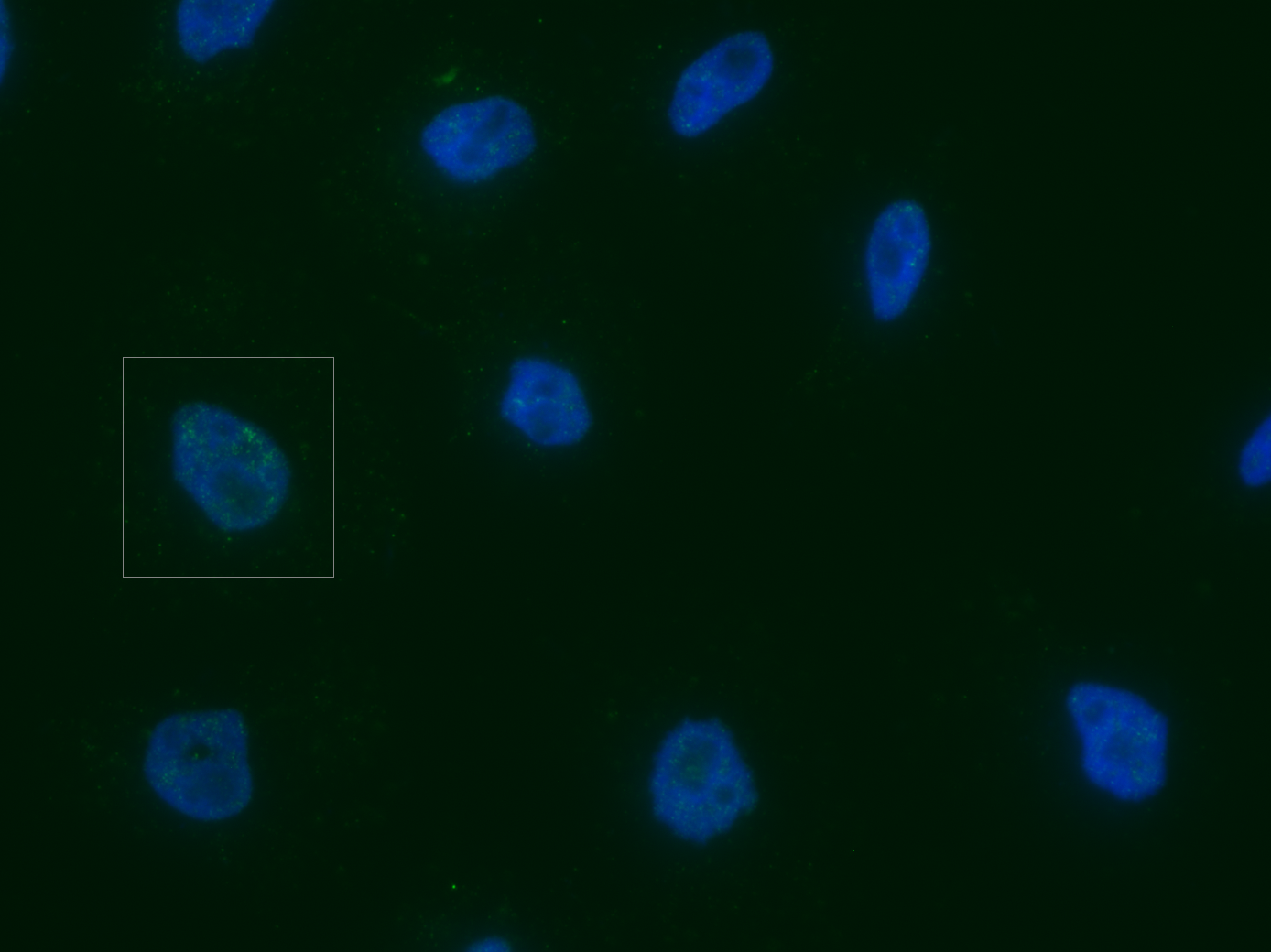

Supplement: Supplementary file 10 — Figure EV1 [file 44319_2025_497_MOESM10_ESM.zip › Figure EV 1C/shControl HU IF RPA32 p S4-S8.tif]

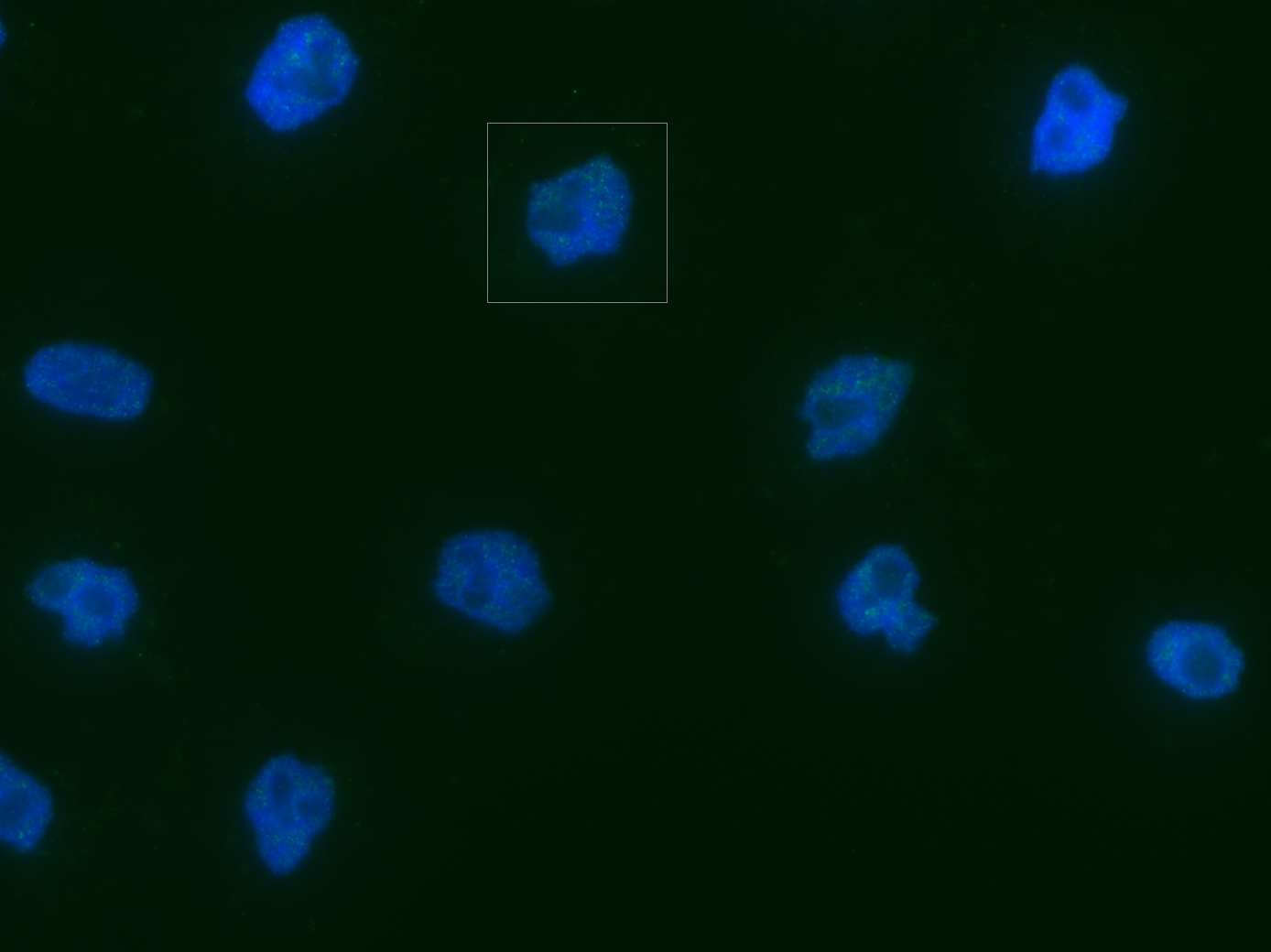

Supplement: Supplementary file 10 — Figure EV1 [file 44319_2025_497_MOESM10_ESM.zip › Figure EV 1C/shControl UT IF RPA32 p S4-S8.tif]

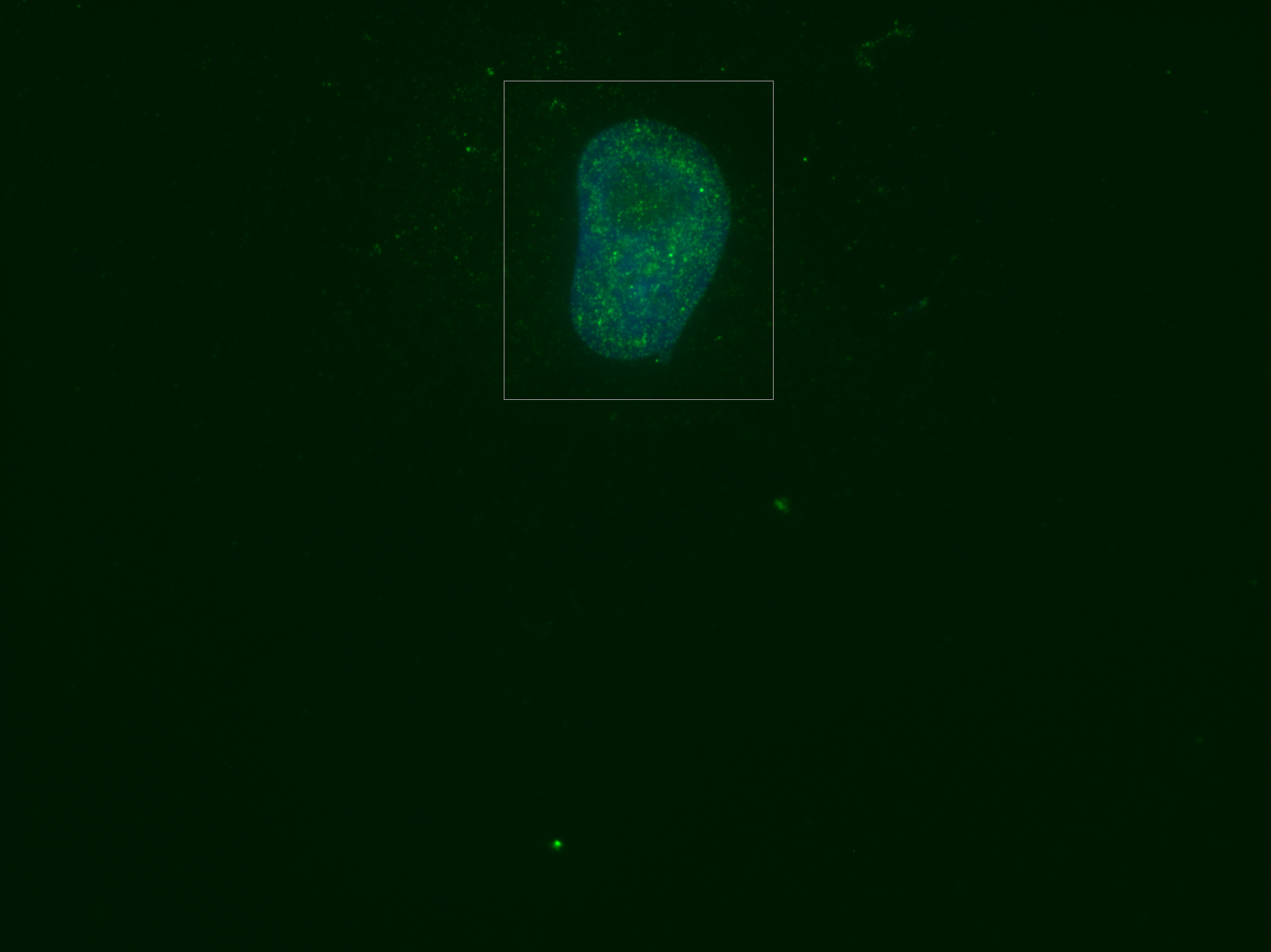

Supplement: Supplementary file 10 — Figure EV1 [file 44319_2025_497_MOESM10_ESM.zip › Figure EV 1C/shRNF20 HU IF RPA32 p S4-S8.tif]

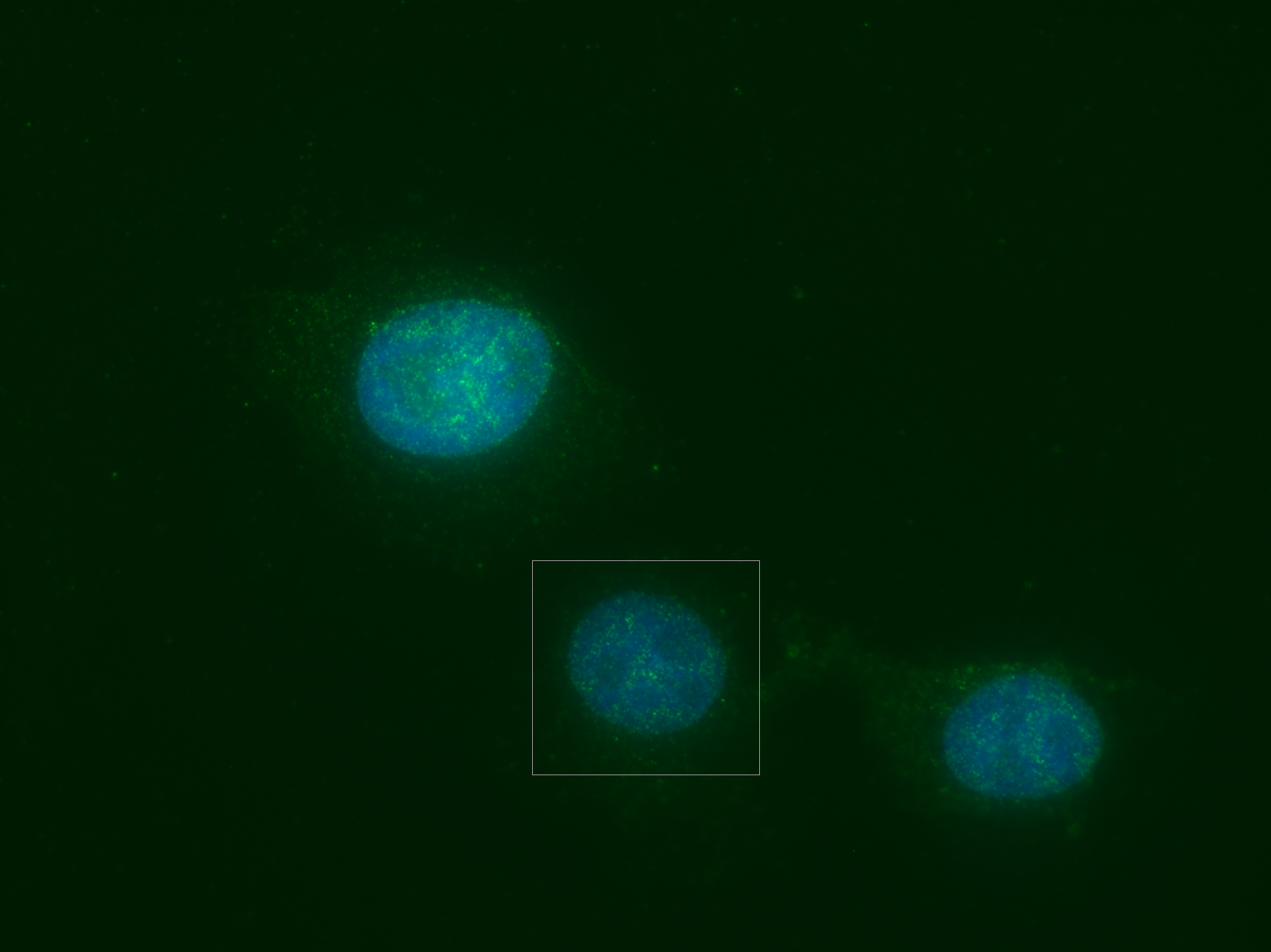

Supplement: Supplementary file 10 — Figure EV1 [file 44319_2025_497_MOESM10_ESM.zip › Figure EV 1C/shRNF20 UT IF RPA32 p S4-S8.tif]

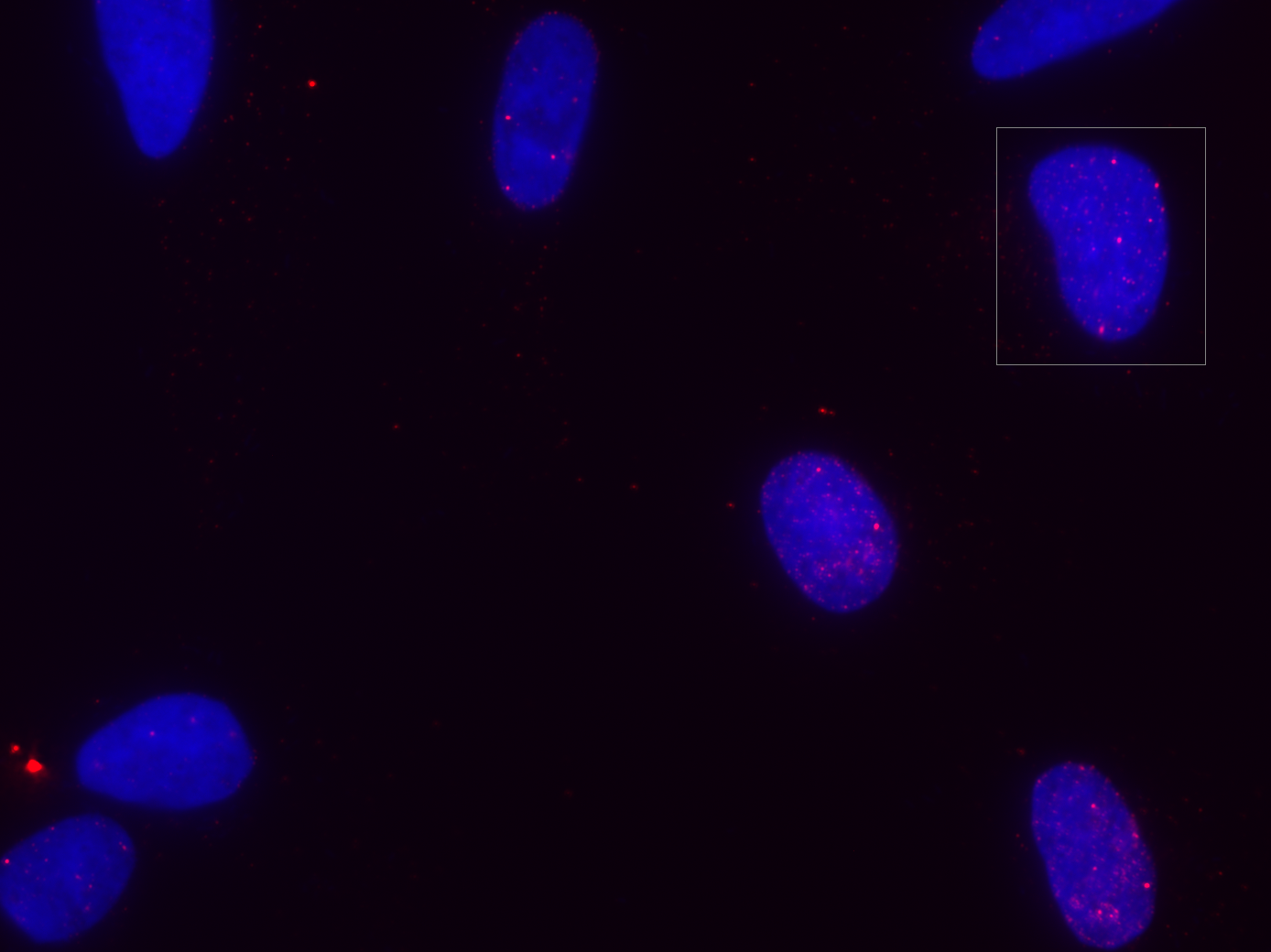

Supplement: Supplementary file 10 — Figure EV1 [file 44319_2025_497_MOESM10_ESM.zip › Figure EV 1E/shControl HU IF BrdU foci.tif]

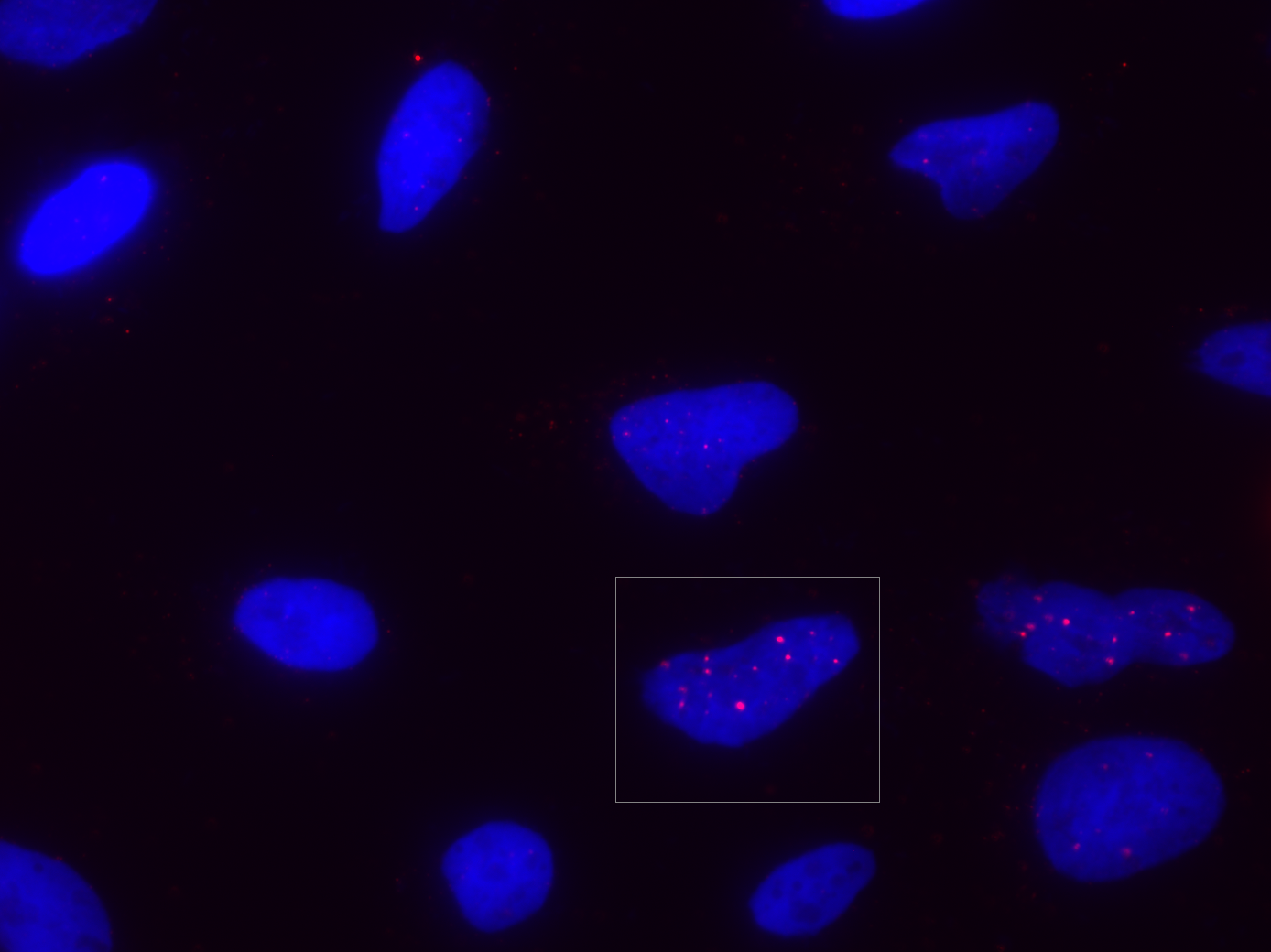

Supplement: Supplementary file 10 — Figure EV1 [file 44319_2025_497_MOESM10_ESM.zip › Figure EV 1E/shControl UT IF BrdU foci.tif]

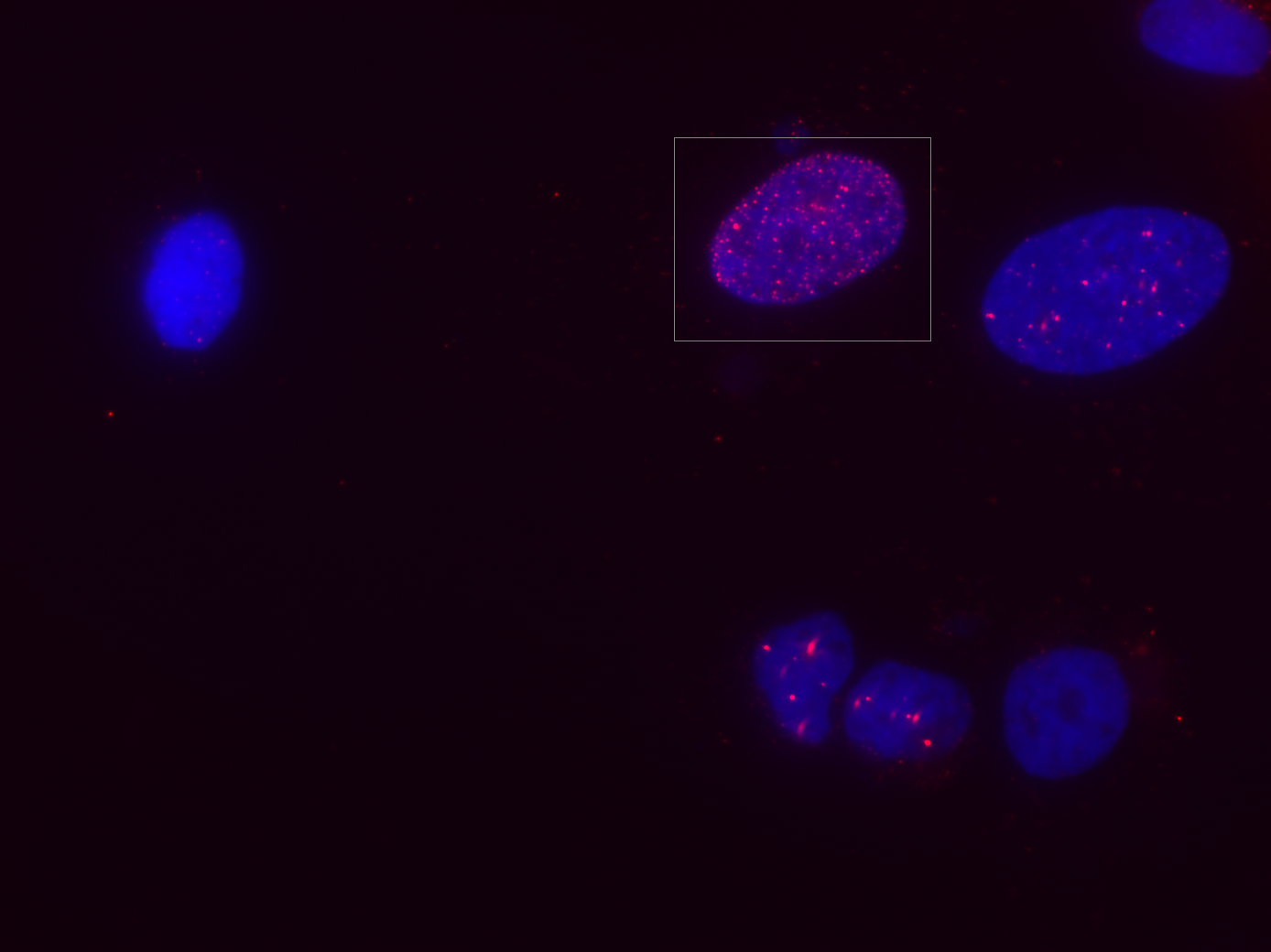

Supplement: Supplementary file 10 — Figure EV1 [file 44319_2025_497_MOESM10_ESM.zip › Figure EV 1E/shRNF20 HU IF BrdU foci.tif]

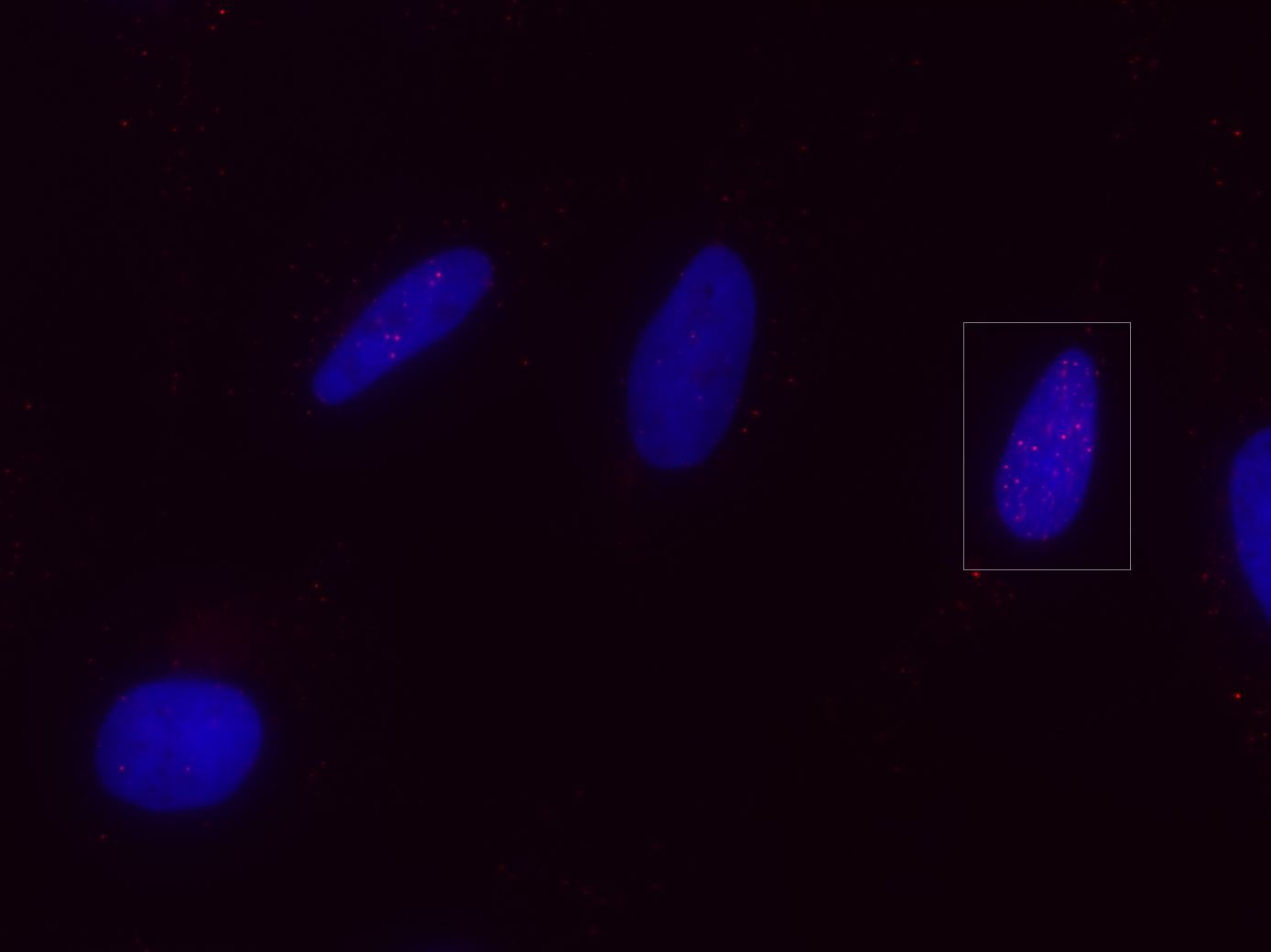

Supplement: Supplementary file 10 — Figure EV1 [file 44319_2025_497_MOESM10_ESM.zip › Figure EV 1E/shRNF20 UT IF BrdU foci.tif]

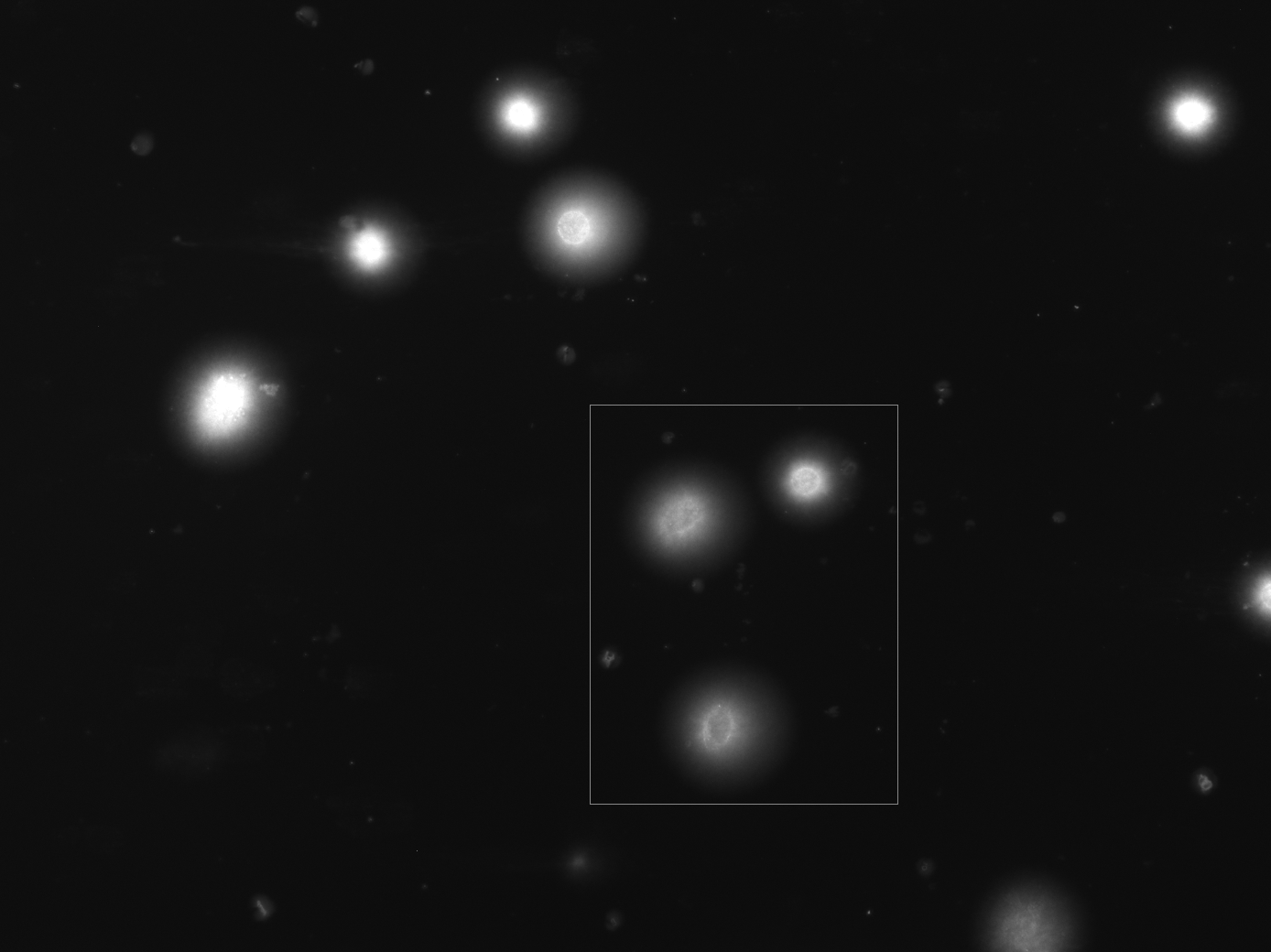

Supplement: Supplementary file 10 — Figure EV1 [file 44319_2025_497_MOESM10_ESM.zip › Figure EV 1G/shControl HU Comet.tif]

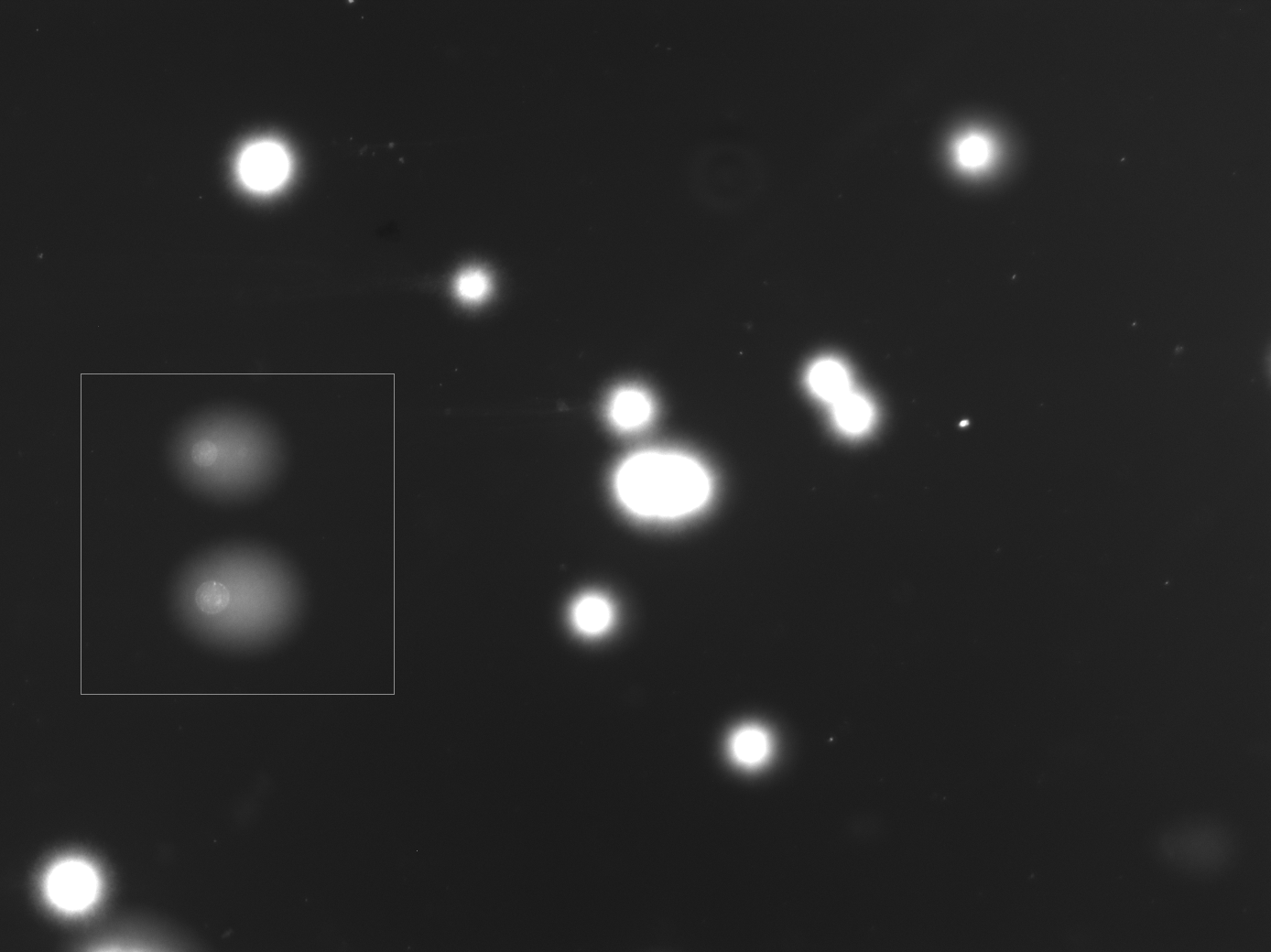

Supplement: Supplementary file 10 — Figure EV1 [file 44319_2025_497_MOESM10_ESM.zip › Figure EV 1G/shRNF20 HU Comet.tif]

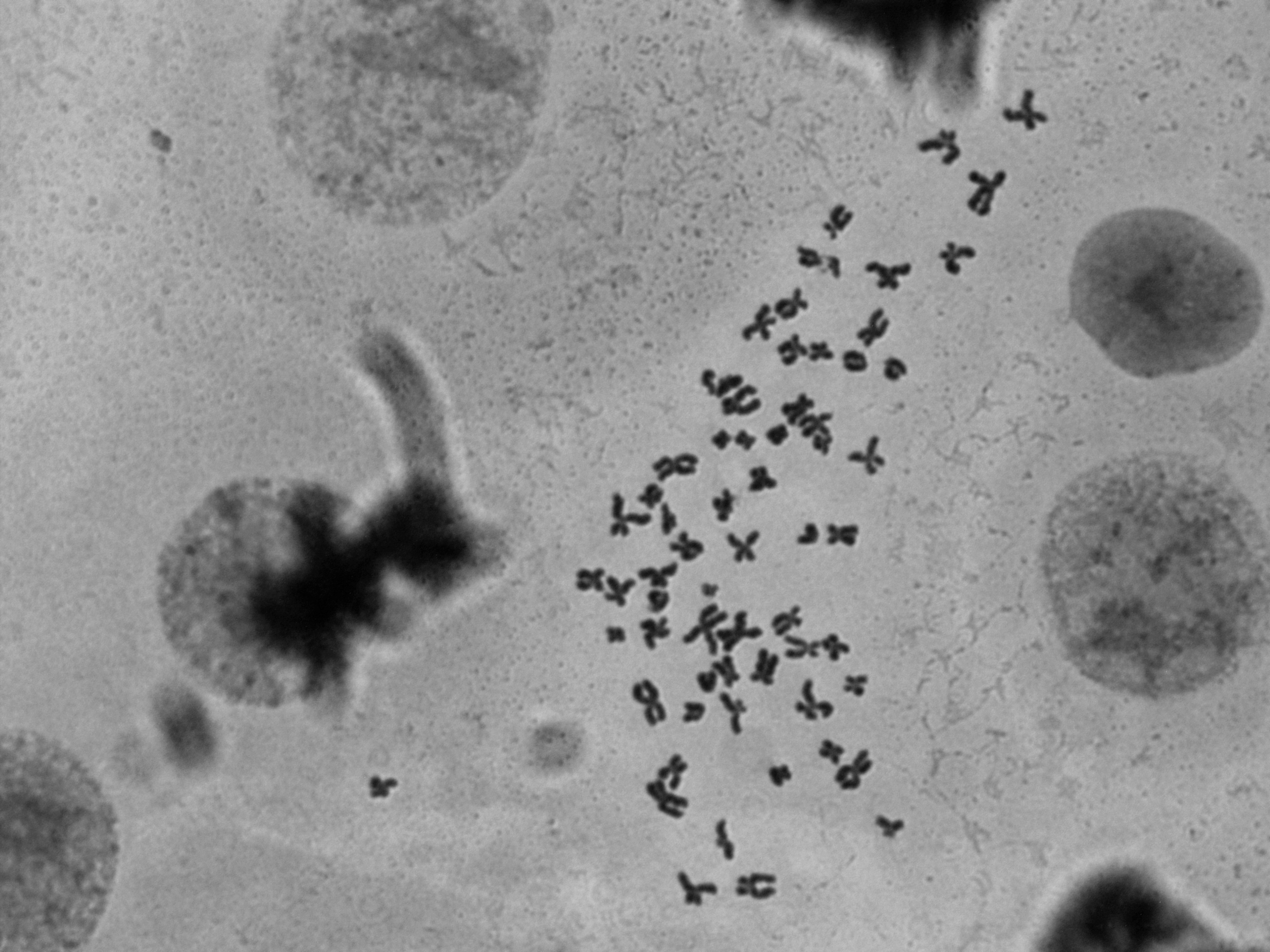

Supplement: Supplementary file 10 — Figure EV1 [file 44319_2025_497_MOESM10_ESM.zip › Figure EV 1I/shControl HU metaphase spread.tif]

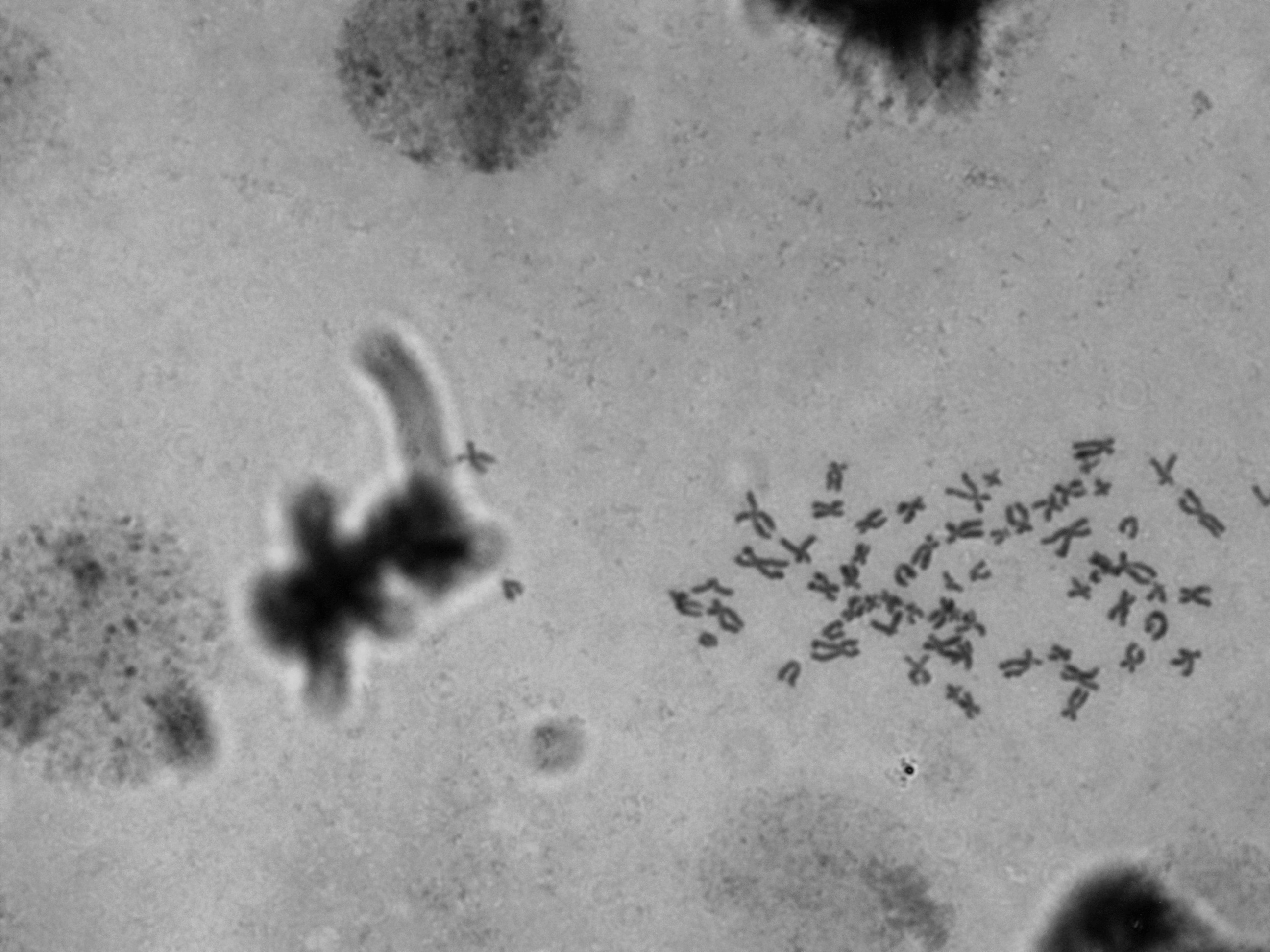

Supplement: Supplementary file 10 — Figure EV1 [file 44319_2025_497_MOESM10_ESM.zip › Figure EV 1I/shControl UT metaphase spread.tif]

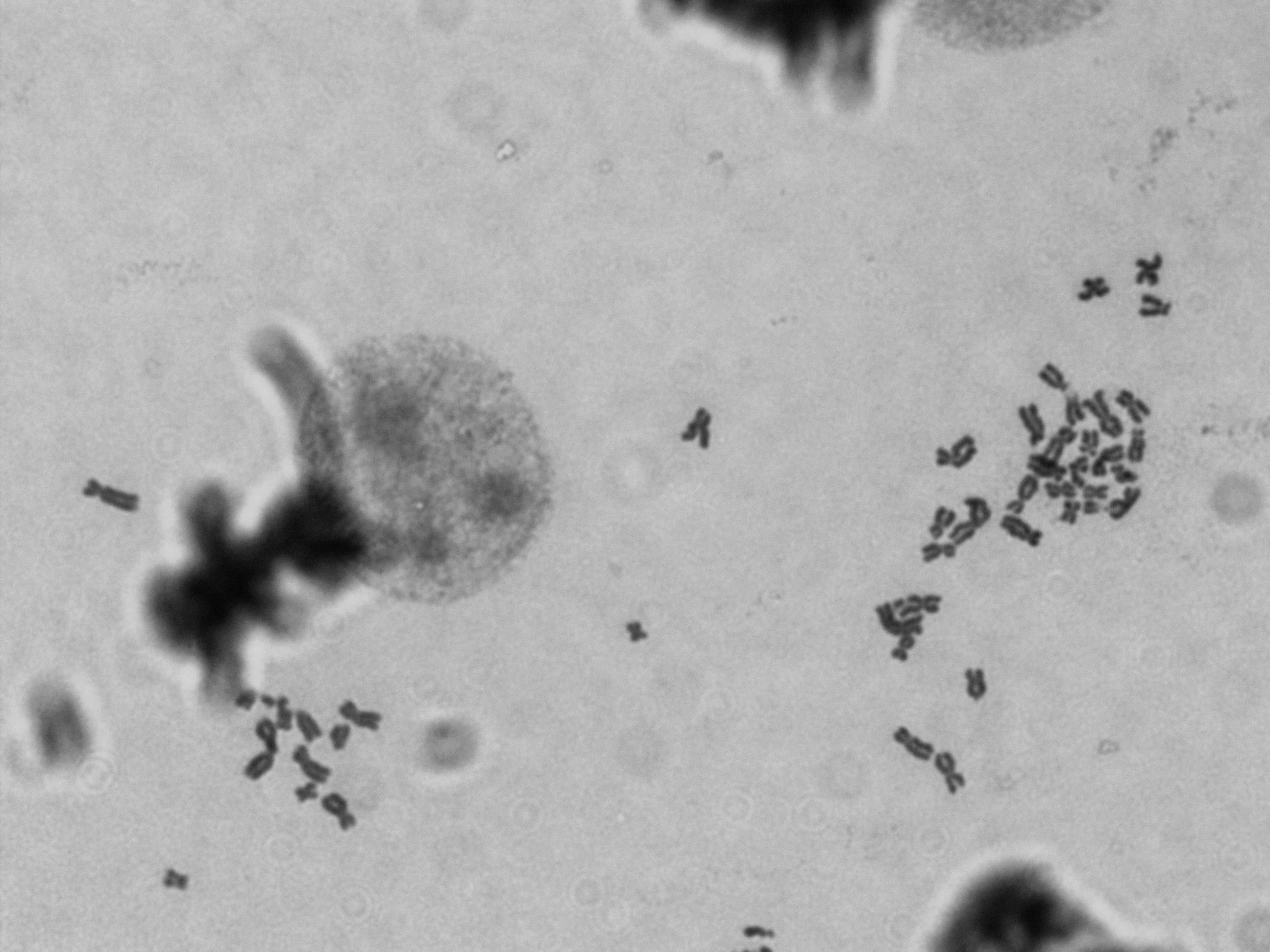

Supplement: Supplementary file 10 — Figure EV1 [file 44319_2025_497_MOESM10_ESM.zip › Figure EV 1I/shRNF20 HU metaphase spread.tif]

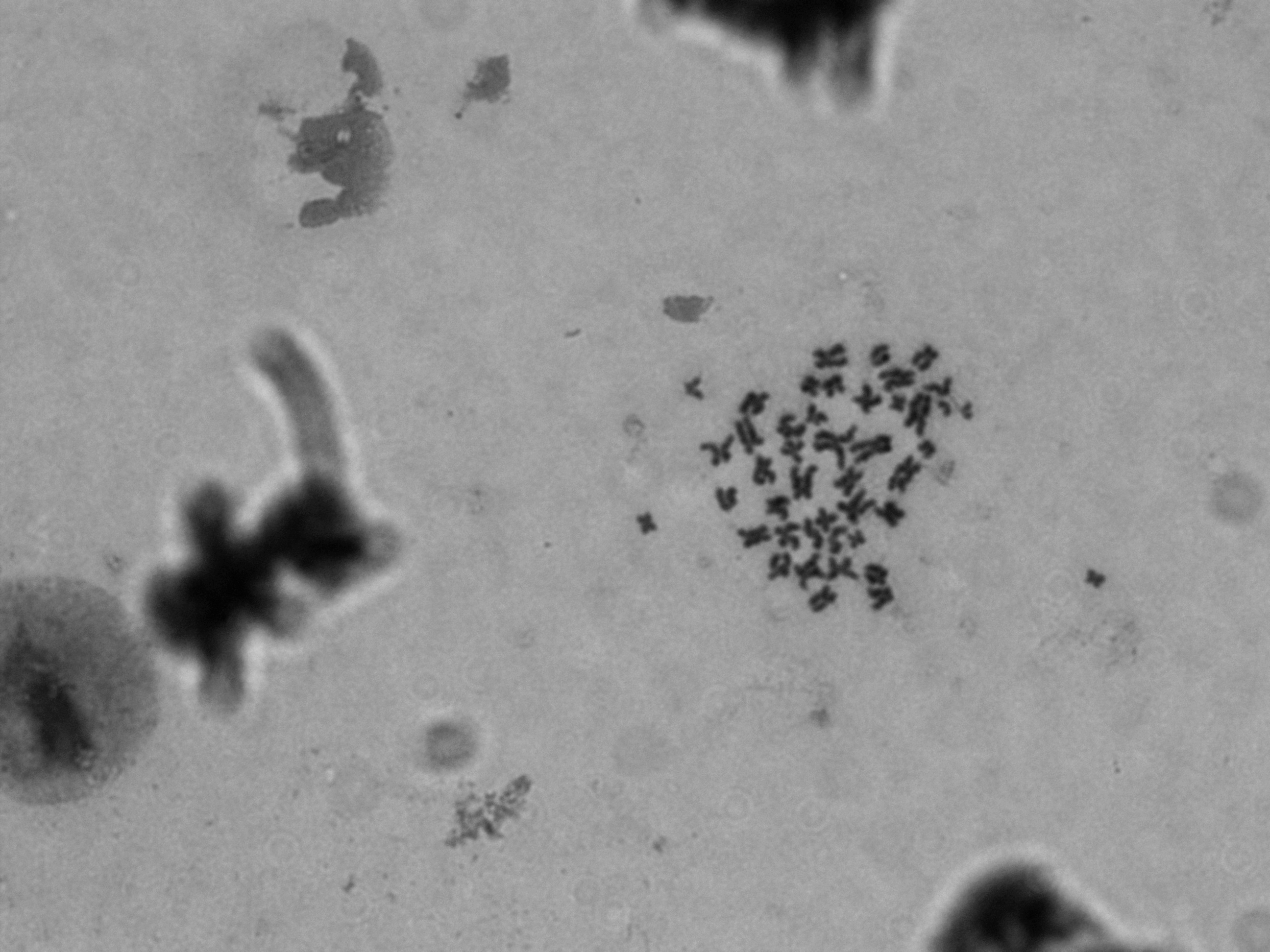

Supplement: Supplementary file 10 — Figure EV1 [file 44319_2025_497_MOESM10_ESM.zip › Figure EV 1I/shRNF20 UT metaphase spread.tif]

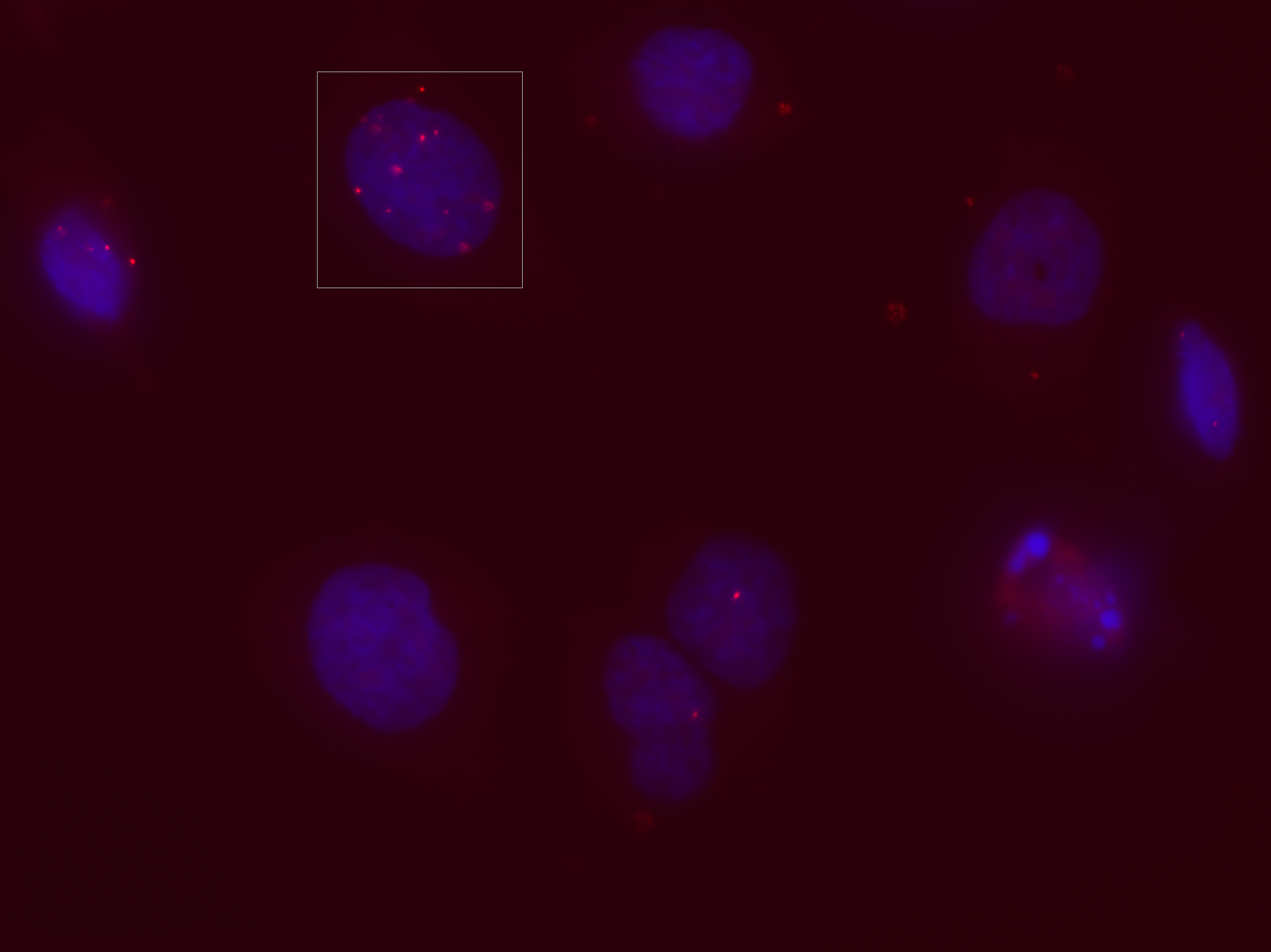

Supplement: Supplementary file 11 — Figure EV2 [file 44319_2025_497_MOESM11_ESM.zip › Figure EV 2A/shControl Progressing H2B K120ub SIRF Only EdU.tif]

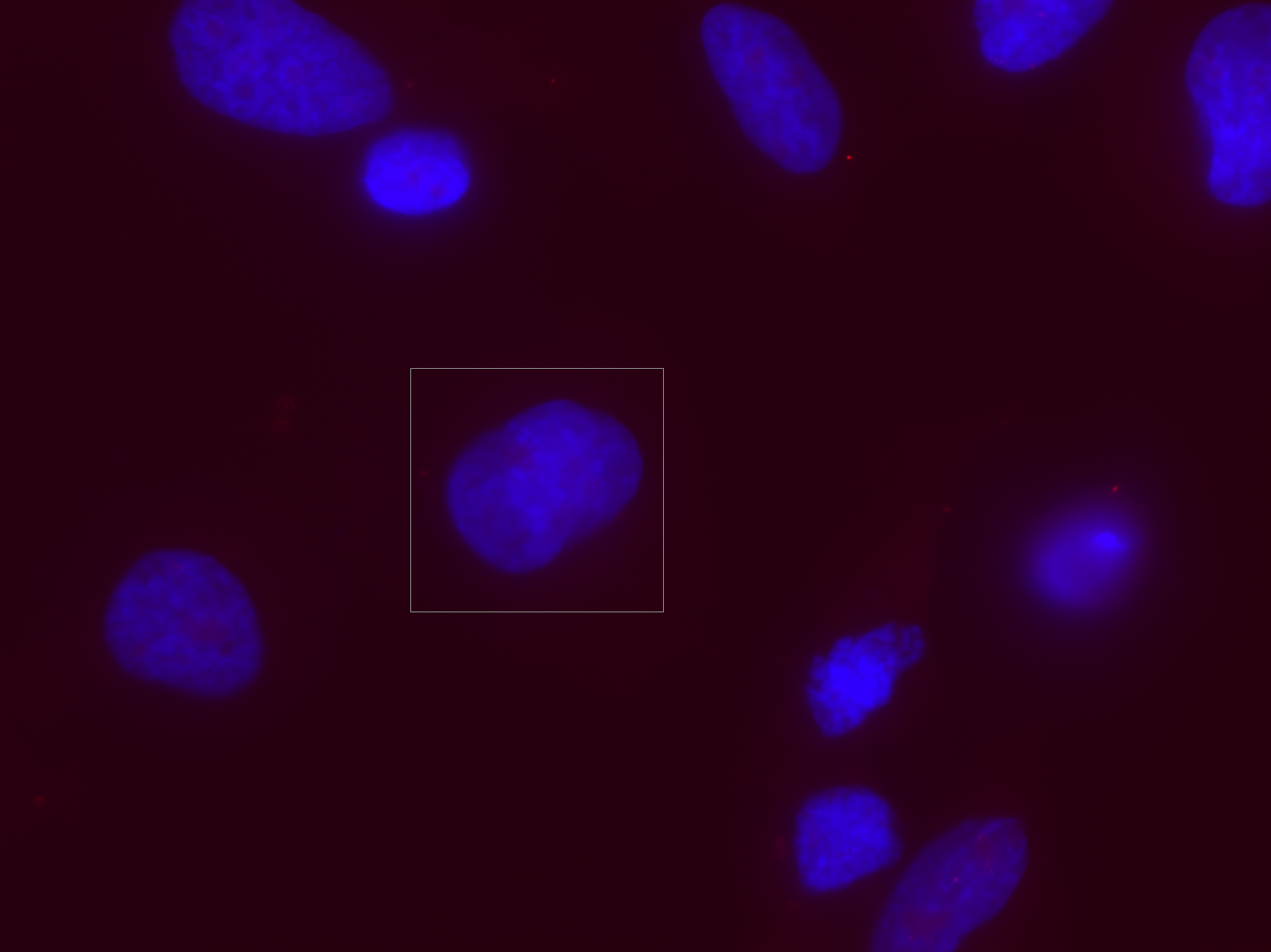

Supplement: Supplementary file 11 — Figure EV2 [file 44319_2025_497_MOESM11_ESM.zip › Figure EV 2A/shControl Stall H2B K120ub SIRF Only EdU.tif]

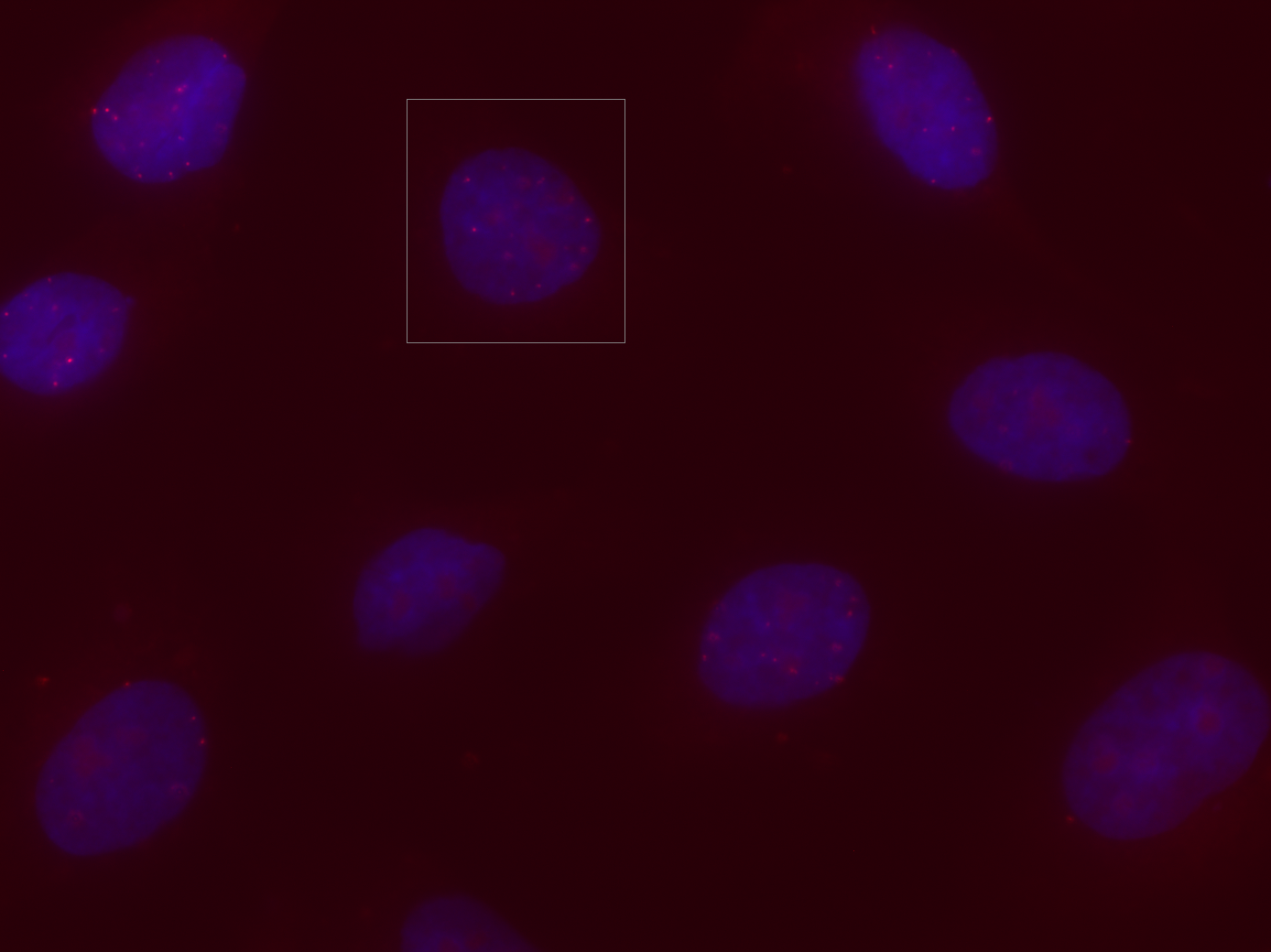

Supplement: Supplementary file 11 — Figure EV2 [file 44319_2025_497_MOESM11_ESM.zip › Figure EV 2A/shRNF20 Progressing H2B K120ub SIRF Only EdU.tif]

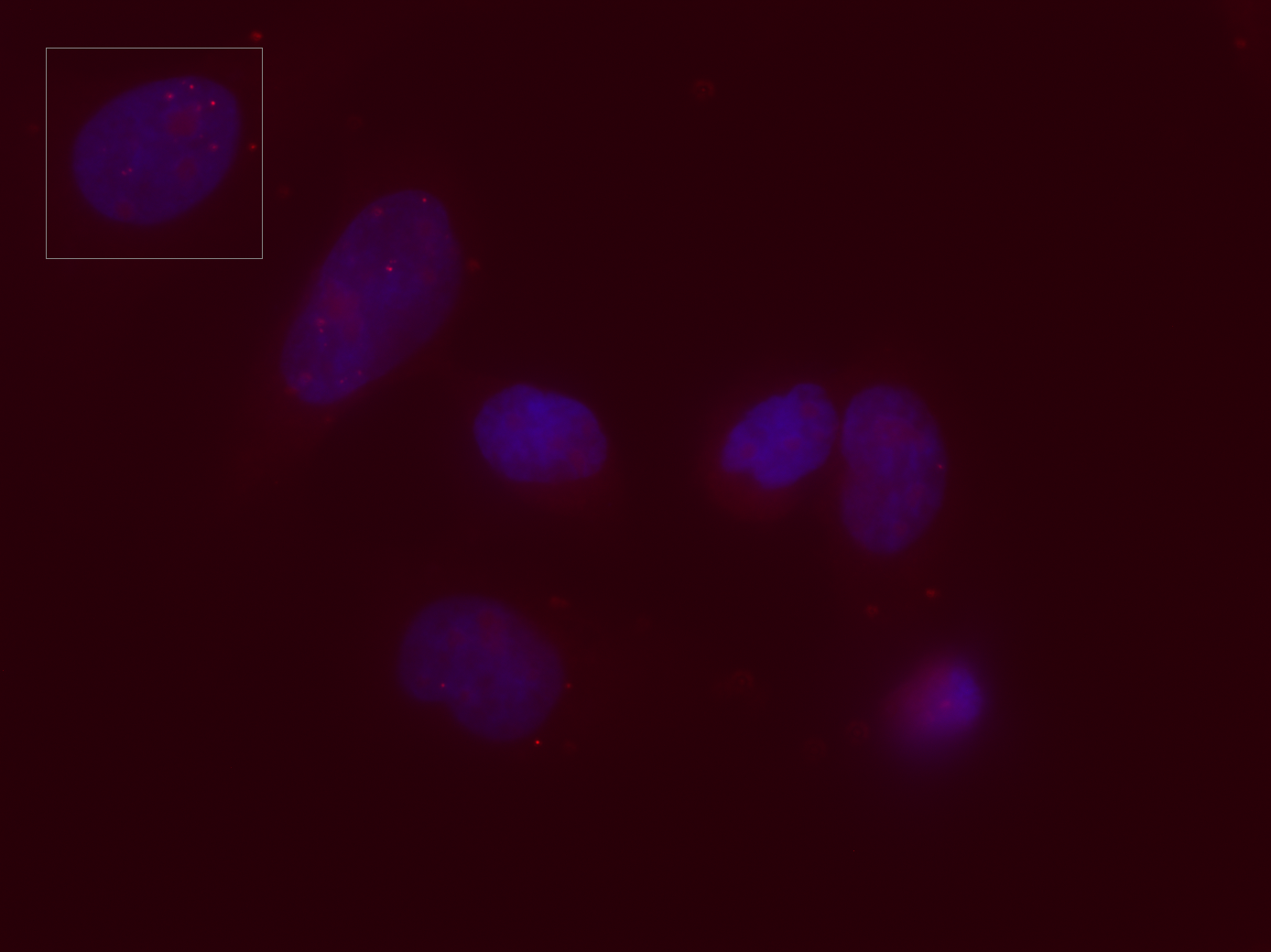

Supplement: Supplementary file 11 — Figure EV2 [file 44319_2025_497_MOESM11_ESM.zip › Figure EV 2A/shRNF20 Stall H2B K120ub SIRF Only EdU.tif]

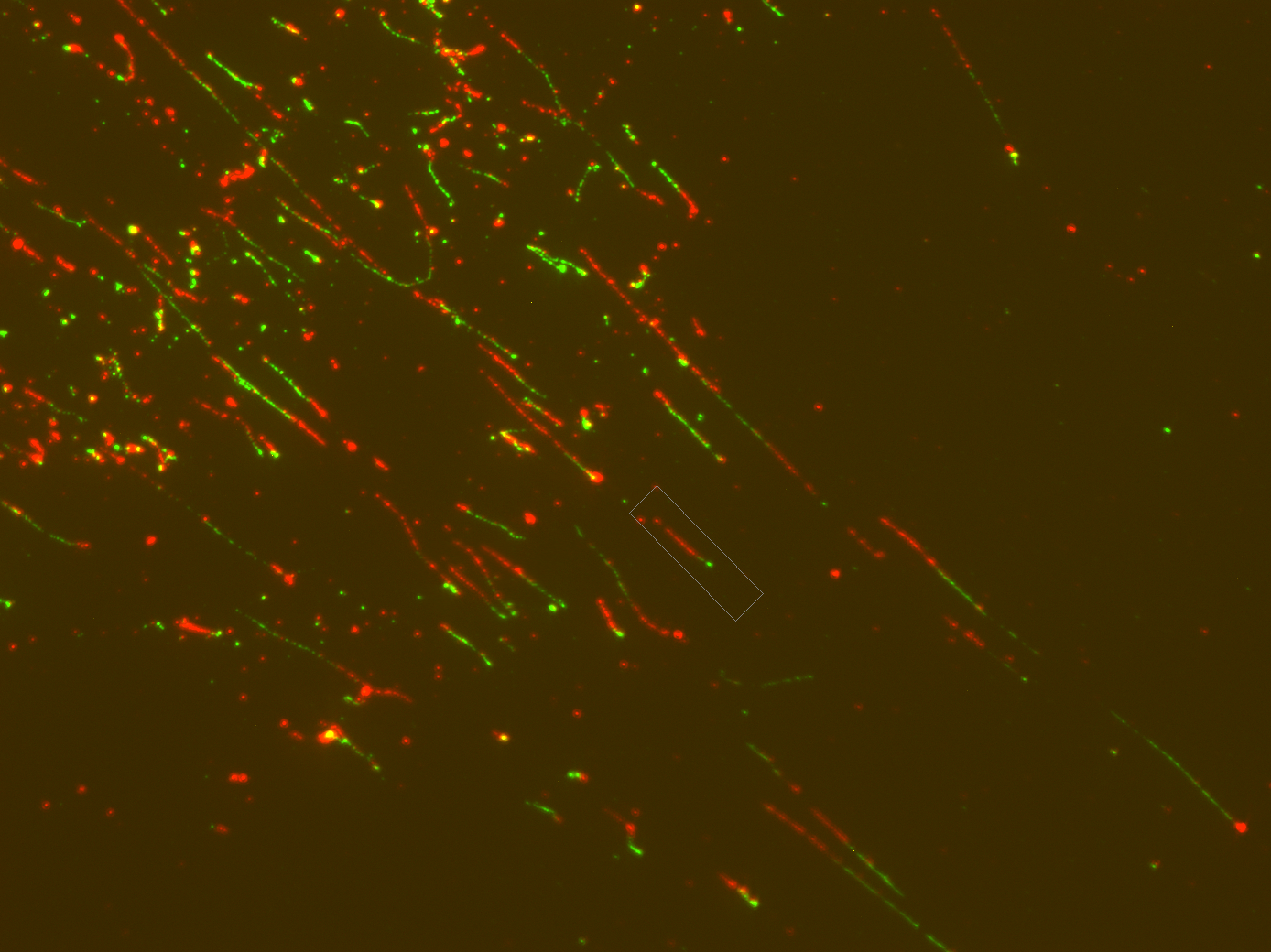

Supplement: Supplementary file 11 — Figure EV2 [file 44319_2025_497_MOESM11_ESM.zip › Figure EV 2F/shBRCA2 DNA fiber fork degradation.tif]

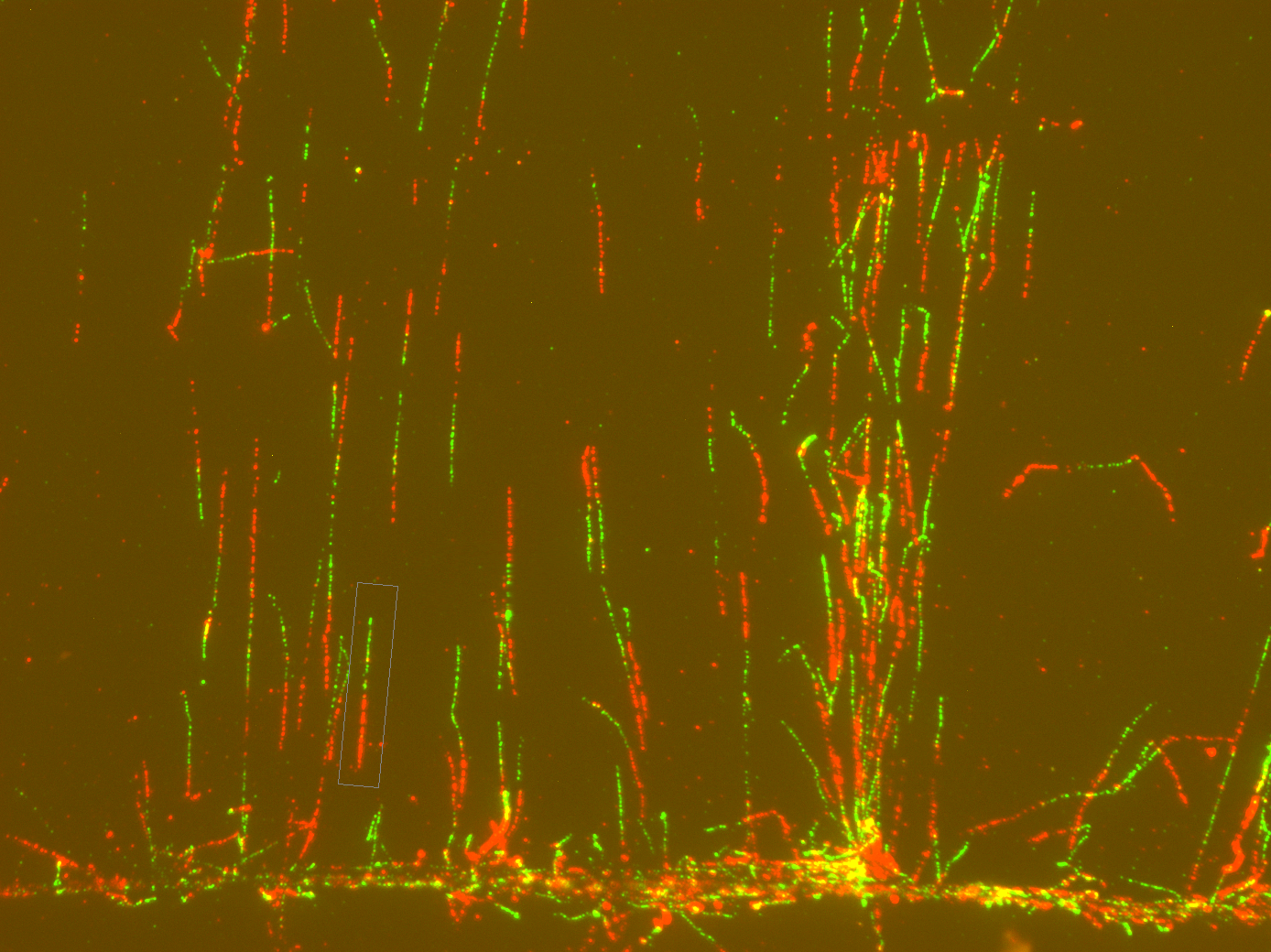

Supplement: Supplementary file 11 — Figure EV2 [file 44319_2025_497_MOESM11_ESM.zip › Figure EV 2F/shControl DNA fiber fork degradation.tif]

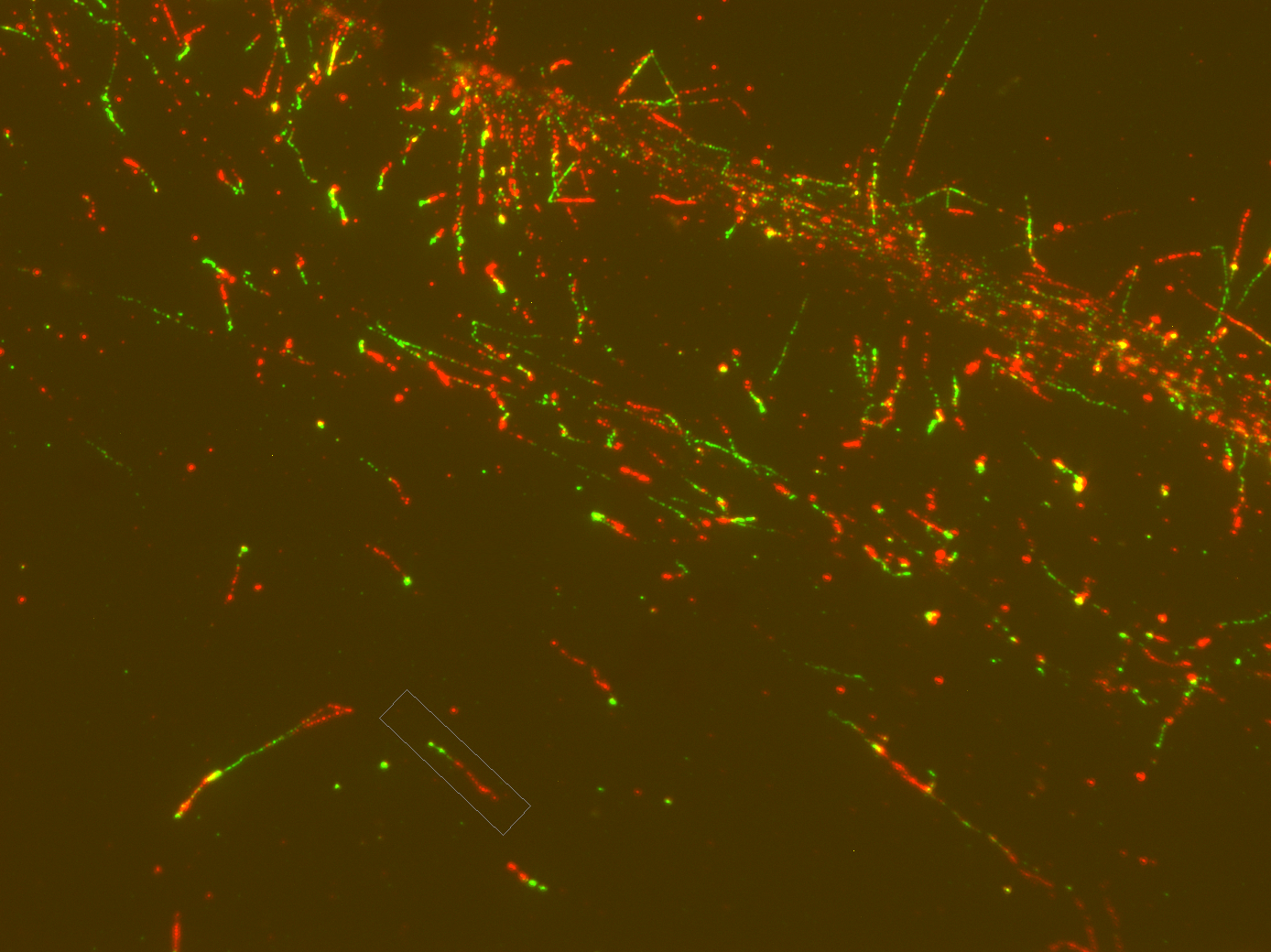

Supplement: Supplementary file 11 — Figure EV2 [file 44319_2025_497_MOESM11_ESM.zip › Figure EV 2F/shRNF20 + shBRCA2 DNA fiber fork degradation.tif]

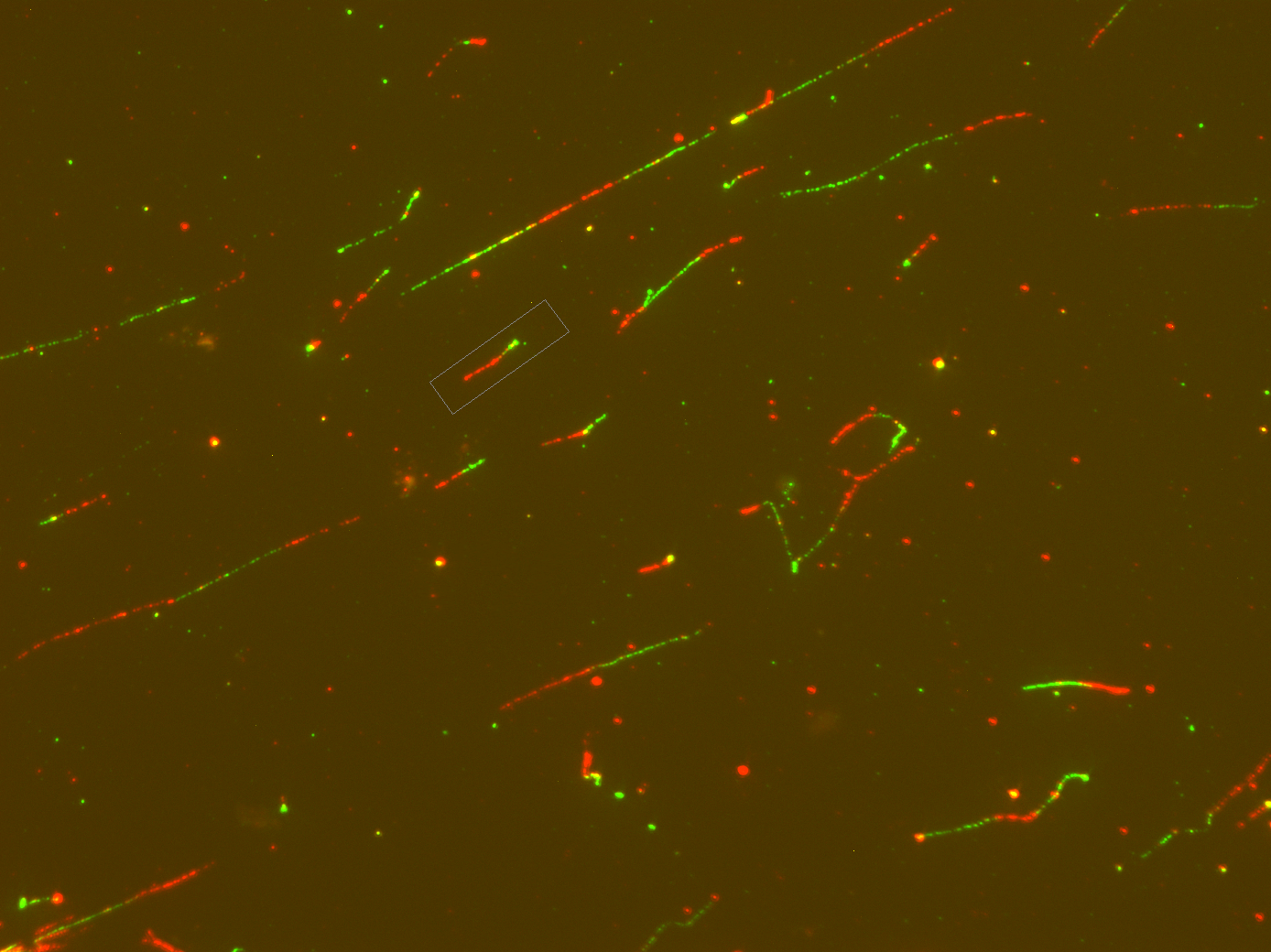

Supplement: Supplementary file 11 — Figure EV2 [file 44319_2025_497_MOESM11_ESM.zip › Figure EV 2F/shRNF20 DNA fiber fork degradation.tif]

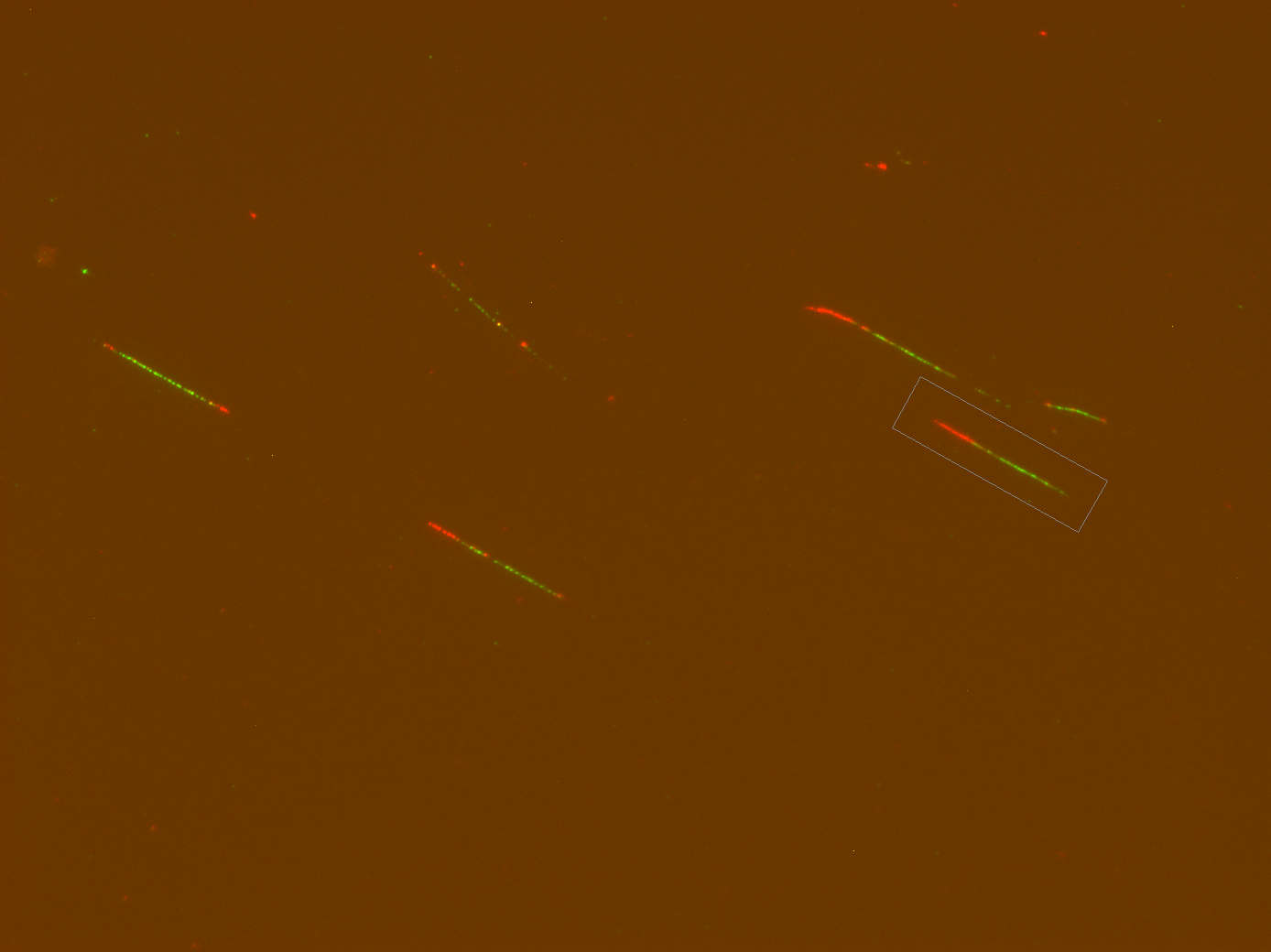

Supplement: Supplementary file 11 — Figure EV2 [file 44319_2025_497_MOESM11_ESM.zip › Figure EV 2H/shControl DNA fiber fork degradation.tif]

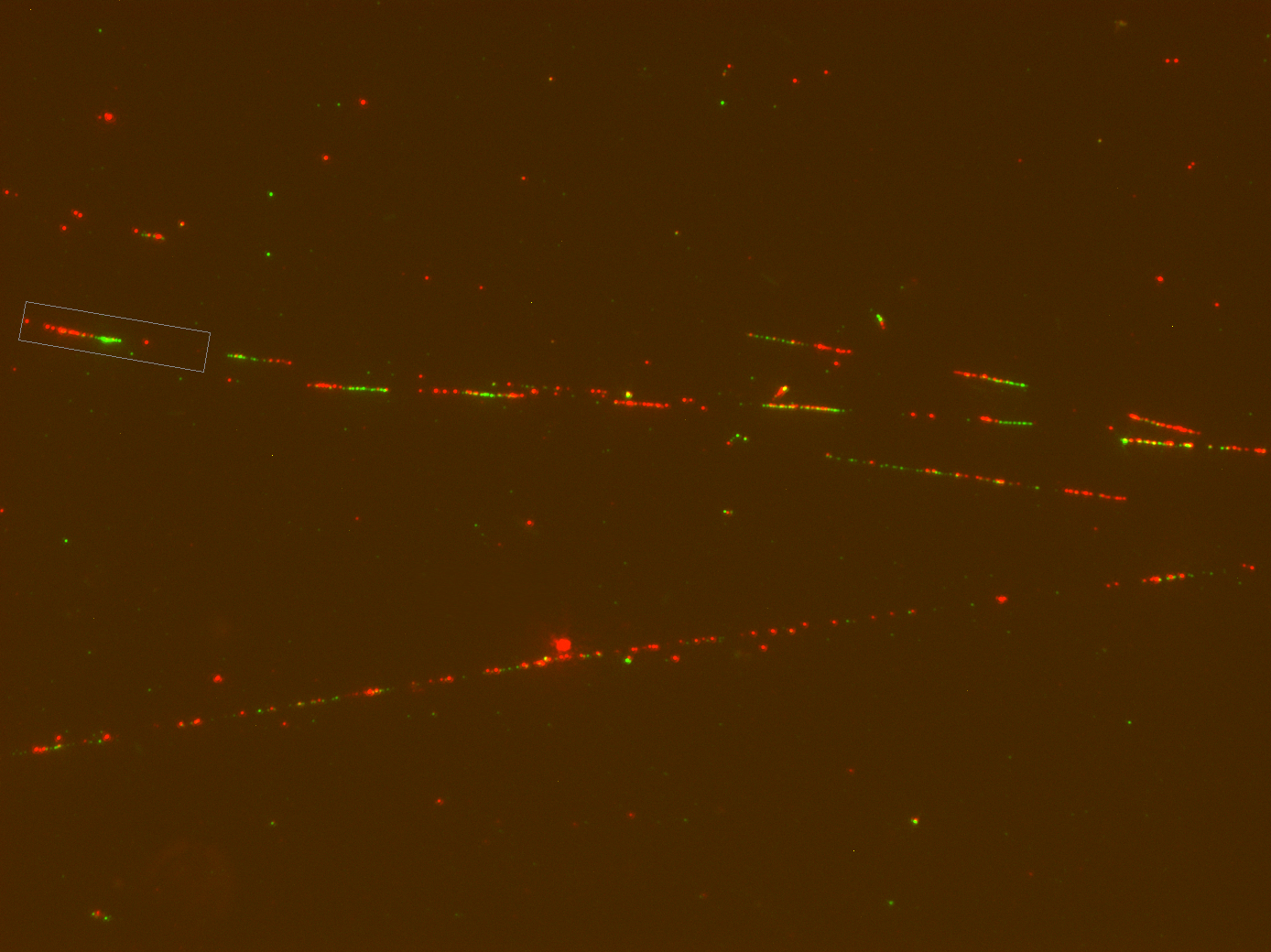

Supplement: Supplementary file 11 — Figure EV2 [file 44319_2025_497_MOESM11_ESM.zip › Figure EV 2H/shRAD51C DNA fiber fork degradation.tif]

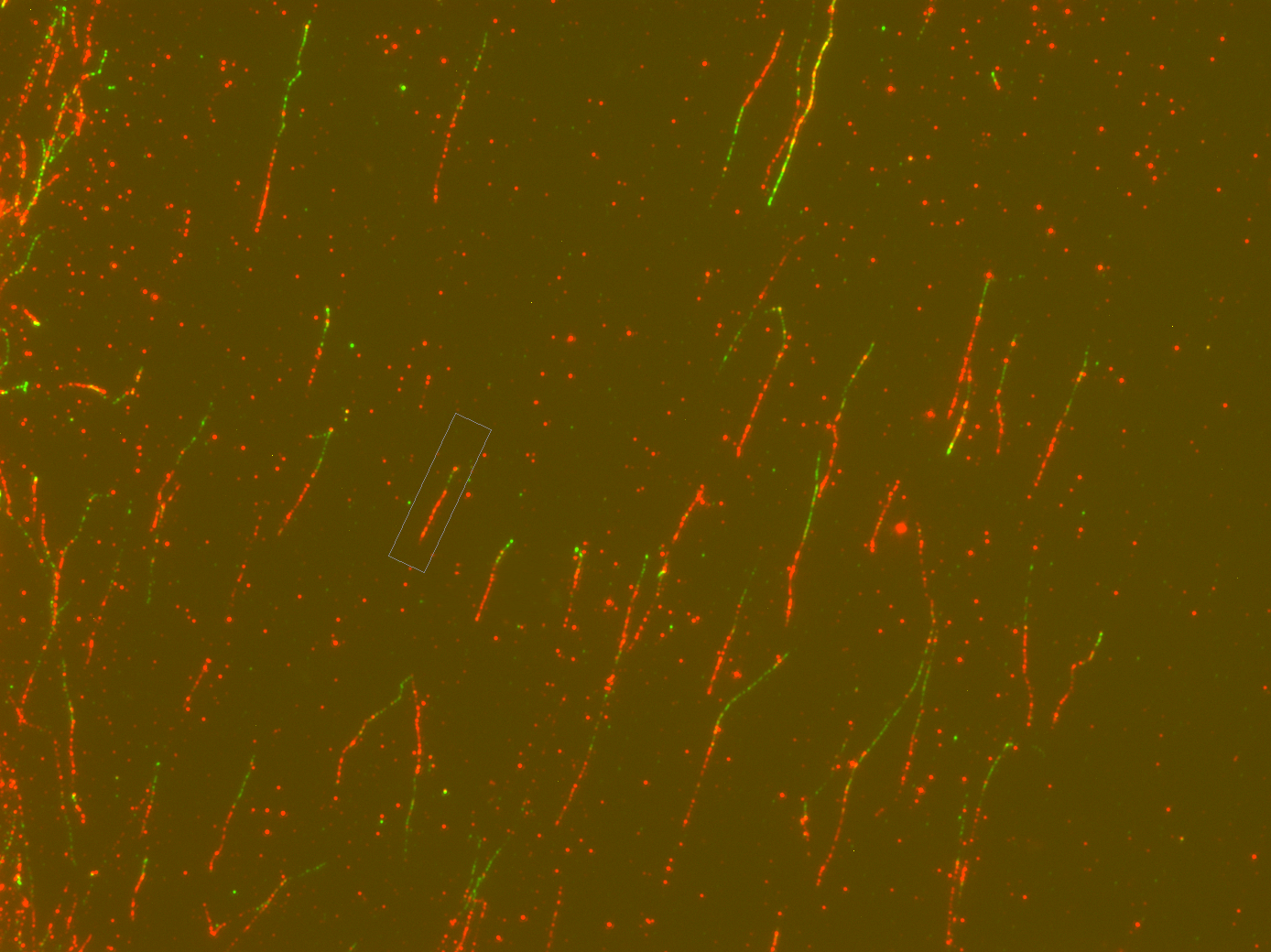

Supplement: Supplementary file 11 — Figure EV2 [file 44319_2025_497_MOESM11_ESM.zip › Figure EV 2H/shRNF20 + shXRCC2 DNA fiber fork degradation.tif]

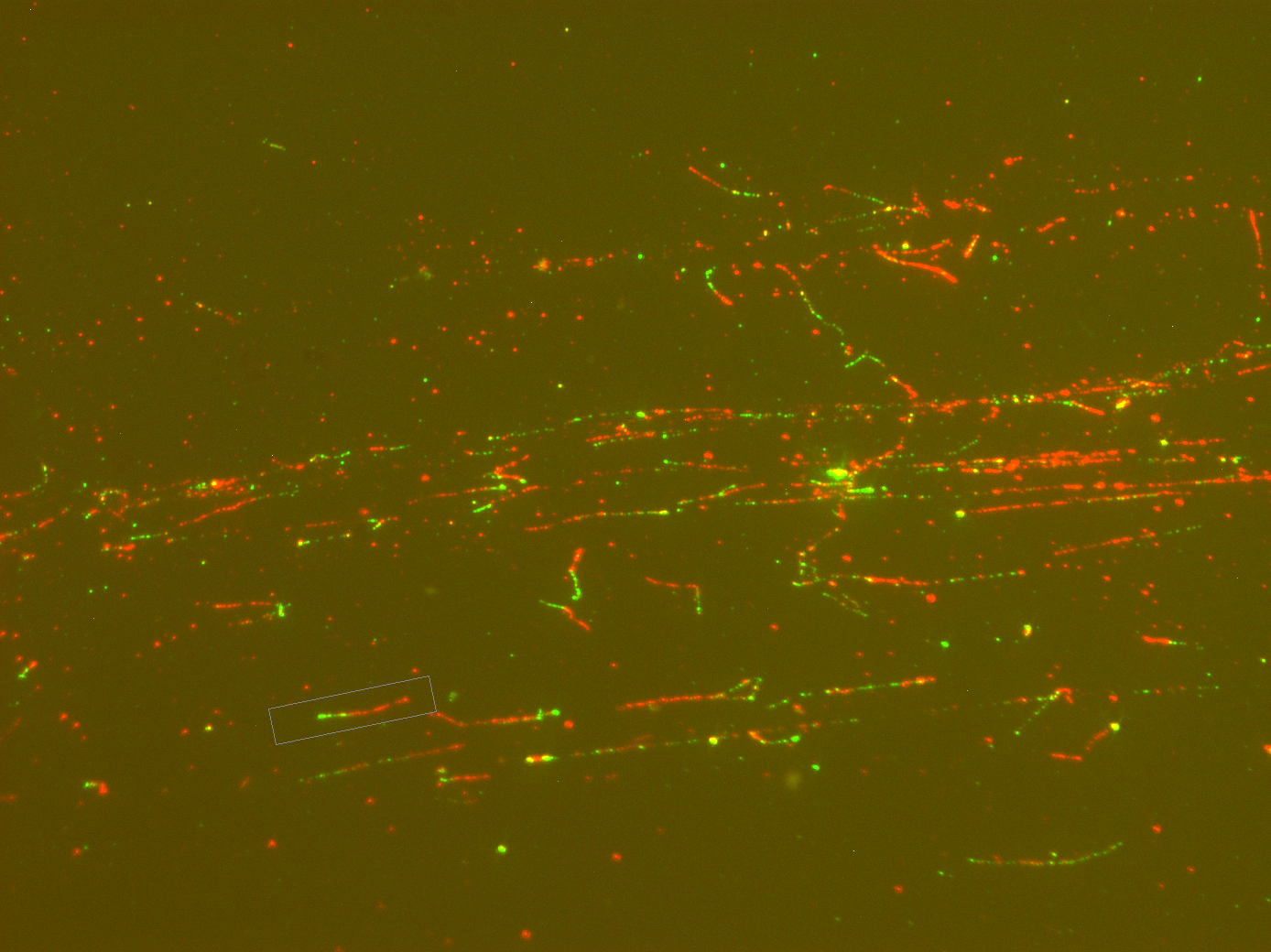

Supplement: Supplementary file 11 — Figure EV2 [file 44319_2025_497_MOESM11_ESM.zip › Figure EV 2H/shRNF20 + shXRCC3 DNA fiber fork degradation.tif]

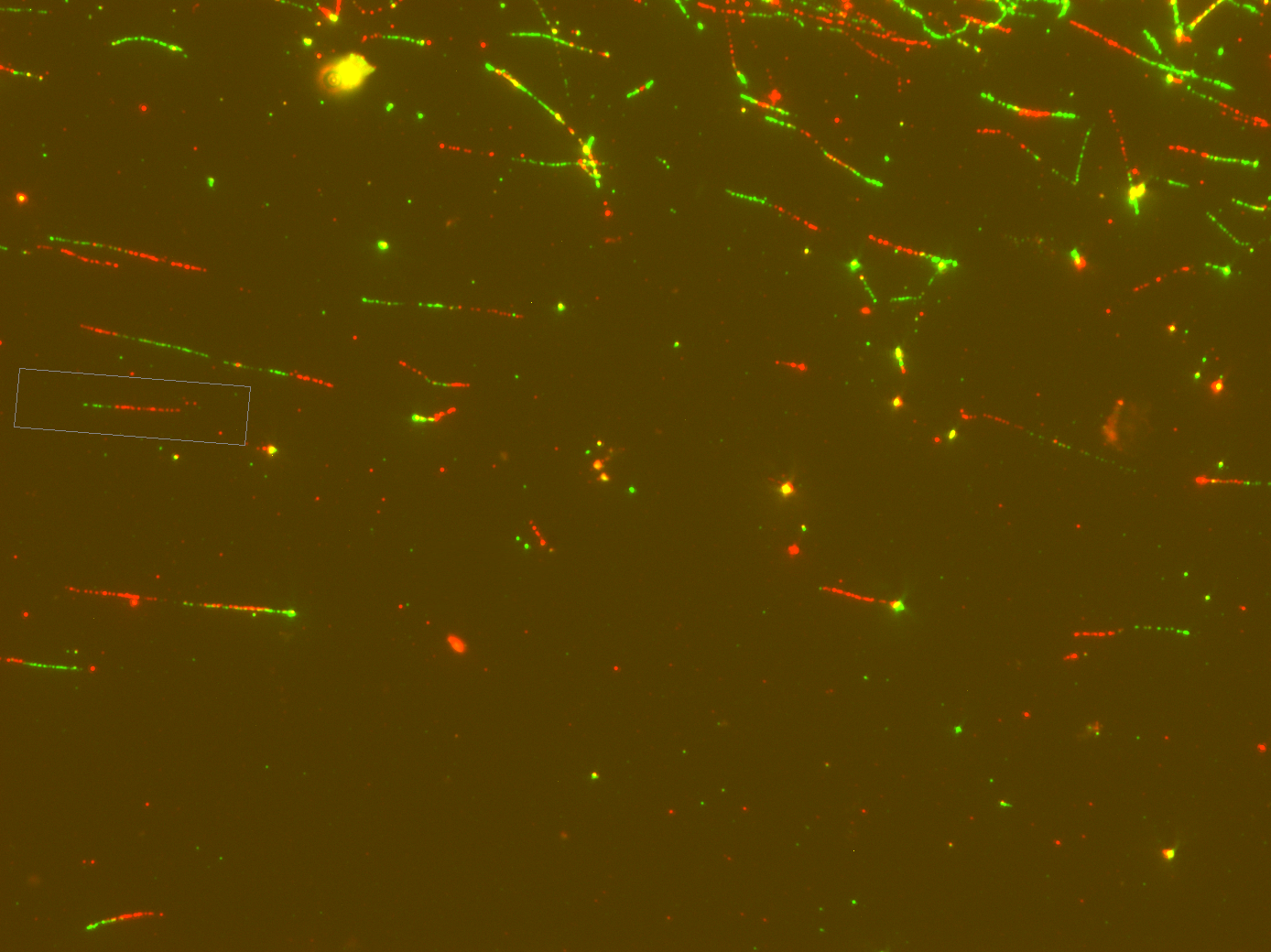

Supplement: Supplementary file 11 — Figure EV2 [file 44319_2025_497_MOESM11_ESM.zip › Figure EV 2H/shRNF20 DNA fiber fork degradation.tif]

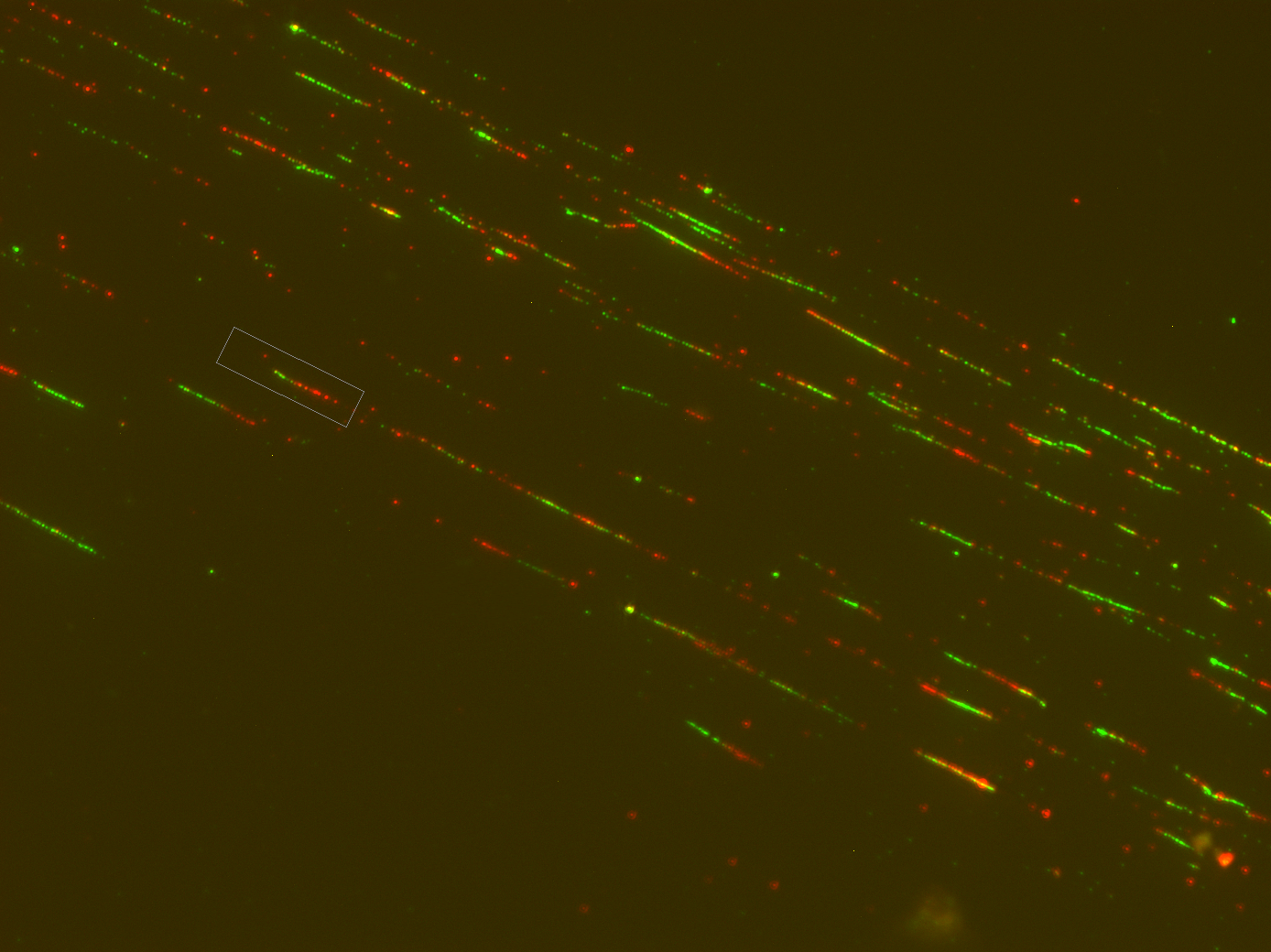

Supplement: Supplementary file 11 — Figure EV2 [file 44319_2025_497_MOESM11_ESM.zip › Figure EV 2H/shRNF20+shRAD51C DNA fiber fork degradation.tif]

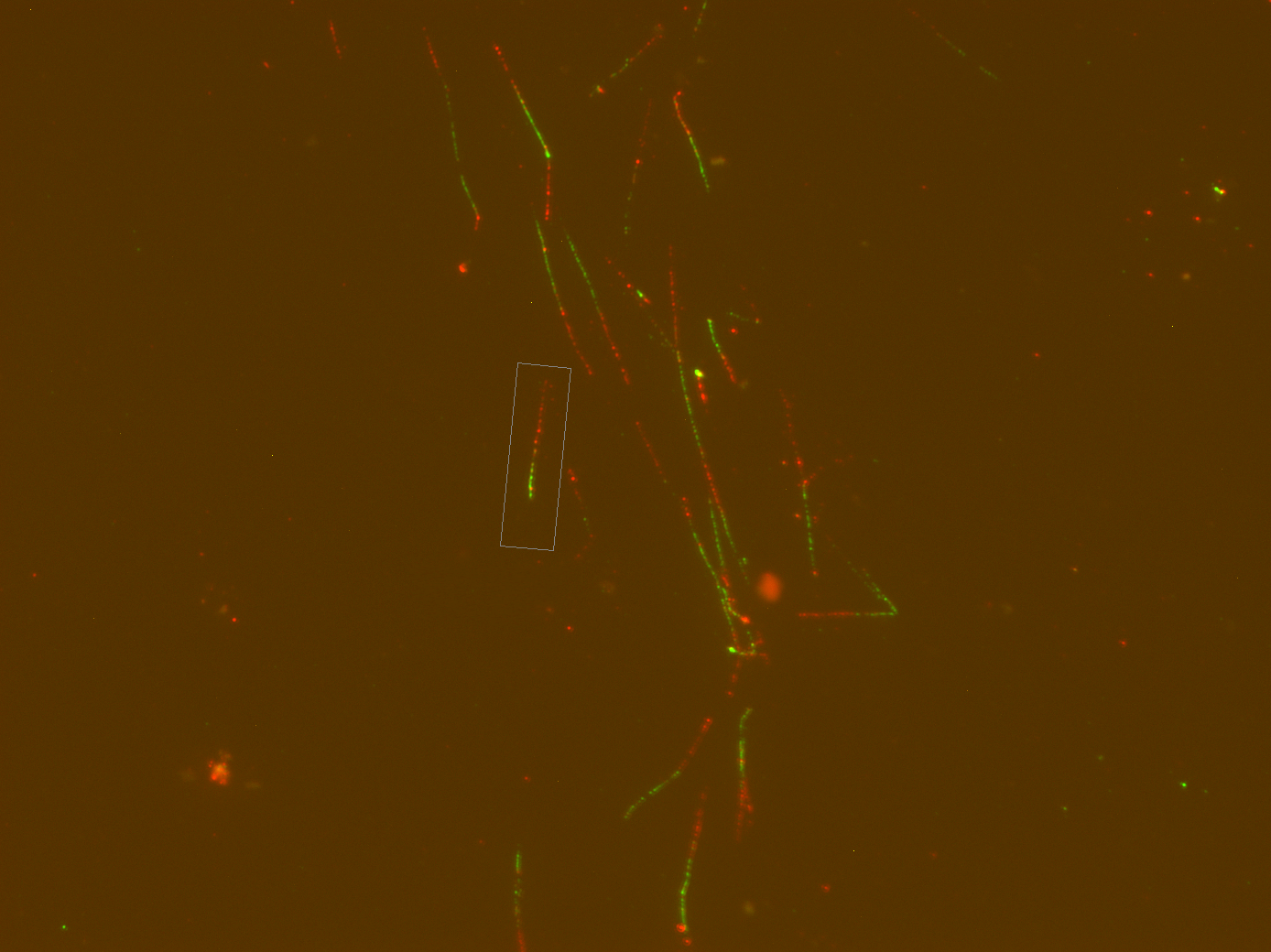

Supplement: Supplementary file 11 — Figure EV2 [file 44319_2025_497_MOESM11_ESM.zip › Figure EV 2H/shXRCC2 DNA fiber fork degradation.tif]

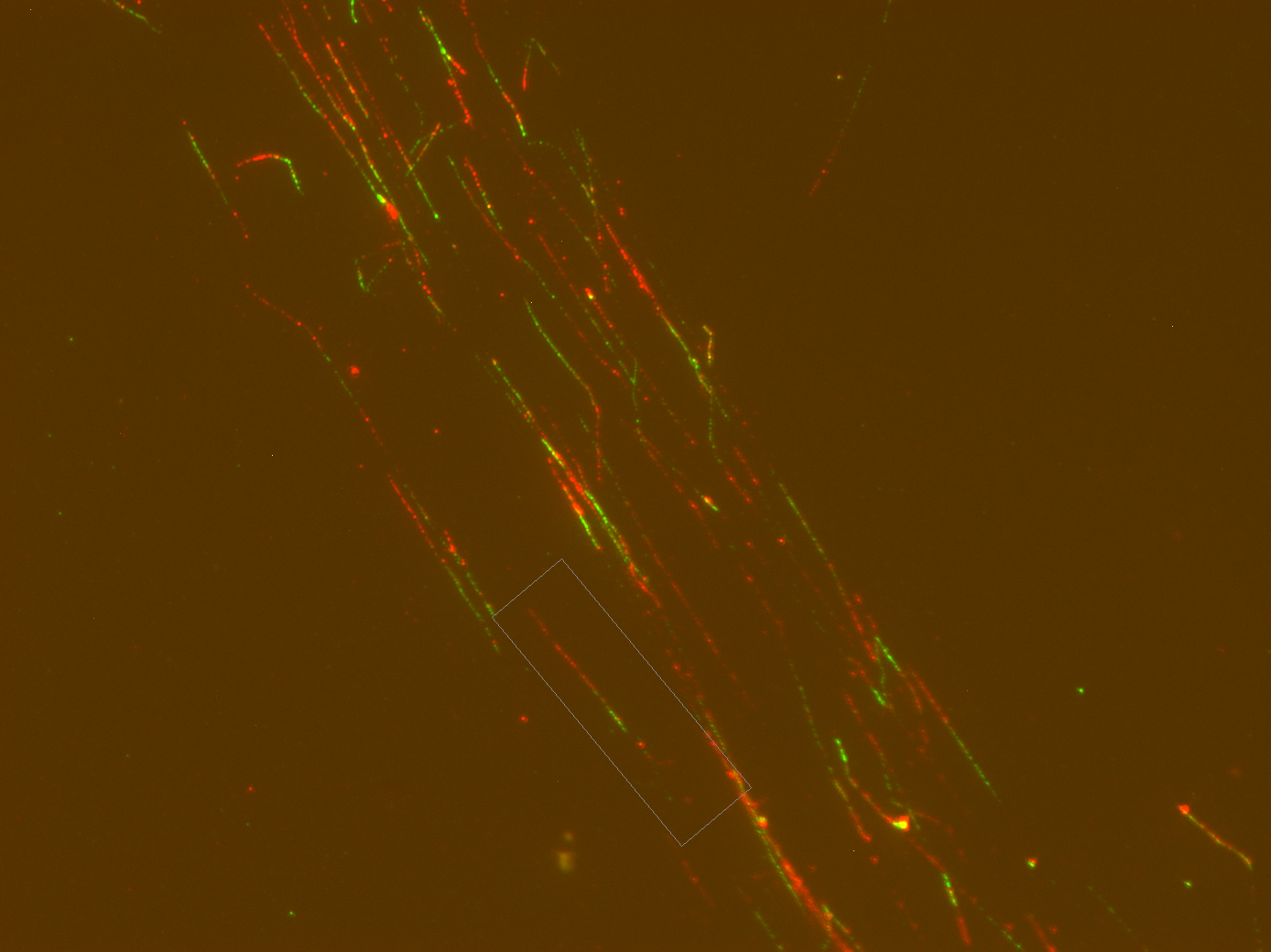

Supplement: Supplementary file 11 — Figure EV2 [file 44319_2025_497_MOESM11_ESM.zip › Figure EV 2H/shXRCC3 DNA fiber fork degradation.tif]

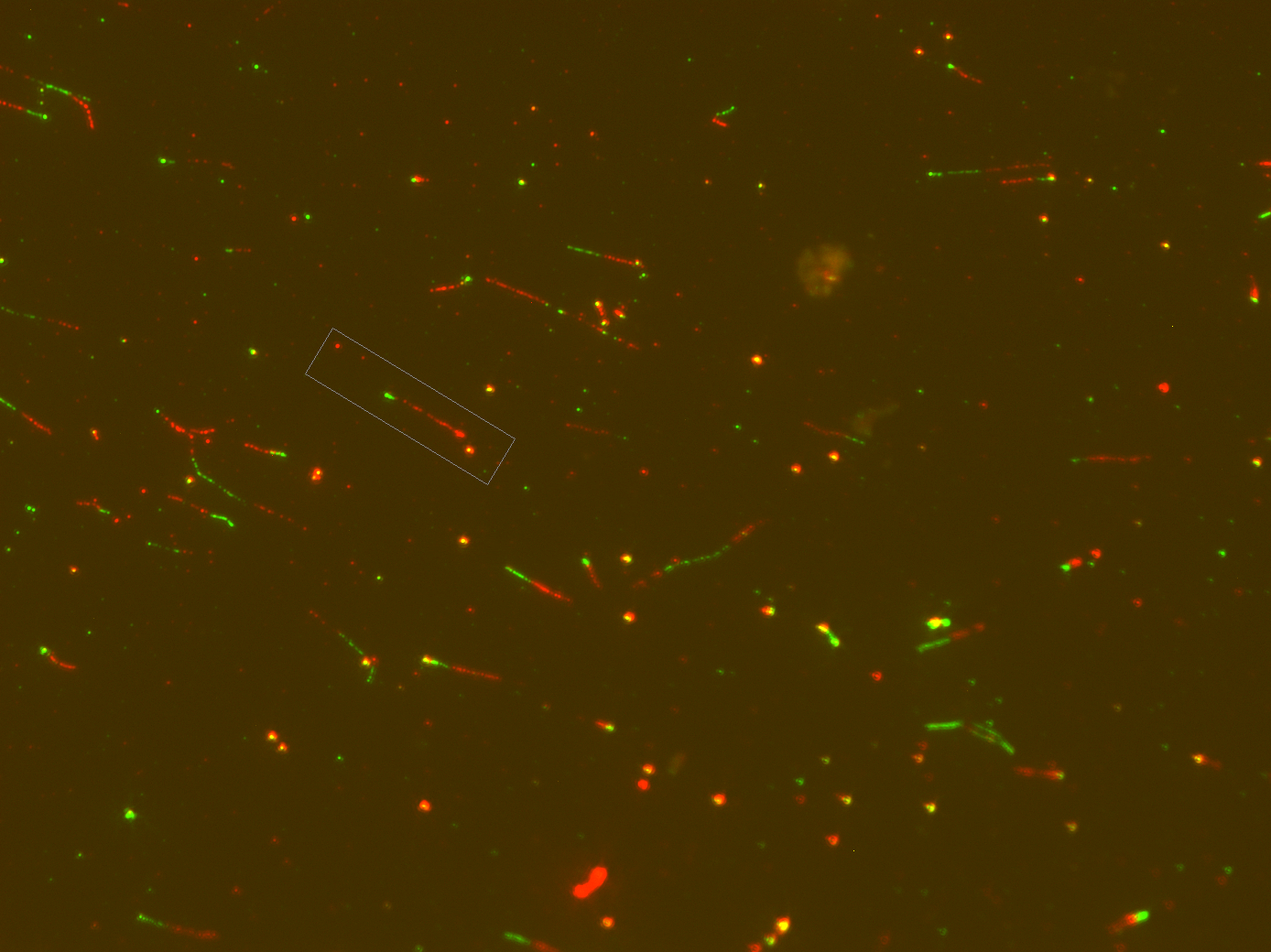

Supplement: Supplementary file 11 — Figure EV2 [file 44319_2025_497_MOESM11_ESM.zip › Figure EV 2J/shRAD51C DNA fiber fork restart.tif]

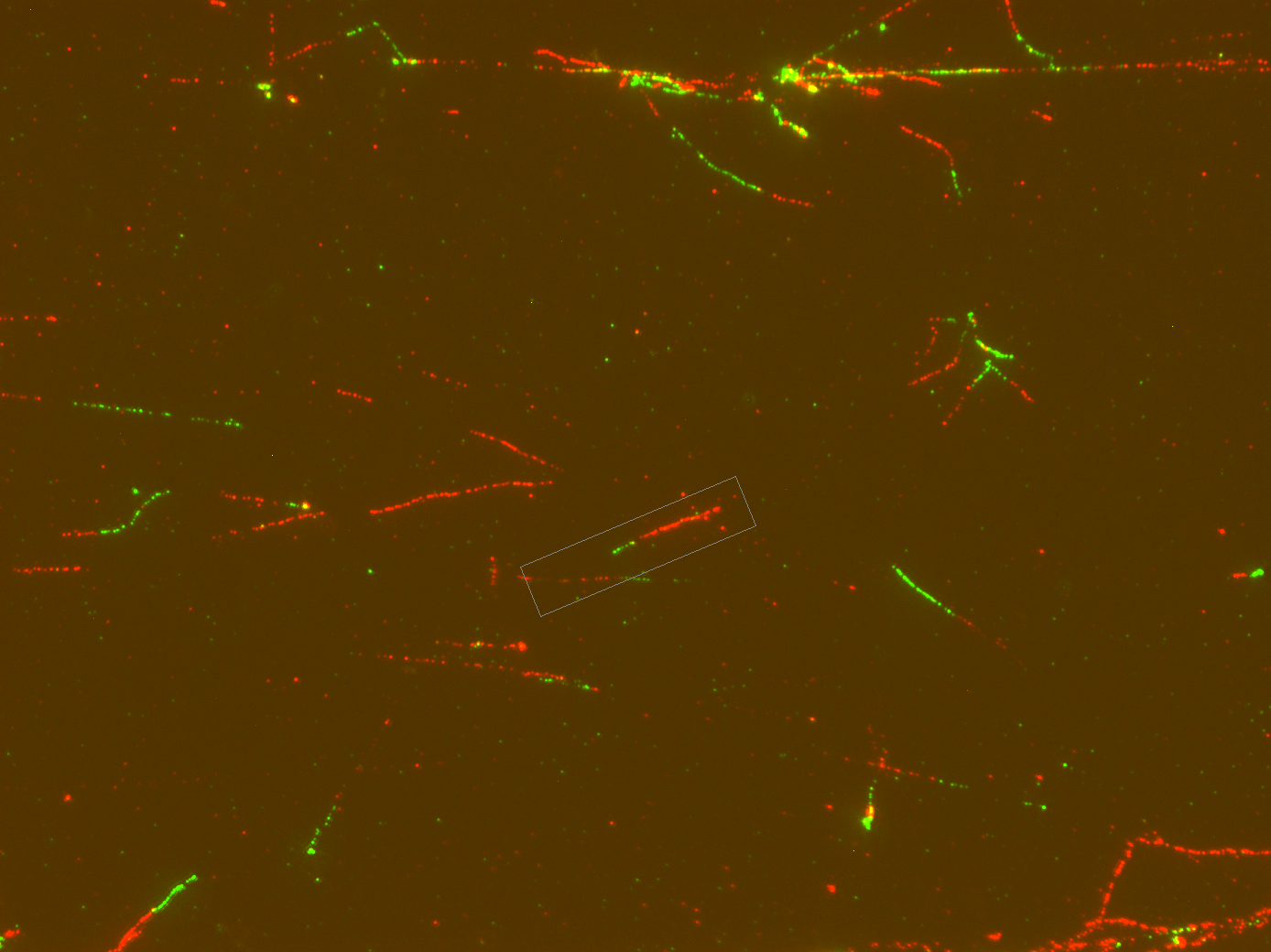

Supplement: Supplementary file 11 — Figure EV2 [file 44319_2025_497_MOESM11_ESM.zip › Figure EV 2J/shRNF20 DNA fiber fork restart.tif]

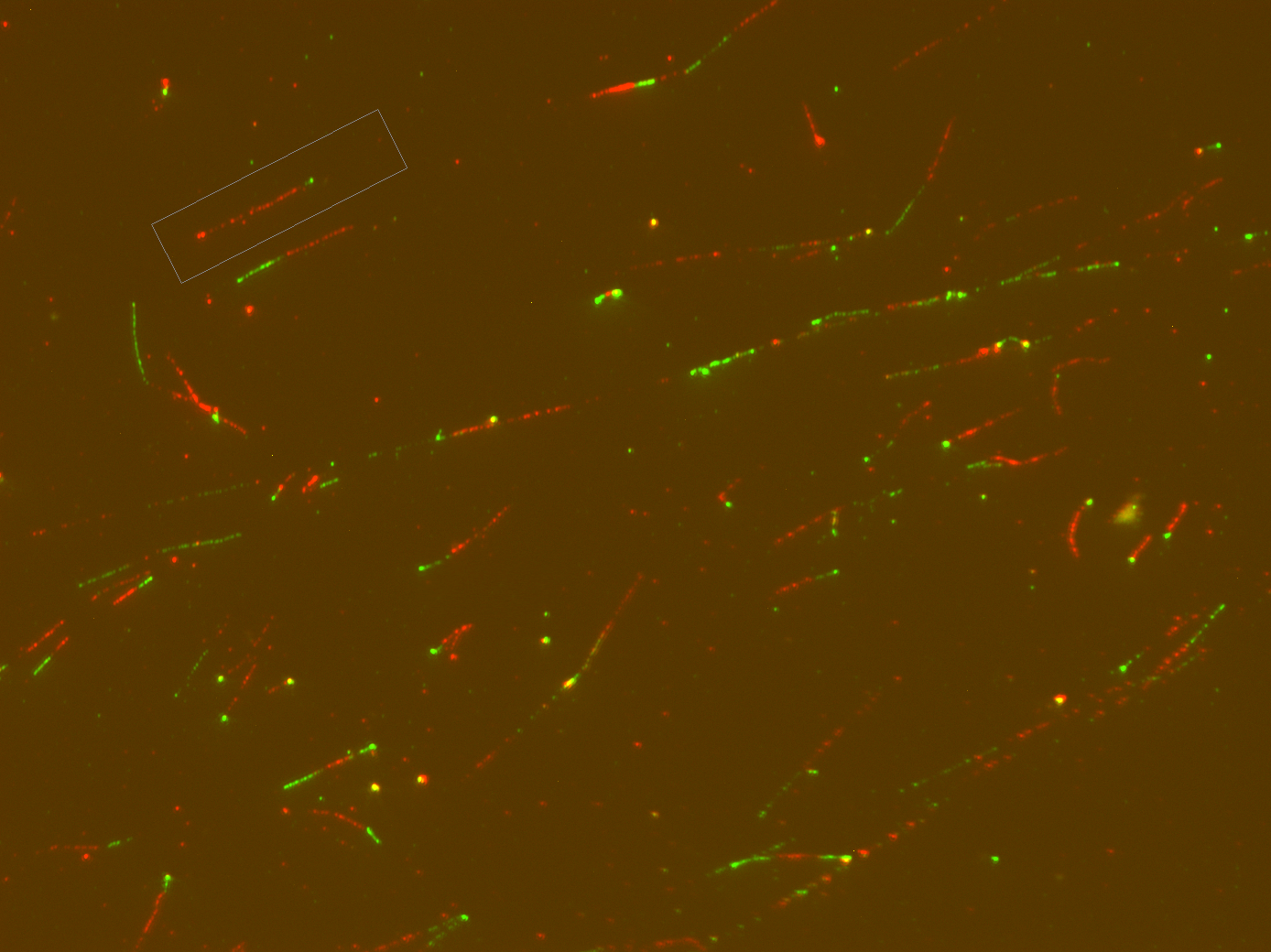

Supplement: Supplementary file 11 — Figure EV2 [file 44319_2025_497_MOESM11_ESM.zip › Figure EV 2J/shRNF20+shRAD51C DNA fiber fork restart.tif]

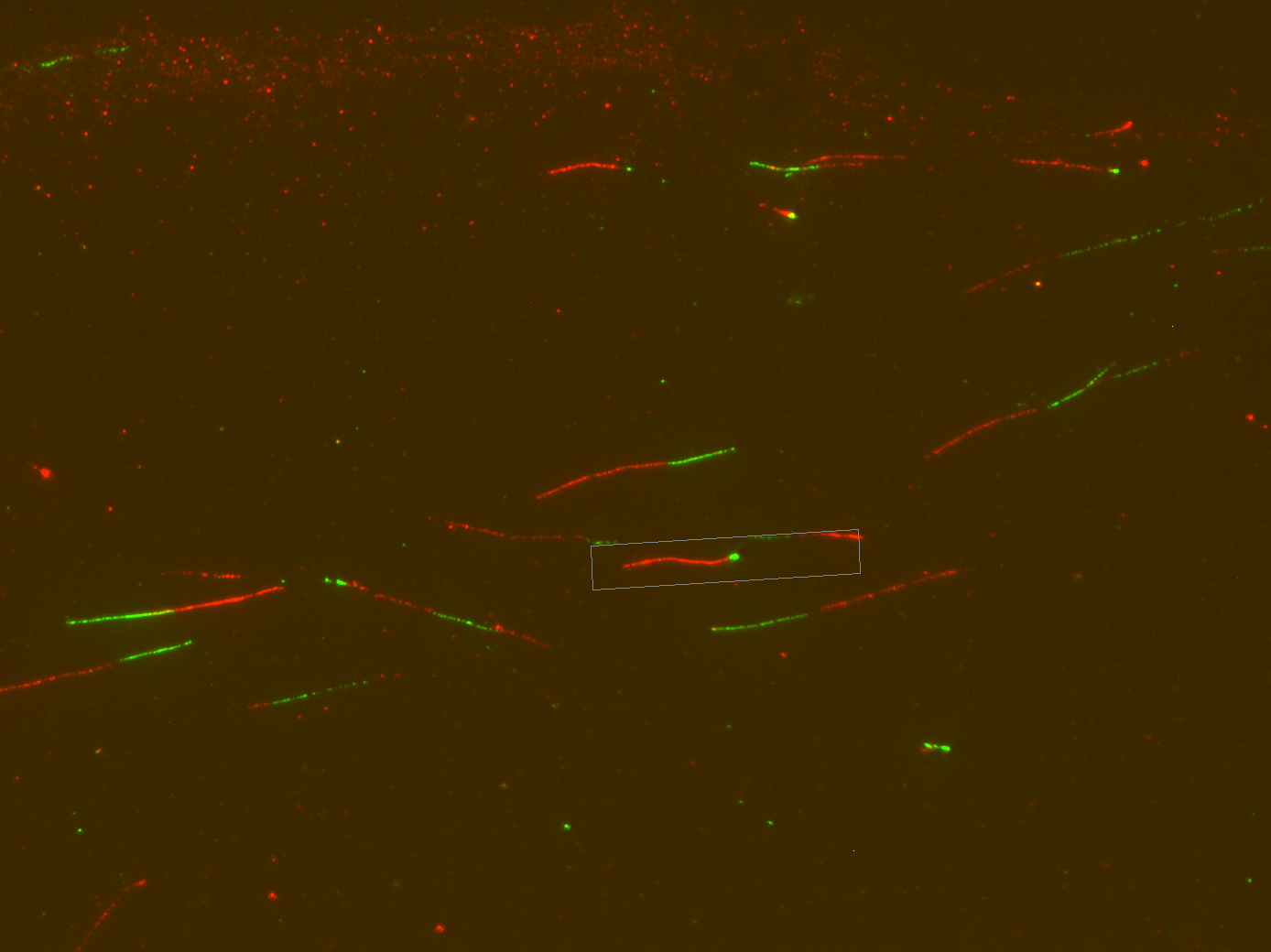

Supplement: Supplementary file 11 — Figure EV2 [file 44319_2025_497_MOESM11_ESM.zip › Figure EV 2J/shRNF20+shXRCC3 DNA fiber fork restart.tif]

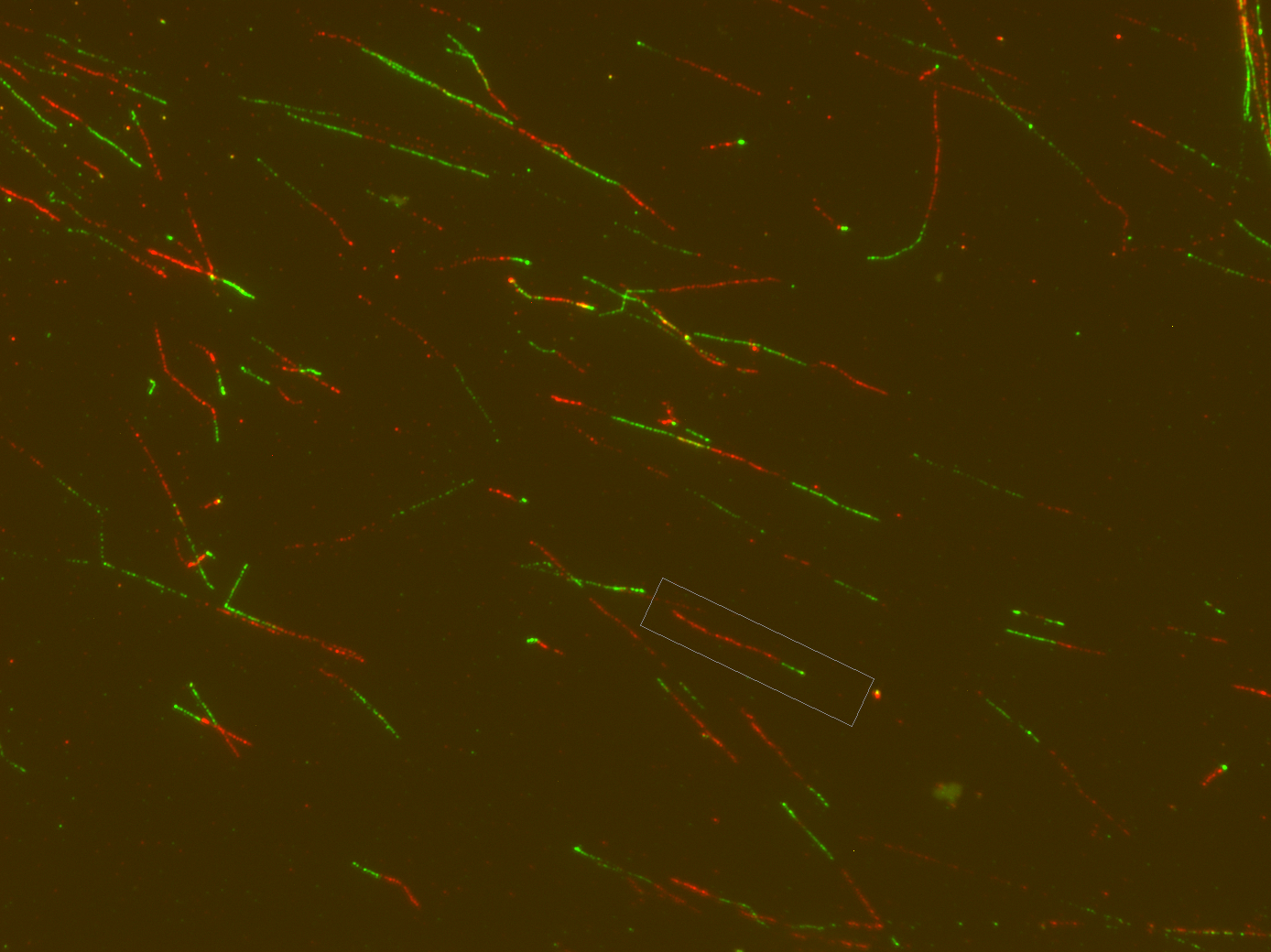

Supplement: Supplementary file 11 — Figure EV2 [file 44319_2025_497_MOESM11_ESM.zip › Figure EV 2J/shXRCC3 DNA fiber fork restart.tif]

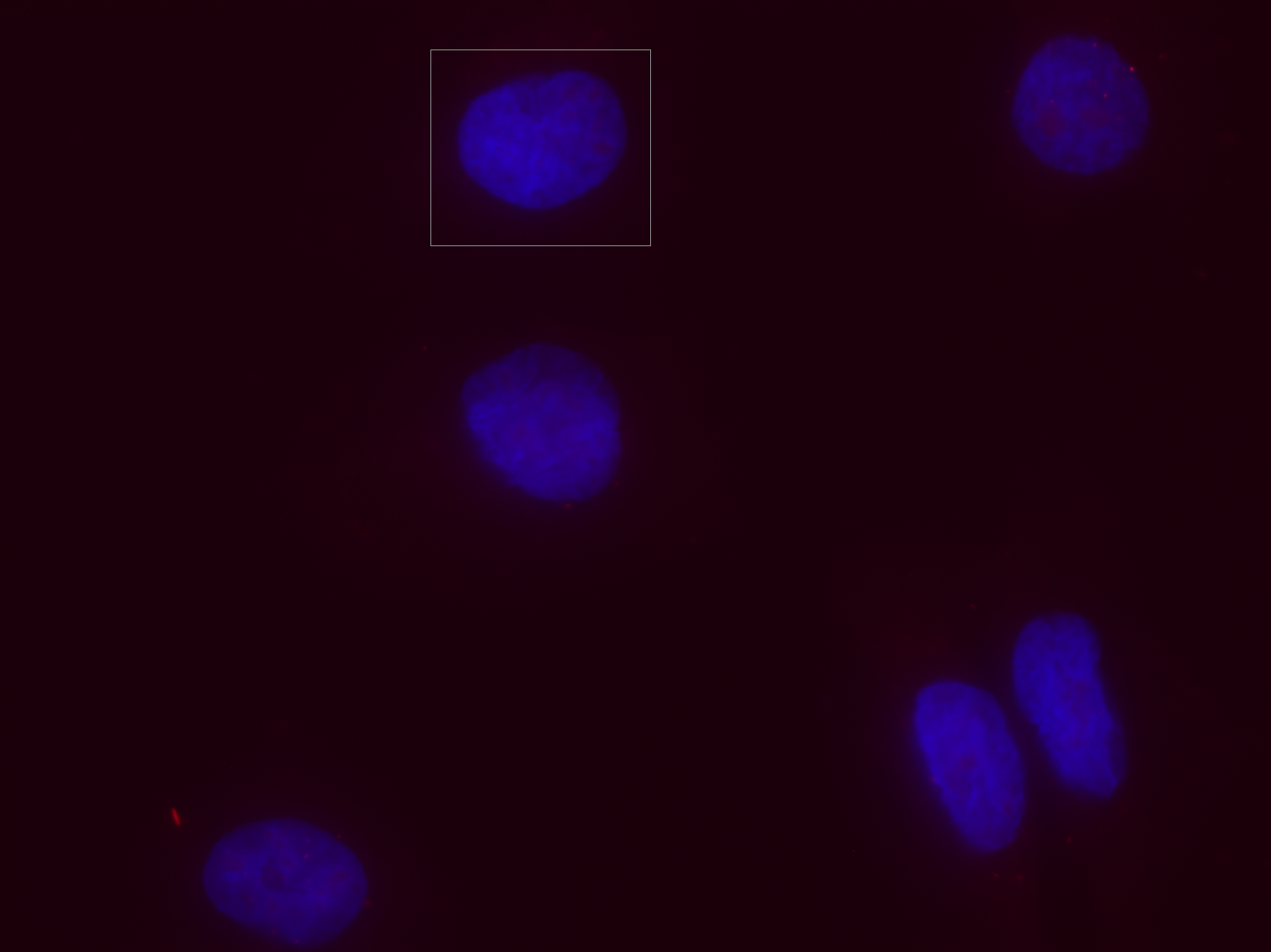

Supplement: Supplementary file 11 — Figure EV2 [file 44319_2025_497_MOESM11_ESM.zip › Figure EV 2L/shControl progressing RNF20 SIRF Only EdU.tif]

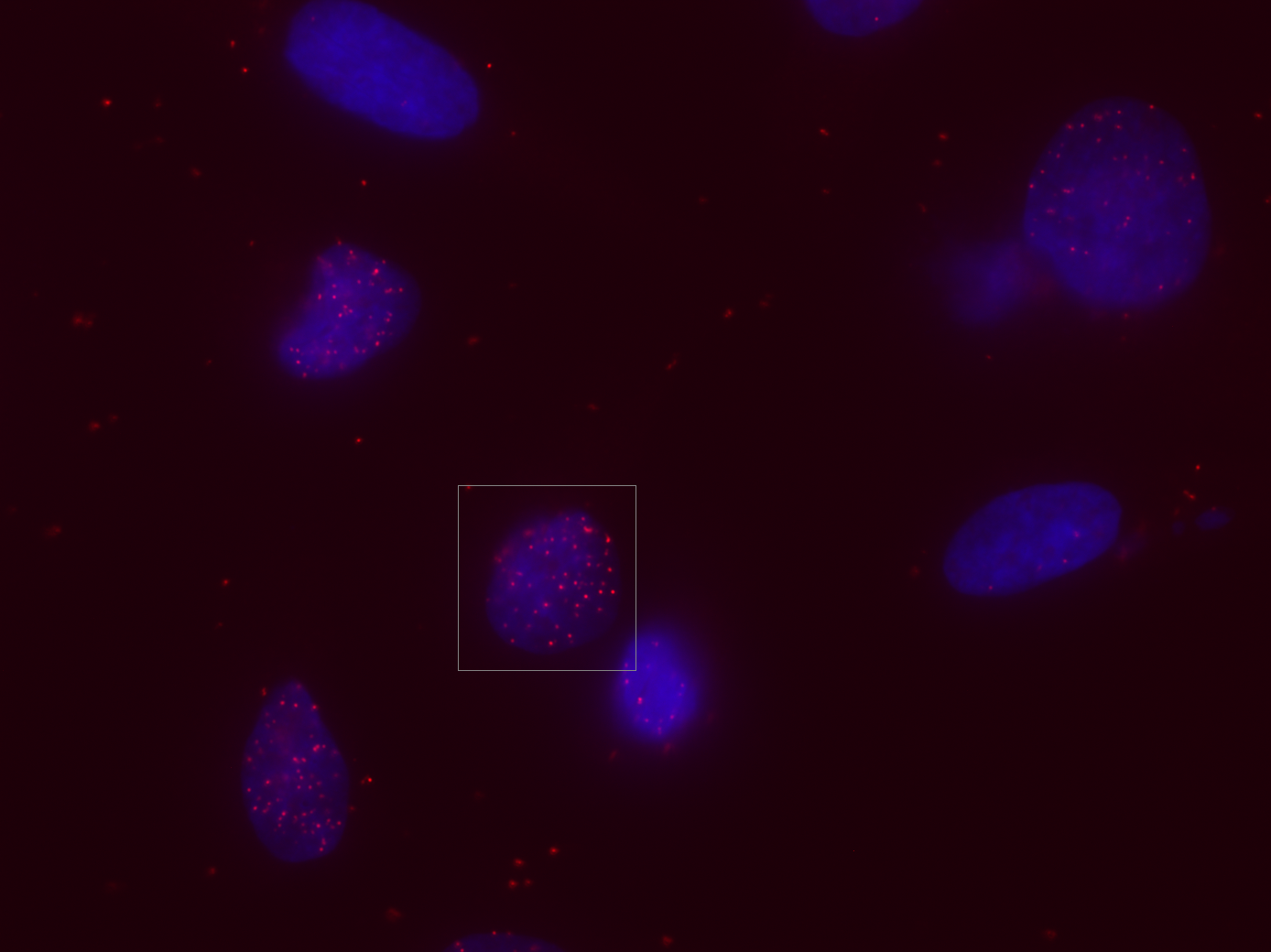

Supplement: Supplementary file 11 — Figure EV2 [file 44319_2025_497_MOESM11_ESM.zip › Figure EV 2L/shControl progressing RNF20 SIRF.tif]

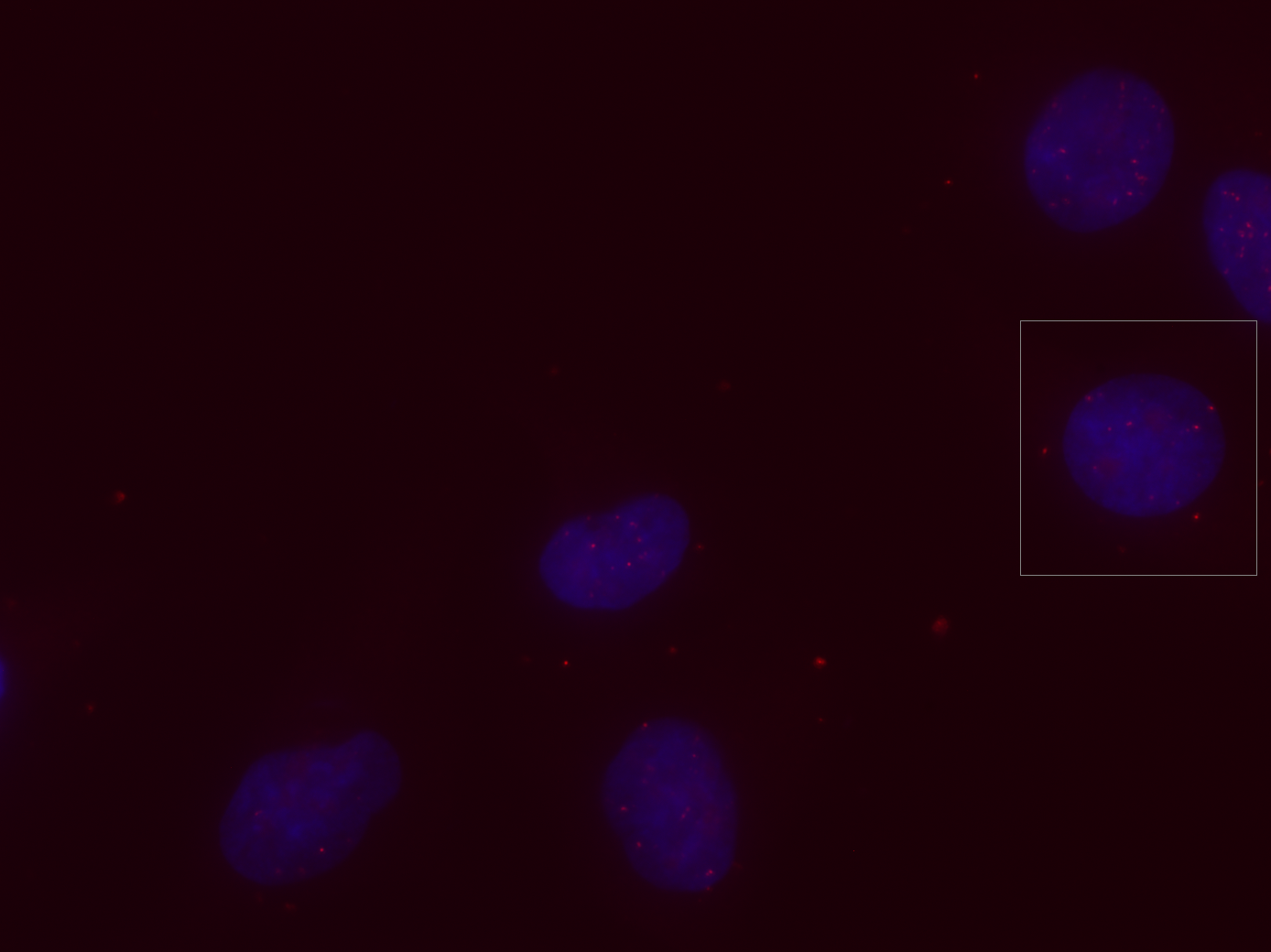

Supplement: Supplementary file 11 — Figure EV2 [file 44319_2025_497_MOESM11_ESM.zip › Figure EV 2L/shControl stall RNF20 SIRF Only EdU.tif]

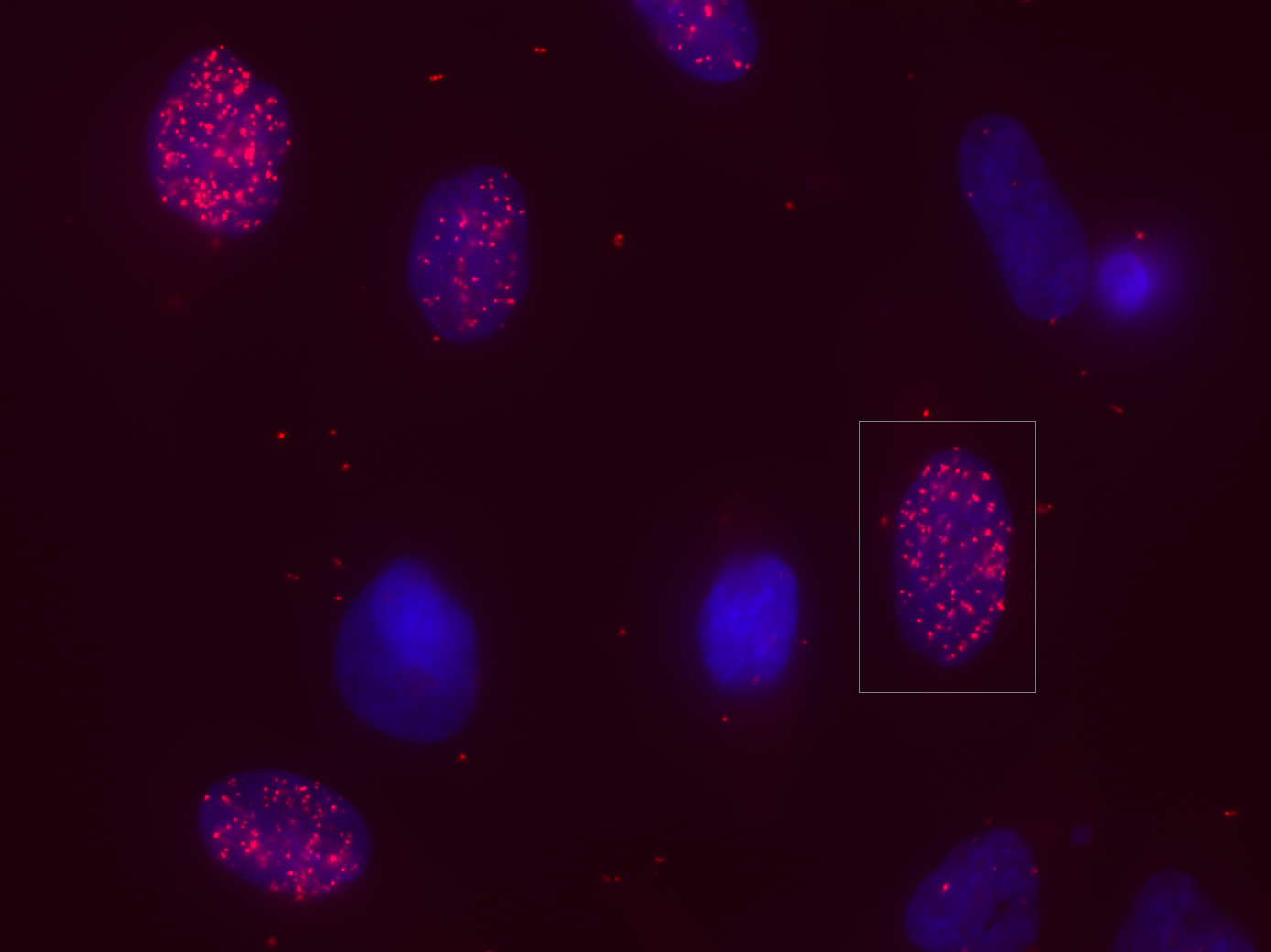

Supplement: Supplementary file 11 — Figure EV2 [file 44319_2025_497_MOESM11_ESM.zip › Figure EV 2L/shControl stall RNF20 SIRF.tif]

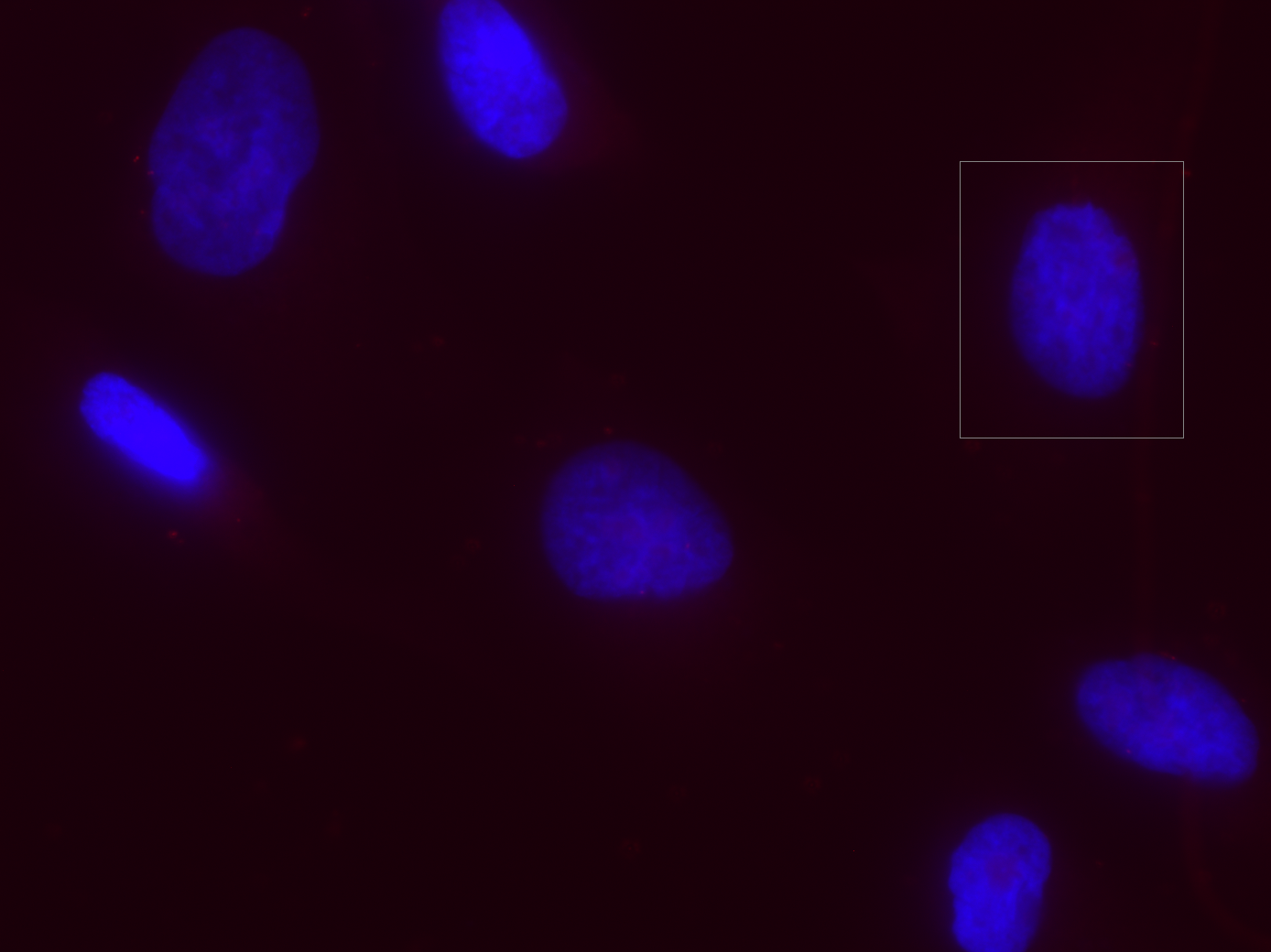

Supplement: Supplementary file 11 — Figure EV2 [file 44319_2025_497_MOESM11_ESM.zip › Figure EV 2L/shRAD51C progressing RNF20 SIRF Only EdU.tif]

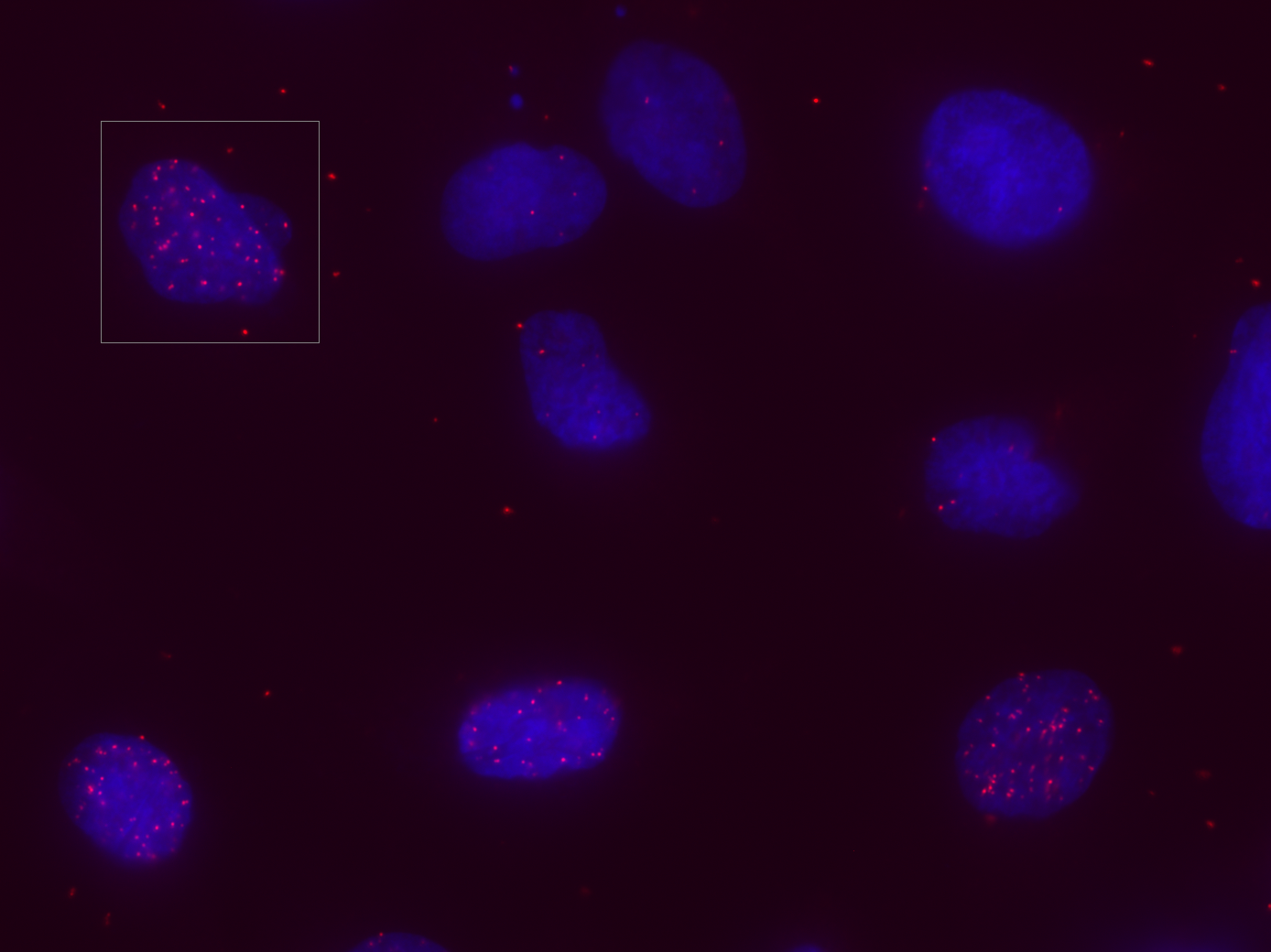

Supplement: Supplementary file 11 — Figure EV2 [file 44319_2025_497_MOESM11_ESM.zip › Figure EV 2L/shRAD51C progressing RNF20 SIRF.tif]

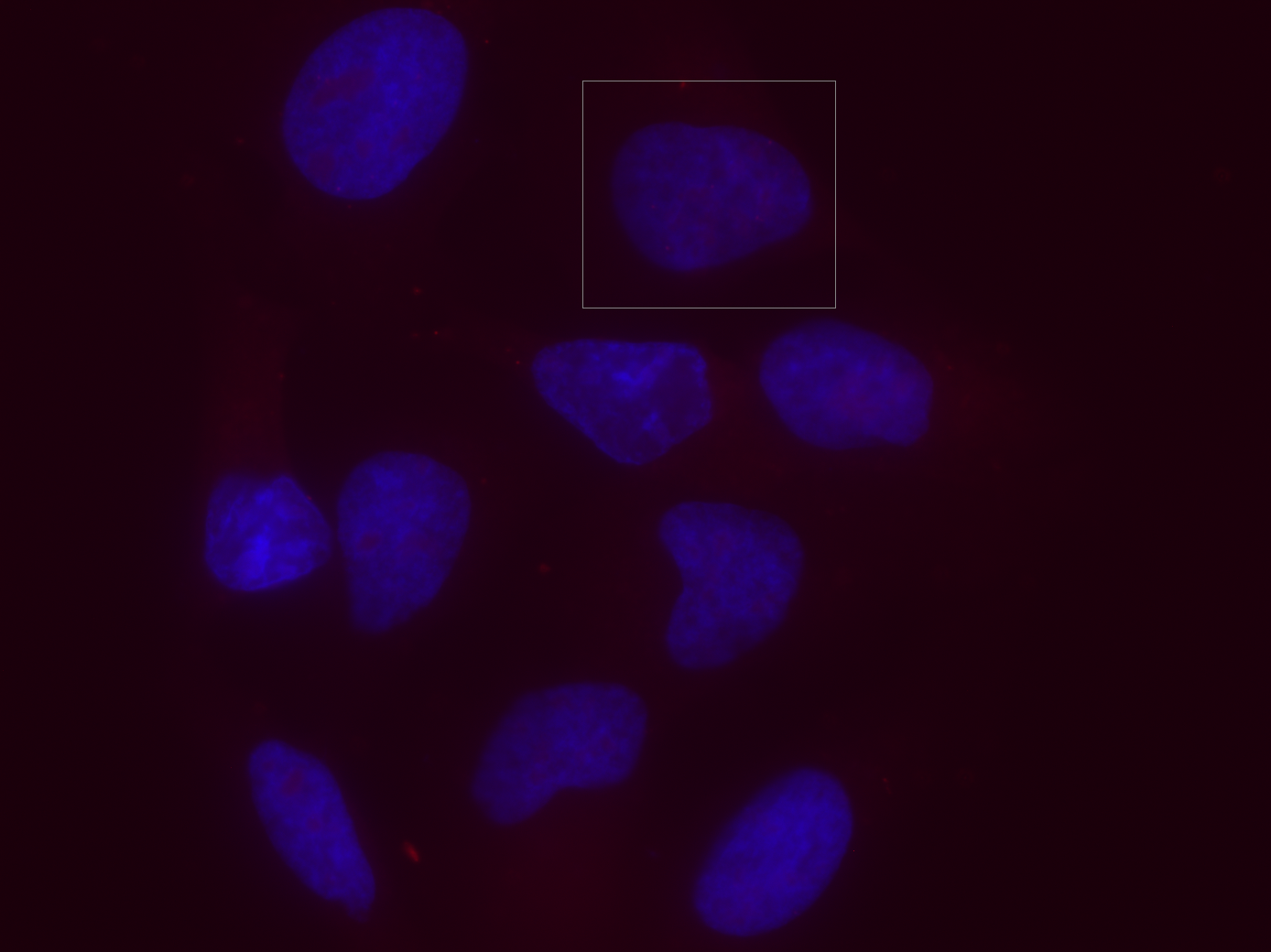

Supplement: Supplementary file 11 — Figure EV2 [file 44319_2025_497_MOESM11_ESM.zip › Figure EV 2L/shRAD51C stall RNF20 SIRF Only EdU.tif]

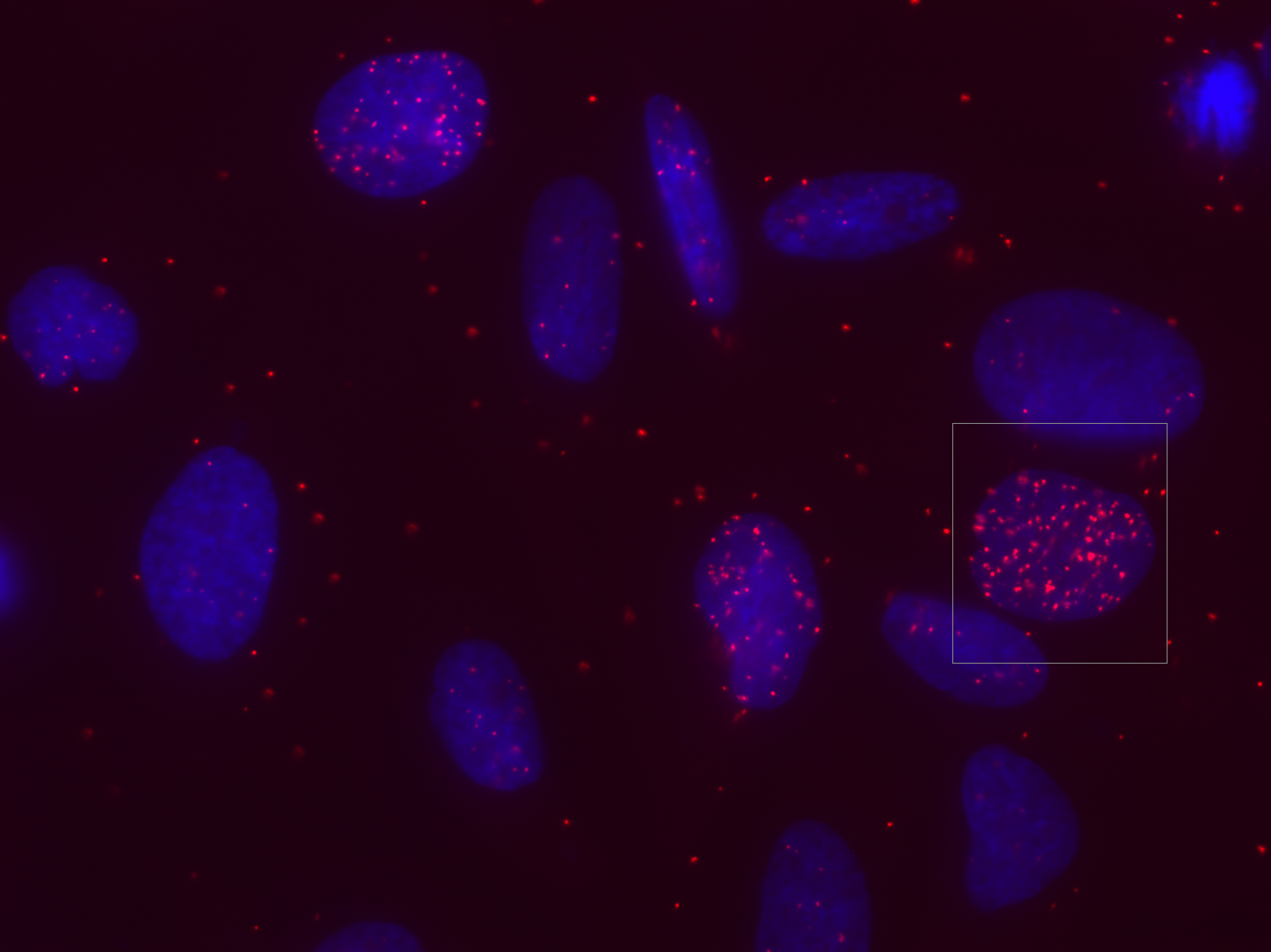

Supplement: Supplementary file 11 — Figure EV2 [file 44319_2025_497_MOESM11_ESM.zip › Figure EV 2L/shRNF20 stall RNF20 SIRF.tif]

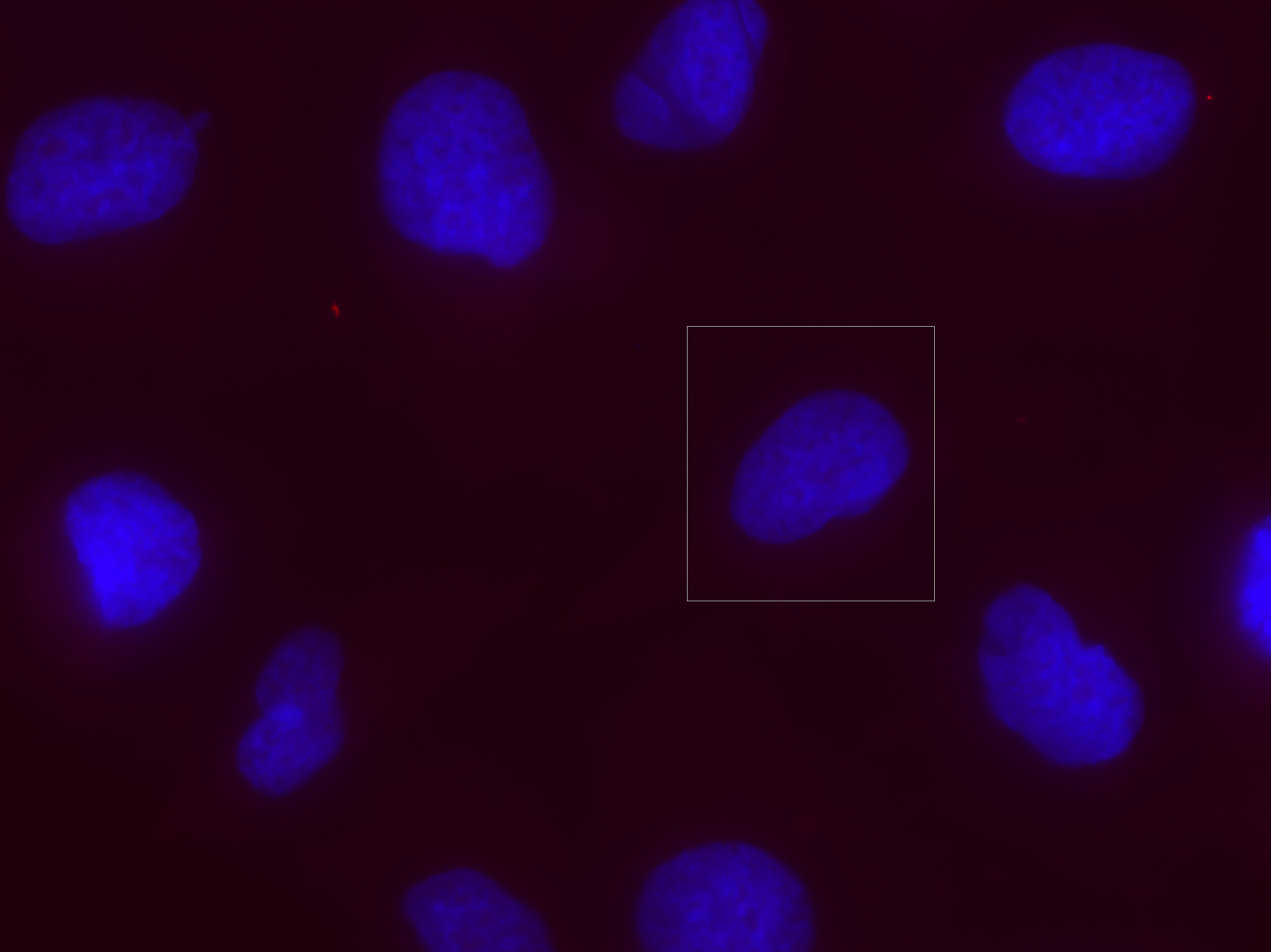

Supplement: Supplementary file 11 — Figure EV2 [file 44319_2025_497_MOESM11_ESM.zip › Figure EV 2M/shControl progressing RNF20 SIRF Only EdU.tif]

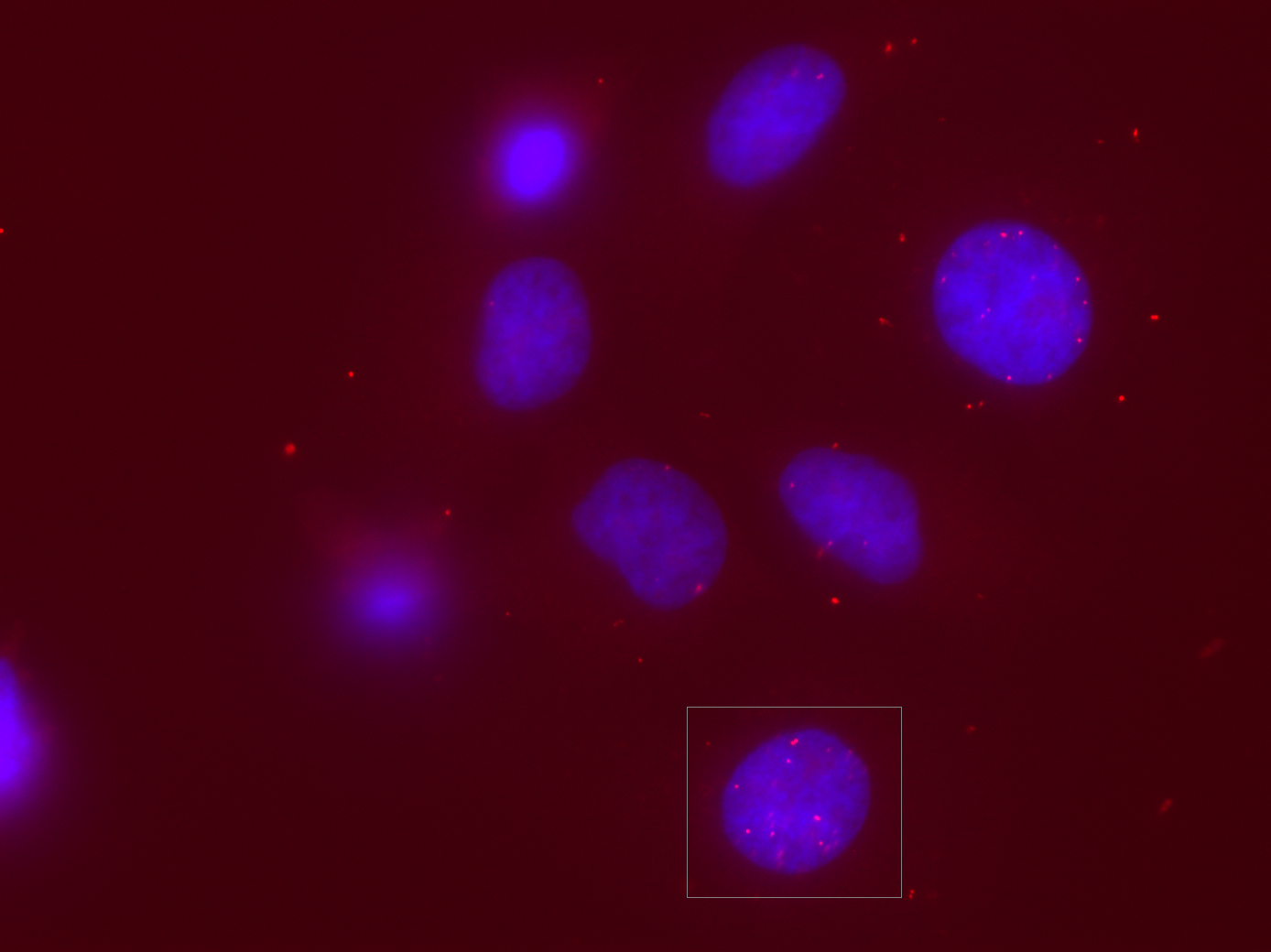

Supplement: Supplementary file 11 — Figure EV2 [file 44319_2025_497_MOESM11_ESM.zip › Figure EV 2M/shControl progressing RNF20 SIRF.tif]

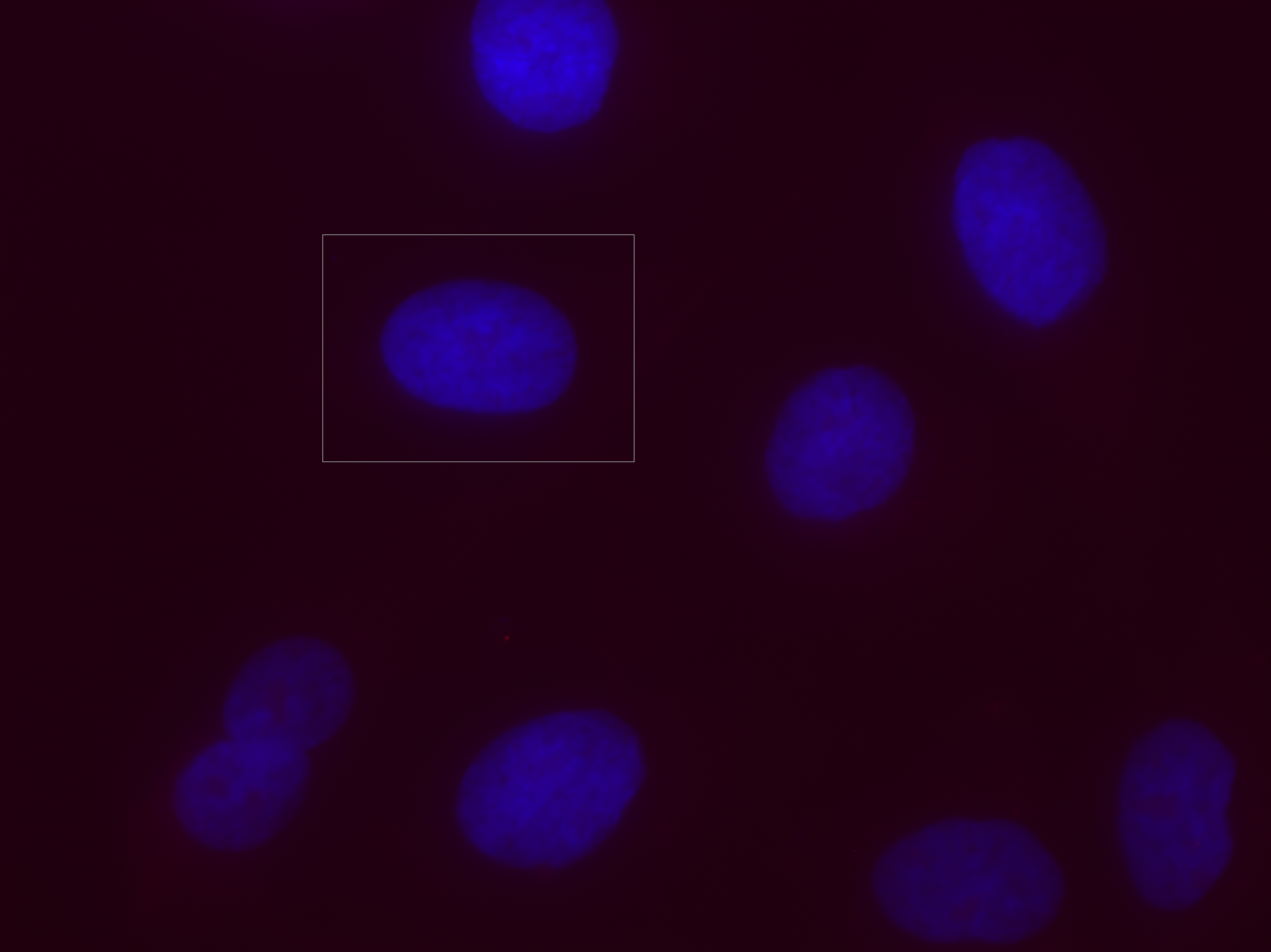

Supplement: Supplementary file 11 — Figure EV2 [file 44319_2025_497_MOESM11_ESM.zip › Figure EV 2M/shControl stall RNF20 SIRF Only EdU.tif]

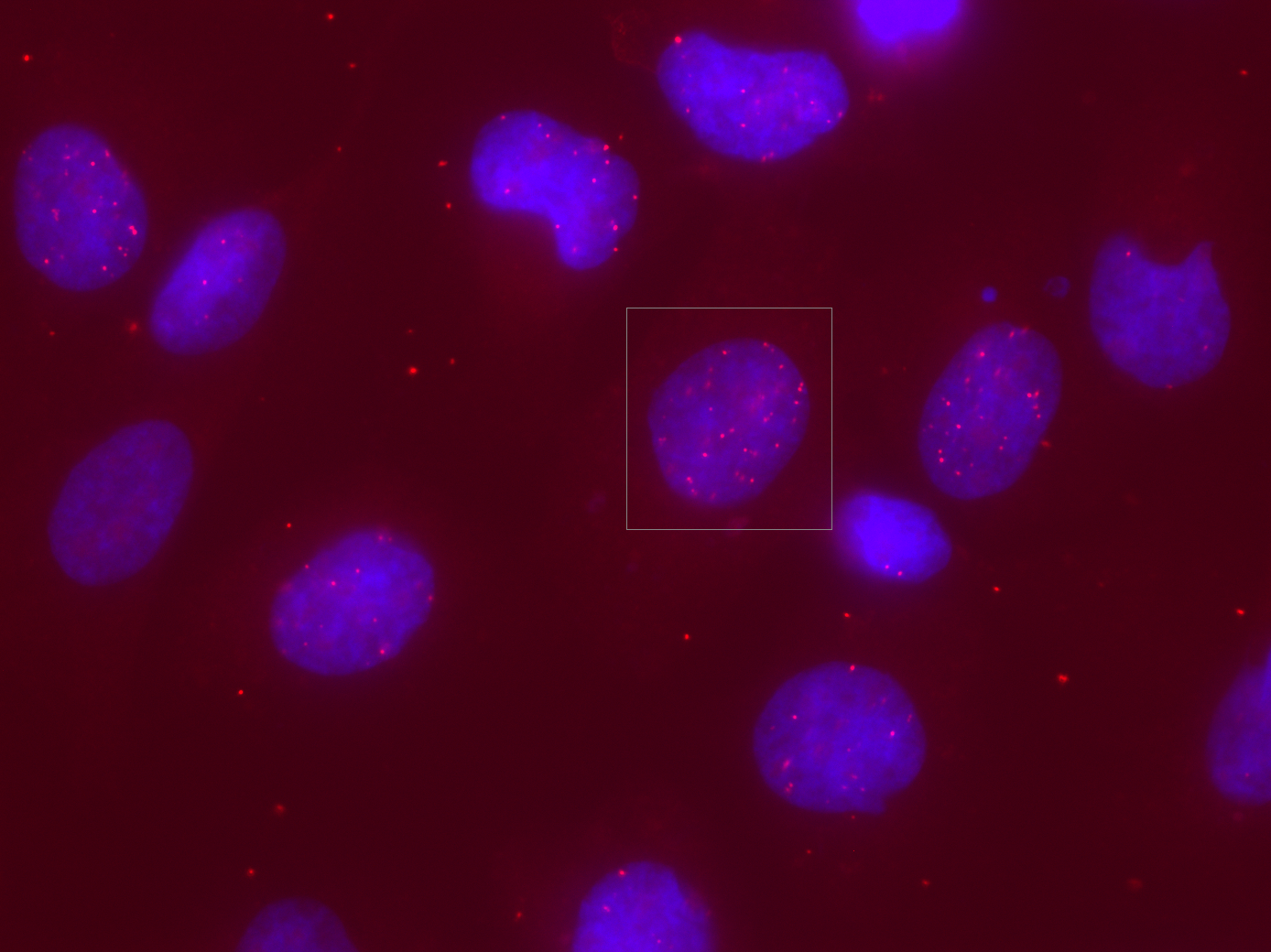

Supplement: Supplementary file 11 — Figure EV2 [file 44319_2025_497_MOESM11_ESM.zip › Figure EV 2M/shControl stall RNF20 SIRF.tif]

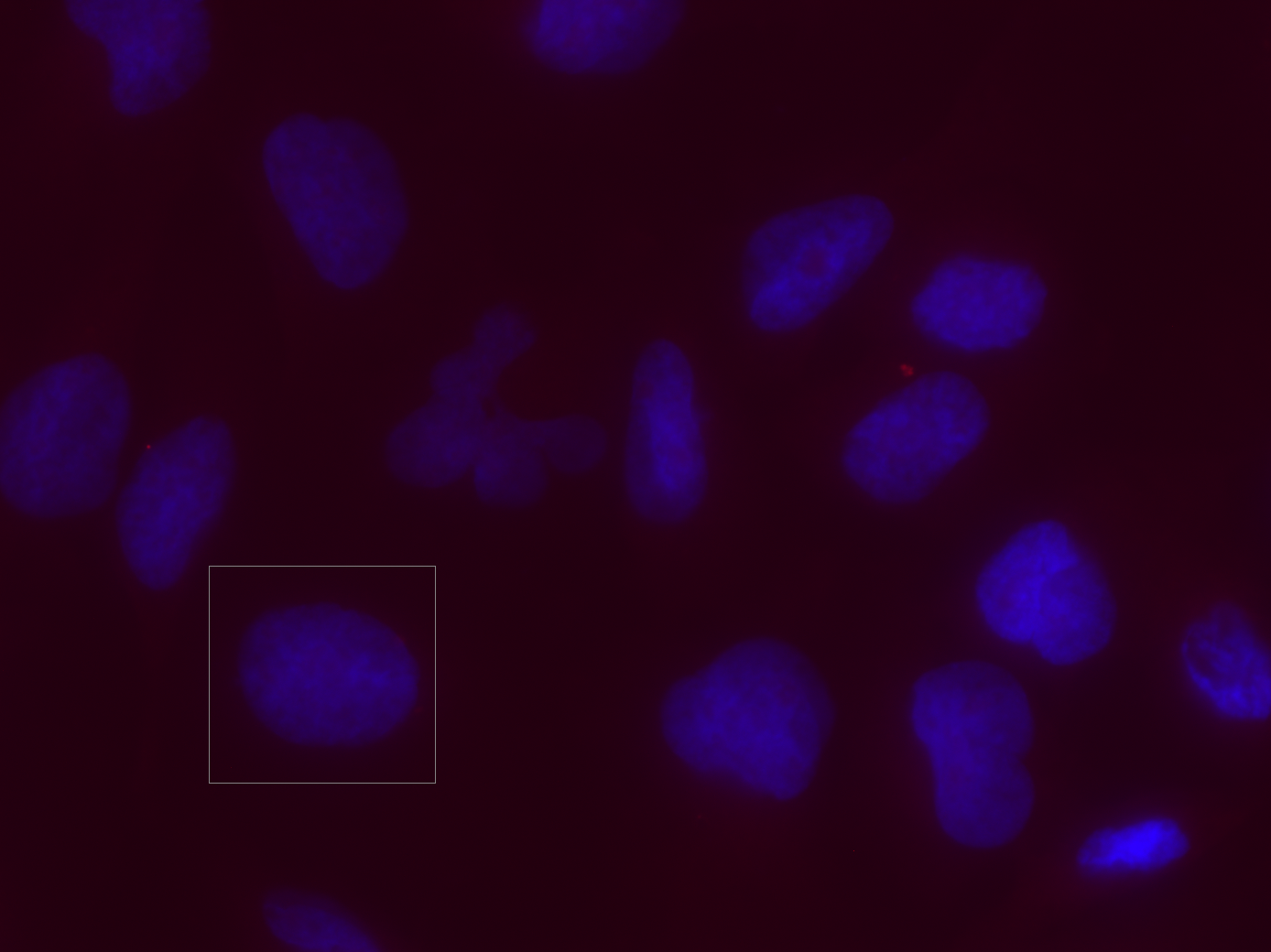

Supplement: Supplementary file 11 — Figure EV2 [file 44319_2025_497_MOESM11_ESM.zip › Figure EV 2M/shRAD51 progressing RNF20 SIRF Only EdU.tif]

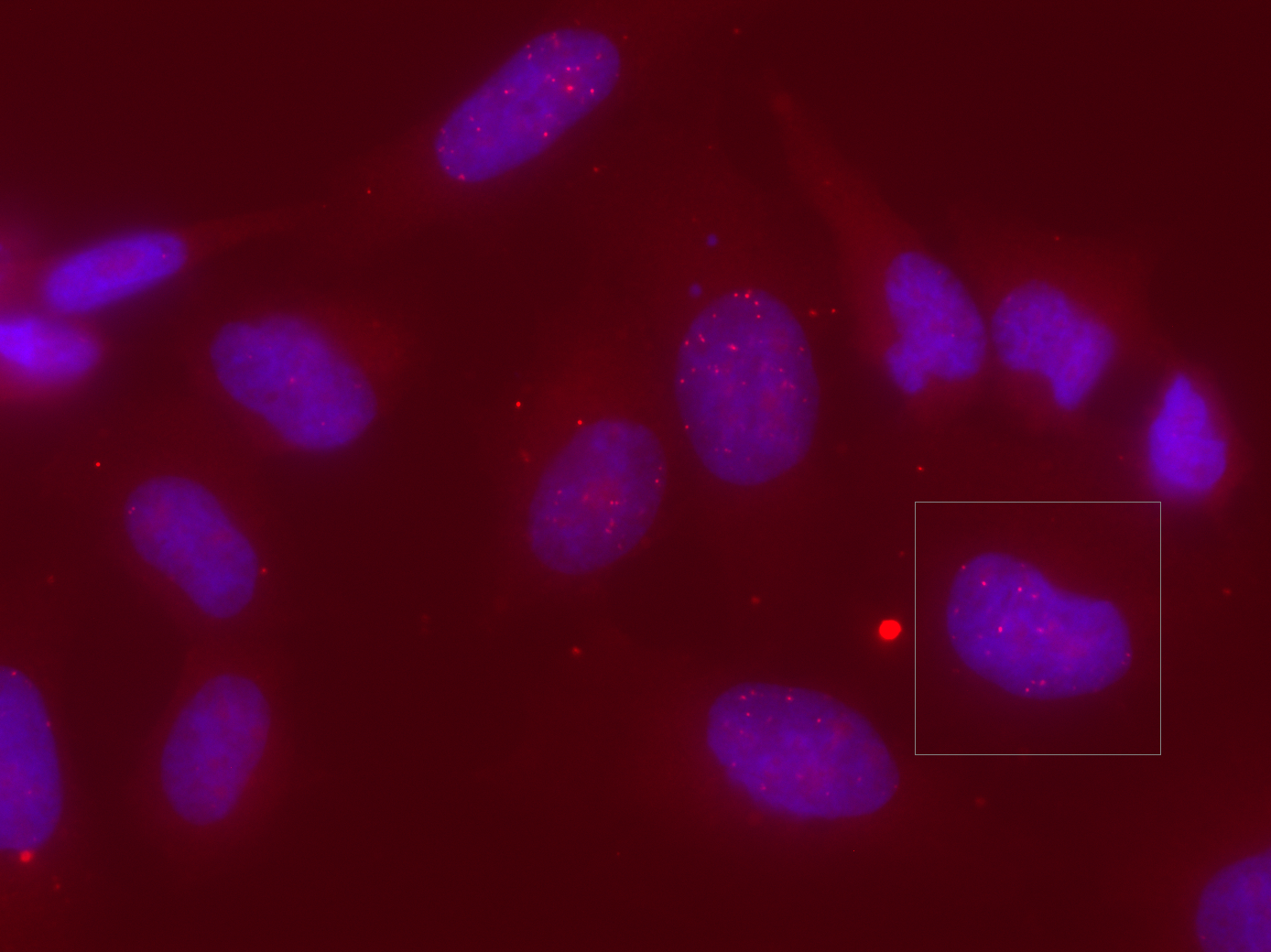

Supplement: Supplementary file 11 — Figure EV2 [file 44319_2025_497_MOESM11_ESM.zip › Figure EV 2M/shRAD51 progressing RNF20 SIRF.tif]

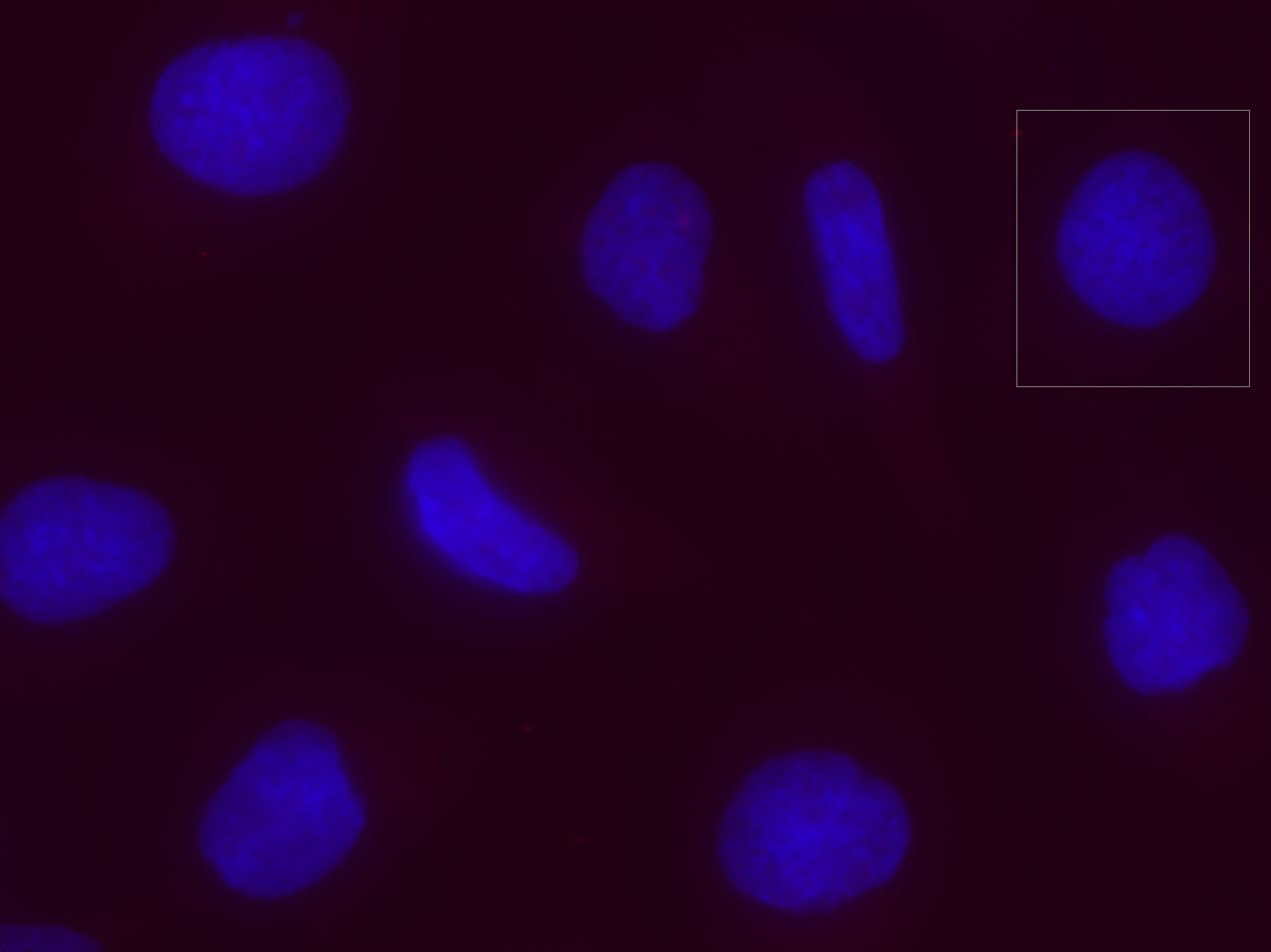

Supplement: Supplementary file 11 — Figure EV2 [file 44319_2025_497_MOESM11_ESM.zip › Figure EV 2M/shRAD51 stall RNF20 SIRF Only EdU.tif]

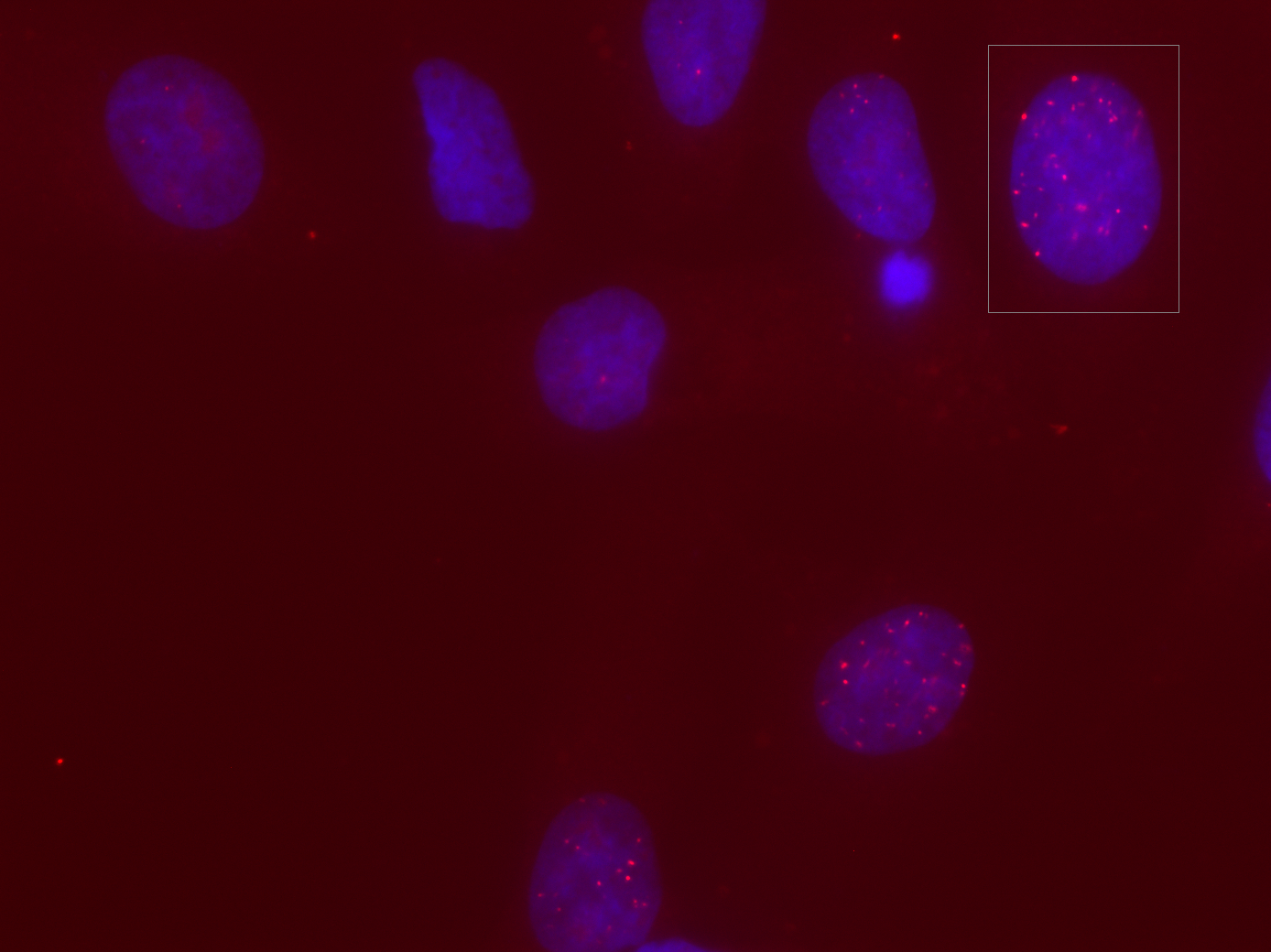

Supplement: Supplementary file 11 — Figure EV2 [file 44319_2025_497_MOESM11_ESM.zip › Figure EV 2M/shRAD51 stall RNF20 SIRF.tif]

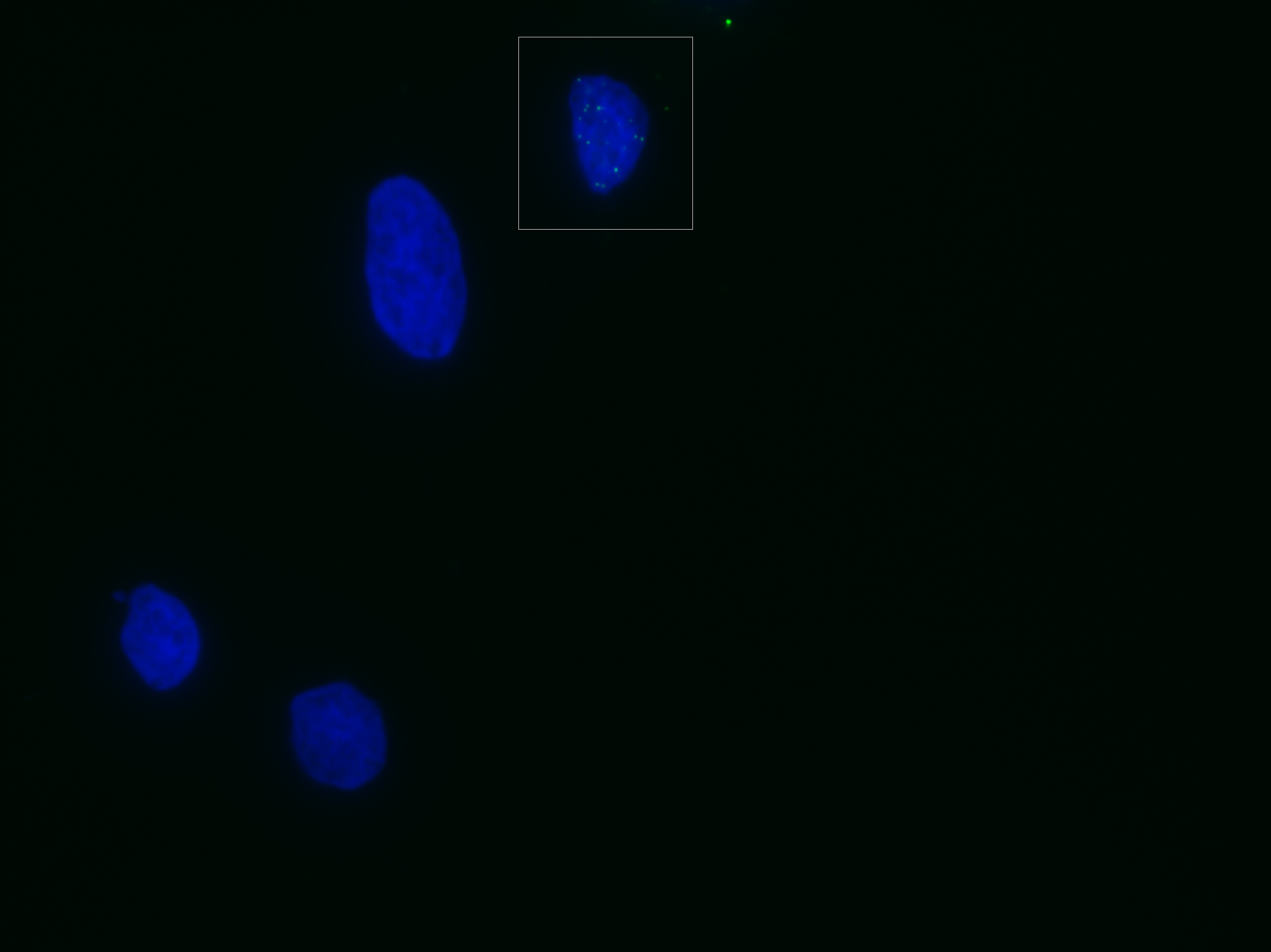

Supplement: Supplementary file 12 — Figure EV3 [file 44319_2025_497_MOESM12_ESM.zip › Figure EV 3A/shControl IF Flag-RNF20 foci.tif]

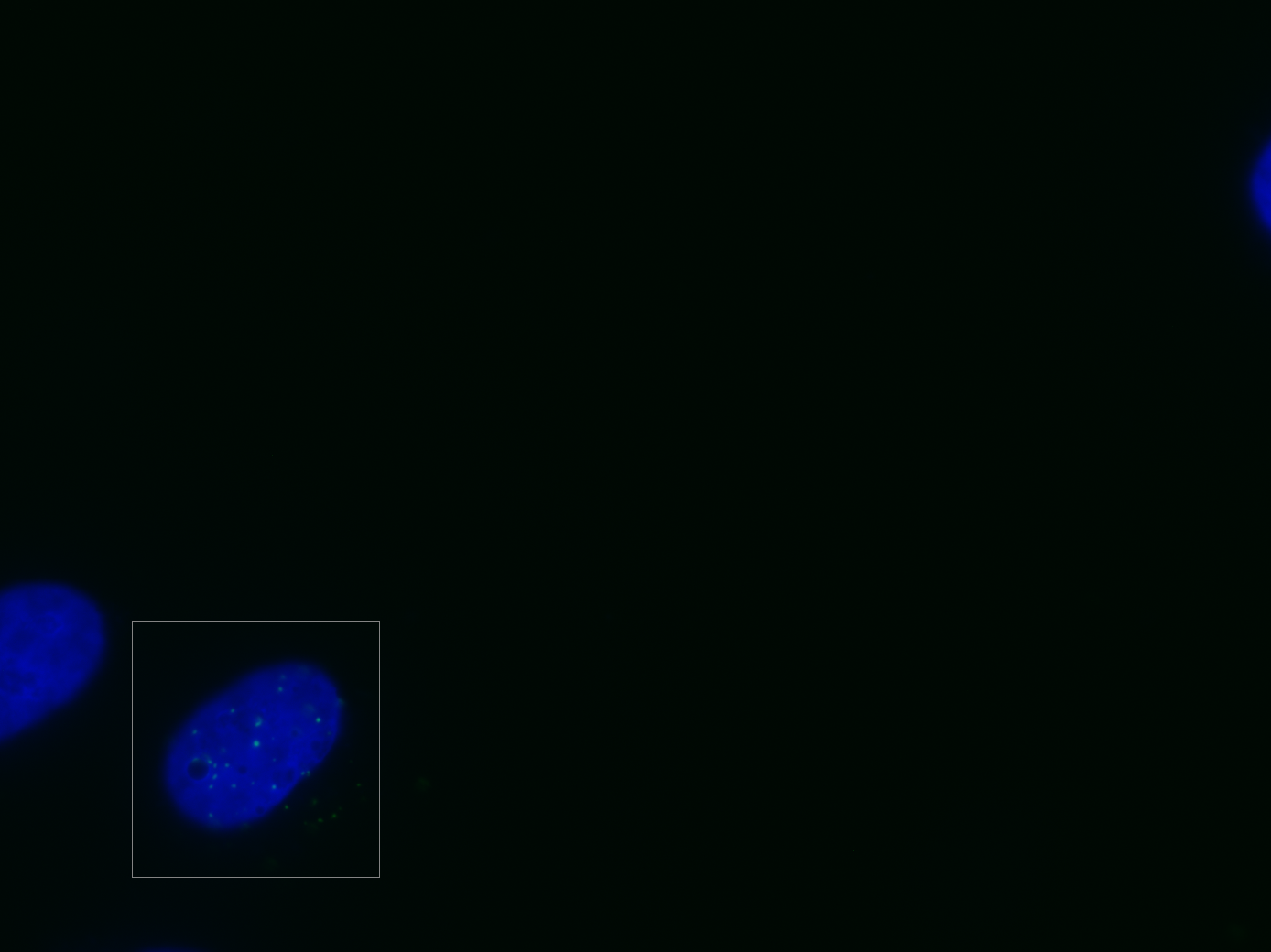

Supplement: Supplementary file 12 — Figure EV3 [file 44319_2025_497_MOESM12_ESM.zip › Figure EV 3A/shRAD51C IF Flag-RNF20 foci.tif]

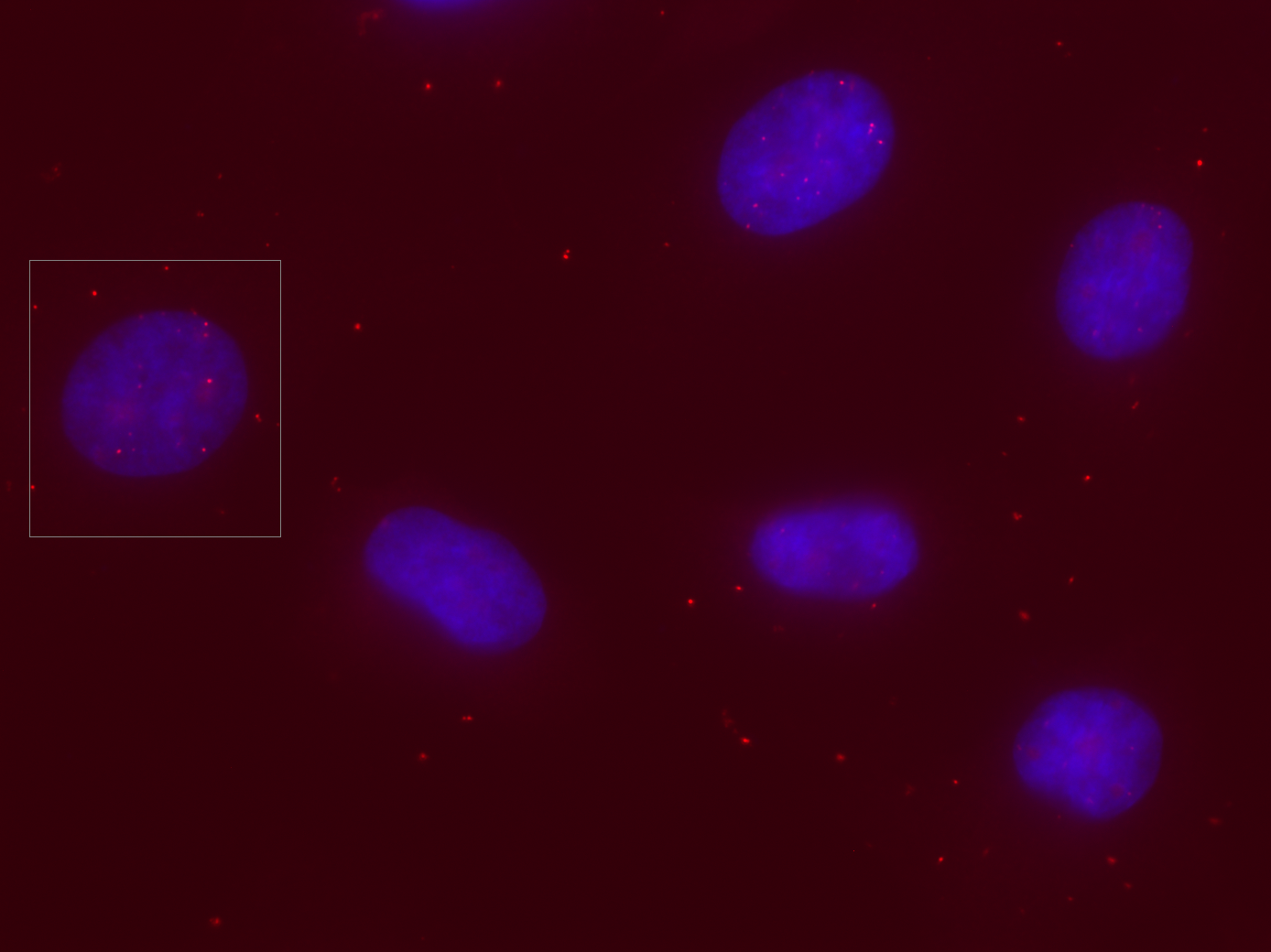

Supplement: Supplementary file 12 — Figure EV3 [file 44319_2025_497_MOESM12_ESM.zip › Figure EV 3C/shControl Progressing RAD51C SIRF.tif]

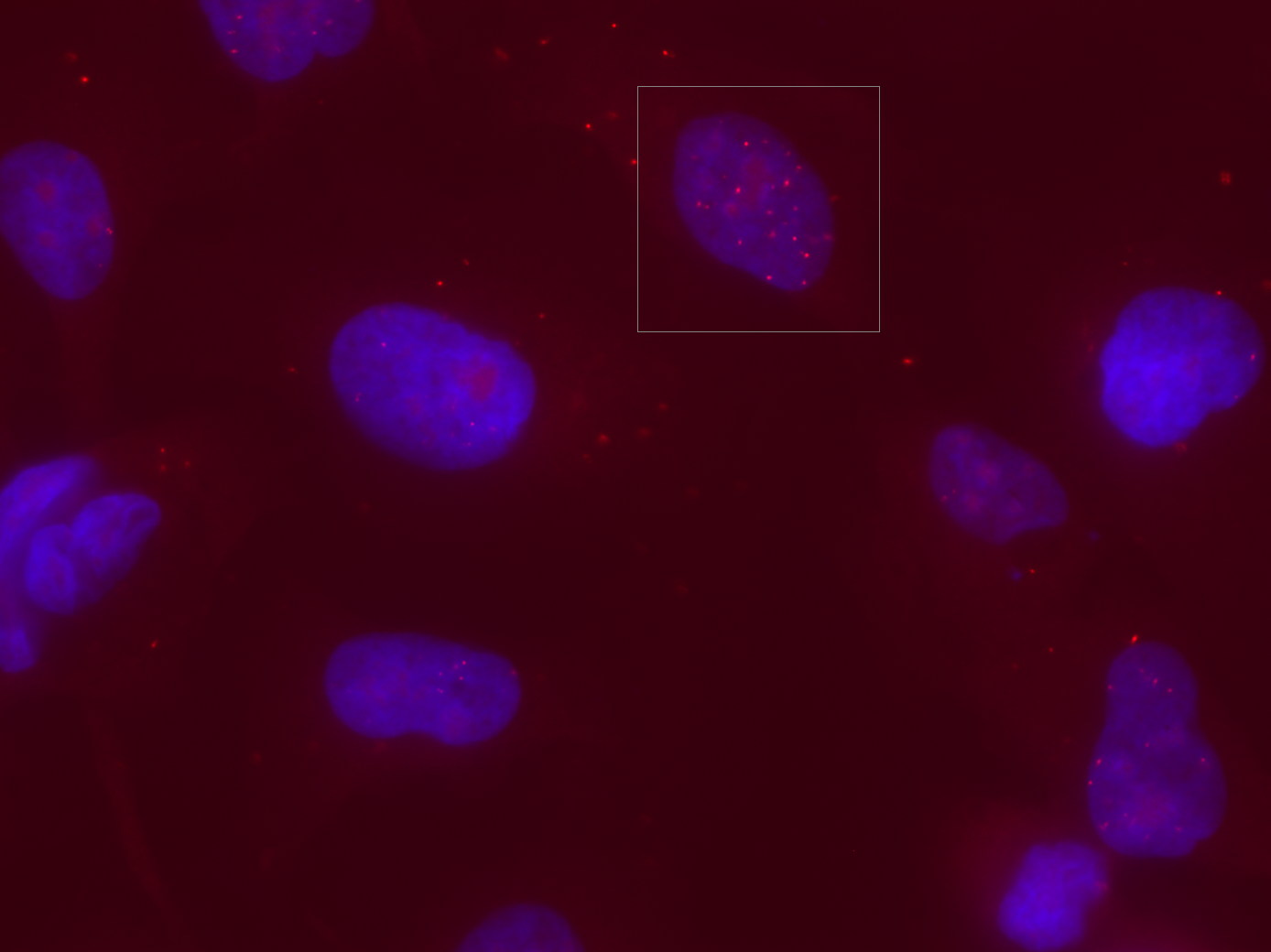

Supplement: Supplementary file 12 — Figure EV3 [file 44319_2025_497_MOESM12_ESM.zip › Figure EV 3C/shControl Stalled RAD51C SIRF.tif]

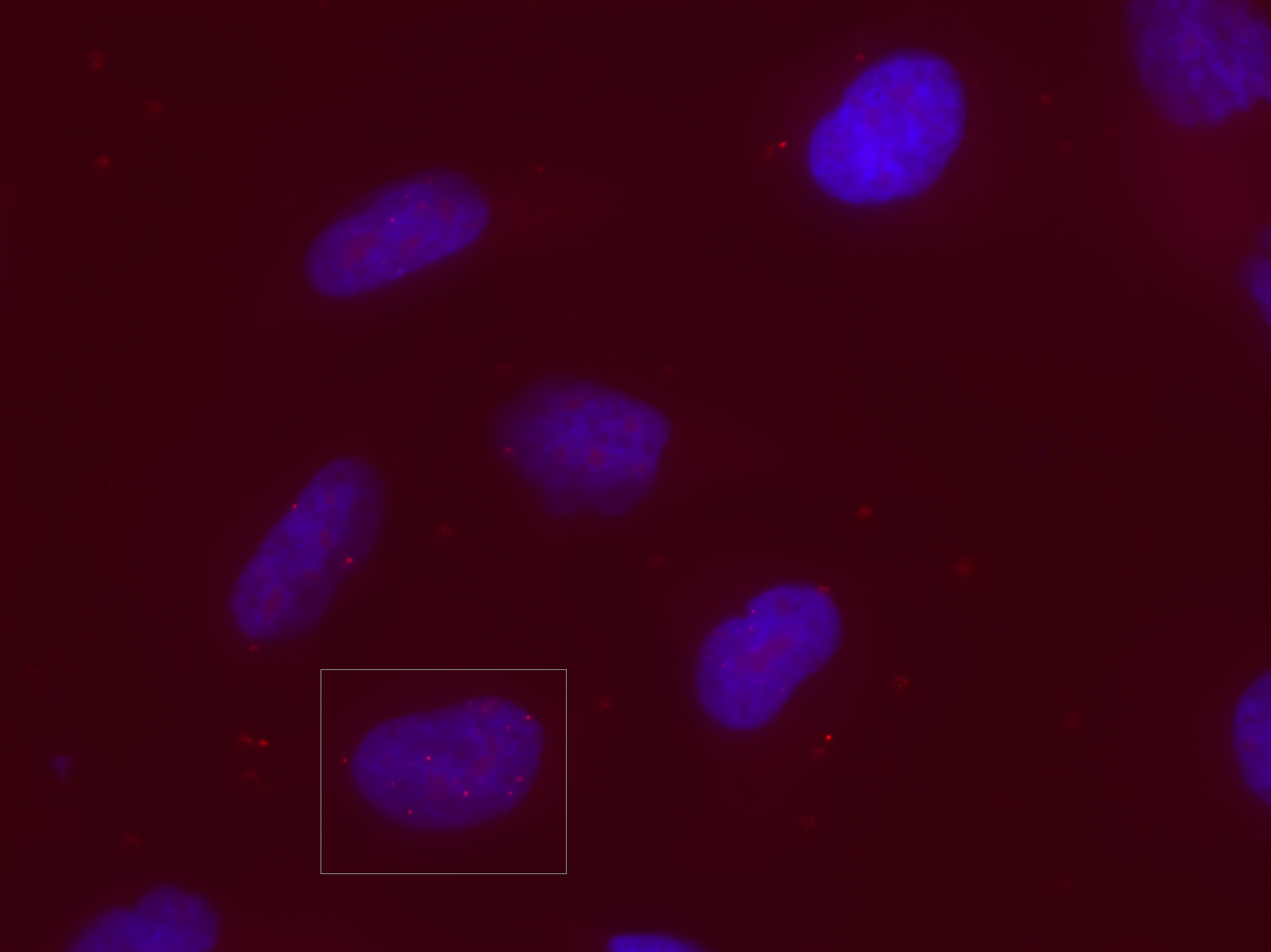

Supplement: Supplementary file 12 — Figure EV3 [file 44319_2025_497_MOESM12_ESM.zip › Figure EV 3C/shRNF20 Progressing RAD51C SIRF.tif]

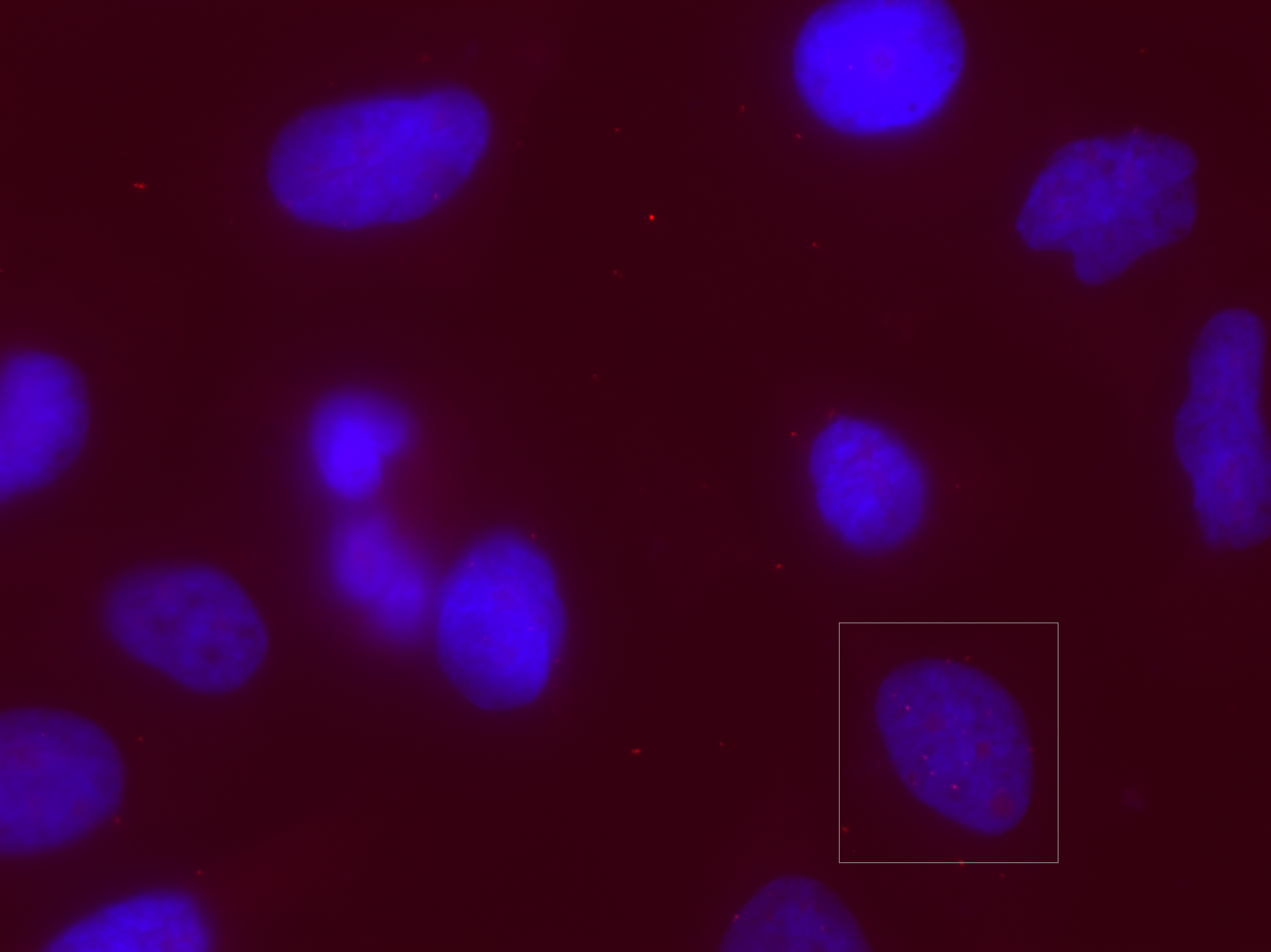

Supplement: Supplementary file 12 — Figure EV3 [file 44319_2025_497_MOESM12_ESM.zip › Figure EV 3C/shRNF20 Stalled RAD51C SIRF.tif]

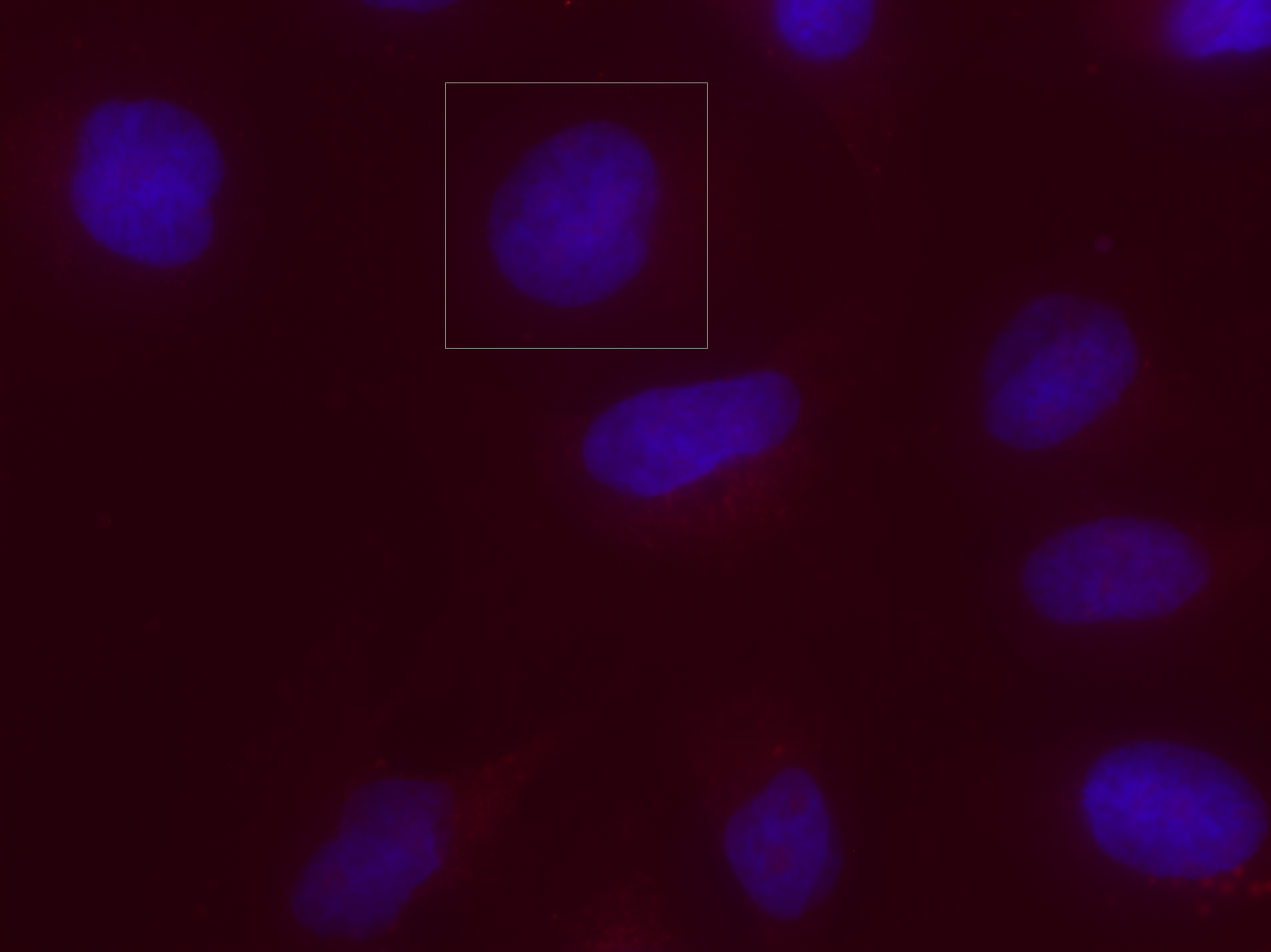

Supplement: Supplementary file 12 — Figure EV3 [file 44319_2025_497_MOESM12_ESM.zip › Figure EV 3D/shControl Progressing RAD51 SIRF Only EdU.tif]

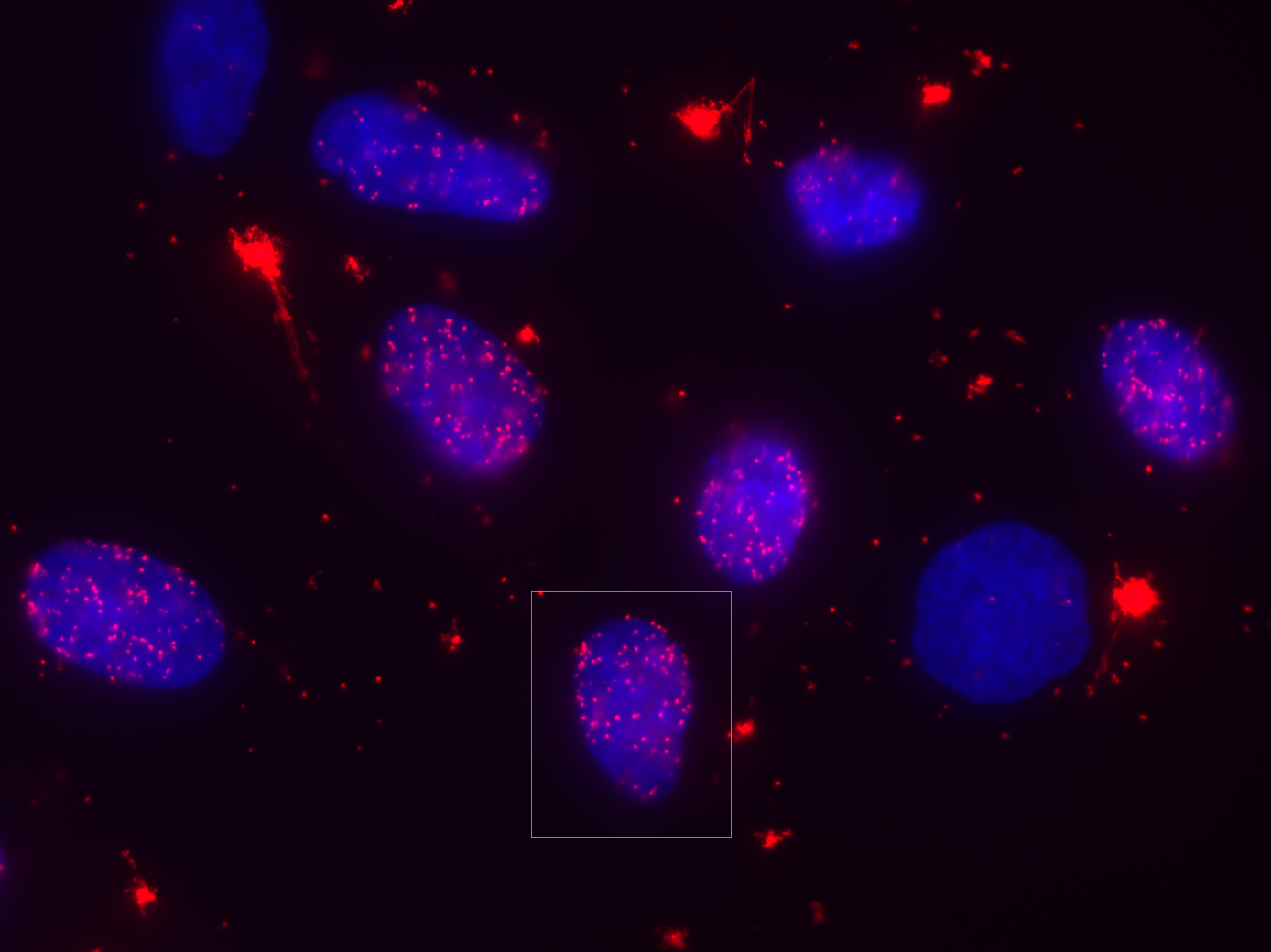

Supplement: Supplementary file 12 — Figure EV3 [file 44319_2025_497_MOESM12_ESM.zip › Figure EV 3D/shControl Progressing RAD51 SIRF.tif]

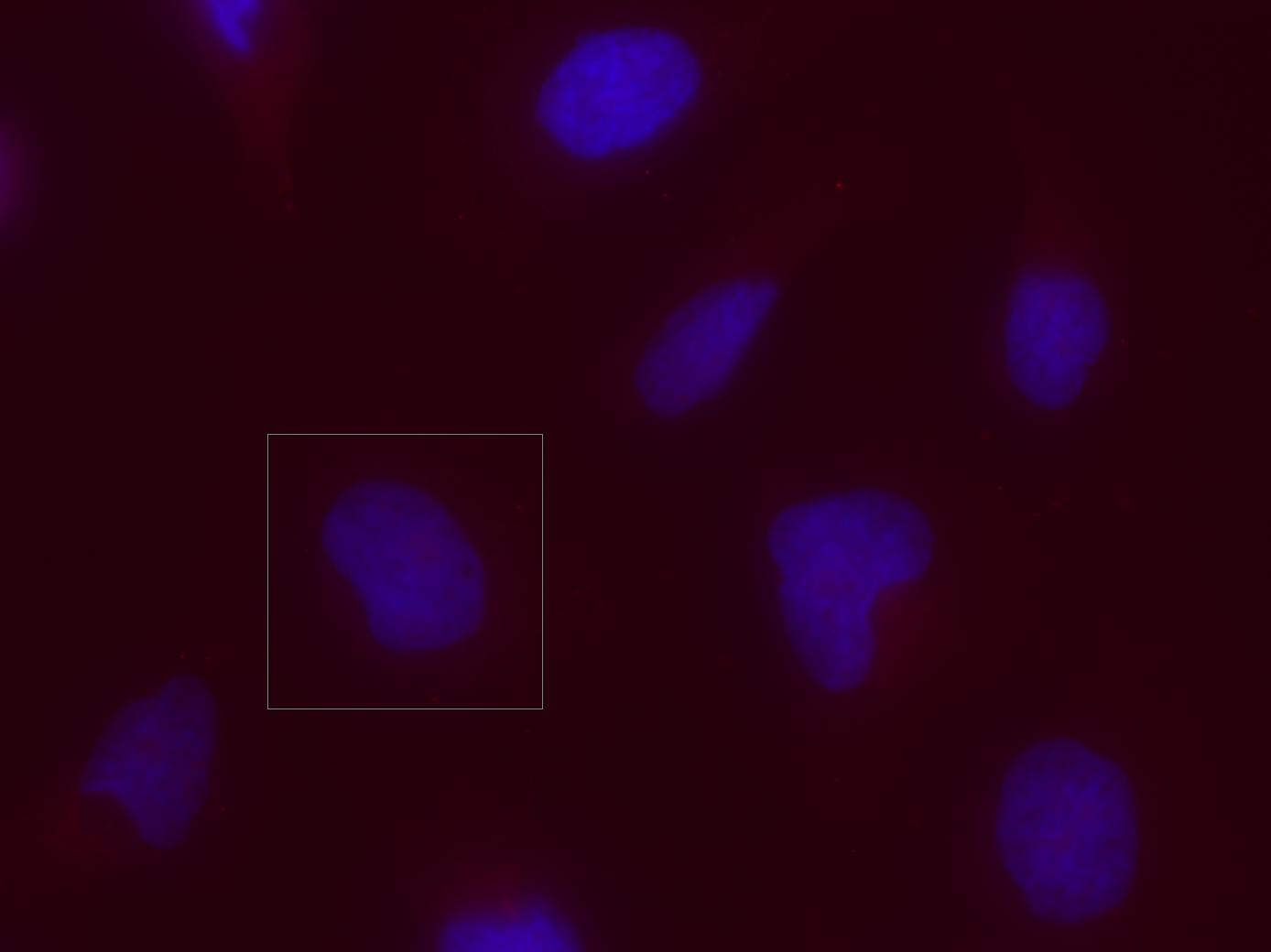

Supplement: Supplementary file 12 — Figure EV3 [file 44319_2025_497_MOESM12_ESM.zip › Figure EV 3D/shControl Stalled RAD51 SIRF Only EdU.tif]

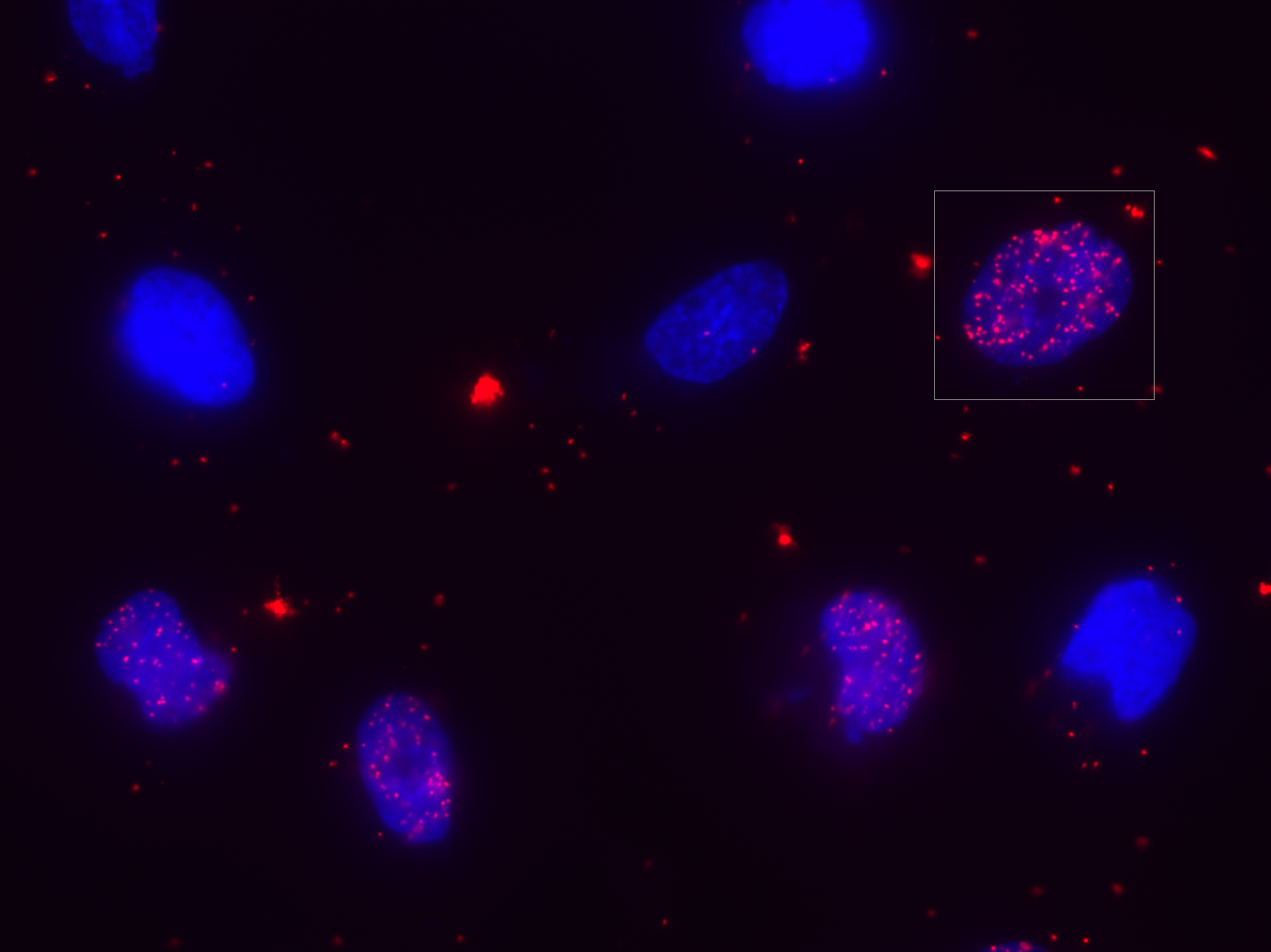

Supplement: Supplementary file 12 — Figure EV3 [file 44319_2025_497_MOESM12_ESM.zip › Figure EV 3D/shControl Stalled RAD51 SIRF.tif]

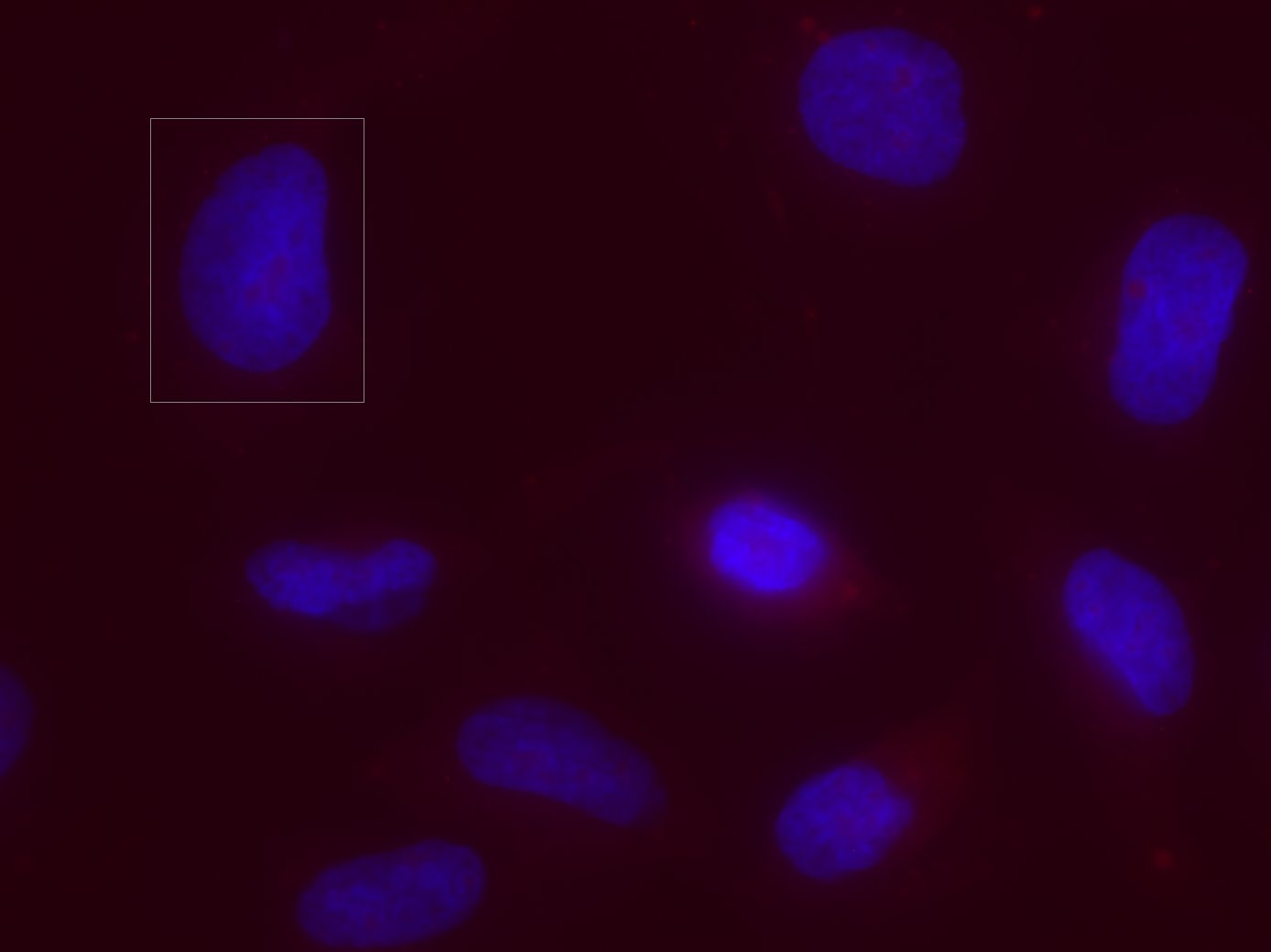

Supplement: Supplementary file 12 — Figure EV3 [file 44319_2025_497_MOESM12_ESM.zip › Figure EV 3D/shRNF20 Progressing RAD51 SIRF Only EdU.tif]

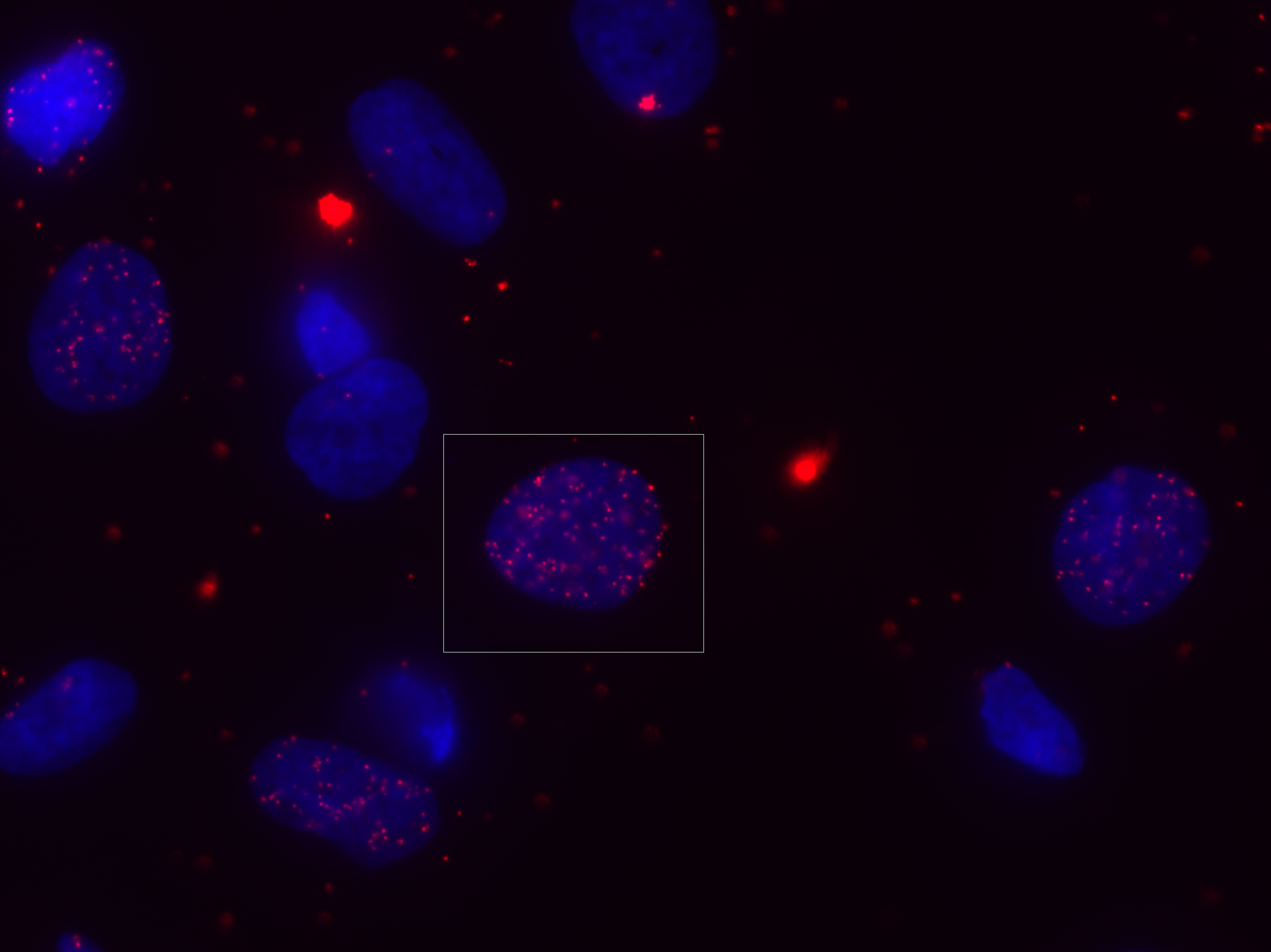

Supplement: Supplementary file 12 — Figure EV3 [file 44319_2025_497_MOESM12_ESM.zip › Figure EV 3D/shRNF20 Progressing RAD51 SIRF.tif]

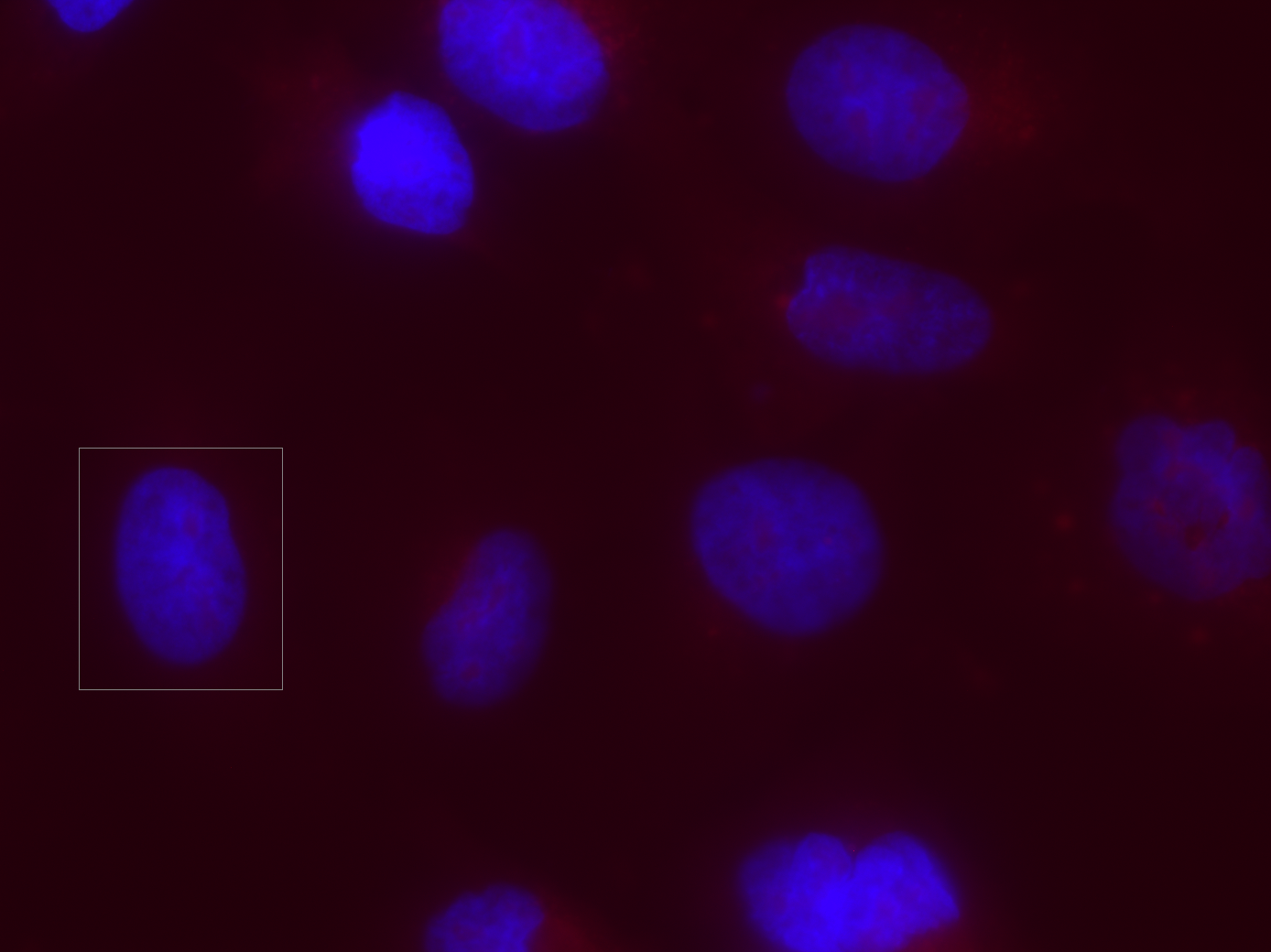

Supplement: Supplementary file 12 — Figure EV3 [file 44319_2025_497_MOESM12_ESM.zip › Figure EV 3D/shRNF20 Stalled RAD51 SIRF Only EdU.tif]

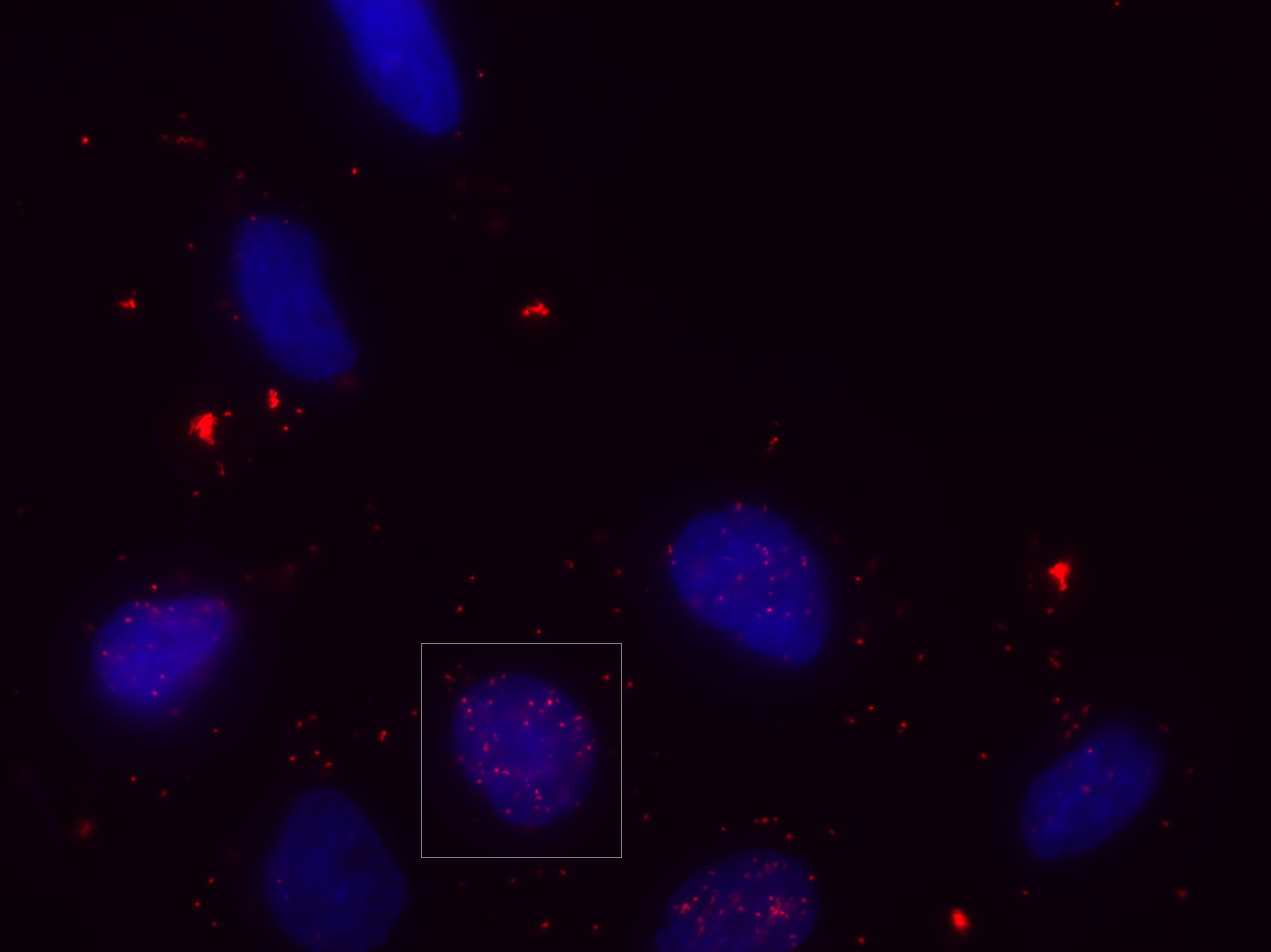

Supplement: Supplementary file 12 — Figure EV3 [file 44319_2025_497_MOESM12_ESM.zip › Figure EV 3D/shRNF20 Stalled RAD51 SIRF.tif]

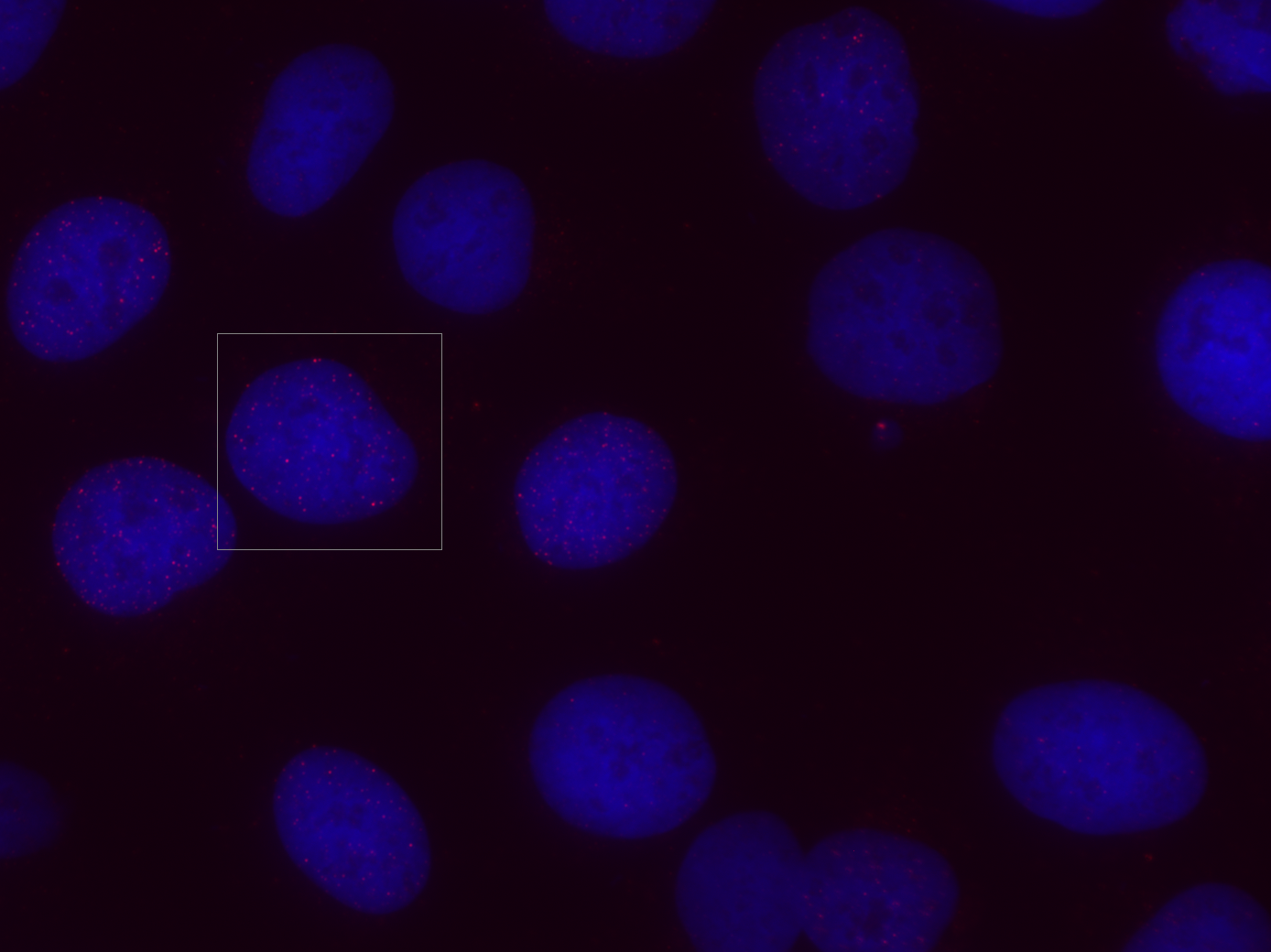

Supplement: Supplementary file 12 — Figure EV3 [file 44319_2025_497_MOESM12_ESM.zip › Figure EV 3E/shControl HU IF RAD51C foci.tif]

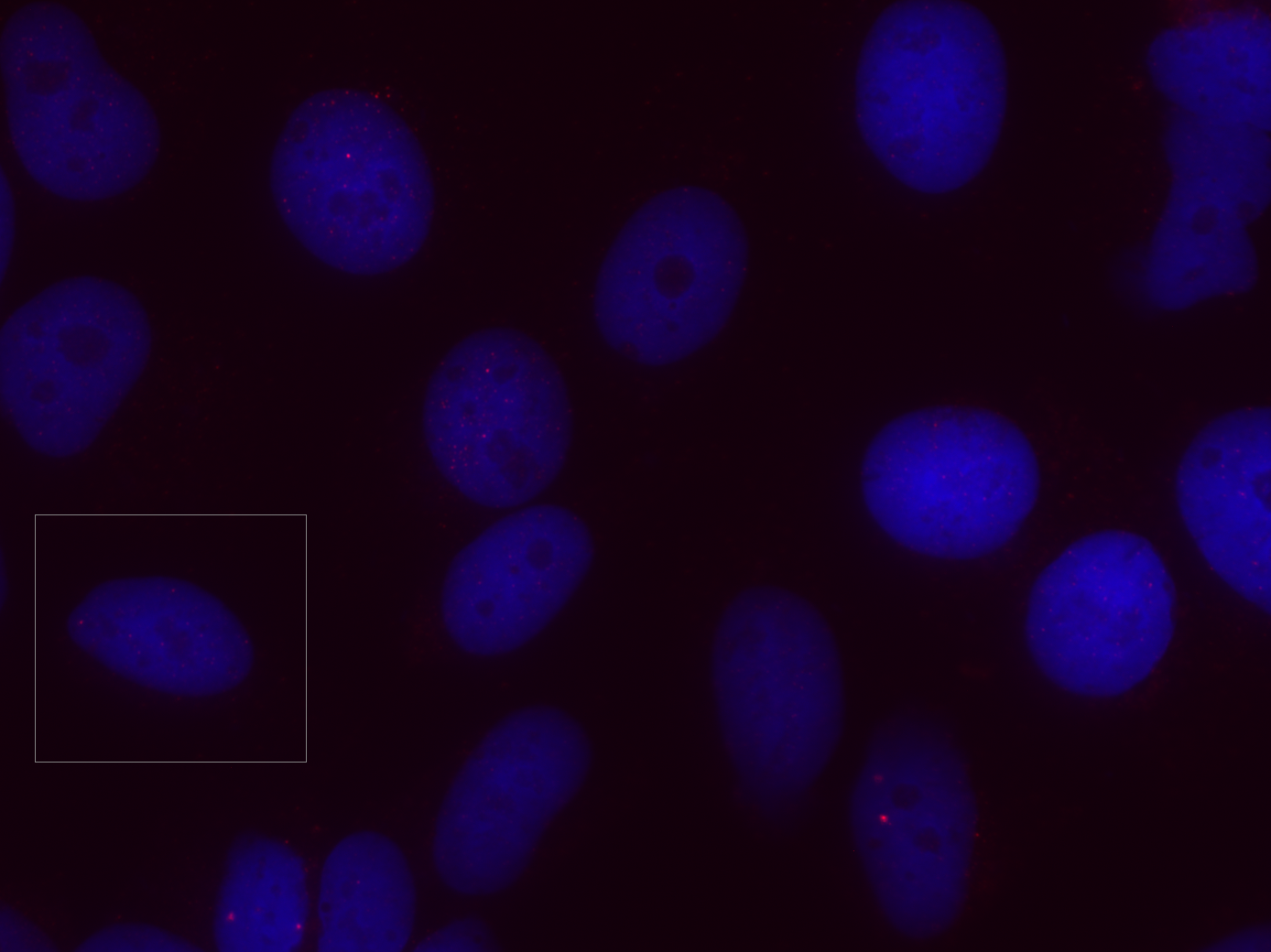

Supplement: Supplementary file 12 — Figure EV3 [file 44319_2025_497_MOESM12_ESM.zip › Figure EV 3E/shRNF20 HU IF RAD51C foci.tif]

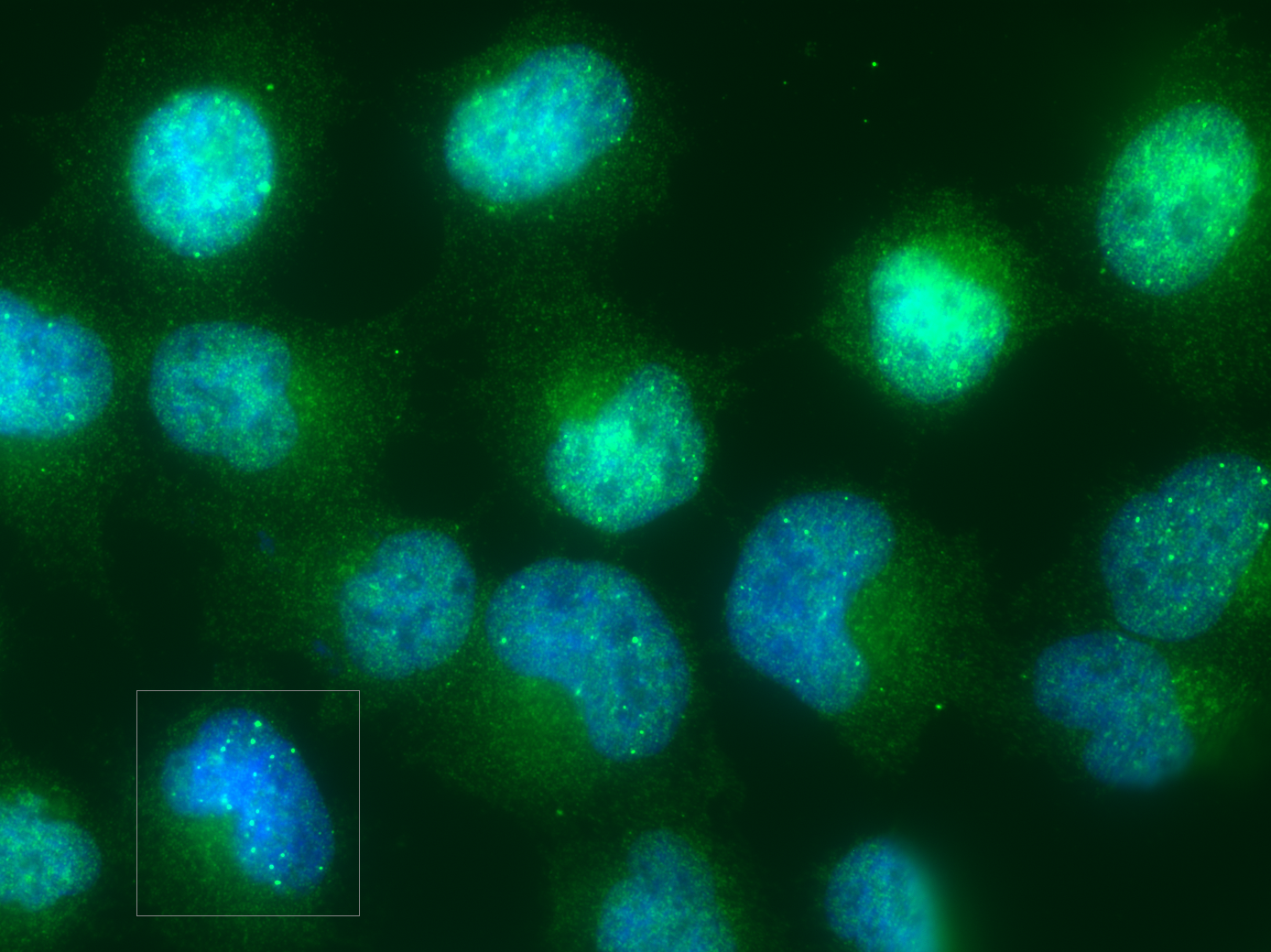

Supplement: Supplementary file 12 — Figure EV3 [file 44319_2025_497_MOESM12_ESM.zip › Figure EV 3G/shControl HU IF RAD51 foci.tif]

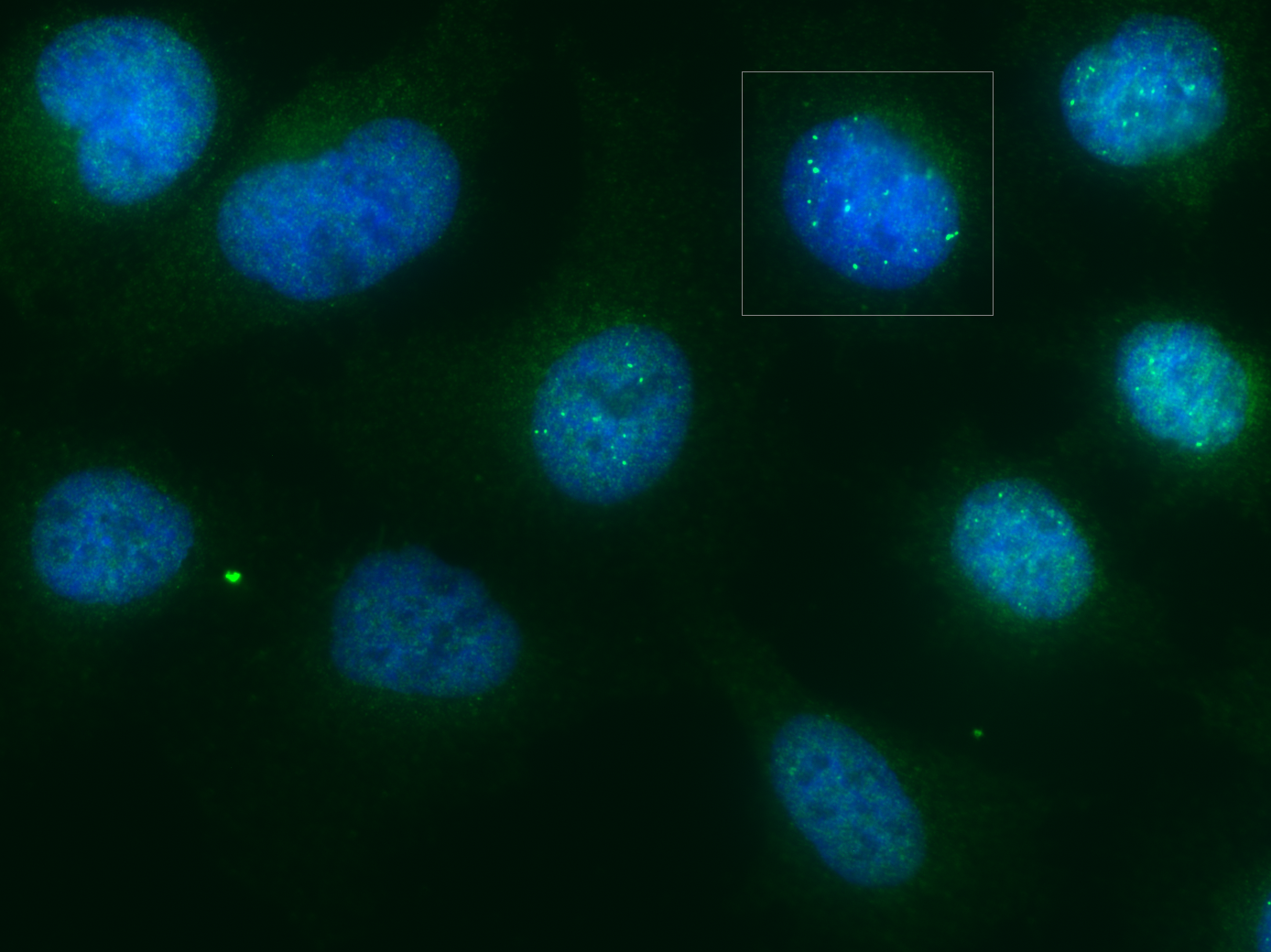

Supplement: Supplementary file 12 — Figure EV3 [file 44319_2025_497_MOESM12_ESM.zip › Figure EV 3G/shRNF20 HU IF RAD51 foci.tif]

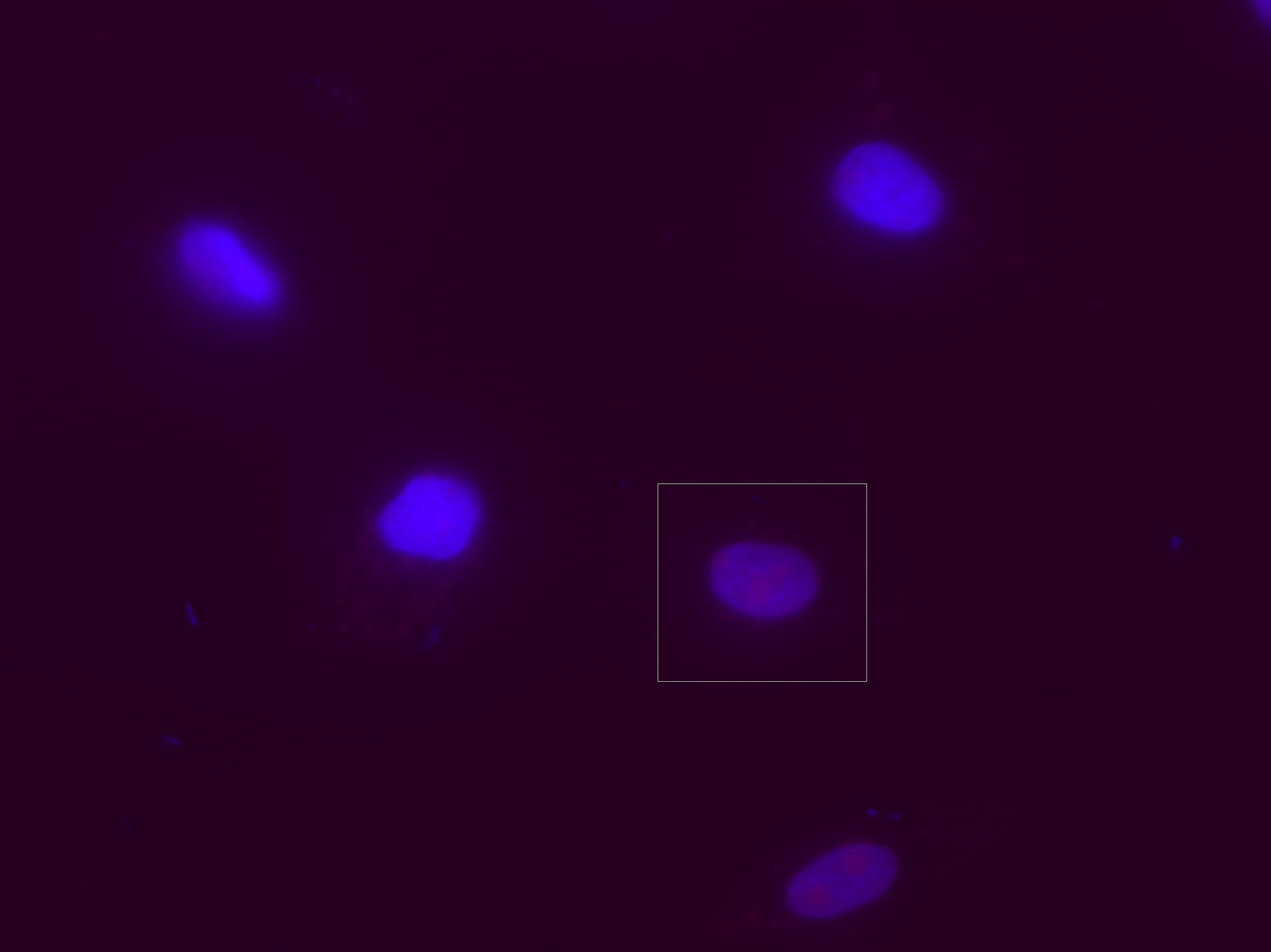

Supplement: Supplementary file 12 — Figure EV3 [file 44319_2025_497_MOESM12_ESM.zip › Figure EV 3I/Only EdU BRCA2 SIRF.tif]

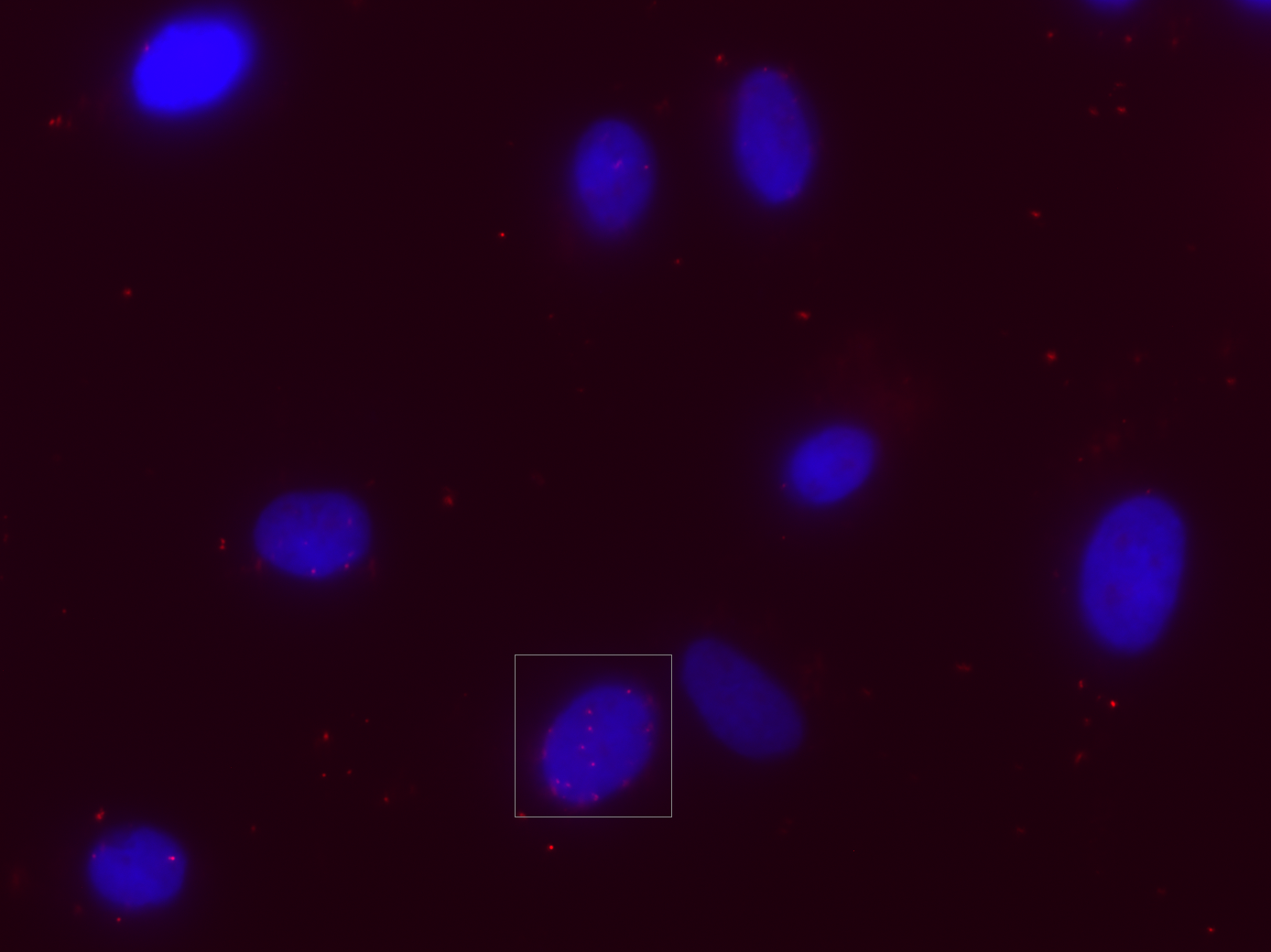

Supplement: Supplementary file 12 — Figure EV3 [file 44319_2025_497_MOESM12_ESM.zip › Figure EV 3I/shControl BRCA2 SIRF.tif]

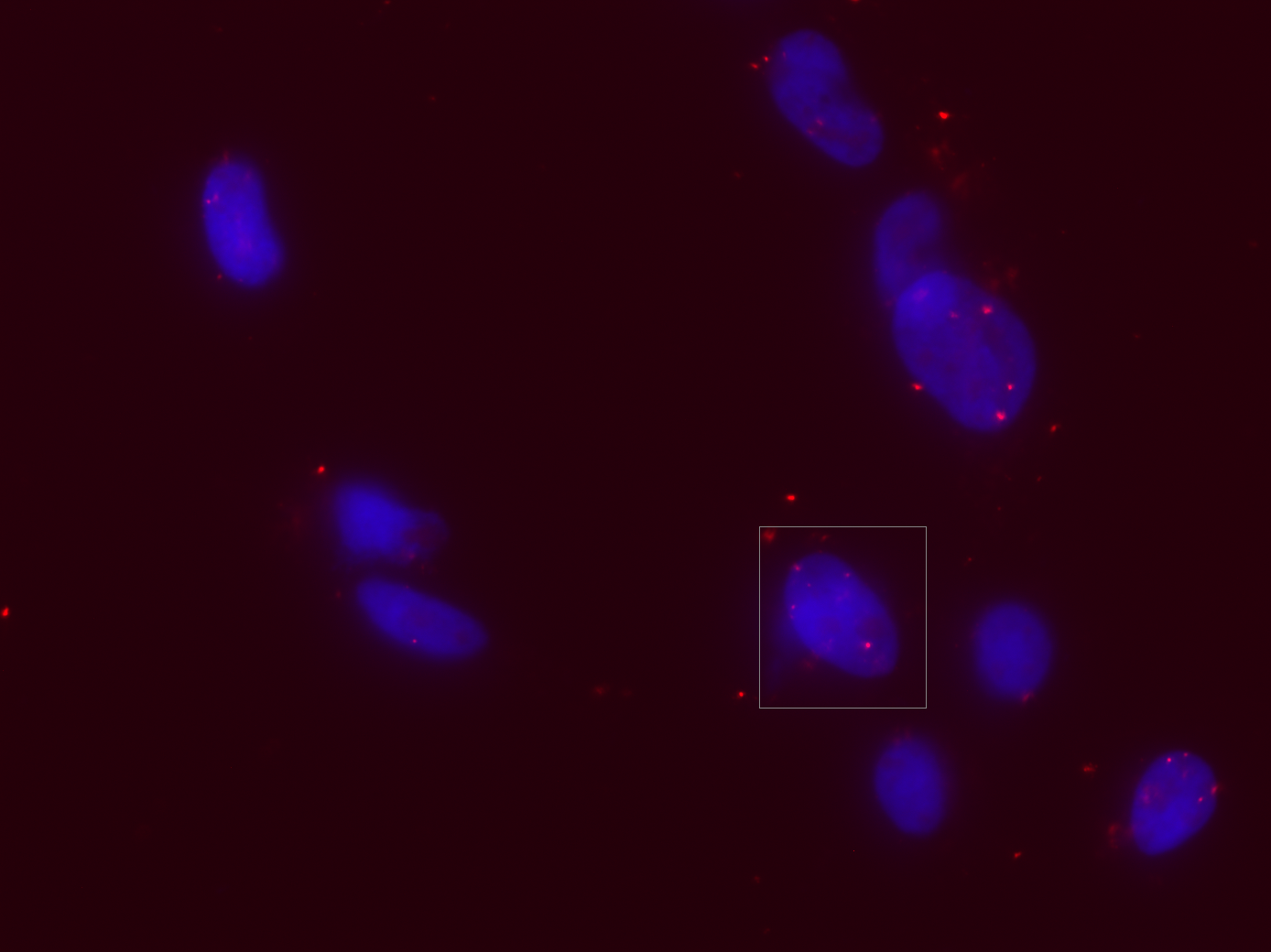

Supplement: Supplementary file 12 — Figure EV3 [file 44319_2025_497_MOESM12_ESM.zip › Figure EV 3I/shRNF20 BRCA2 SIRF.tif]

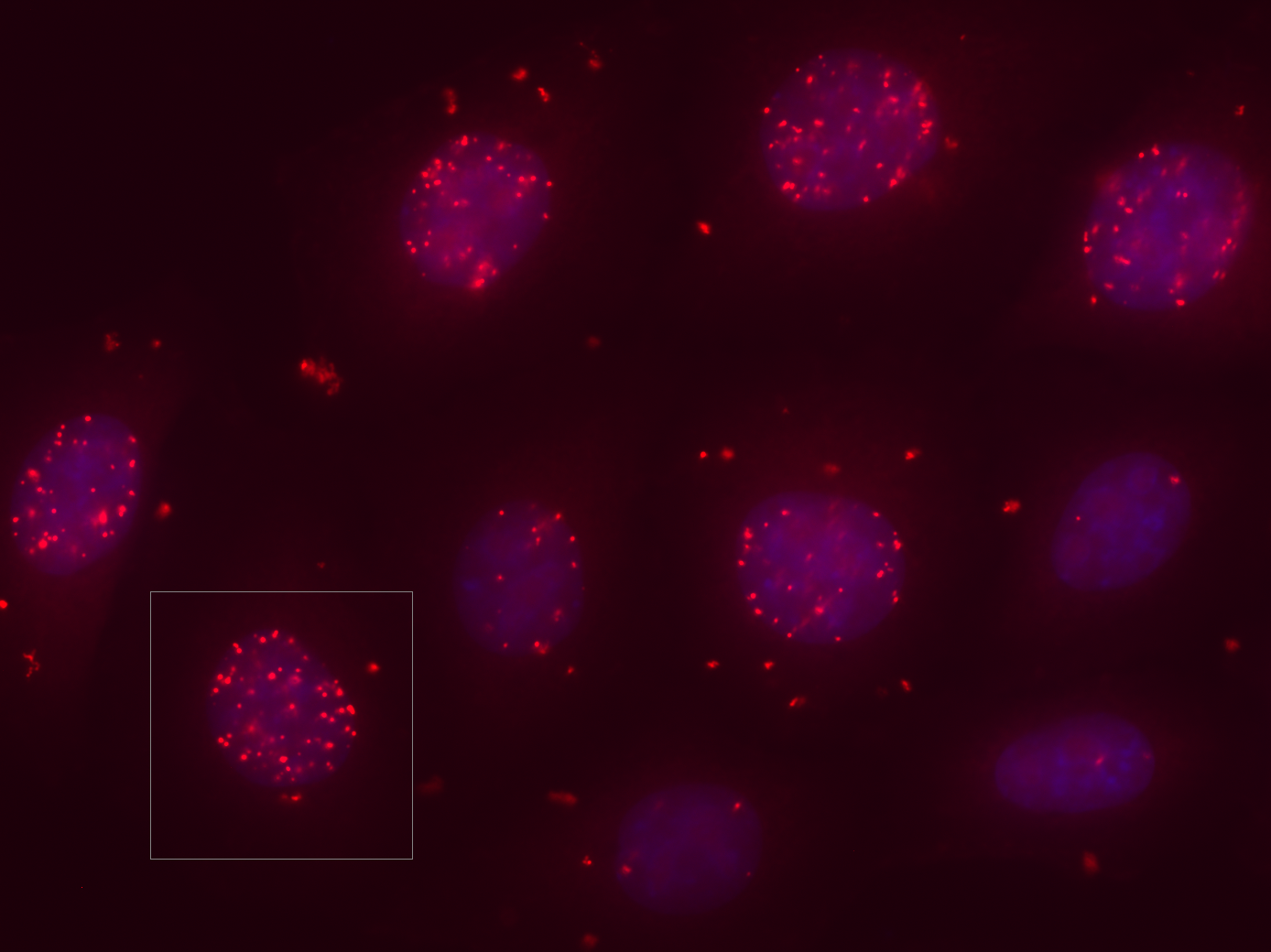

Supplement: Supplementary file 13 — Figure EV4 [file 44319_2025_497_MOESM13_ESM.zip › Figure EV 4A/shControl H2B K120ub SIRF.tif]

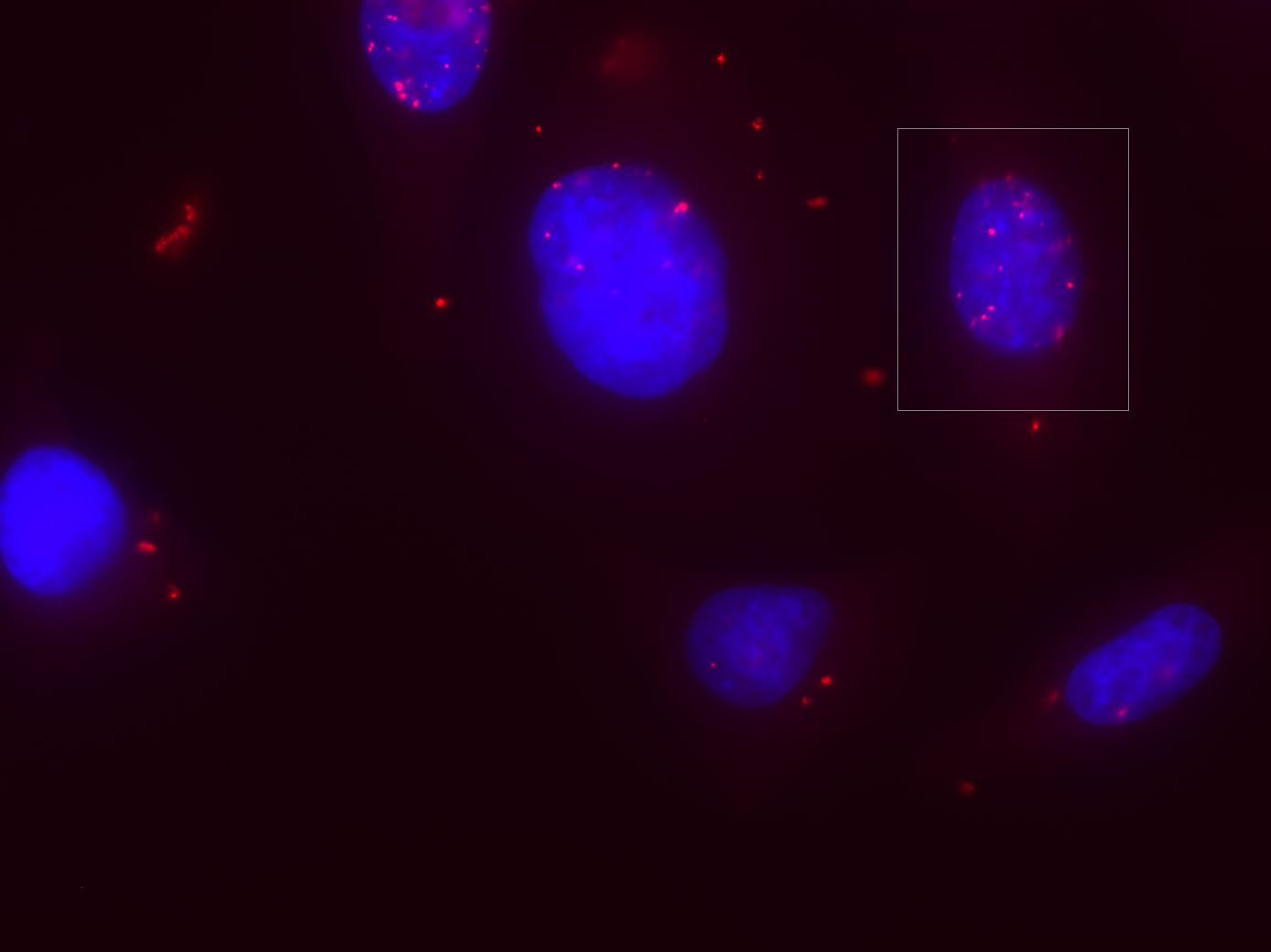

Supplement: Supplementary file 13 — Figure EV4 [file 44319_2025_497_MOESM13_ESM.zip › Figure EV 4A/shRNF20+C922S H2B K120ub SIRF.tif]

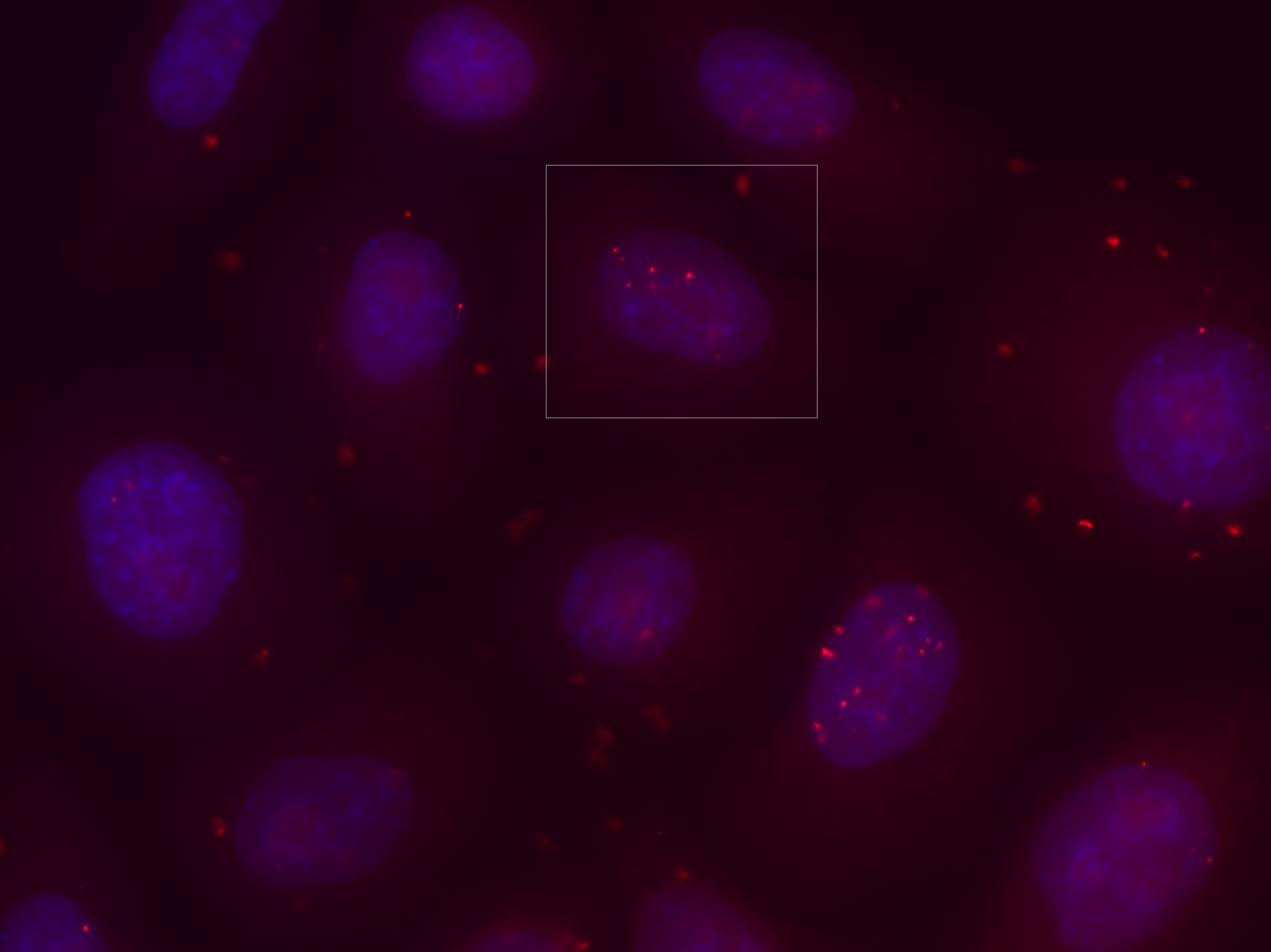

Supplement: Supplementary file 13 — Figure EV4 [file 44319_2025_497_MOESM13_ESM.zip › Figure EV 4A/shRNF20+C960A H2B K120ub SIRF.tif]

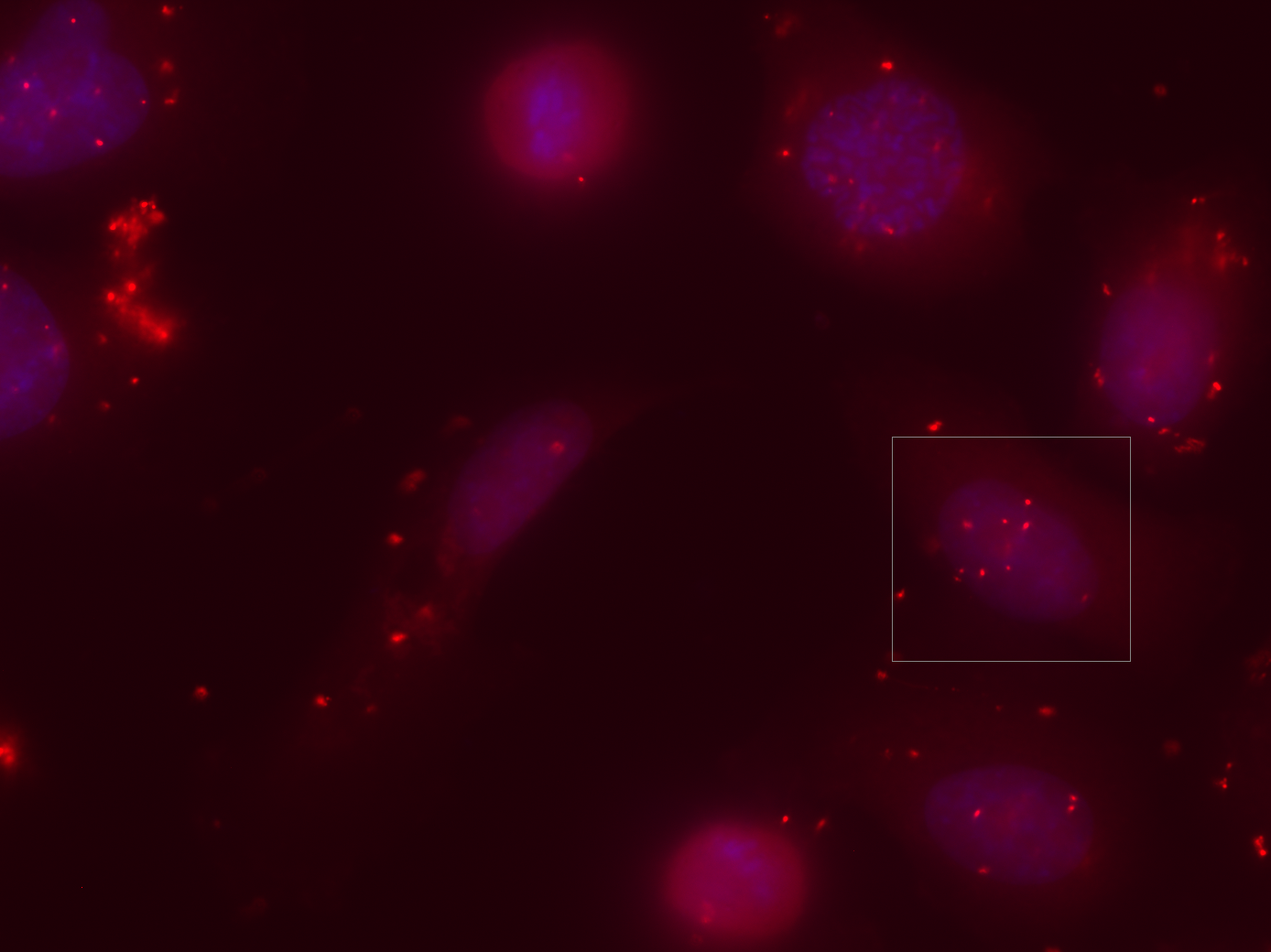

Supplement: Supplementary file 13 — Figure EV4 [file 44319_2025_497_MOESM13_ESM.zip › Figure EV 4A/shRNF20+EV H2B K120ub SIRF.tif]

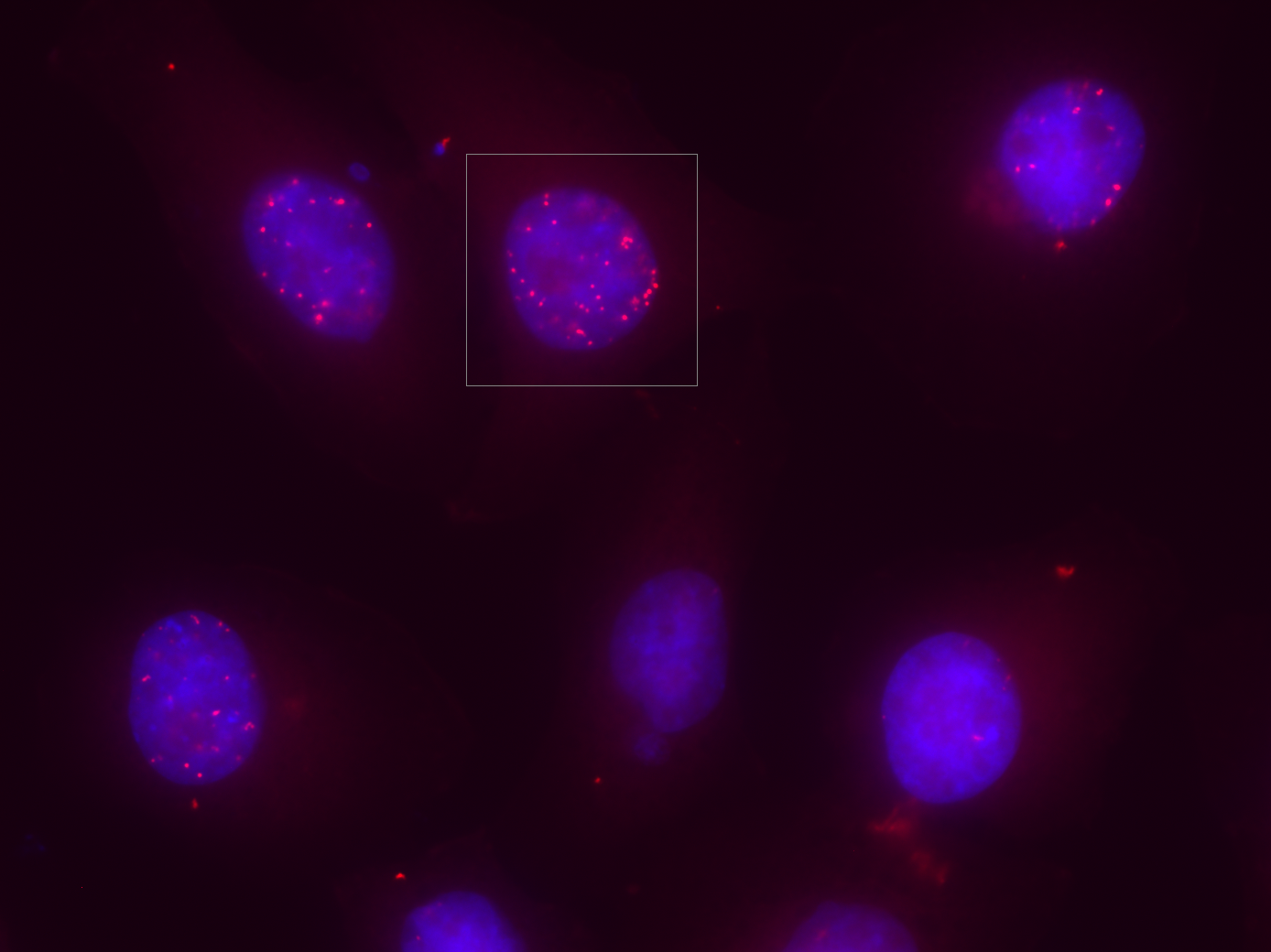

Supplement: Supplementary file 13 — Figure EV4 [file 44319_2025_497_MOESM13_ESM.zip › Figure EV 4A/shRNF20+WT H2B K120ub SIRF.tif]

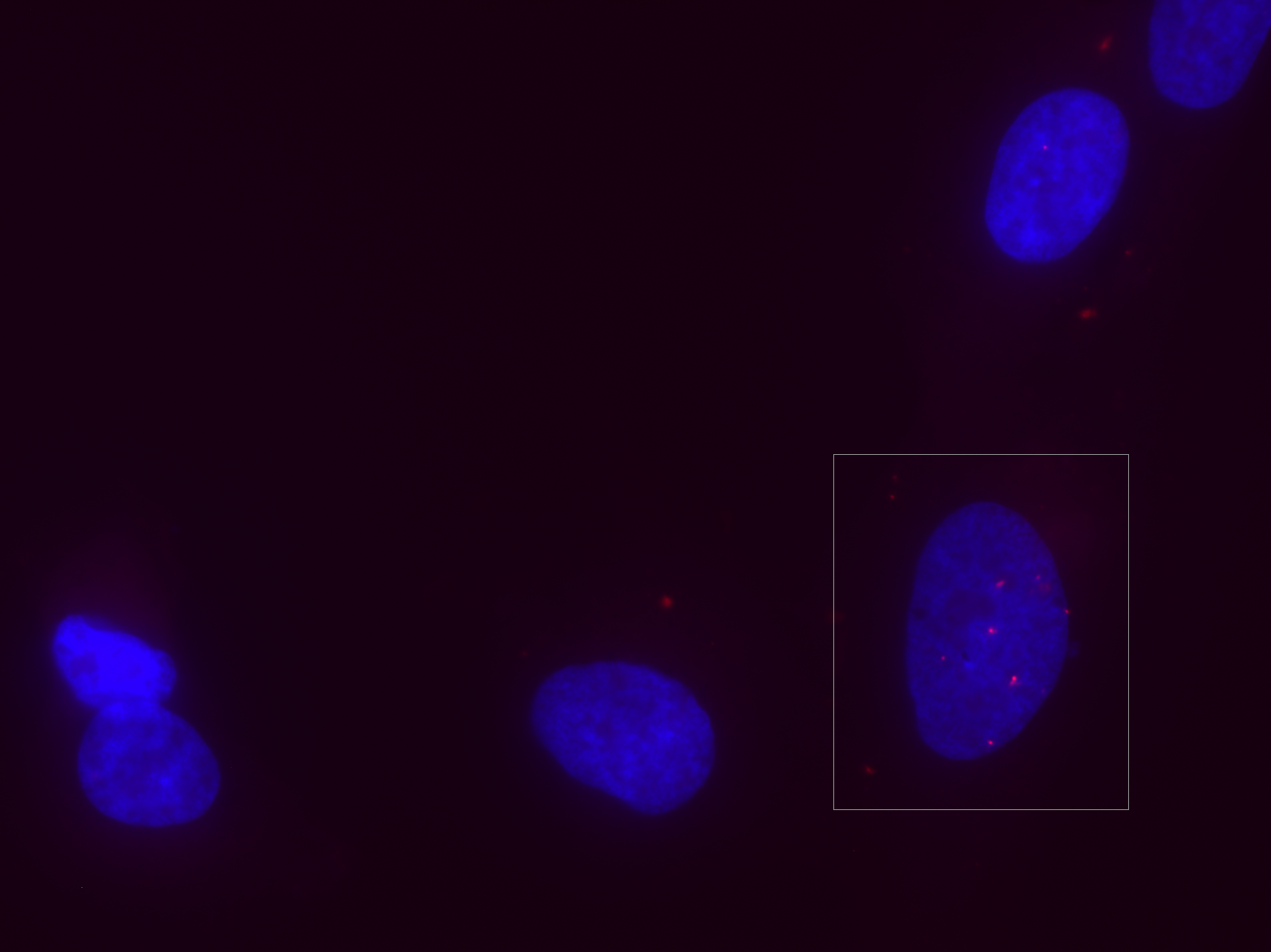

Supplement: Supplementary file 13 — Figure EV4 [file 44319_2025_497_MOESM13_ESM.zip › Figure EV 4C/C922S Flag SIRF.tif]

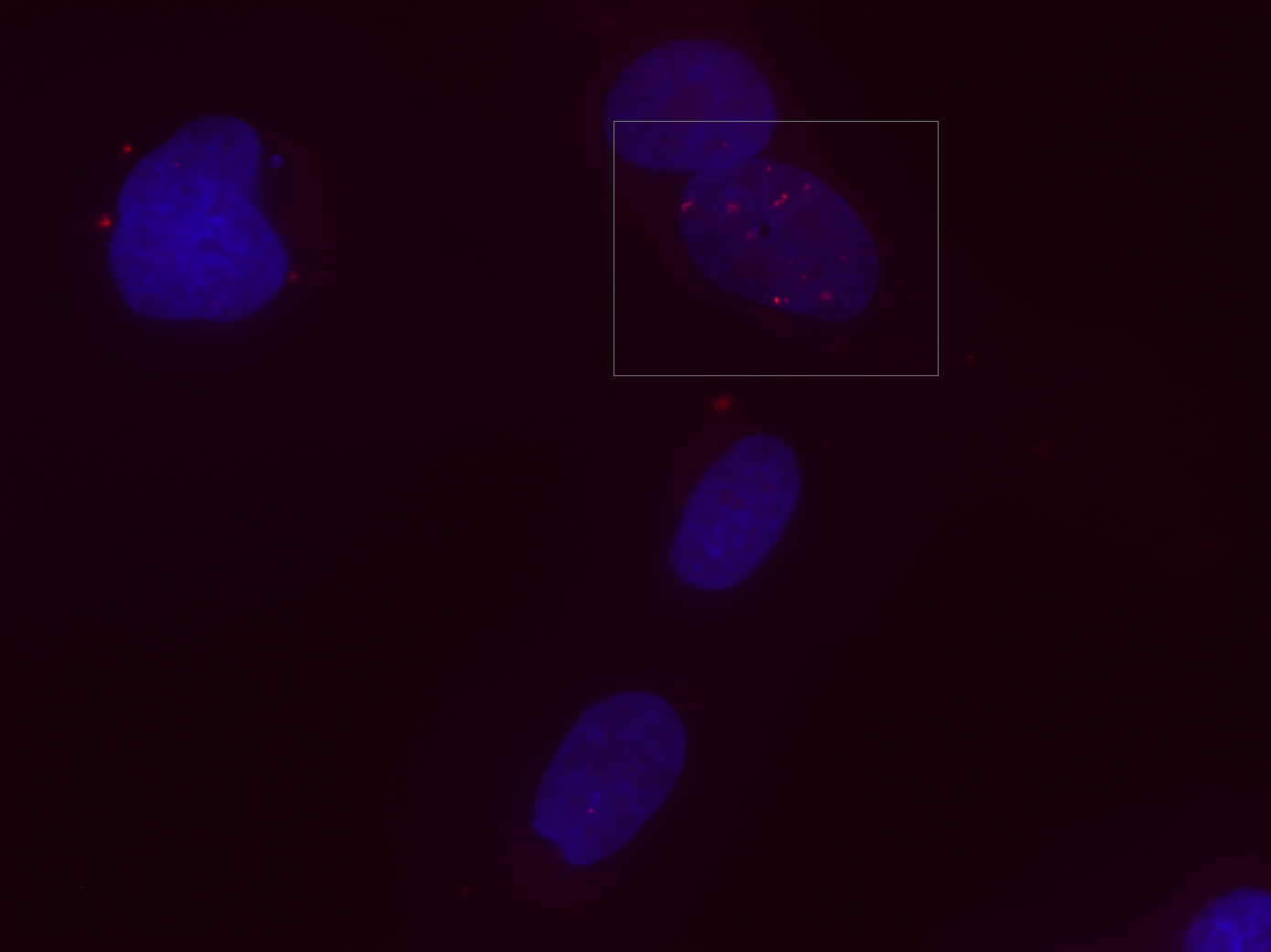

Supplement: Supplementary file 13 — Figure EV4 [file 44319_2025_497_MOESM13_ESM.zip › Figure EV 4C/C960A Flag SIRF.tif]

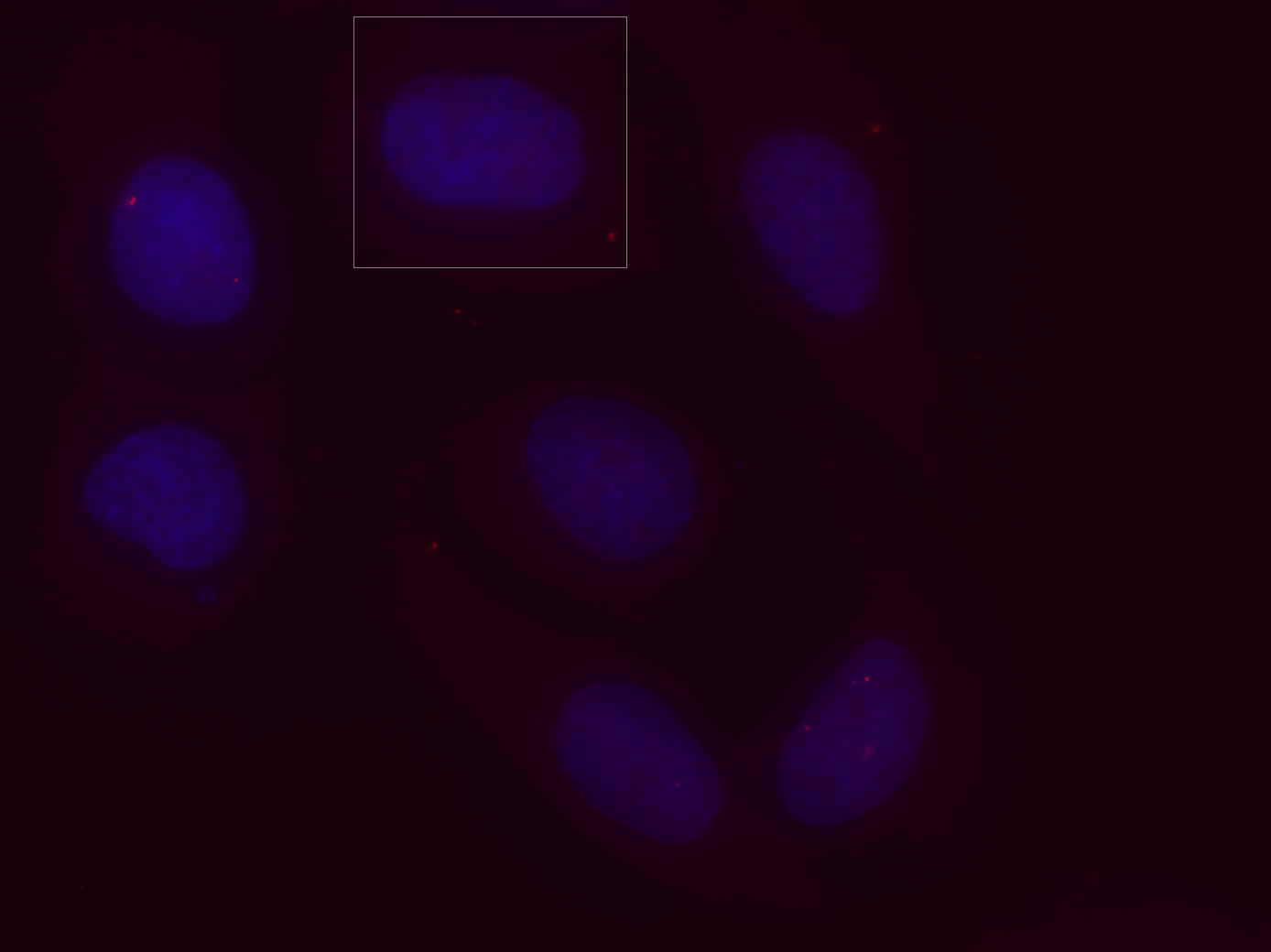

Supplement: Supplementary file 13 — Figure EV4 [file 44319_2025_497_MOESM13_ESM.zip › Figure EV 4C/EV Flag SIRF.tif]

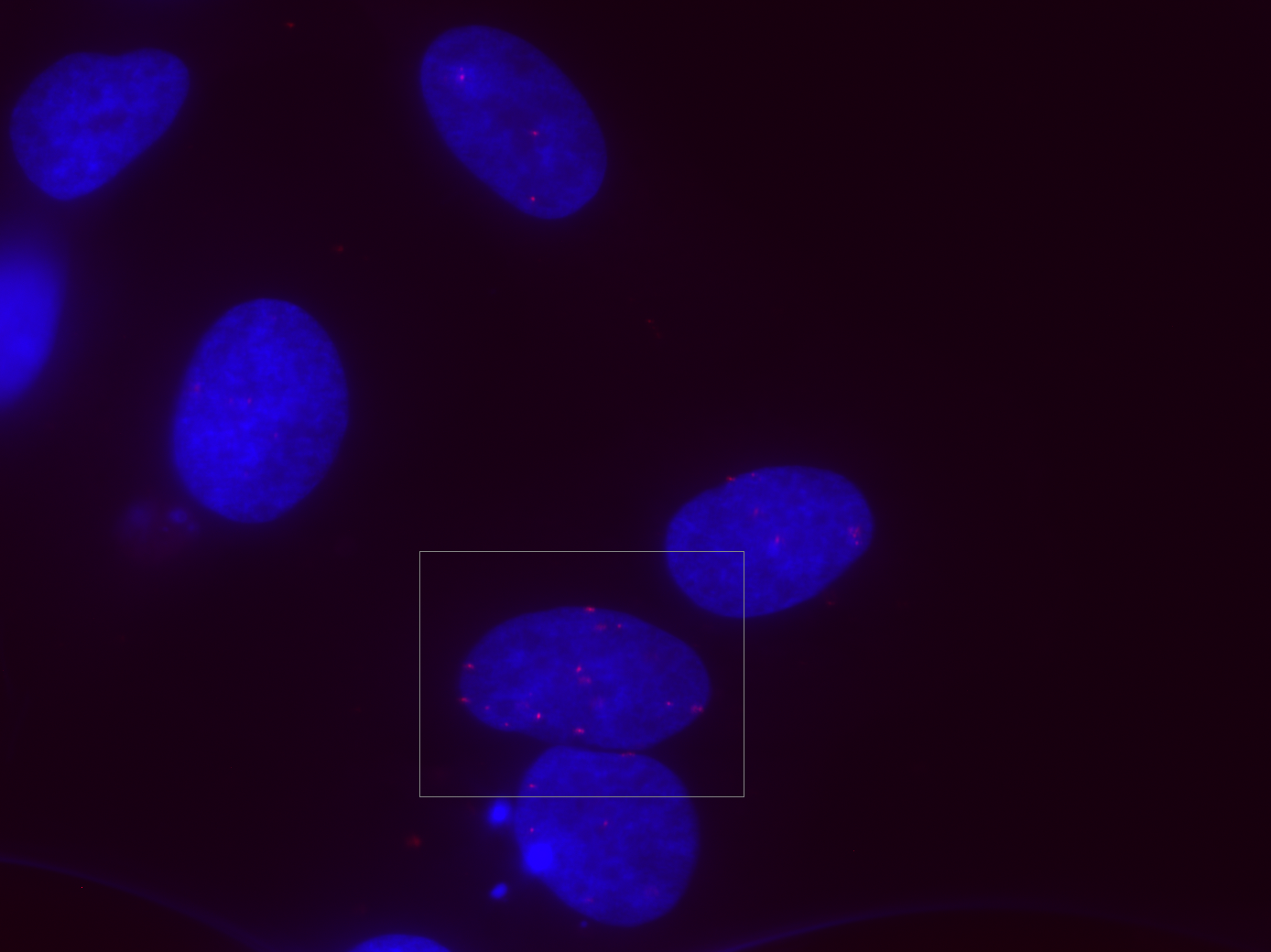

Supplement: Supplementary file 13 — Figure EV4 [file 44319_2025_497_MOESM13_ESM.zip › Figure EV 4C/WT Flag SIRF.tif]

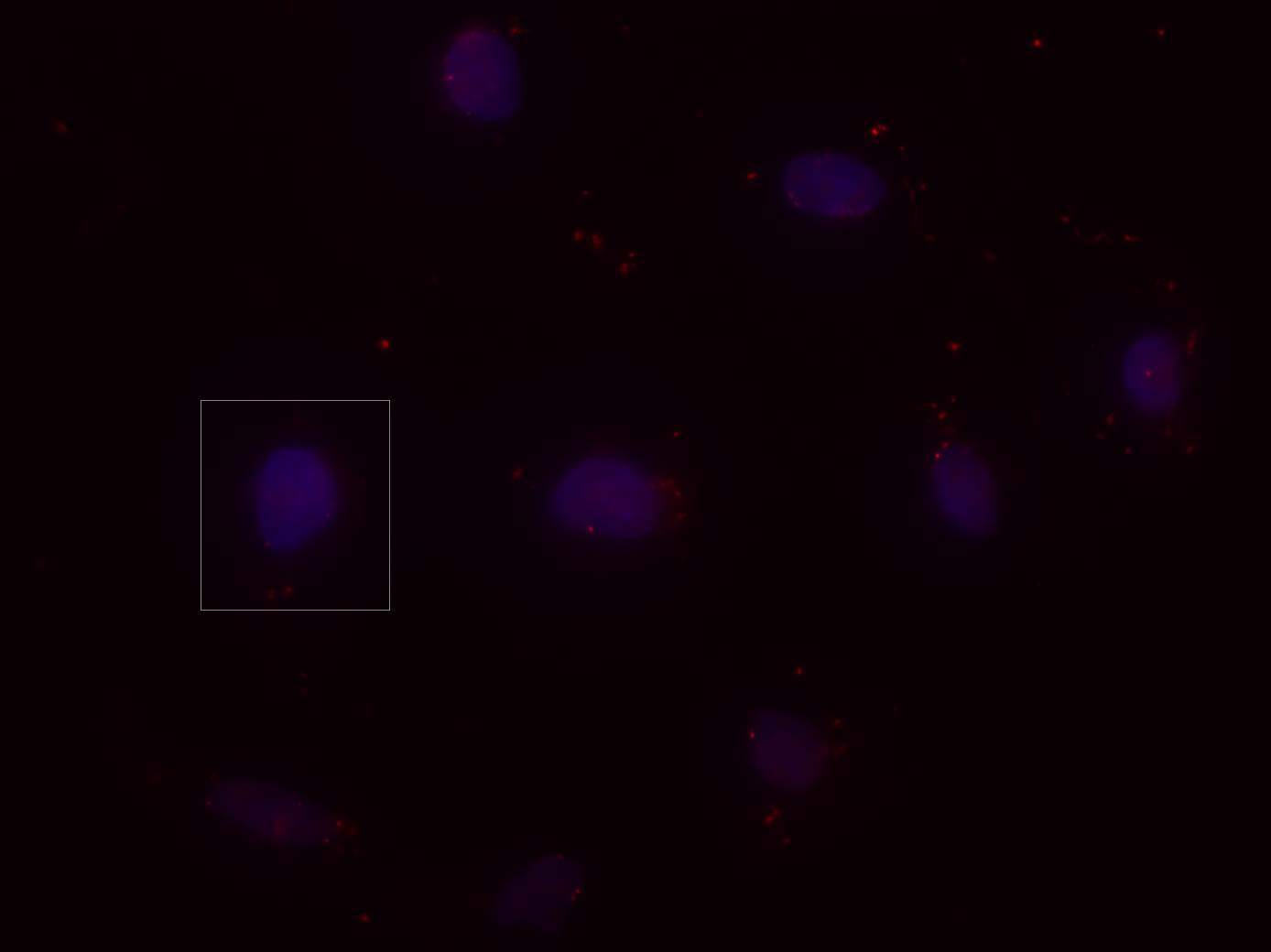

Supplement: Supplementary file 13 — Figure EV4 [file 44319_2025_497_MOESM13_ESM.zip › Figure EV 4E/Only EdU HU H3K4me3 SIRF.tif]

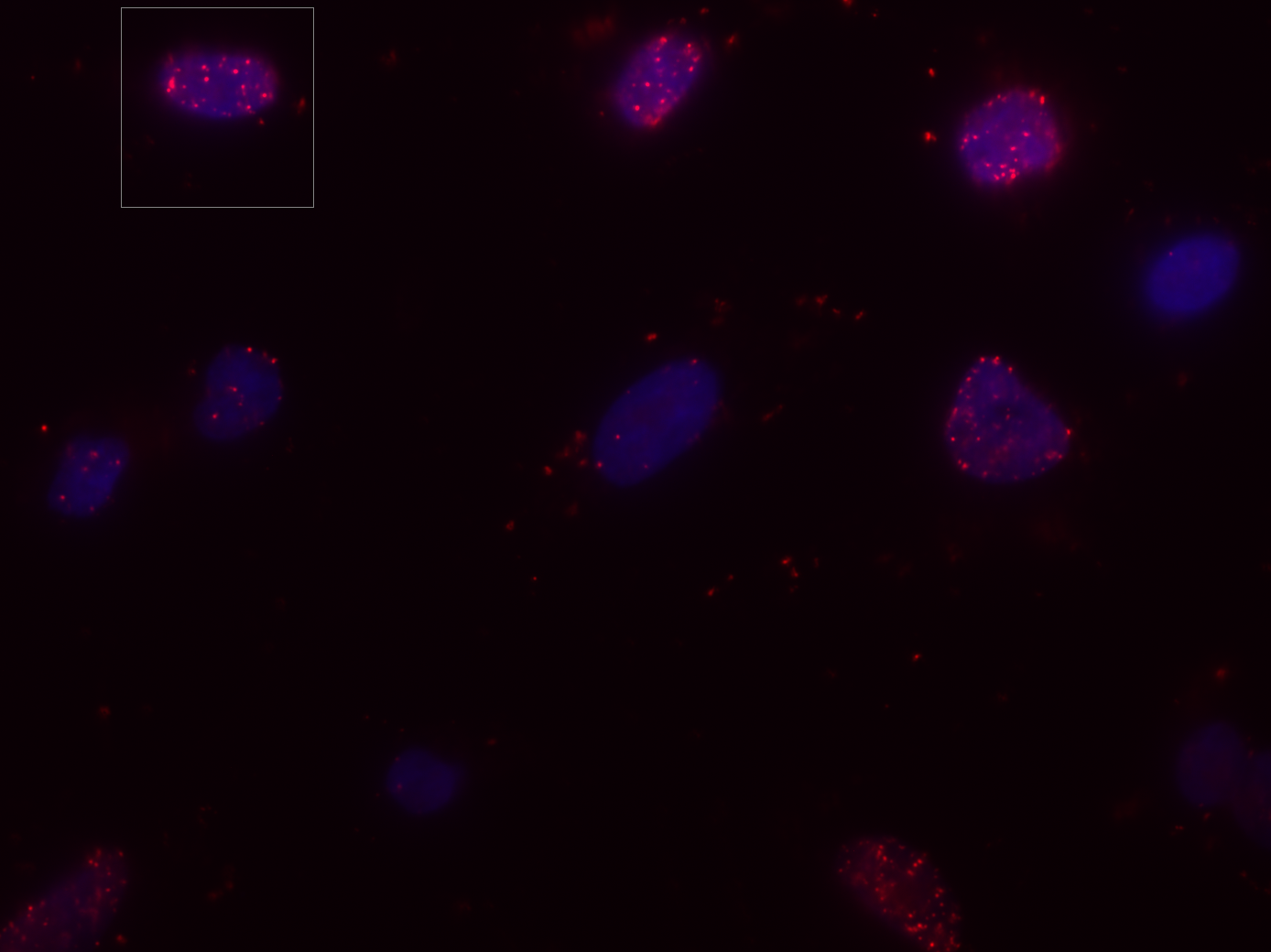

Supplement: Supplementary file 13 — Figure EV4 [file 44319_2025_497_MOESM13_ESM.zip › Figure EV 4E/shControl HU H3K4me3 SIRF.tif]

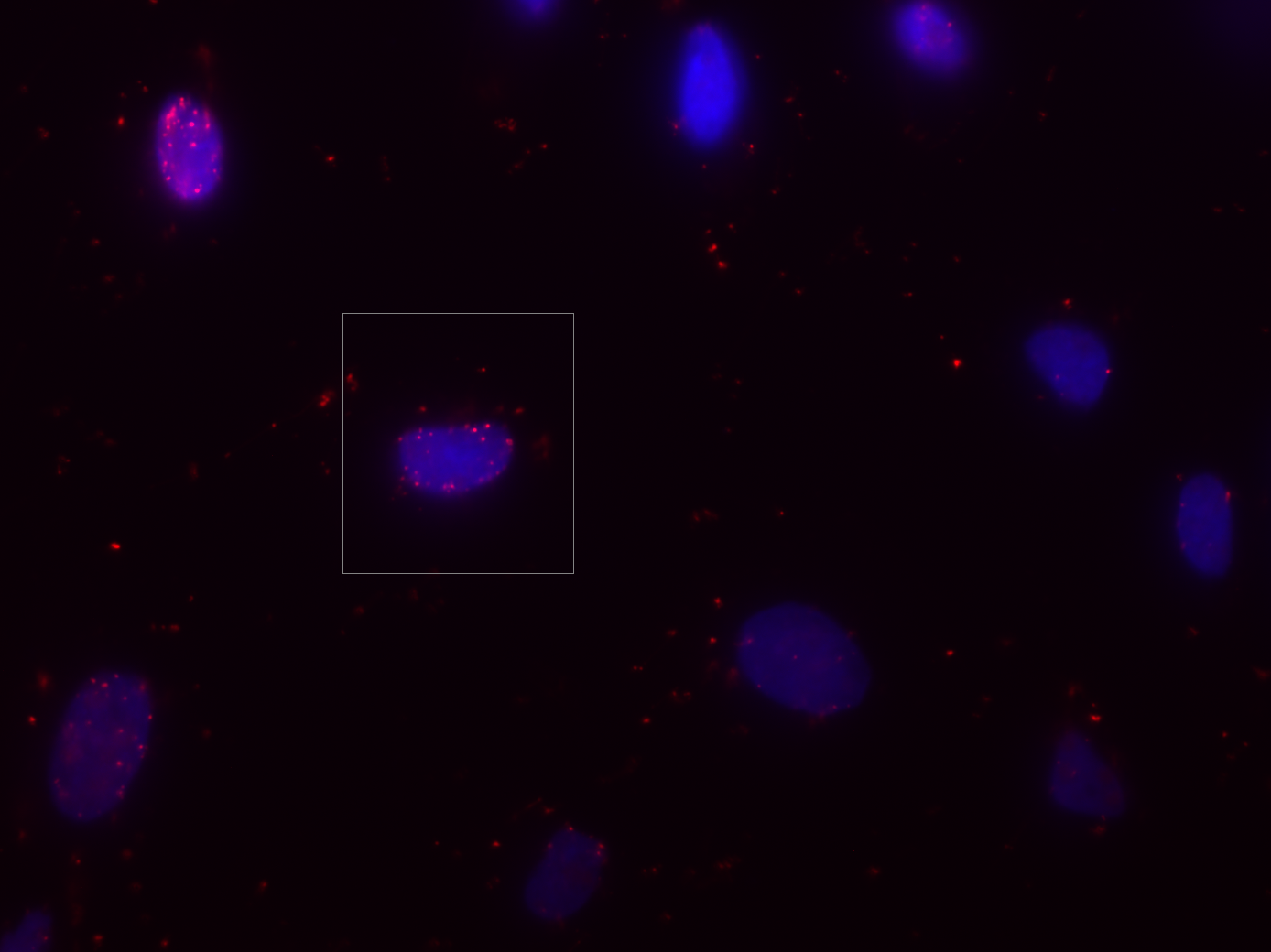

Supplement: Supplementary file 13 — Figure EV4 [file 44319_2025_497_MOESM13_ESM.zip › Figure EV 4E/shRNF20 HU H3K4me3 SIRF.tif]

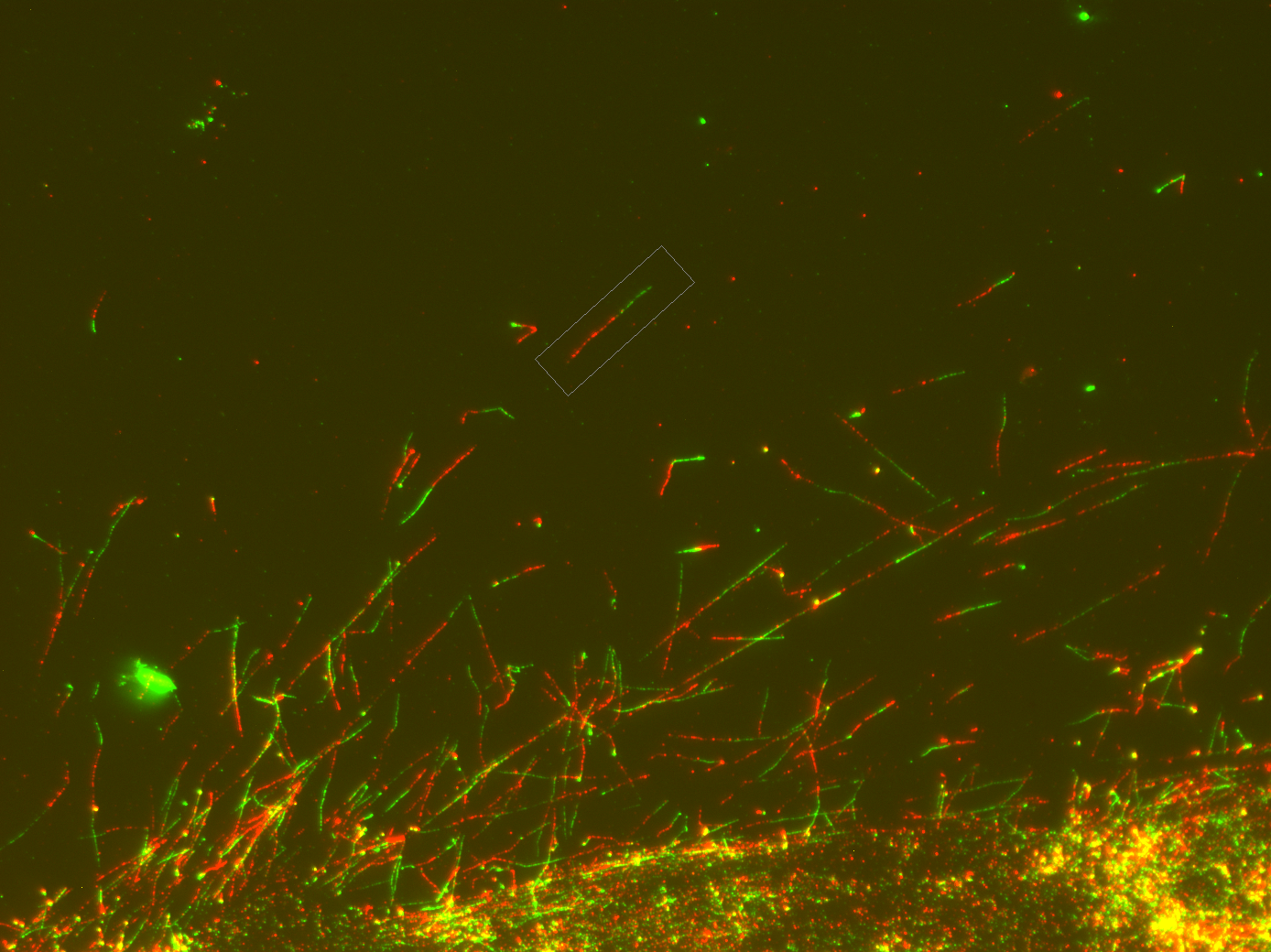

Supplement: Supplementary file 13 — Figure EV4 [file 44319_2025_497_MOESM13_ESM.zip › Figure EV 4H/shControl DNA fiber fork degradation.tif]

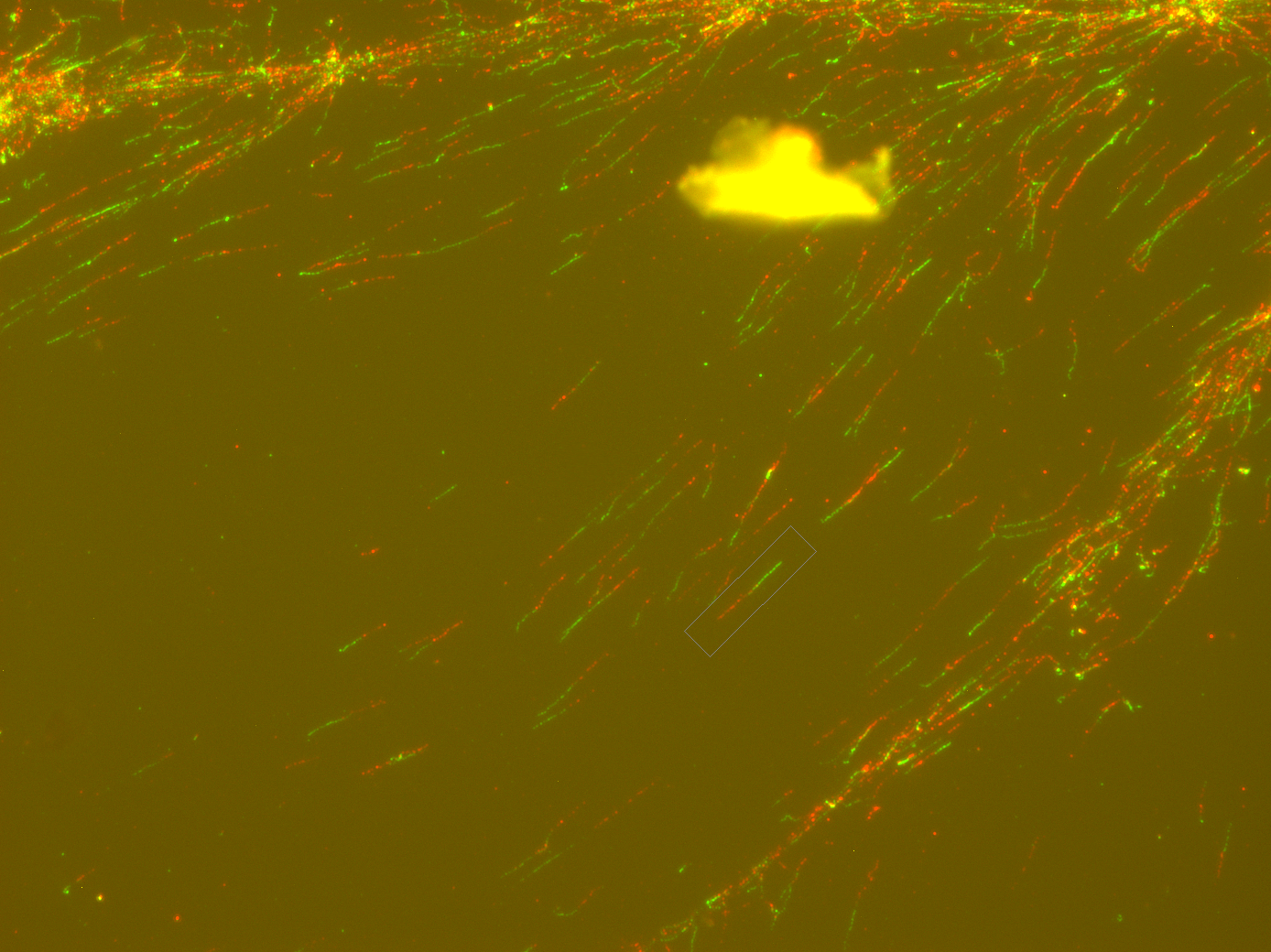

Supplement: Supplementary file 13 — Figure EV4 [file 44319_2025_497_MOESM13_ESM.zip › Figure EV 4H/shControl+Chloroquine DNA fiber fork degradation.tif]

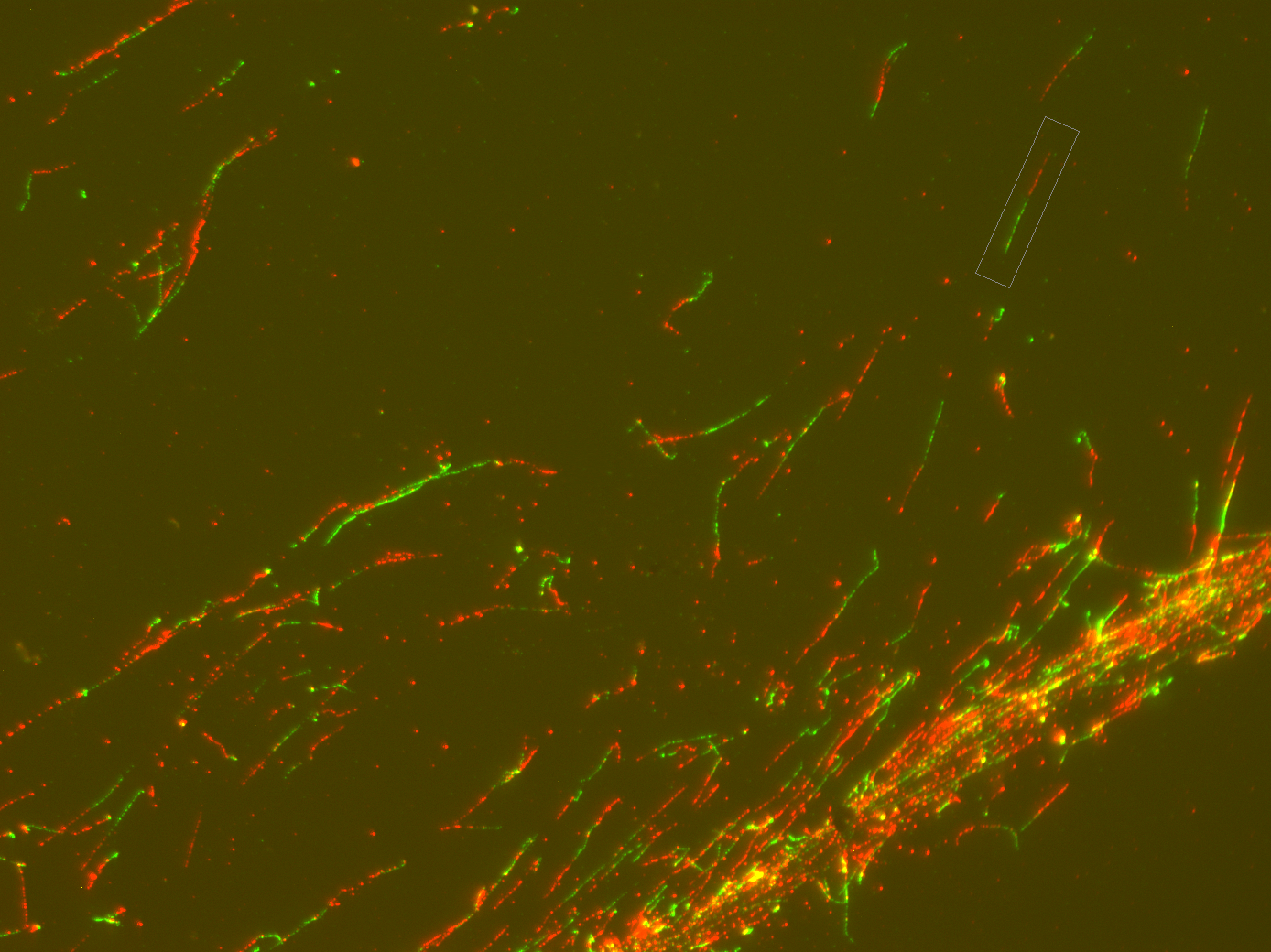

Supplement: Supplementary file 13 — Figure EV4 [file 44319_2025_497_MOESM13_ESM.zip › Figure EV 4H/shControl+TSA DNA fiber fork degradation.tif]

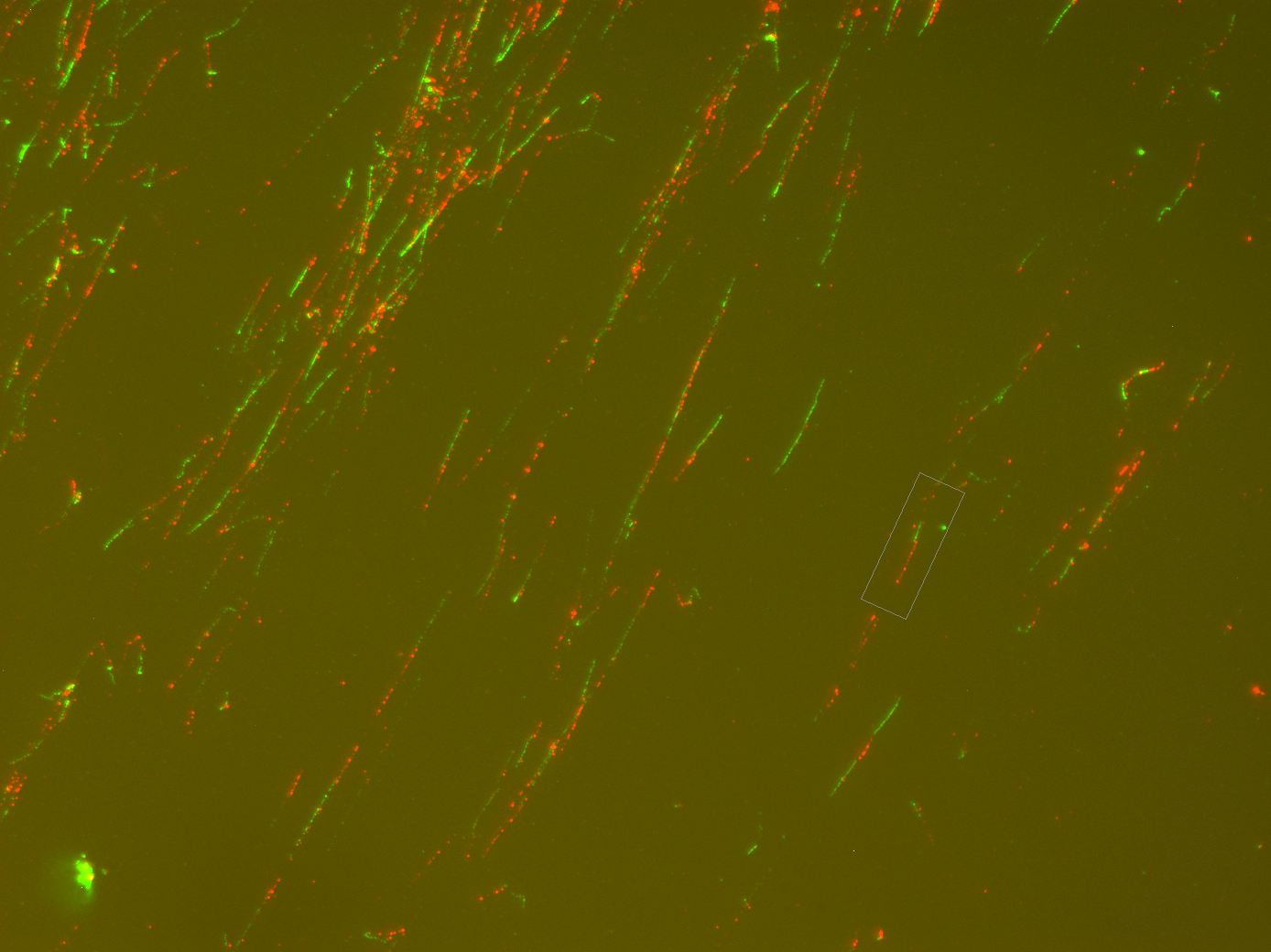

Supplement: Supplementary file 13 — Figure EV4 [file 44319_2025_497_MOESM13_ESM.zip › Figure EV 4H/shRNF20 DNA fiber fork degradation.tif]

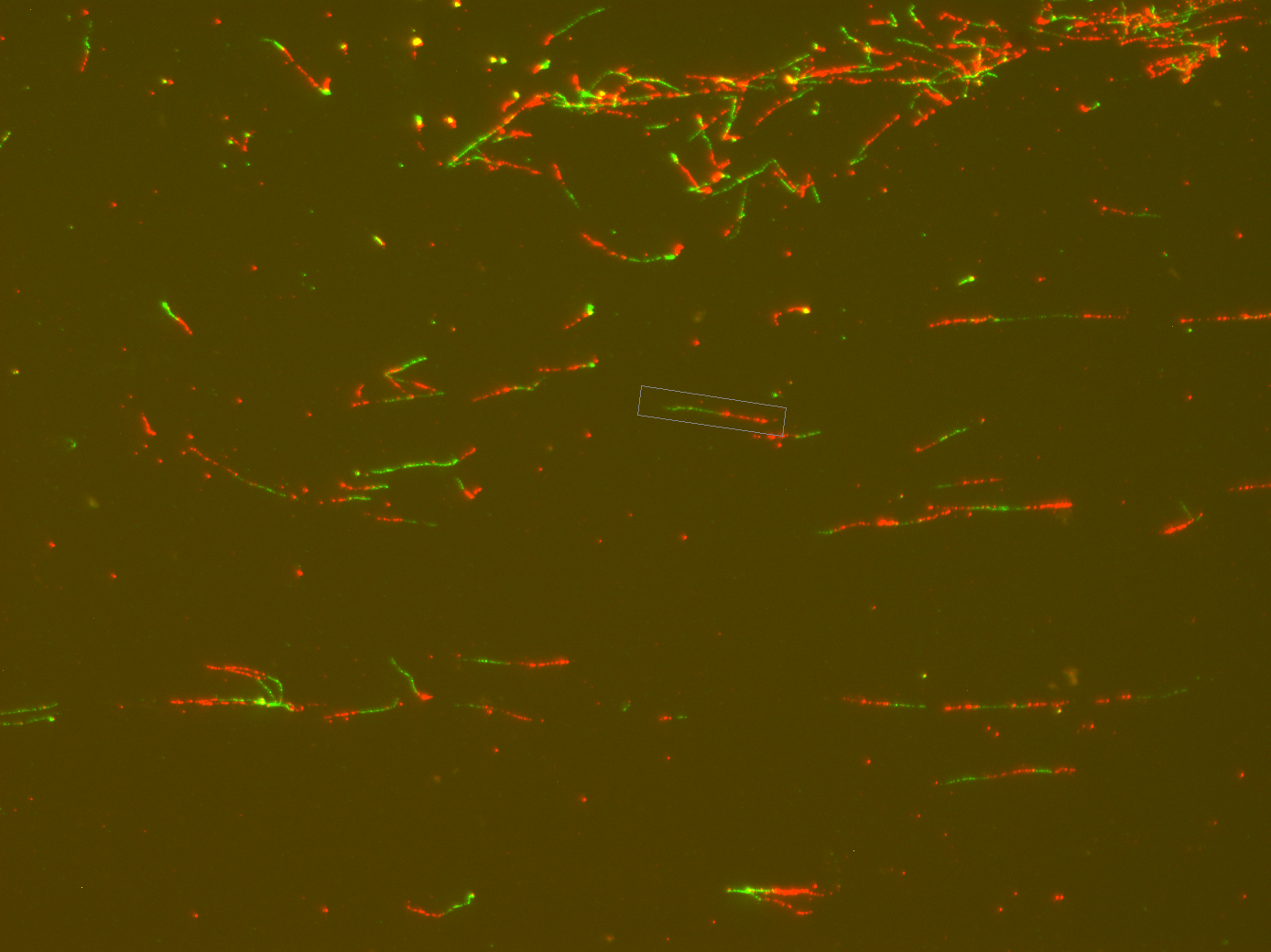

Supplement: Supplementary file 13 — Figure EV4 [file 44319_2025_497_MOESM13_ESM.zip › Figure EV 4H/shRNF20+Chloroquine DNA fiber fork degradation.tif]

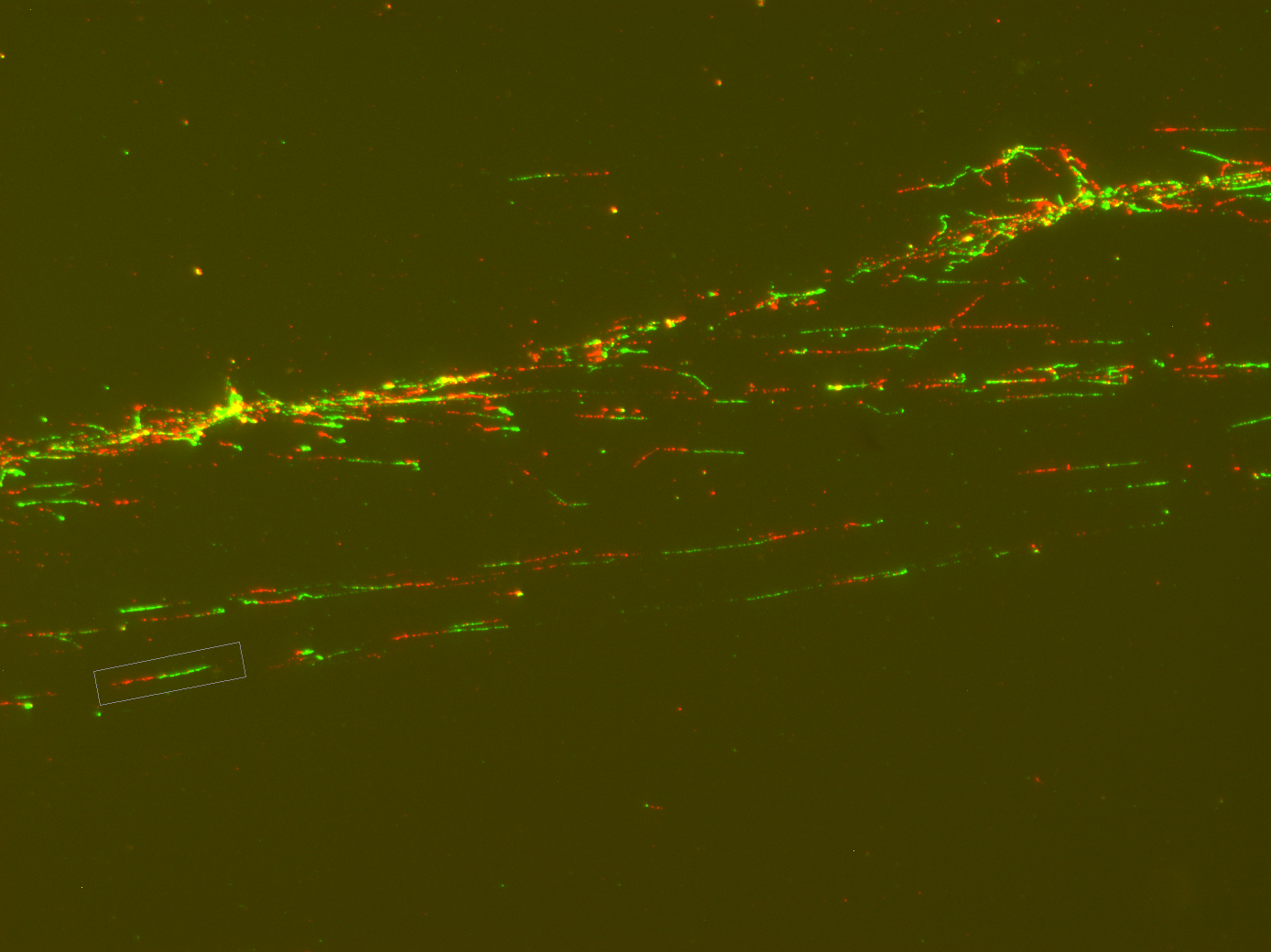

Supplement: Supplementary file 13 — Figure EV4 [file 44319_2025_497_MOESM13_ESM.zip › Figure EV 4H/shRNF20+TSA DNA fiber fork degradation.tif]

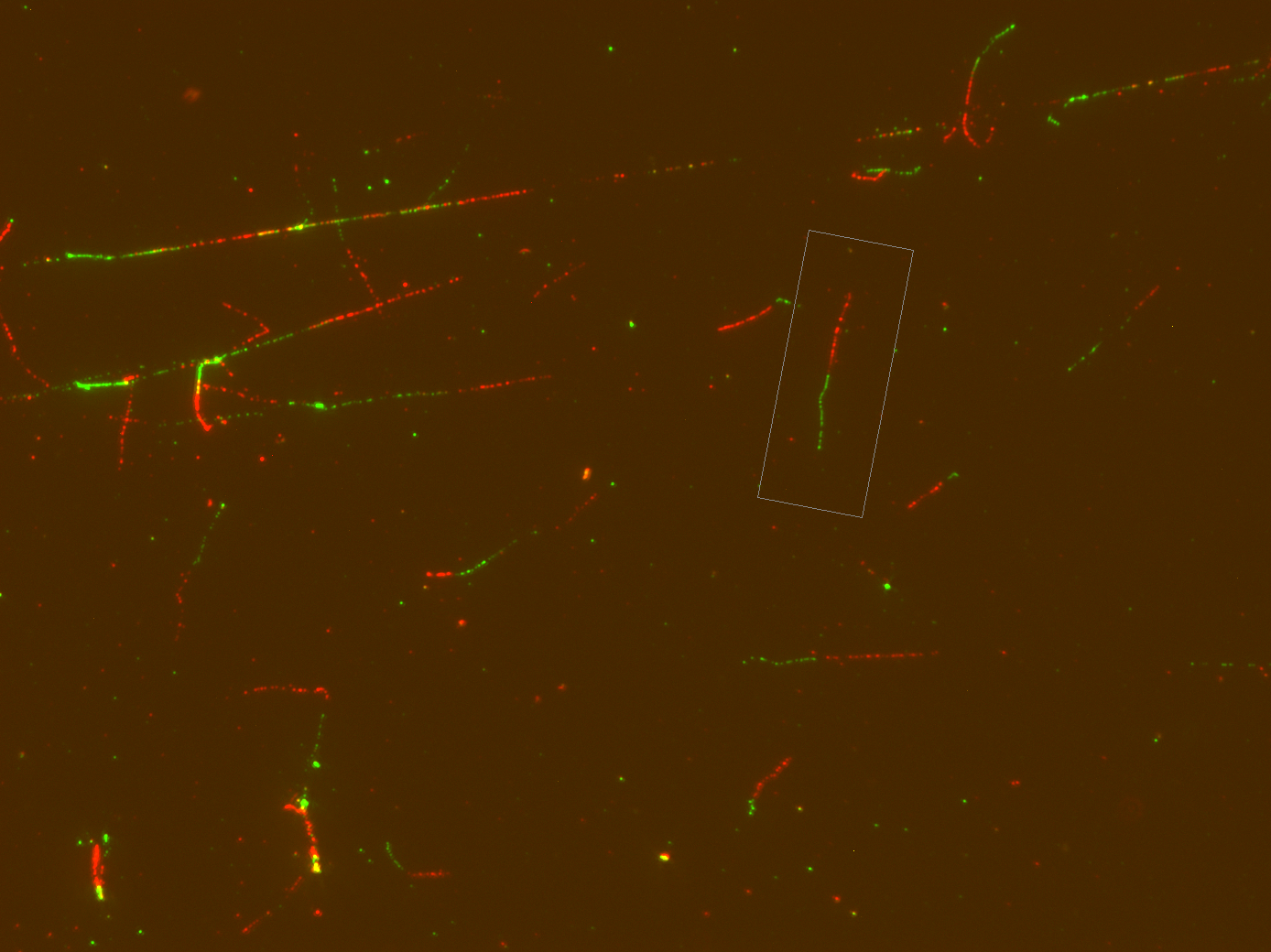

Supplement: Supplementary file 13 — Figure EV4 [file 44319_2025_497_MOESM13_ESM.zip › Figure EV 4I/shControl DNA fiber fork restart.tif]

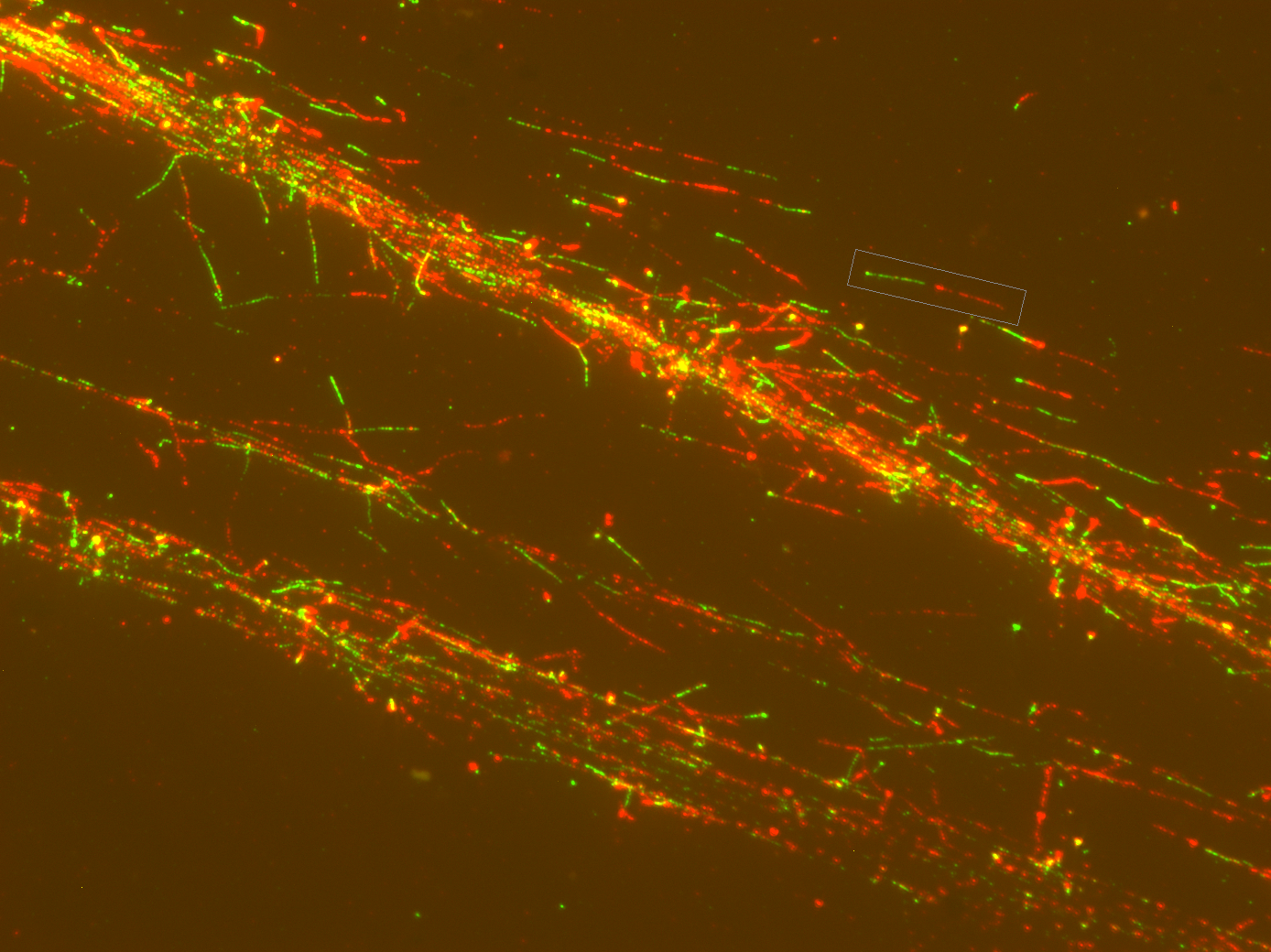

Supplement: Supplementary file 13 — Figure EV4 [file 44319_2025_497_MOESM13_ESM.zip › Figure EV 4I/shControl+chloroquine DNA fiber fork degradation.tif]

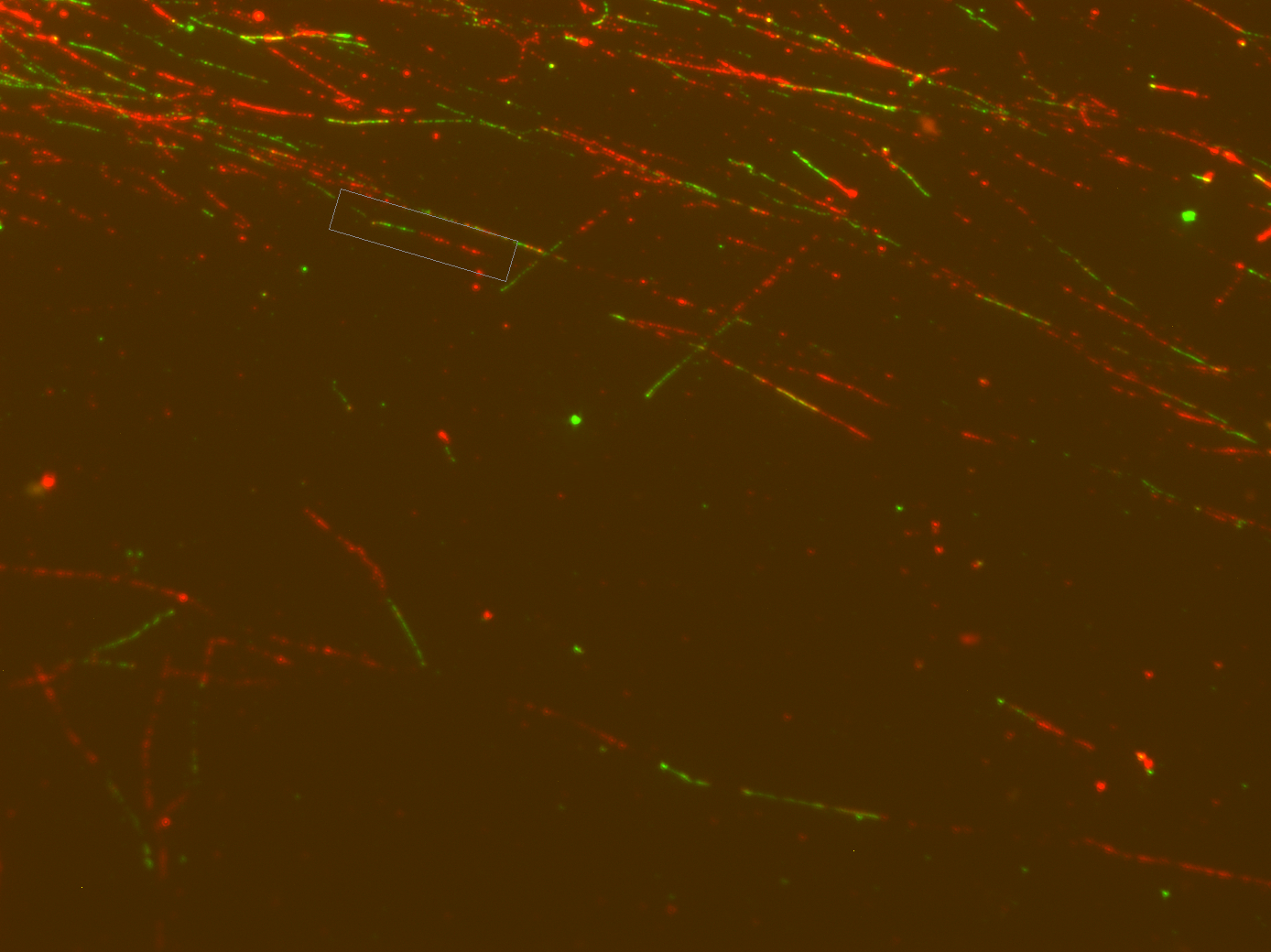

Supplement: Supplementary file 13 — Figure EV4 [file 44319_2025_497_MOESM13_ESM.zip › Figure EV 4I/shControl+TSA DNA fiber fork degradation.tif]

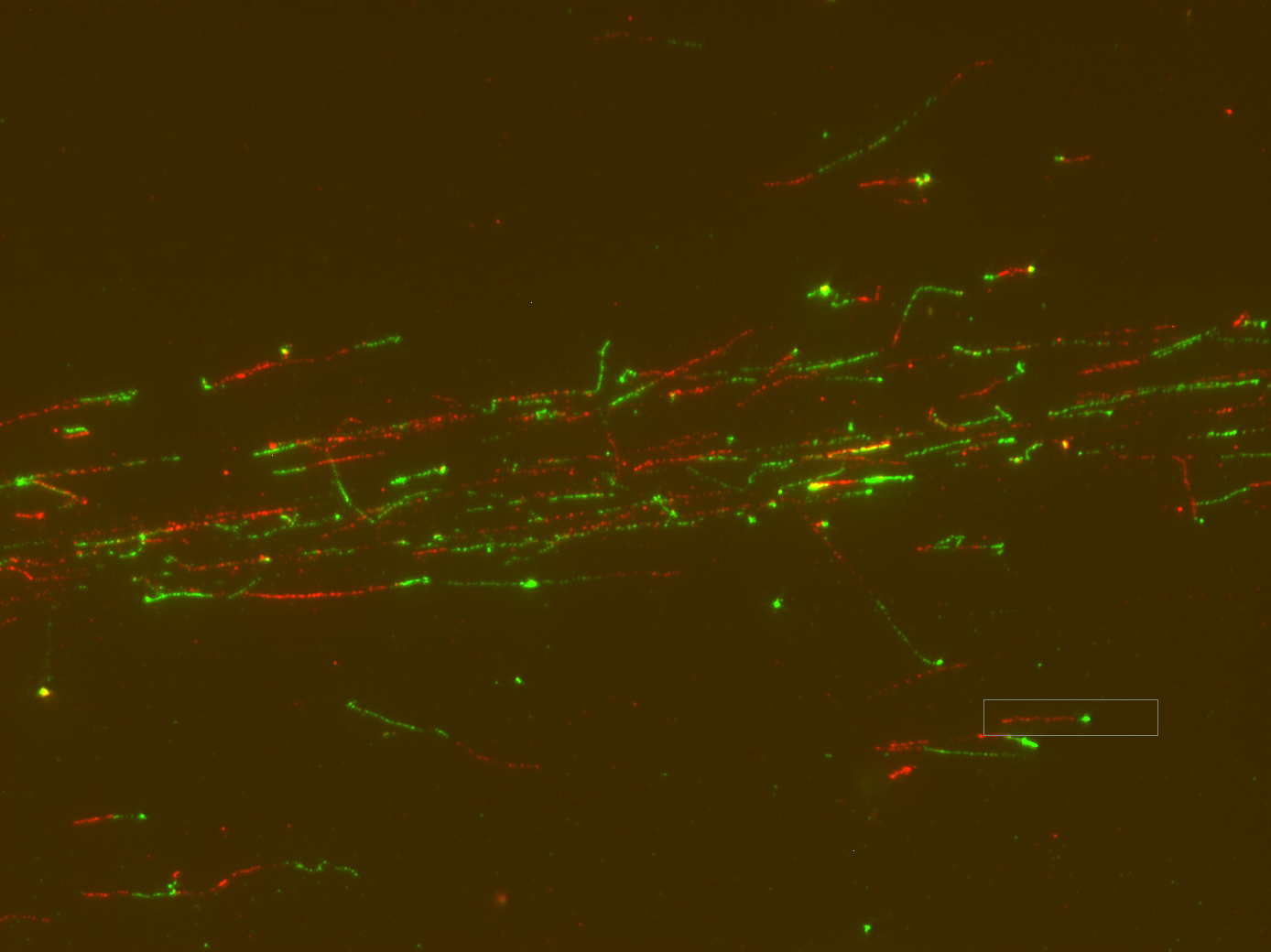

Supplement: Supplementary file 13 — Figure EV4 [file 44319_2025_497_MOESM13_ESM.zip › Figure EV 4I/shRNF20 DNA fiber fork restart.tif]

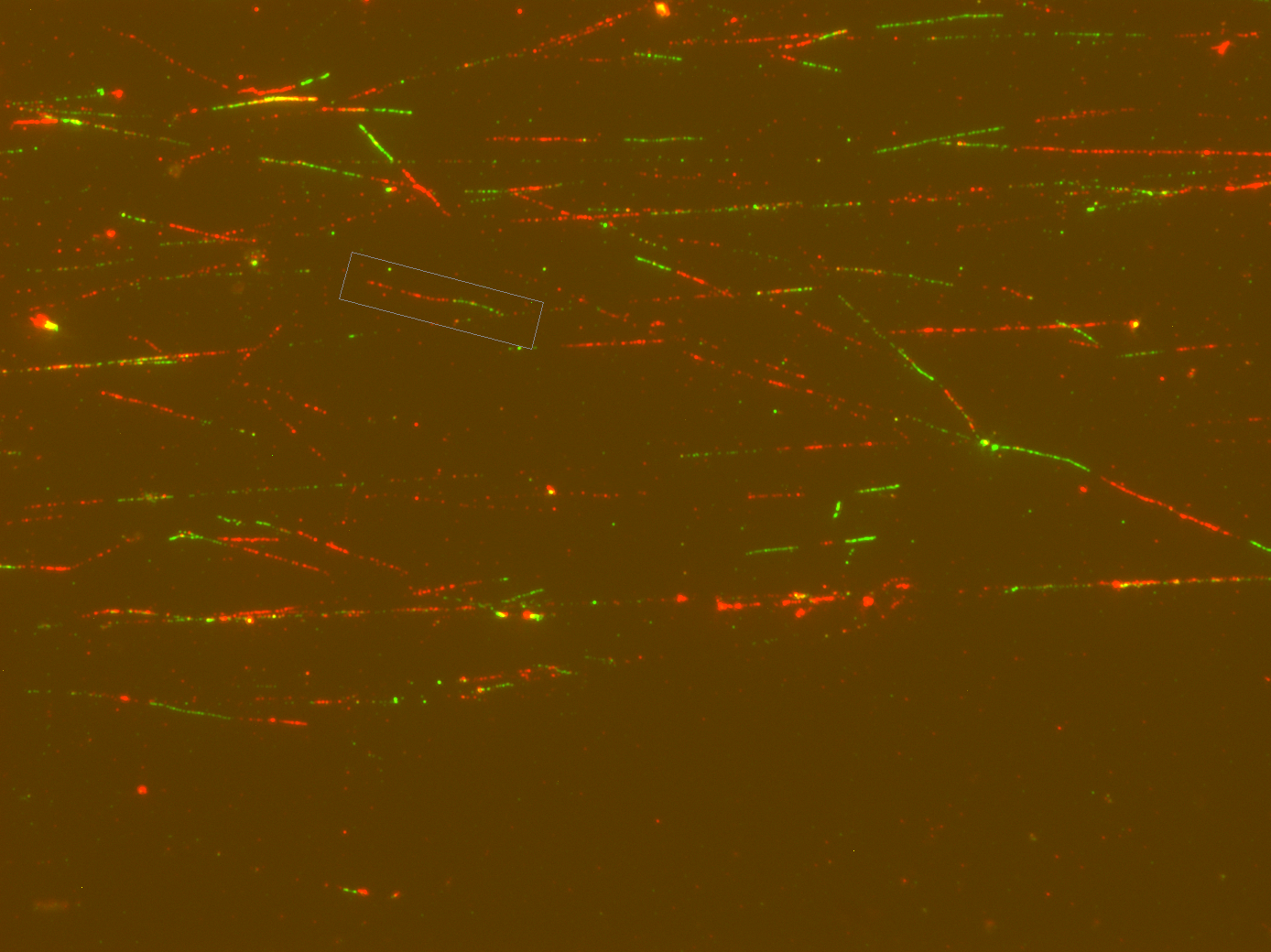

Supplement: Supplementary file 13 — Figure EV4 [file 44319_2025_497_MOESM13_ESM.zip › Figure EV 4I/shRNF20+Chloroquine DNA fiber fork degradation.tif]
