# Supplementary figures and images for: RNF20-mediated H2B monoubiquitination protects stalled forks from degradation and promotes fork restart (part 3 of 3)
Source: EMBO Rep. 2025 Jun 10;26(15):3773–803. doi: 10.1038/s44319-025-00497-3 (PMC12331980; doi:10.1038/s44319-025-00497-3)

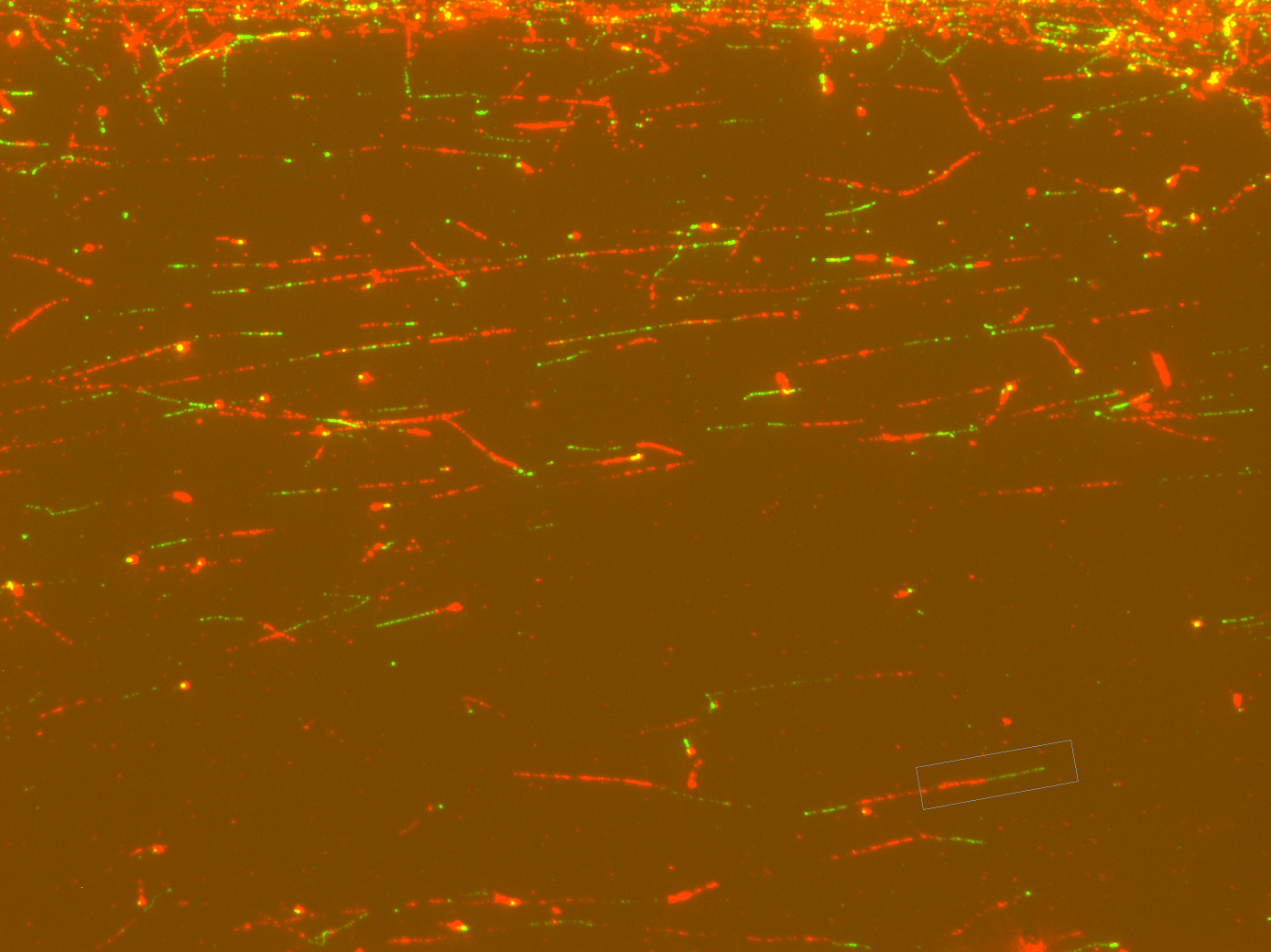

Supplement: Supplementary file 13 — Figure EV4 [file 44319_2025_497_MOESM13_ESM.zip › Figure EV 4I/shRNF20+TSA DNA fiber fork degradation.tif]

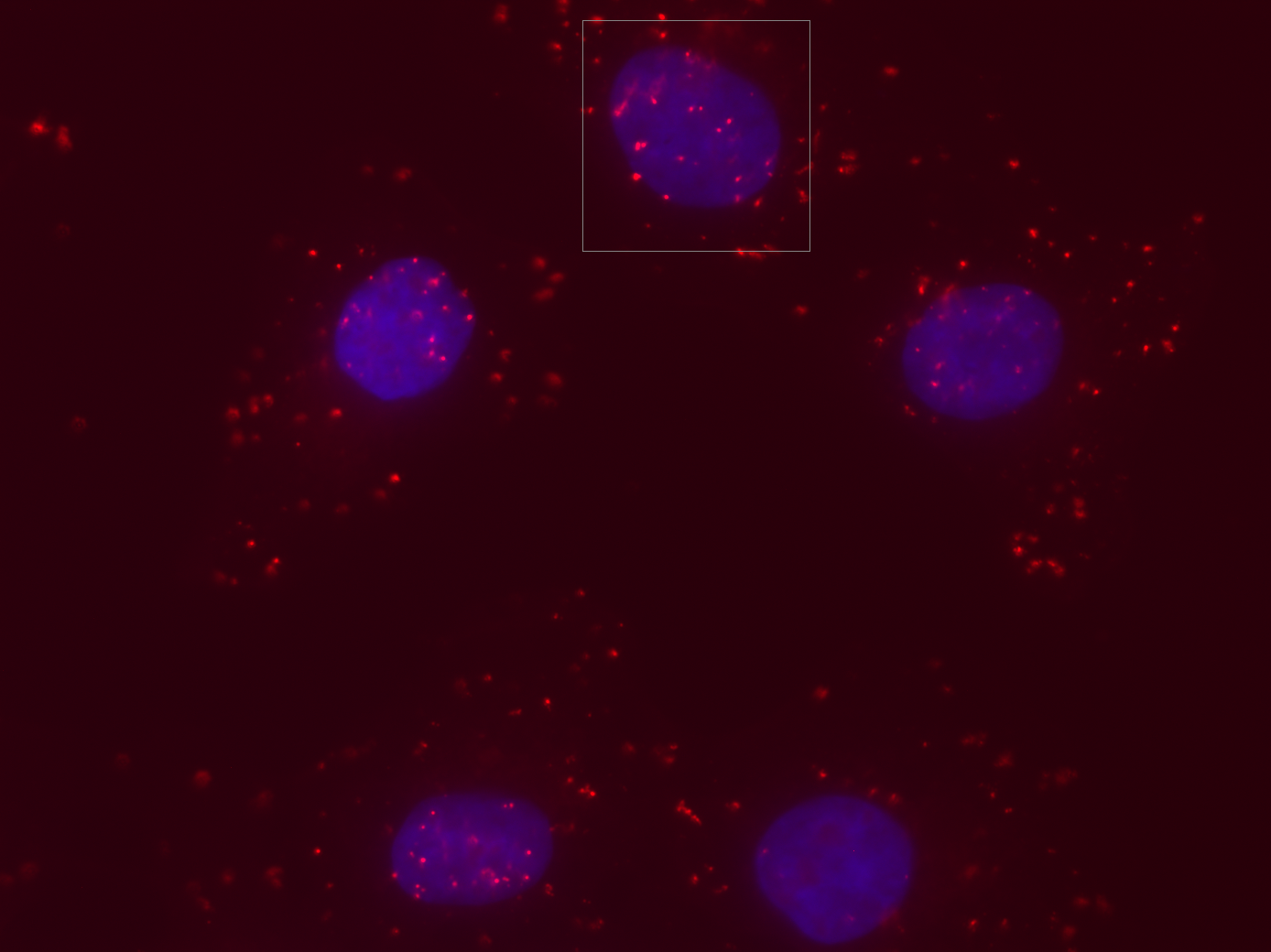

Supplement: Supplementary file 14 — Figure EV5 [file 44319_2025_497_MOESM14_ESM.zip › Figure EV 5A/shControl RAD51 SIRF.tif]

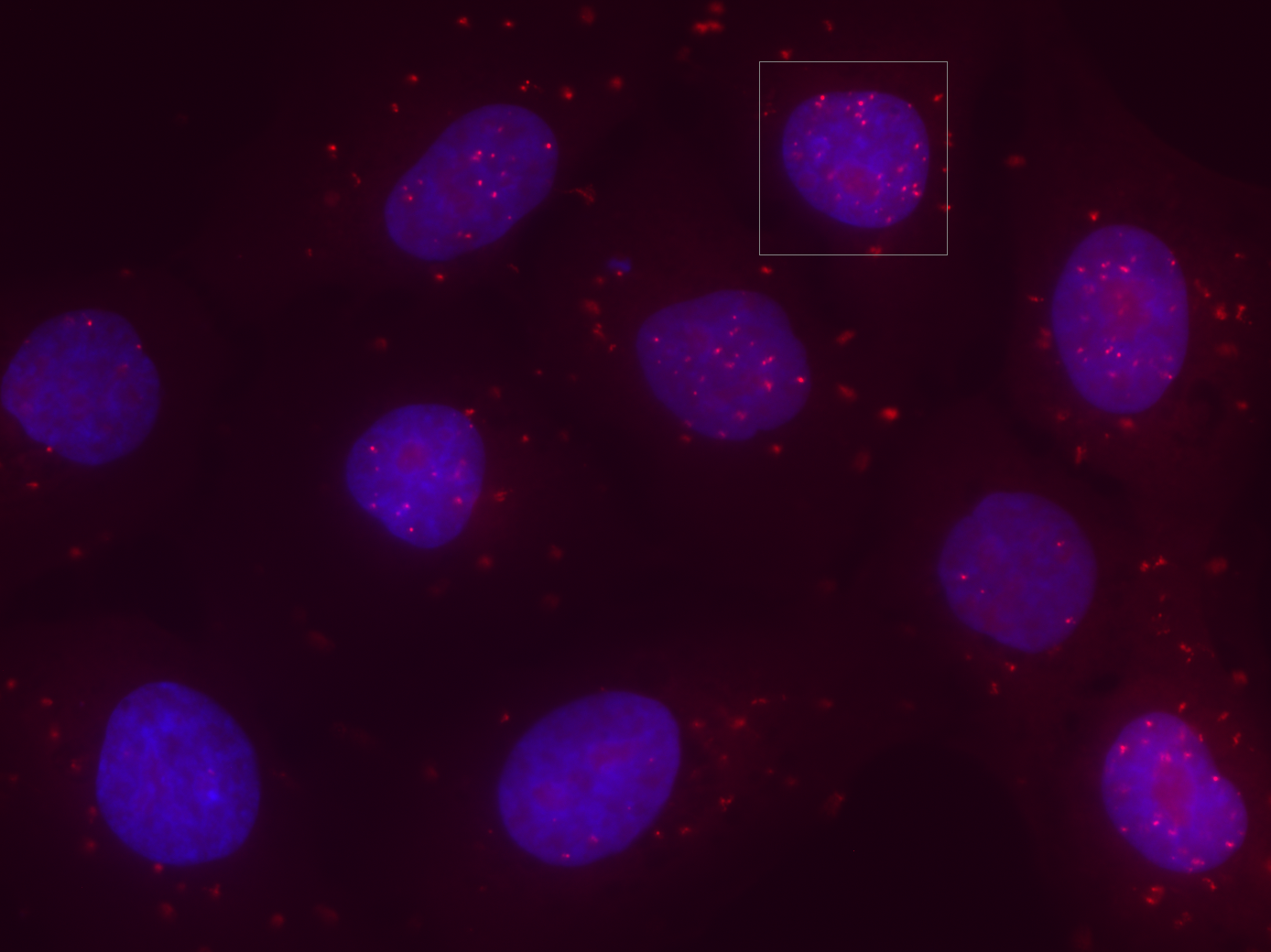

Supplement: Supplementary file 14 — Figure EV5 [file 44319_2025_497_MOESM14_ESM.zip › Figure EV 5A/shControl+chloroquine RAD51 SIRF.tif]

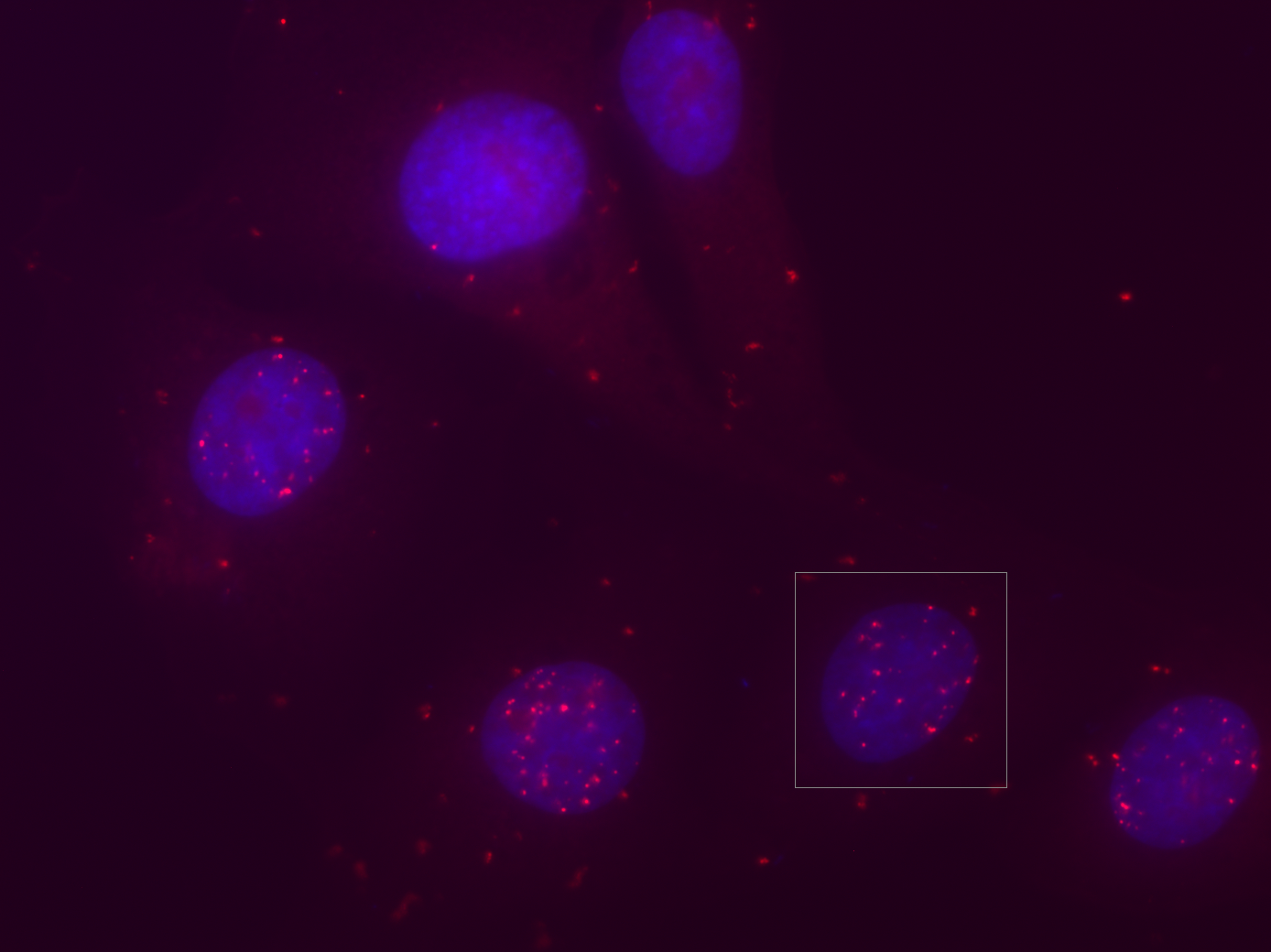

Supplement: Supplementary file 14 — Figure EV5 [file 44319_2025_497_MOESM14_ESM.zip › Figure EV 5A/shControl+TSA RAD51 SIRF.tif]

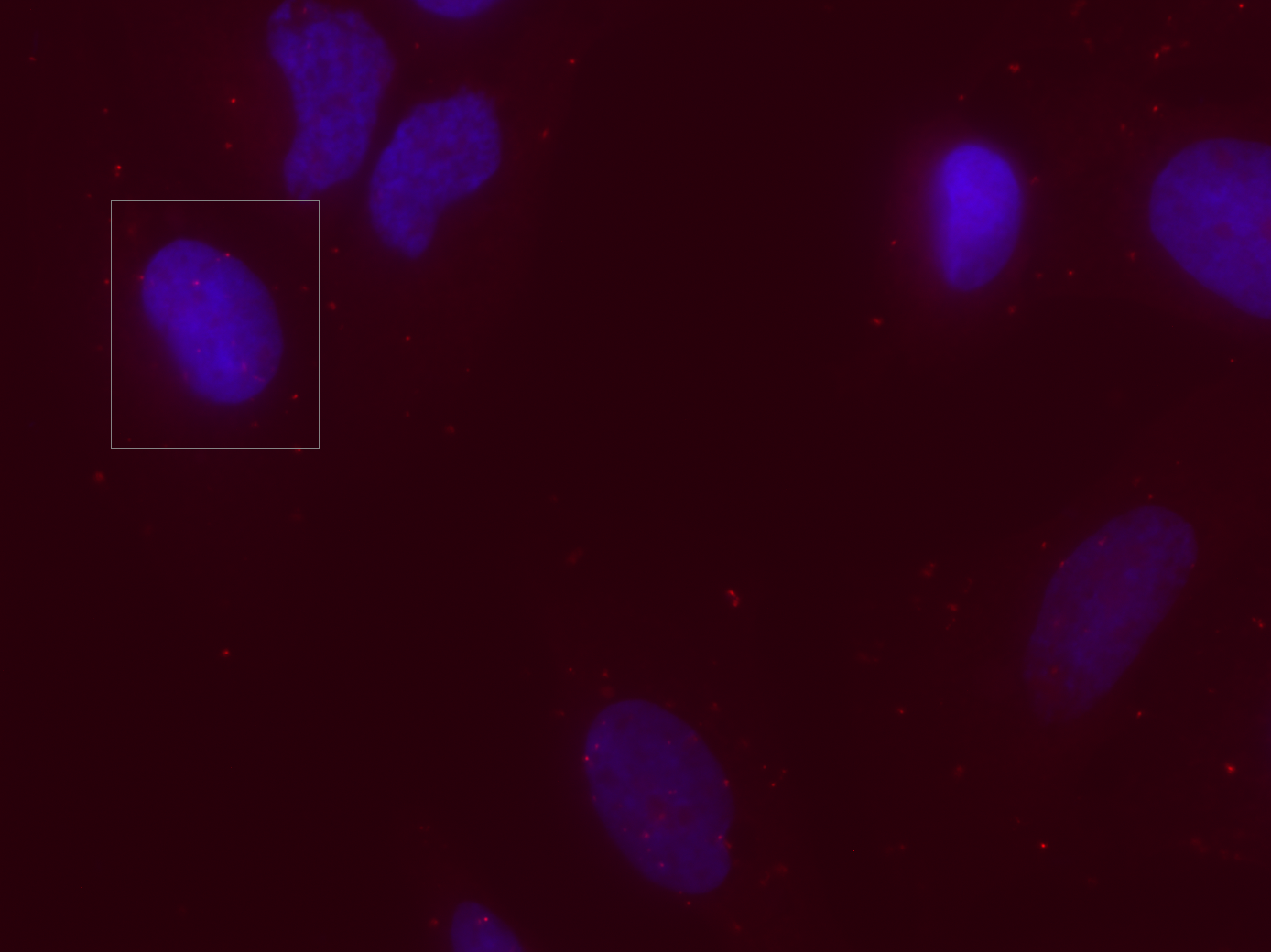

Supplement: Supplementary file 14 — Figure EV5 [file 44319_2025_497_MOESM14_ESM.zip › Figure EV 5A/shRNF20 RAD51 SIRF.tif]

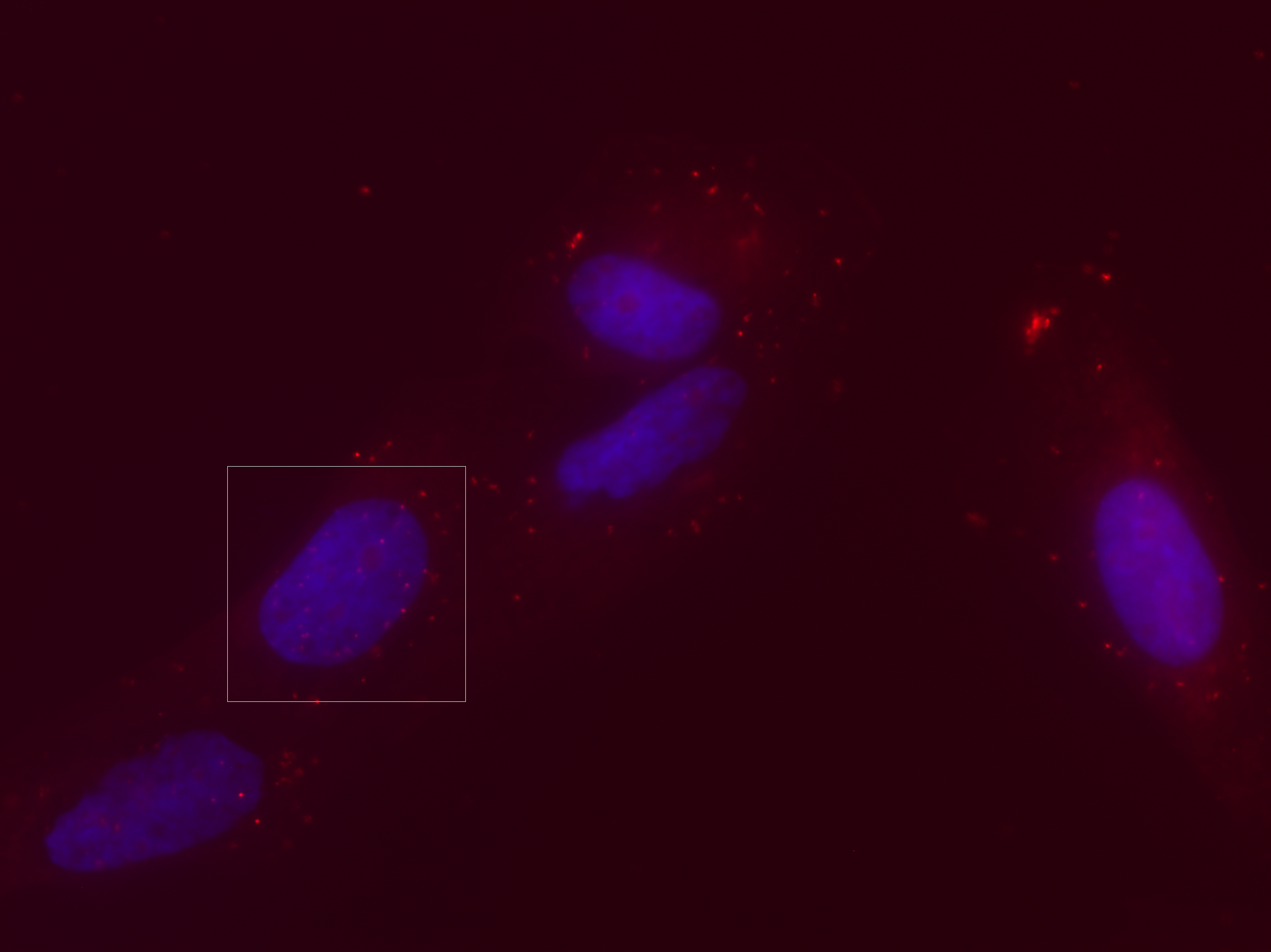

Supplement: Supplementary file 14 — Figure EV5 [file 44319_2025_497_MOESM14_ESM.zip › Figure EV 5A/shRNF20+chloroquine RAD51 SIRF.tif]

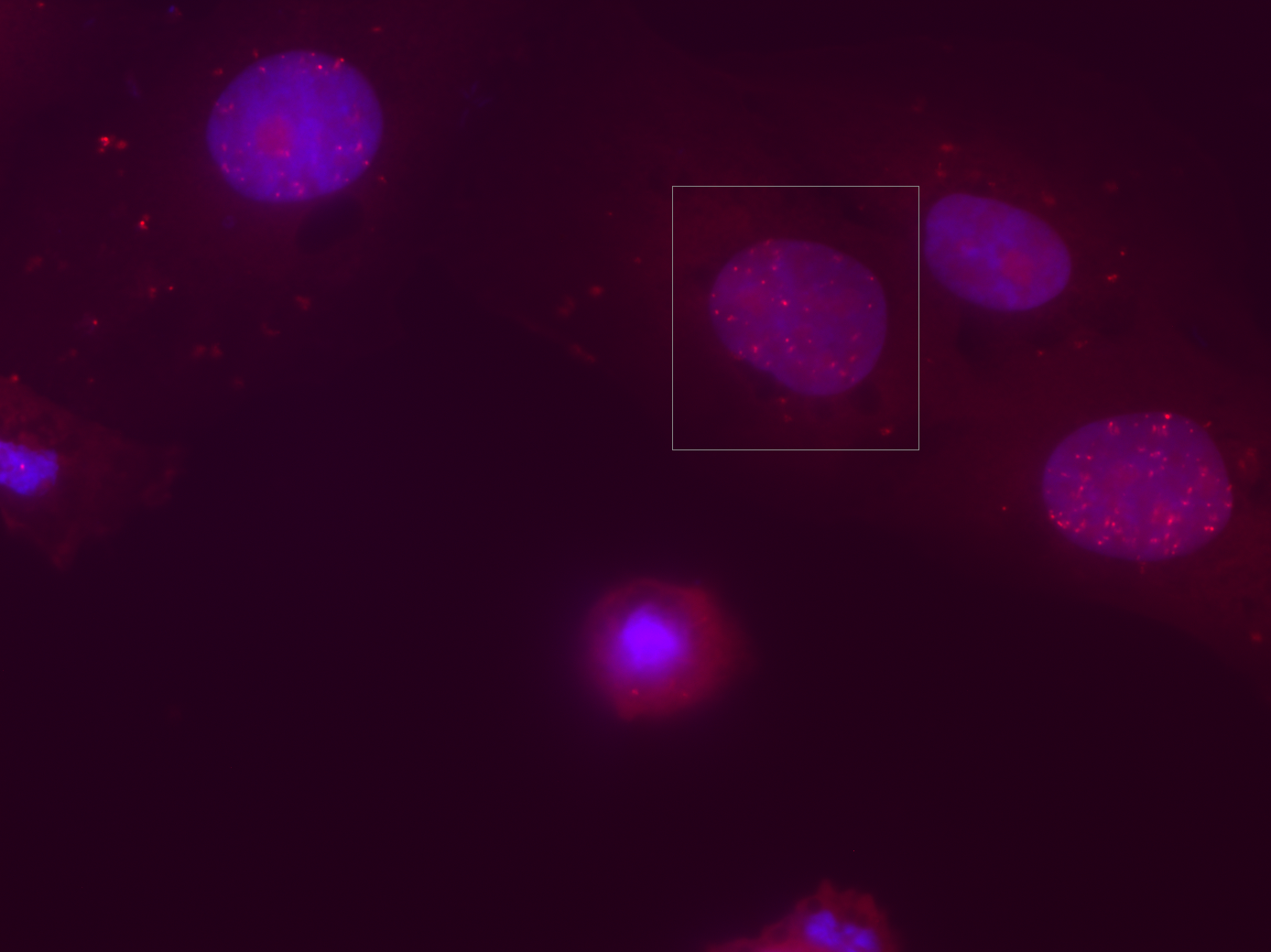

Supplement: Supplementary file 14 — Figure EV5 [file 44319_2025_497_MOESM14_ESM.zip › Figure EV 5A/shRNF20+TSA RAD51 SIRF.tif]

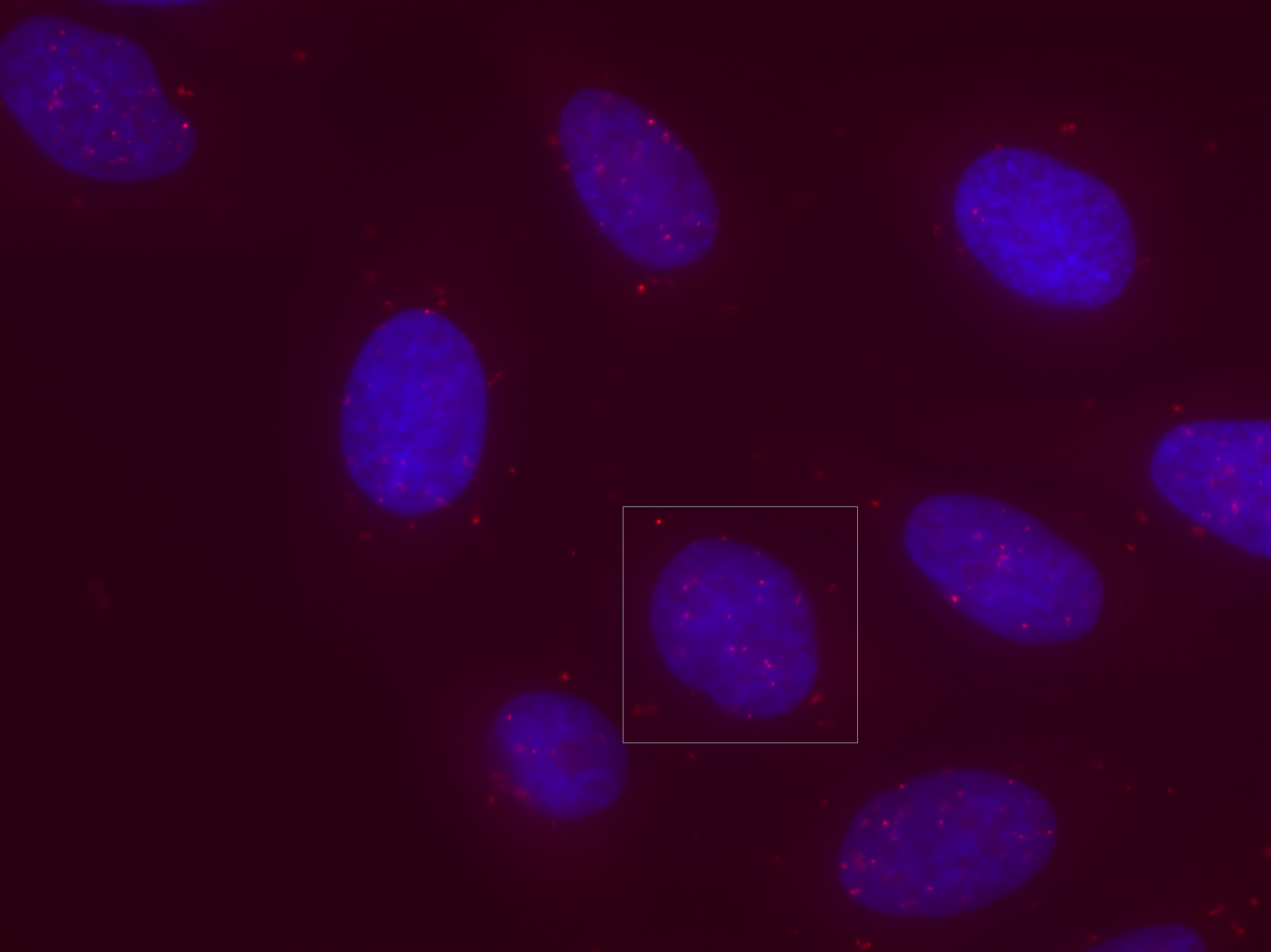

Supplement: Supplementary file 14 — Figure EV5 [file 44319_2025_497_MOESM14_ESM.zip › Figure EV 5C/shControl RAD51C SIRF.tif]

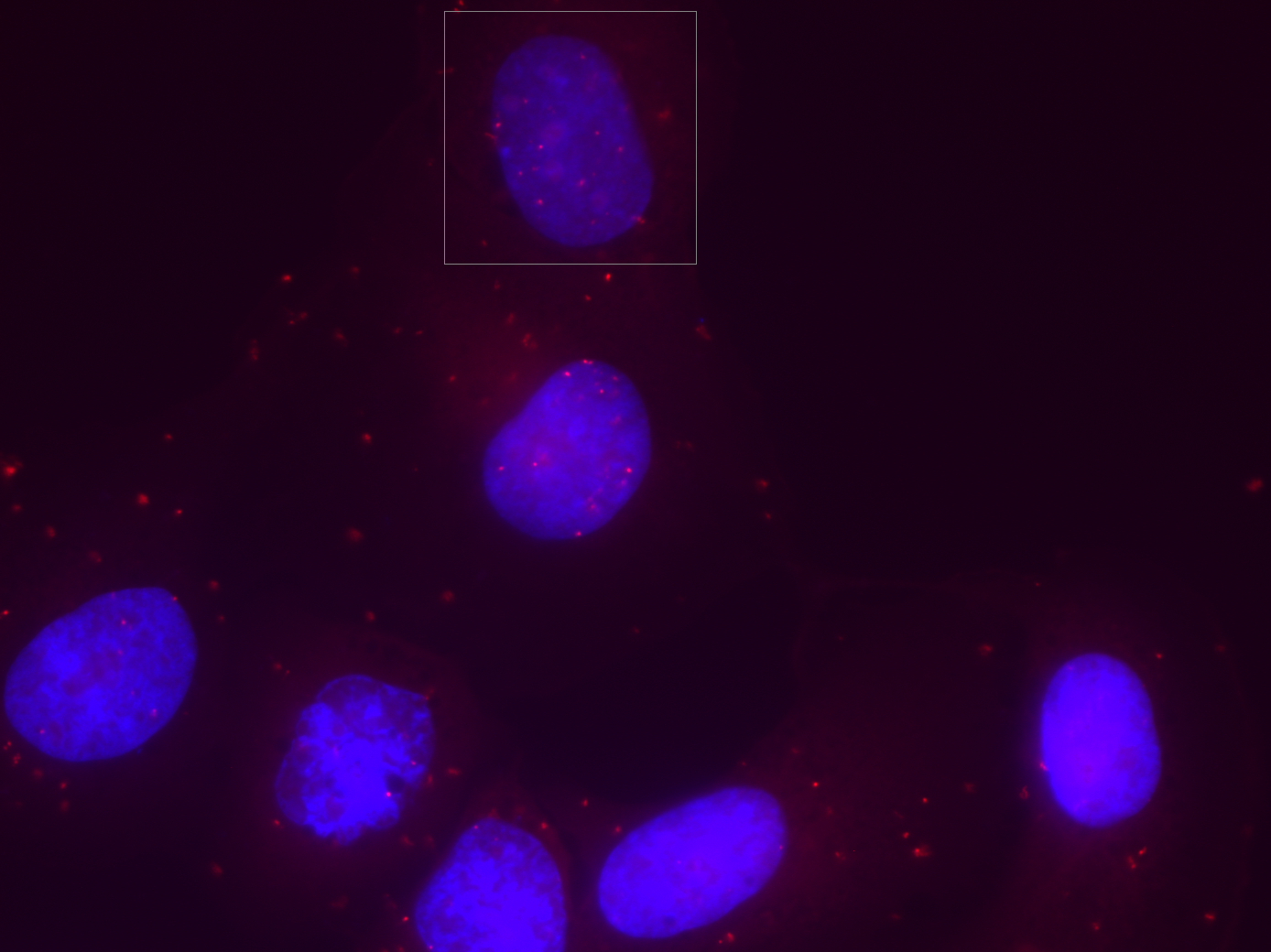

Supplement: Supplementary file 14 — Figure EV5 [file 44319_2025_497_MOESM14_ESM.zip › Figure EV 5C/shControl+chloroquine RAD51C SIRF.tif]

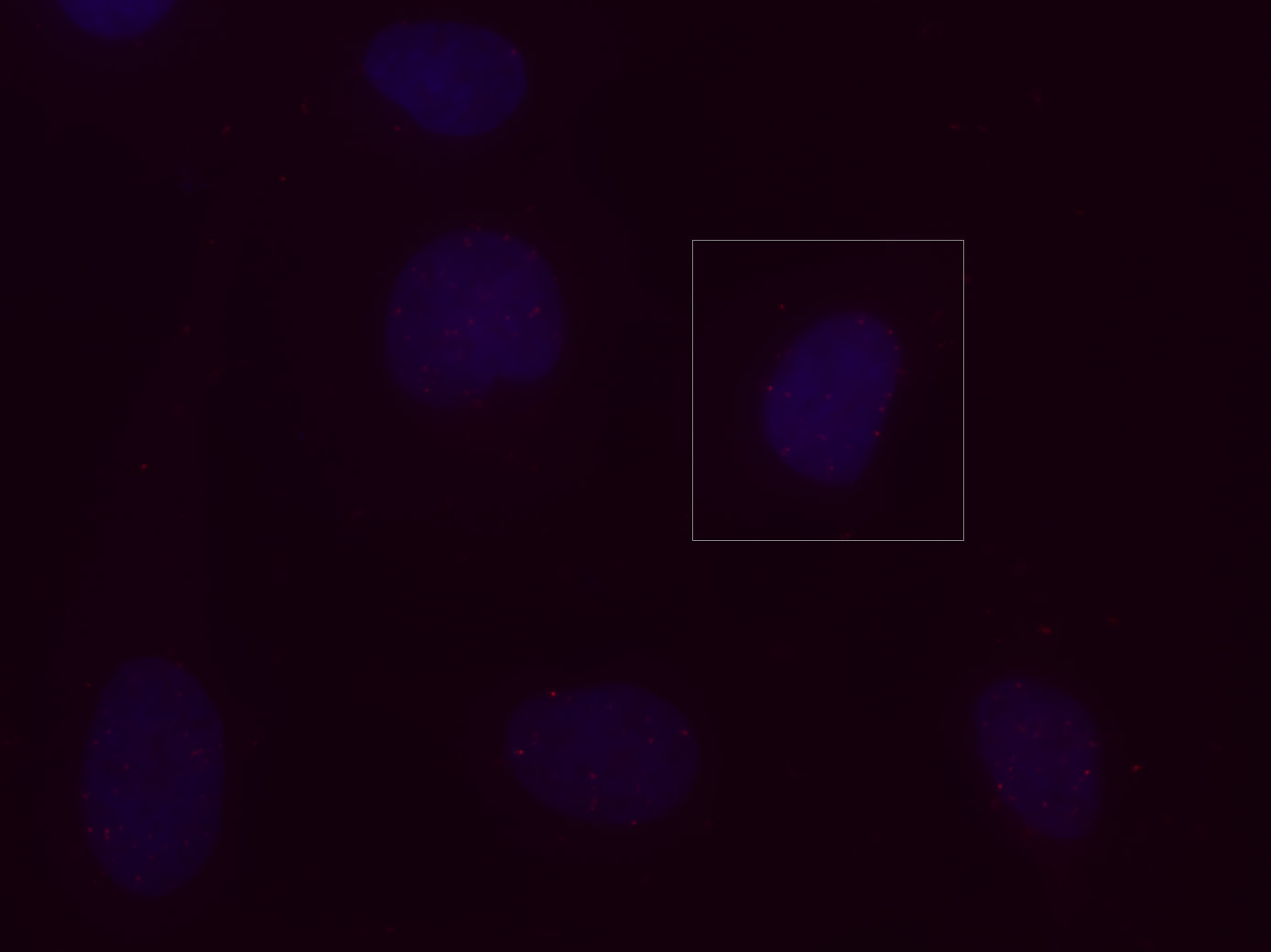

Supplement: Supplementary file 14 — Figure EV5 [file 44319_2025_497_MOESM14_ESM.zip › Figure EV 5C/shControl+TSA RAD51C SIRF.tif]

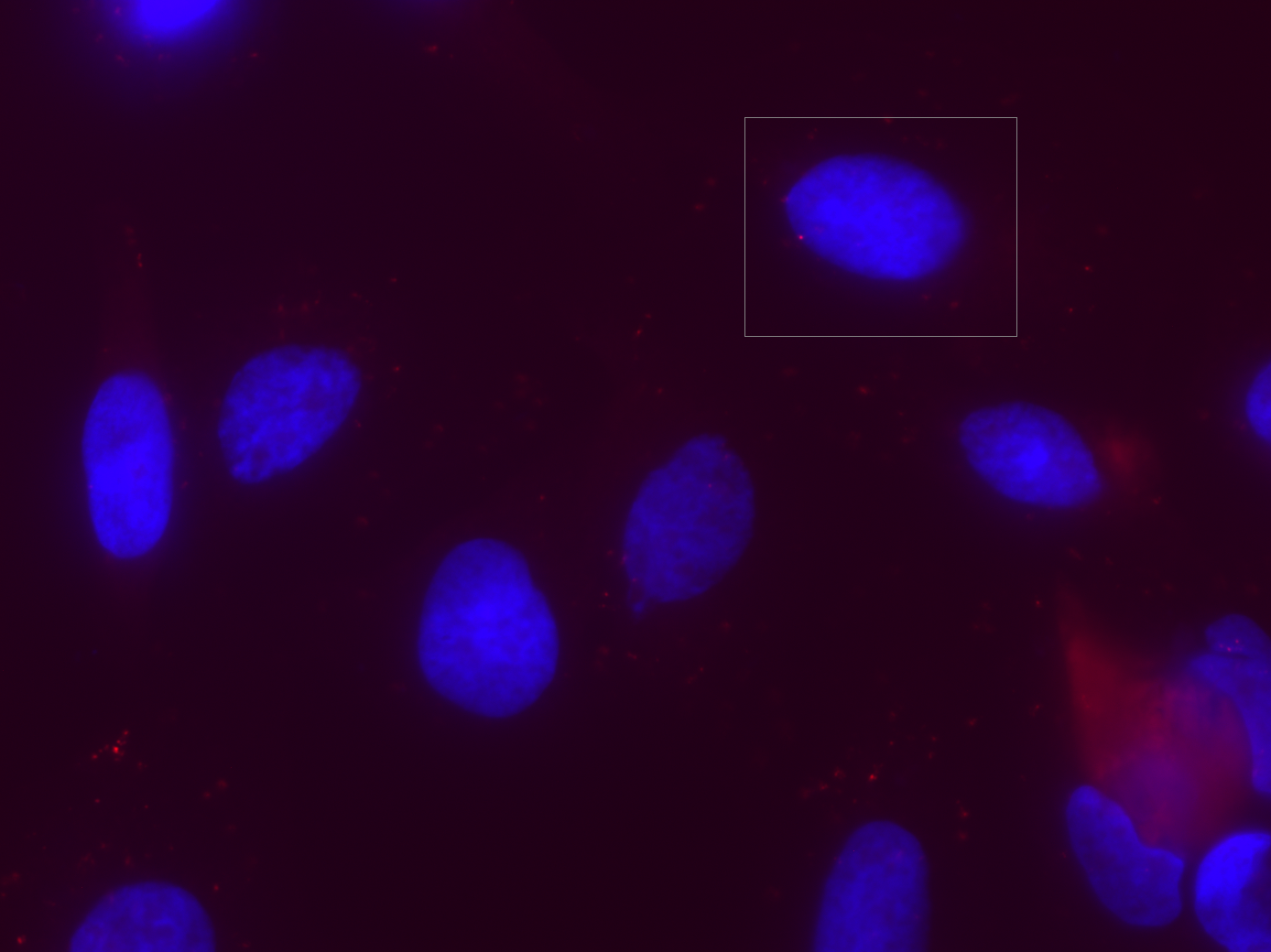

Supplement: Supplementary file 14 — Figure EV5 [file 44319_2025_497_MOESM14_ESM.zip › Figure EV 5C/shRNF20 RAD51C SIRF.tif]

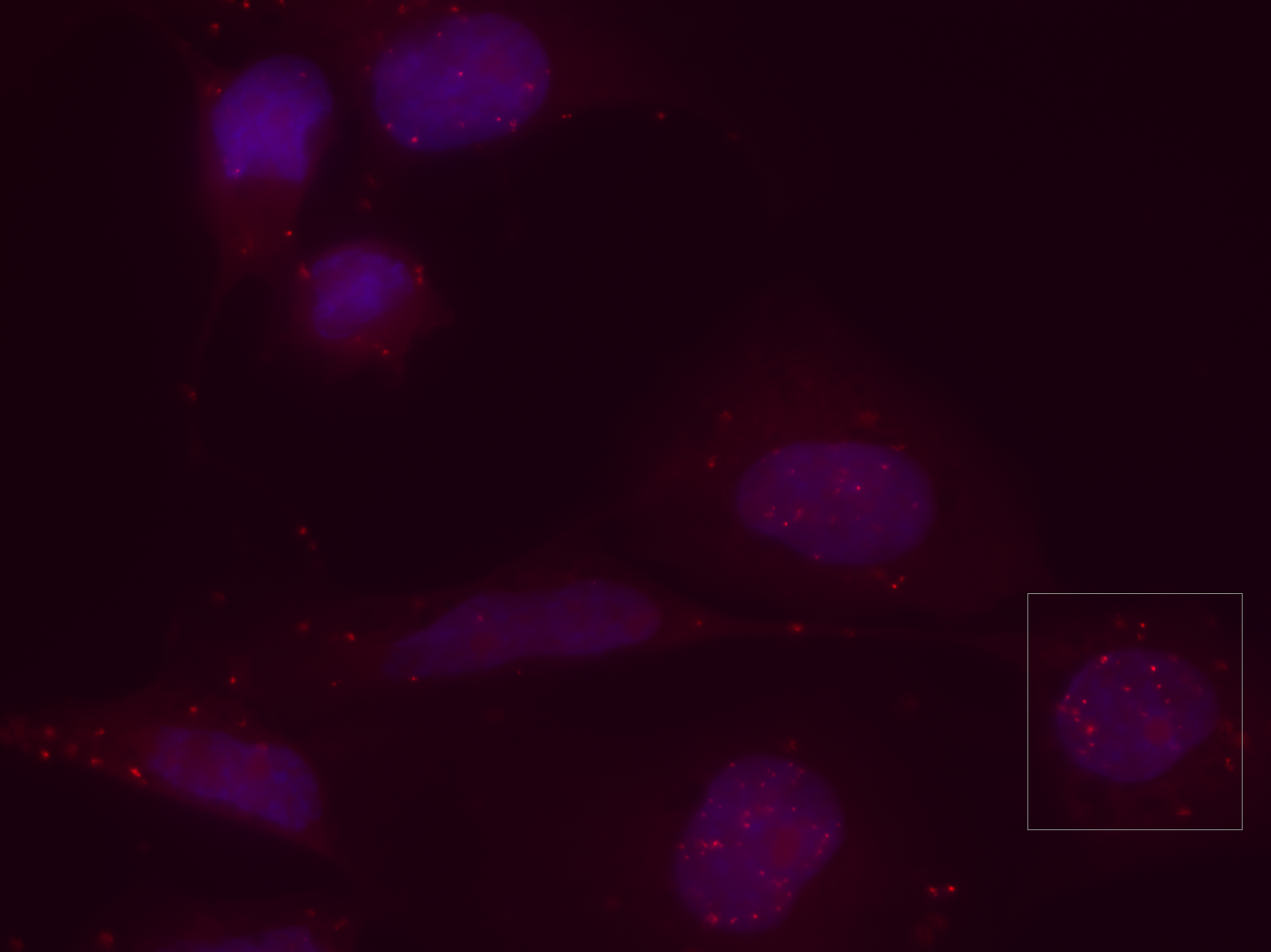

Supplement: Supplementary file 14 — Figure EV5 [file 44319_2025_497_MOESM14_ESM.zip › Figure EV 5C/shRNF20+chloroquine RAD51C SIRF.tif]

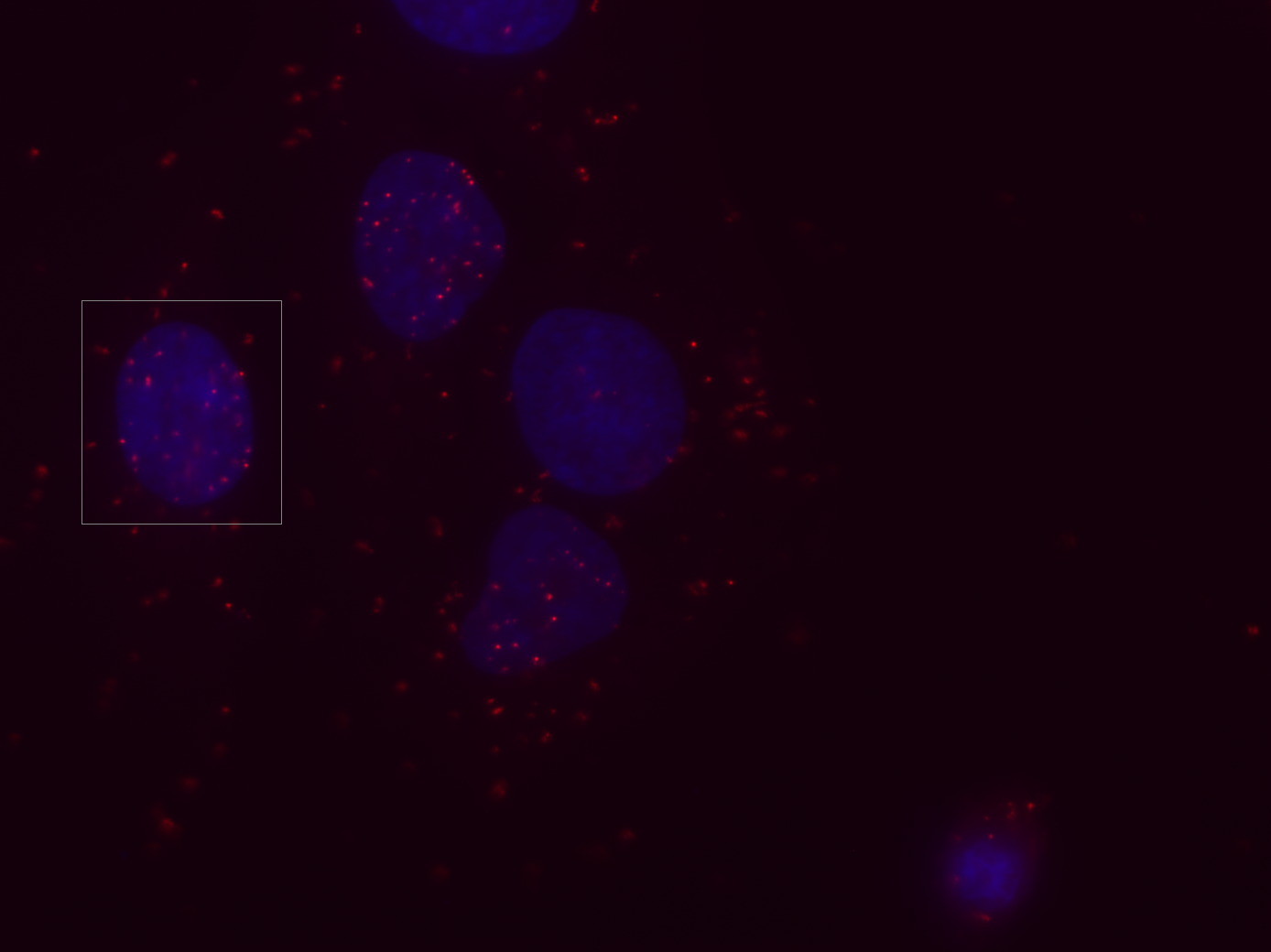

Supplement: Supplementary file 14 — Figure EV5 [file 44319_2025_497_MOESM14_ESM.zip › Figure EV 5C/shRNF20+TSA RAD51C SIRF.tif]

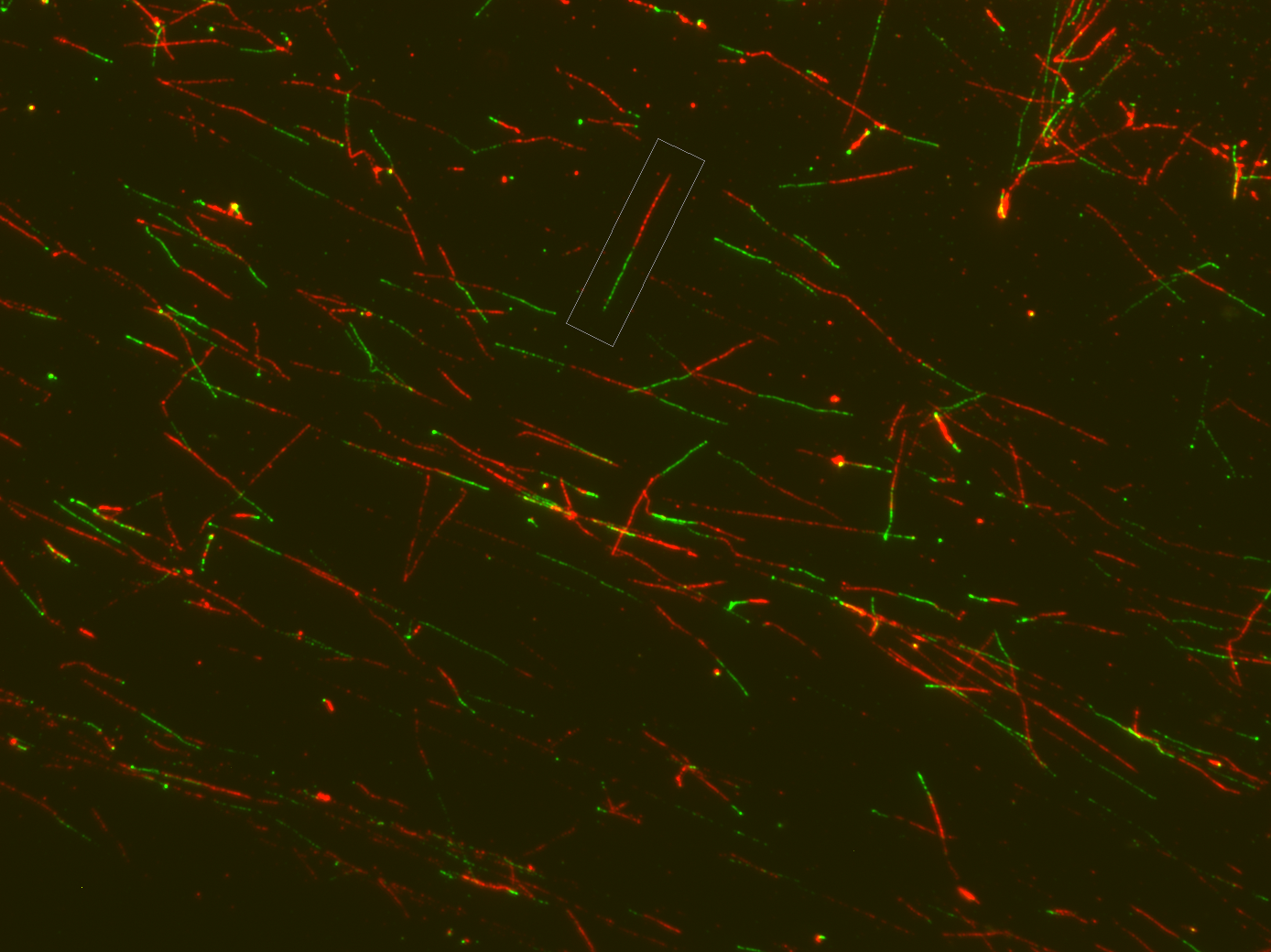

Supplement: Supplementary file 14 — Figure EV5 [file 44319_2025_497_MOESM14_ESM.zip › Figure EV 5F/shControl DNA fiber fork restart.tif]

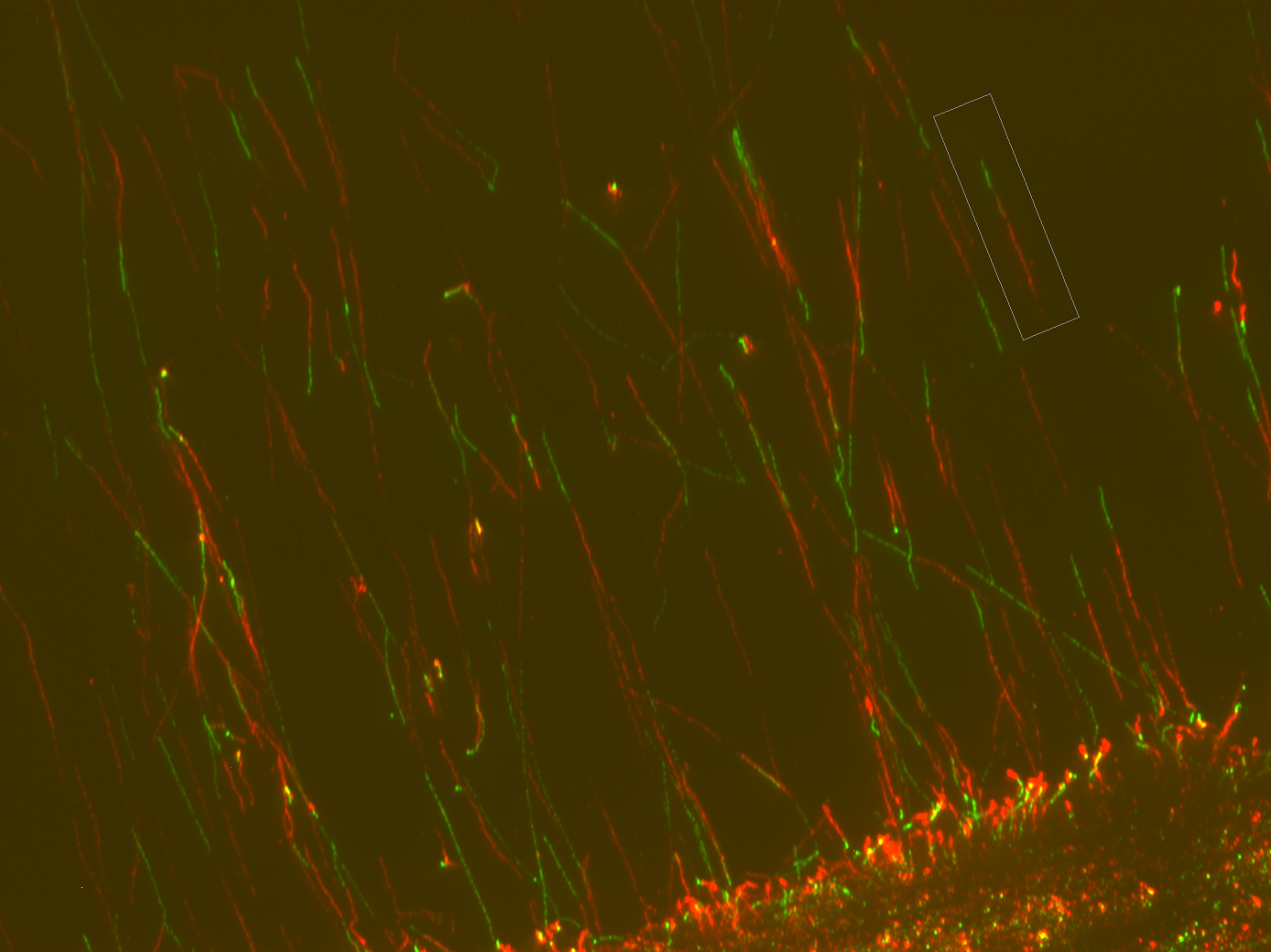

Supplement: Supplementary file 14 — Figure EV5 [file 44319_2025_497_MOESM14_ESM.zip › Figure EV 5F/shRNF20 DNA fiber fork restart.tif]

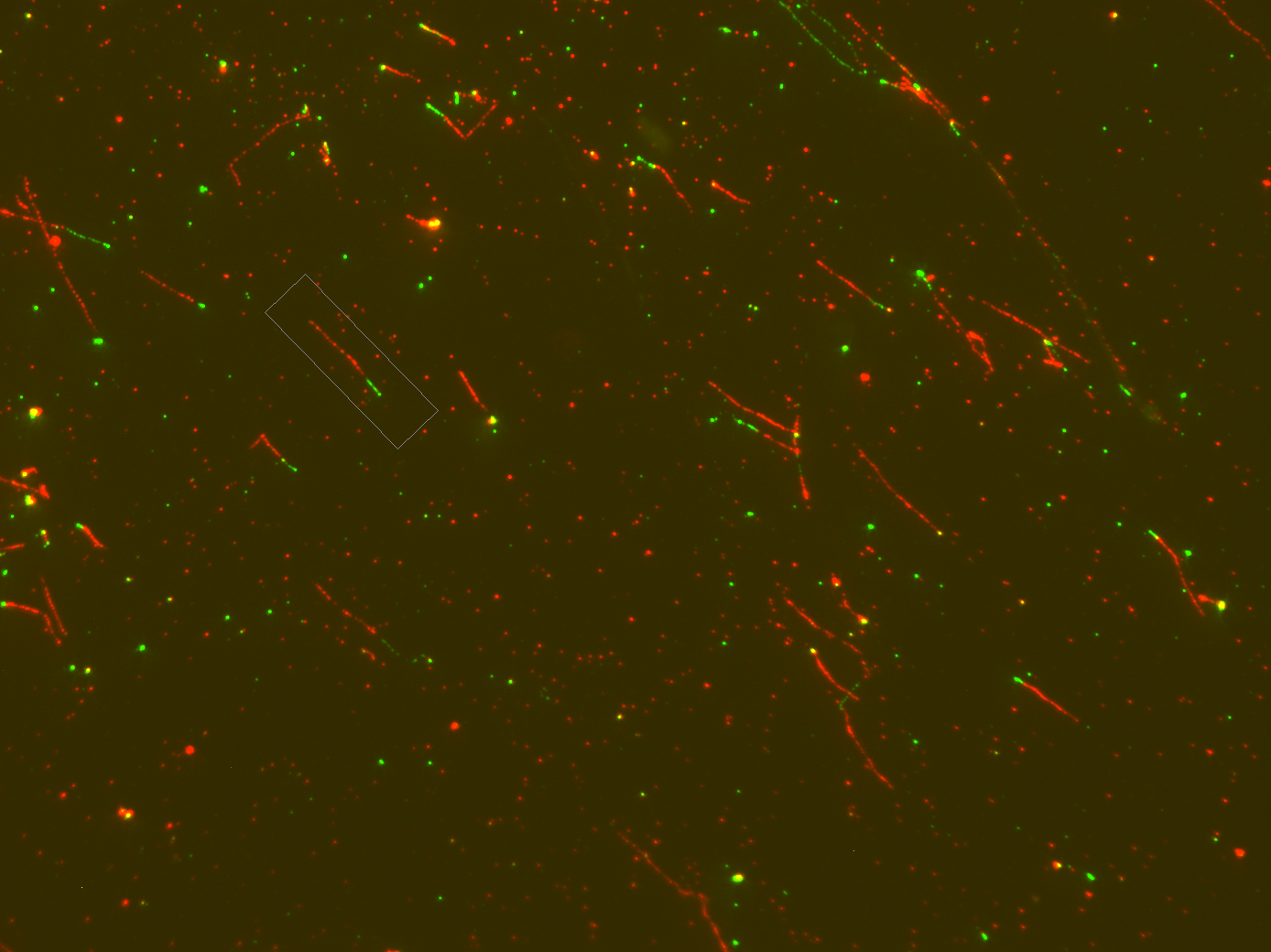

Supplement: Supplementary file 14 — Figure EV5 [file 44319_2025_497_MOESM14_ESM.zip › Figure EV 5F/shRNF20+S172A DNA fiber fork restart.tif]

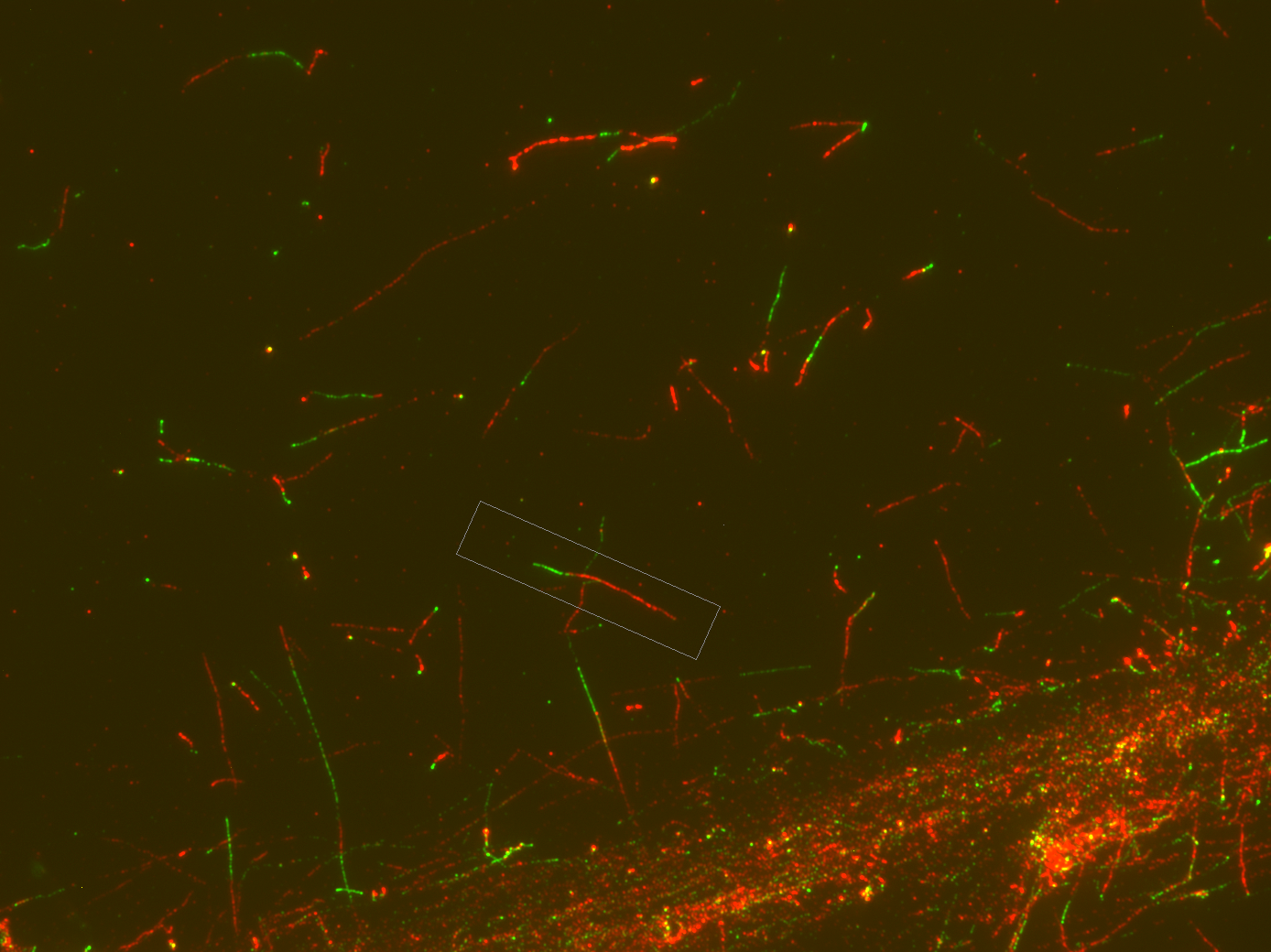

Supplement: Supplementary file 14 — Figure EV5 [file 44319_2025_497_MOESM14_ESM.zip › Figure EV 5F/shRNF20+S553A DNA fiber fork restart.tif]

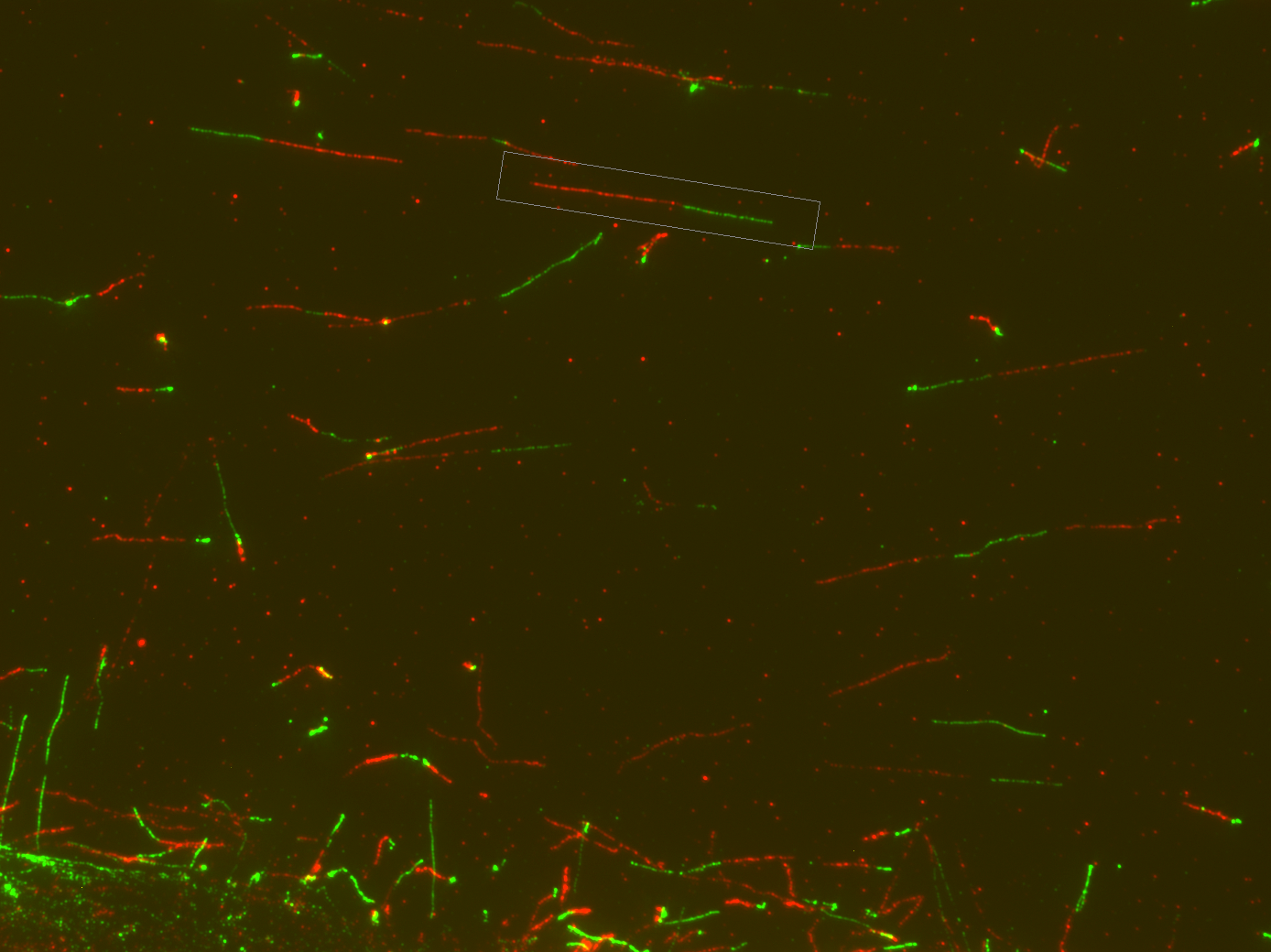

Supplement: Supplementary file 14 — Figure EV5 [file 44319_2025_497_MOESM14_ESM.zip › Figure EV 5F/shRNF20+WT DNA fiber fork restart.tif]
