# Supplementary material for: RNF20-mediated H2B monoubiquitination protects stalled forks from degradation and promotes fork restart
Source: EMBO Rep. 2025 Jun 10;26(15):3773–803. doi: 10.1038/s44319-025-00497-3 (PMC12331980; doi:10.1038/s44319-025-00497-3)
Supplement: Supplementary file 15 — Expanded View Figures [file 44319_2025_497_MOESM15_ESM.pdf]

Expanded View Figures

Figure EV1. Loss of RNF20 leads to genomic instability in U2OS cells.

(**A**) Representative images showing FANCD2 foci formation in control and RNF20-depleted mitotic U2OS cells following 22 h Aphidicolin treatment (0.4 µM) with RO3306 added in the last 6 h. Scale bar = 5 µm. Dotted white line indicates the nuclear content of the cells. (**B**) Quantified box and whiskers plot of FANCD2 foci per cell as indicated in (**A**). Mid-lines represent the medians and + represents mean values. Bounds of box represent 25th and 75th percentiles, whiskers represent 5–95 percentile. Total of ≥70 cells were analyzed for each condition from three experiments. Unpaired *t* test, $^{**}P < 0.01$. P (shRNF20 vs. shControl) = 0.0081. (**C**) Representative images showing pRPA32 S4/8 intensity in control and RNF20-depleted U2OS cells following 6 h recovery from HU (4 mM, 2 h) treatment. Scale bar = 5 µm. Dotted white line indicates the nuclear boundary of cells. (**D**) Quantitative bar plot of pRPA32 S4/8 intensity per cell in cells as indicated in (**C**). Data represents mean ± SEM from three independent experiments. Total of ≥300 cells were analyzed for each condition. Two-way ANOVA, $^{*}P < 0.05$; $^{**}P < 0.01$. P (shControl UT vs. shRNF20 UT) = 0.0144, P (shControl HU vs. shRNF20 HU) = 0.0014. (**E**) Representative images showing BrdU foci in the indicated cells after BrdU incorporation for 48 h followed by HU treatment (4 mM, 2 h). Scale bar = 5 µm. Dotted white line indicates nuclear boundary of the cells. (**F**) Quantitative bar plot showing percent of cells with BrdU foci in cells as shown in (**E**). Data represents mean ± SD from three independent experiments. Total of ≥250 cells were analyzed for each condition. Two-way ANOVA, $^{*}P < 0.05$. P (shControl UT vs. shRNF20 UT) = 0.0350, P (shControl HU vs. shRNF20 HU) = 0.0488 (**G**) Representative images for alkaline comet assay in the indicated U2OS cells, either left untreated or treated with 4 mM HU for 2 h. Scale bar = 5 µm. (**H**) Scatter plot showing quantitative analysis of comet tail moments in indicated cells. Total of ≥250 cells were analyzed for each condition. Black bars represent median values. Mann–Whitney test, $^{****}P < 0.0001$. P (shControl HU vs. shRNF20 HU) < 0.0001. (**I**) Representative images of metaphase spreads in the indicated cells with or without 2 mM HU treatment for 4 h. Arrows indicate breaks in chromosomes. Scale bar = 10 µm. (**J**) Quantitative plot showing number of chromosomal aberrations per spread, including breaks, gaps and radials, in cells as shown in (**I**). A minimum of 50 spreads were calculated for each sample. Data represents mean ± SD from three independent experimental repeats. Mann–Whitney test, $^{****}P < 0.0001$. P (shControl HU vs. shRNF20 HU) < 0.0001. Source data are available online for this figure.

 © The Author(s)

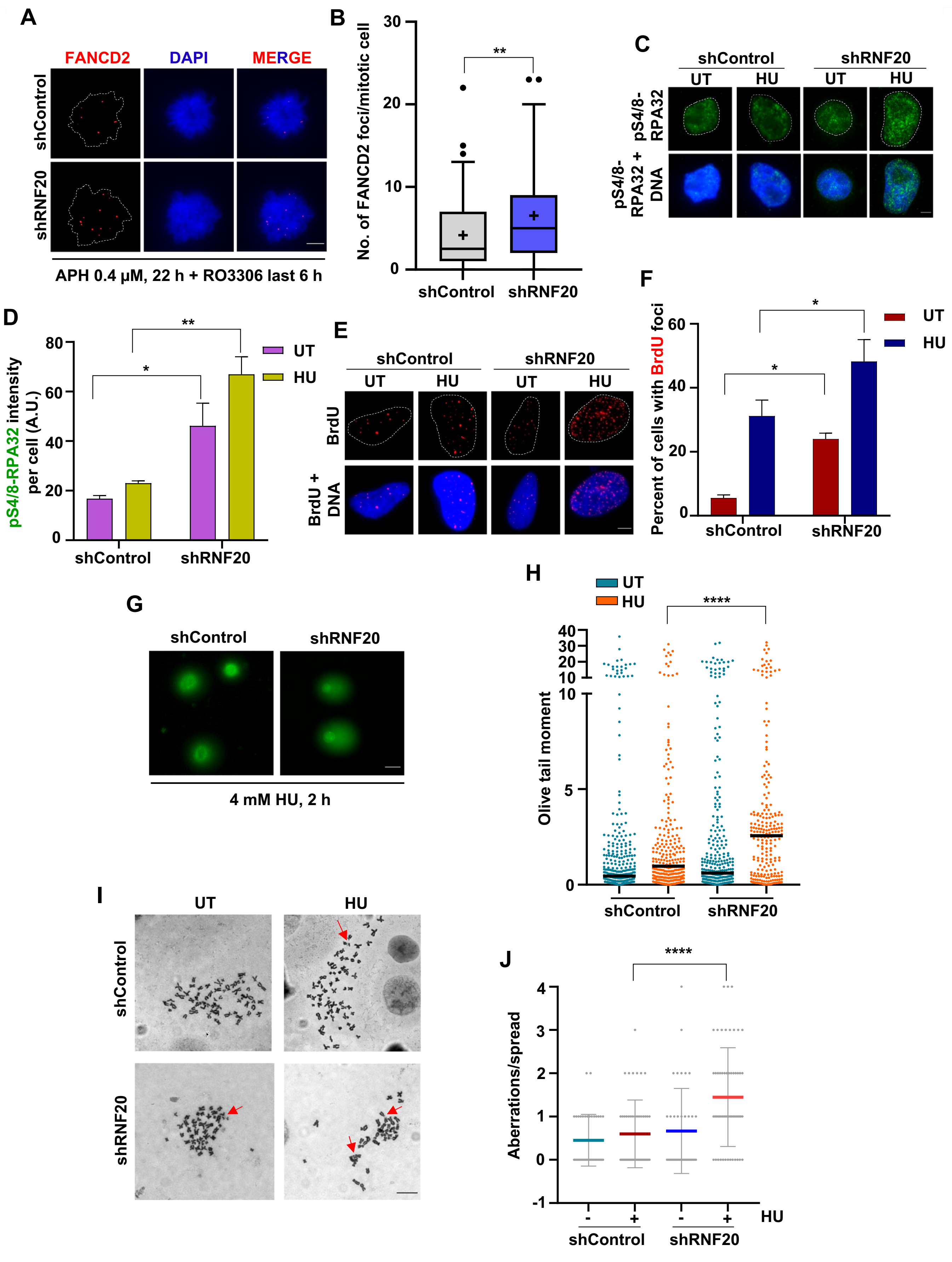
A
FANCD2
DAPI
MERGE
shControl
shRNF20
APH 0.4 μM, 22 h + RO3306 last 6 h
B
No. of FANCD2 foci/mitotic cell
shControl
shRNF20
C
shControl
shRNF20
UT
HU
pS4/8-RPA32
pS4/8-RPA32 + DNA
D
pS4/8-RPA32 intensity per cell (A.U.)
UT
HU
E
BrdU
BrdU + DNA
F
Percent of cells with BrdU foci
G
4 mM HU, 2 h
H
Olive tail moment
I
J
Aberrations/spread
HU

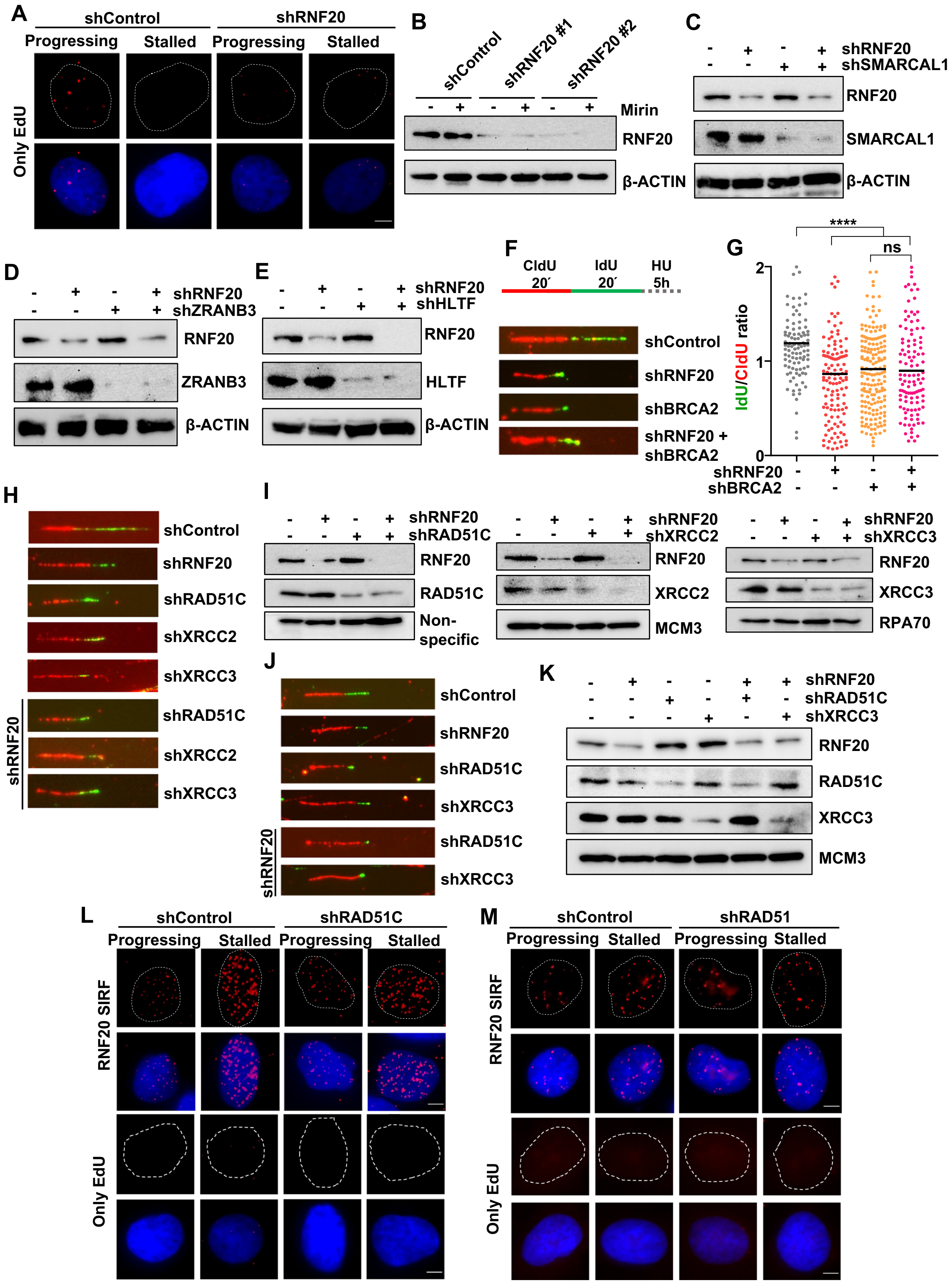
A
shControl
shRNF20
Progressing
Stalled
Progressing
Stalled
Only EdU
B
shControl
shRNF20 #1
shRNF20 #2
Mirin
RNF20
β-ACTIN
C
shRNF20
shSMARCAL1
RNF20
SMARCAL1
β-ACTIN
D
shRNF20
shZRANB3
RNF20
ZRANB3
β-ACTIN
E
shRNF20
shHLTF
RNF20
HLTF
β-ACTIN
F
CldU 20´
IdU 20´
HU 5h
shControl
shRNF20
shBRCA2
shRNF20 + shBRCA2
G
IdU/CldU ratio
****
ns
shRNF20
shBRCA2
H
shControl
shRNF20
shRAD51C
shXRCC2
shXRCC3
shRNF20
shRAD51C
shXRCC2
shXRCC3
I
shRNF20
shRAD51C
RNF20
RAD51C
Non-specific
shRNF20
shXRCC2
RNF20
XRCC2
MCM3
shRNF20
shXRCC3
RNF20
XRCC3
RPA70
J
shControl
shRNF20
shRAD51C
shXRCC3
shRNF20
shRAD51C
shXRCC3
K
shRNF20
shRAD51C
shXRCC3
RNF20
RAD51C
XRCC3
MCM3
L
shControl
shRAD51C
Progressing
Stalled
Progressing
Stalled
RNF20 SIRF
Only EdU
M
shControl
shRAD51
Progressing
Stalled
Progressing
Stalled
RNF20 SIRF
Only EdU

 © The Author(s)

Figure EV2. RNF20 is epistatic to RAD51 paralogs in stalled fork protection and restart.

(**A**) Representative images showing negative control of H2B K120ub SIRF (only EdU) for progressing and stalled replication sites in control and RNF20-depleted U2OS cells. Scale bar = 5 µm. Dotted white line indicates nuclear boundary of the cells. (**B**) Western blot showing depletion of RNF20 in U2OS cells transfected with shRNF20 #1 and #2 compared to shControl transfected cells with or without mirin treatment. (**C**) Western blot showing co-depletion of RNF20 and SMARCAL1 in U2OS cells. (**D**) Western blot showing co-depletion of RNF20 and ZRANB3 in U2OS cells. (**E**) Western blot showing co-depletion of RNF20 and HLTF in U2OS cells. (**F**) Scheme of DNA fiber labeling protocol (top) and representative DNA fibers showing fork stability in the indicated cells after 4 mM HU treatment for 5 h. (**G**) Quantitative scatter plot of IdU to CldU tract length ratio in cells as shown in (**F**). A total of ≥100 fibers were calculated for each sample from three experiments. Black bars represent median values. Mann–Whitney test, ****$P < 0.0001$. ns, non-significant. P (shControl vs. shRNF20; shControl vs. shBRCA2; shControl vs. shRNF20 + shBRCA2) < 0.0001, P (shRNF20 vs. shRNF20 + shBRCA2) = 0.4327. (**H**) Representative DNA fibers showing fork stability in the indicated cells treated with 4 mM HU for 5 h. (**I**) Western blot showing single and co-depletions of RNF20, RAD51C, XRCC2 and XRCC3 in U2OS cells transfected with the indicated shRNAs. (**J**) Representative DNA fibers showing replication fork restart in the indicated U2OS cells after release from 2 mM HU treatment for 2 h. (**K**) Western blot showing single and co-depletions of RNF20, RAD51C and XRCC3 in U2OS cells transfected with the indicated shRNAs. (**L**) Representative images of RNF20 SIRF signals at progressing and stalled replication forks in control and RAD51C-depleted U2OS cells. Scale bar = 5 µm. Dotted white line indicates the nuclear boundary of cells. (**M**) Representative images of RNF20 SIRF signals at progressing and stalled replication forks in control and RAD51-depleted U2OS cells. Scale bar = 5 µm. Dotted white line indicates the nuclear boundary of cells. Source data are available online for this figure.

© The Author(s)

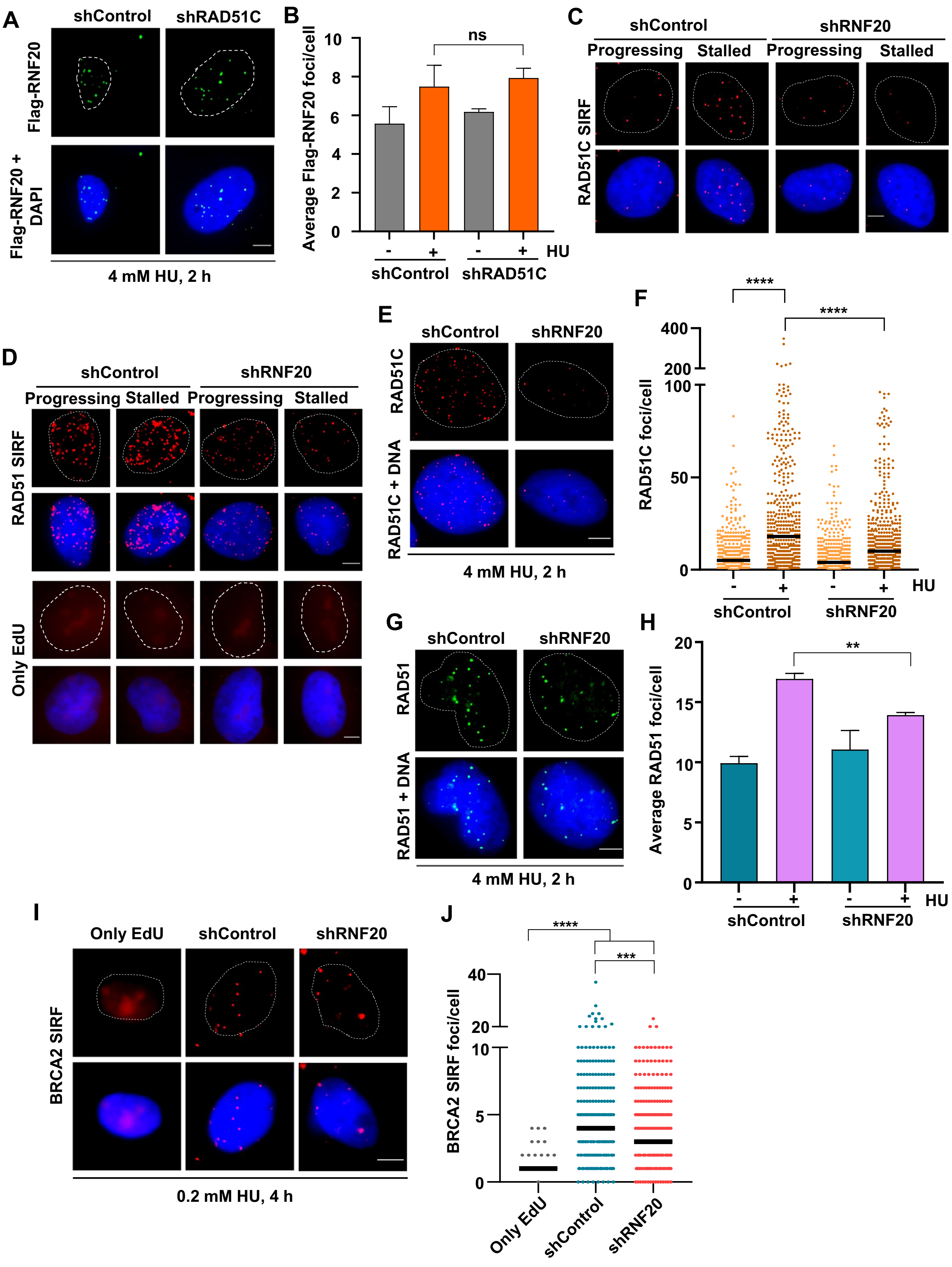
A
shControl
shRAD51C
Flag-RNF20
Flag-RNF20 + DAPI
4 mM HU, 2 h
B
Average Flag-RNF20 foci/cell
ns
10
8
6
4
2
0
- + - + HU
shControl shRAD51C
C
shControl
shRNF20
Progressing Stalled Progressing Stalled
RAD51C SIRF
D
shControl
shRNF20
Progressing Stalled Progressing Stalled
RAD51 SIRF
Only EdU
E
shControl
shRNF20
RAD51C
RAD51C + DNA
4 mM HU, 2 h
F
RAD51C foci/cell
****
****
400
200
100
50
0
- + - + HU
shControl shRNF20
G
shControl
shRNF20
RAD51
RAD51 + DNA
4 mM HU, 2 h
H
Average RAD51 foci/cell
**
20
15
10
5
0
- + - + HU
shControl shRNF20
I
Only EdU
shControl
shRNF20
BRCA2 SIRF
0.2 mM HU, 4 h
J
BRCA2 SIRF foci/cell
****
***
40
20
10
5
0
Only EdU
shControl
shRNF20

 © The Author(s)

Figure EV3. RNF20 facilitates the recruitment of RAD51/RAD51 paralogs to the stalled replication sites.

(**A**) Representative images showing Flag-RNF20 foci in control and RAD51C depleted U2OS cells after 4 mM HU treatment for 2 h. Scale bar = 5 μm. Dotted white line indicates the nuclear boundary of cells. (**B**) Quantitative bar plot showing average Flag-RNF20 foci per cell in cells as shown in (**A**). Data represents mean ± SEM from three independent experiments. Total of ≥250 cells were analyzed for each condition. Unpaired *t* test, ns, non-significant. *P* (shControl HU vs. shRNF20 HU) = 0.7320. (**C**) Representative images of RAD51C SIRF signals in the indicated shRNA transfected U2OS cells in unperturbed (progressing) or HU treated (stalled) conditions. Scale bar = 5 μm. Dotted white line indicates the nuclear boundary of cells. (**D**) Representative images of RAD51 SIRF signals in the indicated shRNA transfected U2OS cells in unperturbed (progressing) or HU treated (stalled) conditions. Scale bar = 5 μm. Dotted white line indicates the nuclear boundary of cells. (**E**) Representative images showing RAD51C foci formation in control and RNF20-depleted U2OS cells during replication stress induced by 4 mM HU, 2 h treatment. Scale bar = 5 μm. Dotted white line indicates the nuclear boundary of cells. (**F**) Quantitative scatter plot showing RAD51C foci per cell for cells as shown in (**E**). Total of ≥300 cells were analyzed for each condition from three independent experiments. Black bars represent median. Mann–Whitney test, *****P* < 0.0001. *P* (shControl UT vs. shControl HU; shControl HU vs. shRNF20 HU) < 0.0001. (**G**) Representative images showing RAD51 foci formation in control and RNF20-depleted cells subjected to HU treatment (4 mM, 2 h). Scale bar = 5 μm. Dotted white line indicates the nuclear boundary of cells. (**H**) Quantification of average RAD51 foci per cell as shown in (**G**). Data represents mean ± SEM from three independent experiments. Total of ≥250 cells were analyzed for each condition. Unpaired *t* test, ***P* < 0.01. *P* (shControl HU vs. shRNF20 HU) = 0.0039. (**I**) Representative images of BRCA2 SIRF signals in the indicated shRNA transfected U2OS cells in HU treated (stalled) conditions. Scale bar = 5 μm. Dotted white line indicates the nuclear boundary of cells. (**J**) Quantitative scatter plot showing BRCA2 SIRF foci per cell for cells as shown in (**I**). Total of ≥300 cells were analyzed for each condition from three independent experiments. ≥110 cells were counted for only EdU control. Black bars represent median. Mann–Whitney test, ****P* < 0.001; *****P* < 0.0001. *P* (shControl vs. shRNF20) = 0.0001, *P* (Only Edu vs. shControl; Only Edu vs. shRNF20) < 0.0001. Source data are available online for this figure.

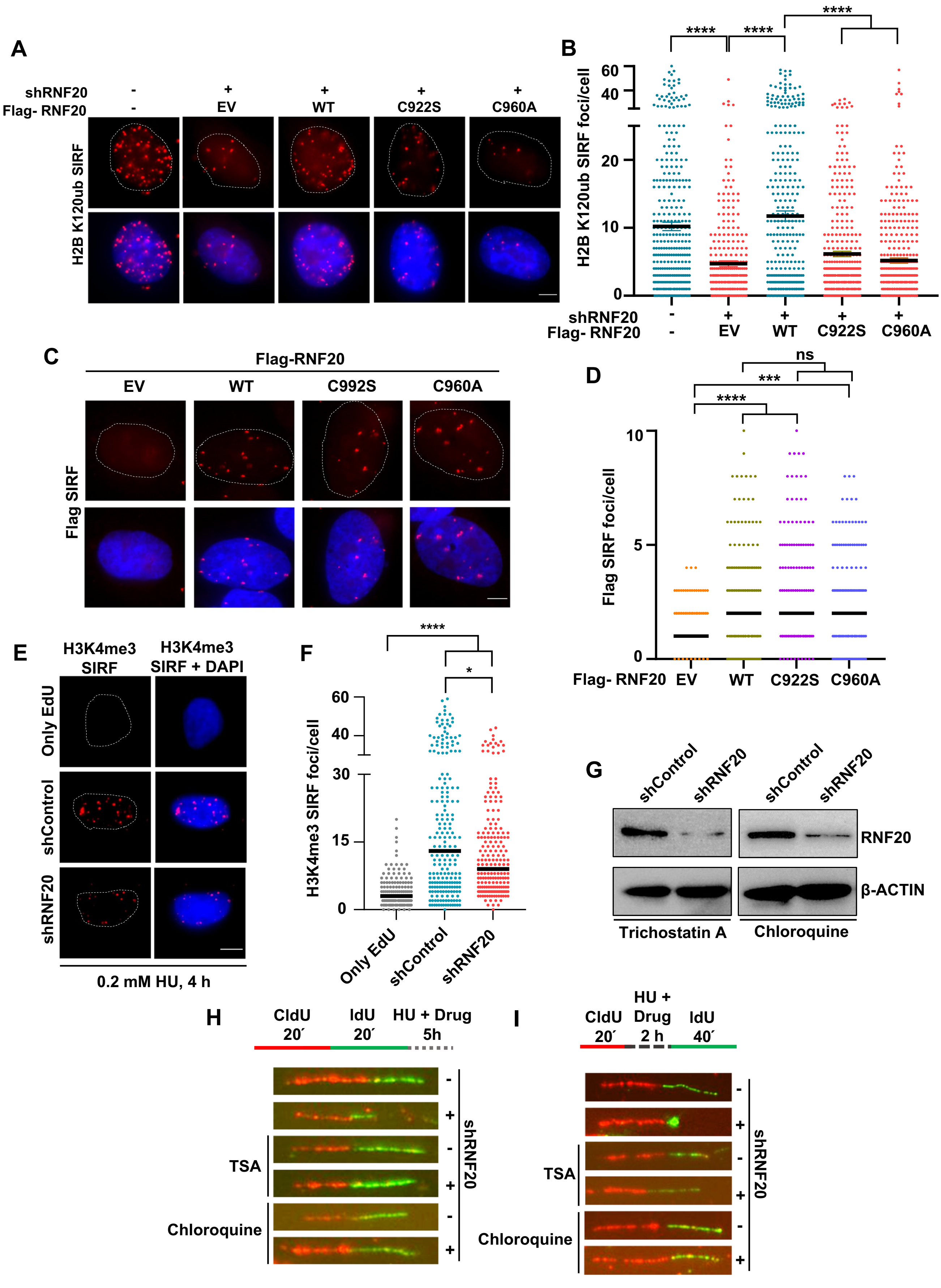

A
shRNF20 - + + + +
Flag- RNF20 - EV WT C922S C960A
H2B K120ub SIRF
B
H2B K120ub SIRF foci/cell
****
****
****
shRNF20 - + + + +
Flag- RNF20 - EV WT C922S C960A
C
Flag-RNF20
EV WT C992S C960A
Flag SIRF
D
ns
***
****
Flag SIRF foci/cell
Flag- RNF20 EV WT C922S C960A
E
H3K4me3 SIRF
H3K4me3 SIRF + DAPI
Only EdU
shControl
shRNF20
0.2 mM HU, 4 h
F
****
*
H3K4me3 SIRF foci/cell
Only EdU
shControl
shRNF20
G
shControl shRNF20 shControl shRNF20
RNF20
β-ACTIN
Trichostatin A
Chloroquine
H
CldU 20´
IdU 20´
HU + Drug 5h
TSA
Chloroquine
shRNF20
I
CldU 20´
HU + Drug 2 h
IdU 40´
TSA
Chloroquine
shRNF20

 © The Author(s)

Figure EV4. Catalytic activity of RNF20 is important for regulating chromatin dynamics at the stalled fork sites.

(**A**) Representative images depicting H2B K120ub SIRF signals in U2OS cells expressing either WT or C922S/C960A RNF20 after endogenous RNF20 depletion. Scale bar = 5 µm. Dotted white line indicates the nuclear boundary of cells. (**B**) Scatter plot quantification of H2B K120ub SIRF signals in cells as shown in (**A**). Total of ≥250 cells were analyzed for each condition from three independent experiments. Data represents mean ± SEM. Mann–Whitney *t* test, *****P* < 0.0001. *P* (shControl vs. shRNF20 + EV; shRNF20 + EV vs. shRNF20 + WT; shRNF20 + WT vs. shRNF20 + C922S; shRNF20 + WT vs. shRNF20 + C960A) < 0.0001. (**C**) Representative images of Flag-RNF20 SIRF signals in cells expressing WT or RING domain mutants of RNF20 at stalled fork sites. Scale bar = 5 µm. Dotted white line indicates the nuclear boundary of cells. (**D**) Scatter plot of Flag-RNF20 SIRF signals in cells as shown in (**C**). Total of ≥150 cells were analyzed for each condition from three independent experiments. Black bars represent median. Mann–Whitney *t* test, ****P* < 0.001; *****P* < 0.0001; ns, non-significant. *P* (EV vs. WT; EV vs. C922S) < 0.0001, *P* (EV vs. C960A) = 0.0003, *P* (WT vs. C922S) = 0.2296, *P* (WT vs. C960A) = 0.3429. (**E**) Representative images depicting H3K4me3 SIRF signals at stalled forks (0.2 mM HU, 4 h) in control and RNF20-depleted cells. Scale bar = 5 µm. Dotted white line indicates the nuclear boundary of cells. (**F**) Scatter plot quantification of H3K4me3 SIRF signals in cells as shown in (**E**). Total of ≥200 cells were analyzed for each condition from three independent experiments. Black bars represent median. Mann–Whitney *t* test, **P* < 0.05; *****P* < 0.0001. *P* (Only EdU vs. shControl; Only EdU vs. shRNF20) < 0.0001, (shControl vs. shRNF20) = 0.0122. (**G**) Immunoblot showing depletion of RNF20 in U2OS cells treated with HU (4 mM) + TSA (0.2 µM) or chloroquine (20 µg/ml) along with RNF20 shRNA. (**H**) DNA fiber labelling protocol (top) and representative DNA fibers showing fork protection in control and RNF20-depleted cells under HU (4 mM) and TSA (0.2 µM)/chloroquine (20 µg/ml) treatment. (**I**) DNA fiber labeling protocol (top) and representative DNA fibers depicting fork restart occurring in control and RNF20-depleted cells under HU (2 mM) and TSA (0.2 µM) or chloroquine (20 µg/ml) treatment. Source data are available online for this figure.

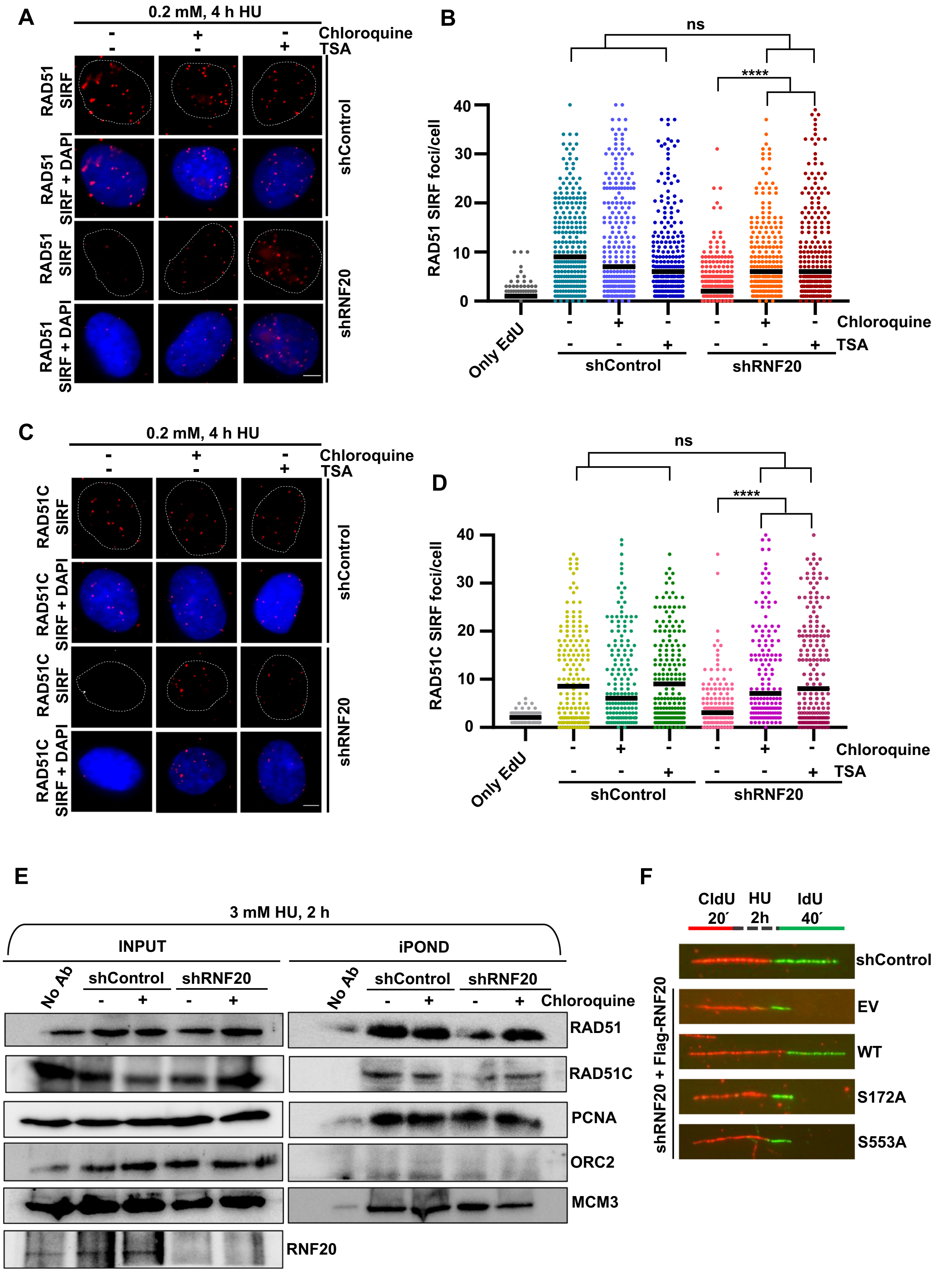
A
0.2 mM, 4 h HU
Chloroquine
TSA
RAD51 SIRF
RAD51 SIRF + DAPI
shControl
shRNF20
B
ns
****
RAD51 SIRF foci/cell
Only EdU
Chloroquine
TSA
shControl
shRNF20
C
0.2 mM, 4 h HU
RAD51C SIRF
RAD51C SIRF + DAPI
D
RAD51C SIRF foci/cell
E
3 mM HU, 2 h
INPUT
iPOND
No Ab
RAD51
RAD51C
PCNA
ORC2
MCM3
RNF20
F
CldU 20´
HU 2h
IdU 40´
shRNF20 + Flag-RNF20
EV
WT
S172A
S553A

Figure EV5. Chromatin relaxation rescues the recruitment of RAD51/RAD51 paralogs to stalled forks in RNF20-deficient cells.

(**A**) Representative images depicting RAD51 SIRF foci in control and RNF20-depleted U2OS cells treated with HU (0.2 mM) and TSA (0.2 µM) or chloroquine (20 µg/ml). Scale bar = 5 µm. Dotted white line indicates the nuclear boundary of cells. (**B**) Quantification of RAD51 SIRF foci in cells as shown in (**A**). Total of ≥250 cells were analyzed for each condition from three independent experiments. Black bars represent median. Mann–Whitney *t* test, *****P* < 0.0001. *P* (shRNF20 vs. shRNF20 + chloroquine; shRNF20 vs. shRNF20 + TSA) < 0.0001. Kruskal–Wallis test with multiple comparisons, ns: non-significant. *P* (shControl + chloroquine vs. shRNF20 + chloroquine) = 0.3593, *P* (shControl + TSA vs. shRNF20 + TSA) > 0.9999. (**C**) Representative images depicting RAD51C SIRF foci in control and RNF20-depleted U2OS cells treated with HU (0.2 mM) and TSA (0.2 µM) or chloroquine (20 µg/ml). Scale bar = 5 µm. Dotted white line indicates the nuclear boundary of cells. (**D**) Quantification of RAD51C SIRF foci in cells as shown in (**C**). Total of ≥150 cells were analyzed for each condition from three independent experiments. Data represents median. Mann–Whitney *t* test, *****P* < 0.0001. *P* (shRNF20 vs. shRNF20 + chloroquine; shRNF20 vs. shRNF20 + TSA) < 0.0001. Kruskal–Wallis test with multiple comparisons, ns: non-significant, *P* (shControl + chloroquine vs. shRNF20 + chloroquine) >0.9999, *P* (shControl + TSA vs. shRNF20 + TSA) > 0.9999. (**E**) Western blots showing localization of indicated proteins at stalled replication forks in control and RNF20-depleted cells by IdU-iPOND. Control and RNF20-depleted cells were pulsed with IdU followed by treatment with 3 mM HU for 2 h. IdU IP was performed and elutes were resolved by SDS-PAGE and blotted with indicated antibodies. (**F**) DNA fiber labeling protocol (top). Representative DNA fibers showing fork stalling and restart in the indicated U2OS cells after release from 2 mM HU, 2 h treatment (bottom).Source data are available online for this figure.
